# International symposium on peripheral nerve repair and regeneration and 2nd club Brunelli meeting

**DOI:** 10.1186/1749-7221-5-5

**Published:** 2010-03-09

**Authors:** Mehmet Turgut, Stefano Geuna

**Affiliations:** 1 Department of Neurosurgery, Adnan Menderes University School of Medicine, Aydın, Turkey; 2 Department of Clinical and Biological Sciences, San Luigi Gonzaga School of Medicine, University of Turin, Italy

## Abstract

The International Symposium "Peripheral Nerve Repair and Regeneration and 2nd Club Brunelli Meeting" was held on December 4-5, 2009 in Turin, Italy (Organizers: Bruno Battiston, Stefano Geuna, Isabelle Perroteau, Pierluigi Tos). Interest in the study of peripheral nerve regeneration is very much alive because complete recovery of nerve function almost never occurs after nerve reconstruction and, often, the clinical outcome is rather poor. Therefore, there is a need for defining innovative strategies for improving the success of recovery after nerve lesion and repair and this meeting was intended to discuss, from a multidisciplinary point of view, some of today's most important issues in this scientific field, arising from both basic and clinical neurosciences.

## Background

Interest in the study of peripheral nerve repair and regeneration has increased significantly over the last twenty years since, while in the past most nerve traumas and diseases were not surgically treated, today the number of nerve reconstructions performed is progressively increasing due to the continuous improvement in surgical technology and to the spread of microsurgical skills among surgeons worldwide. Unfortunately, in spite of the impressive technical advancements in nerve reconstruction, complete recovery and normalization of nerve function almost never occur and the clinical outcome is often poor. It can be thus expected that the increasing number of patients receiving nerve surgery will represent an important stimulus for more research in this scientific field.

## Key issues

**Figure 1:**
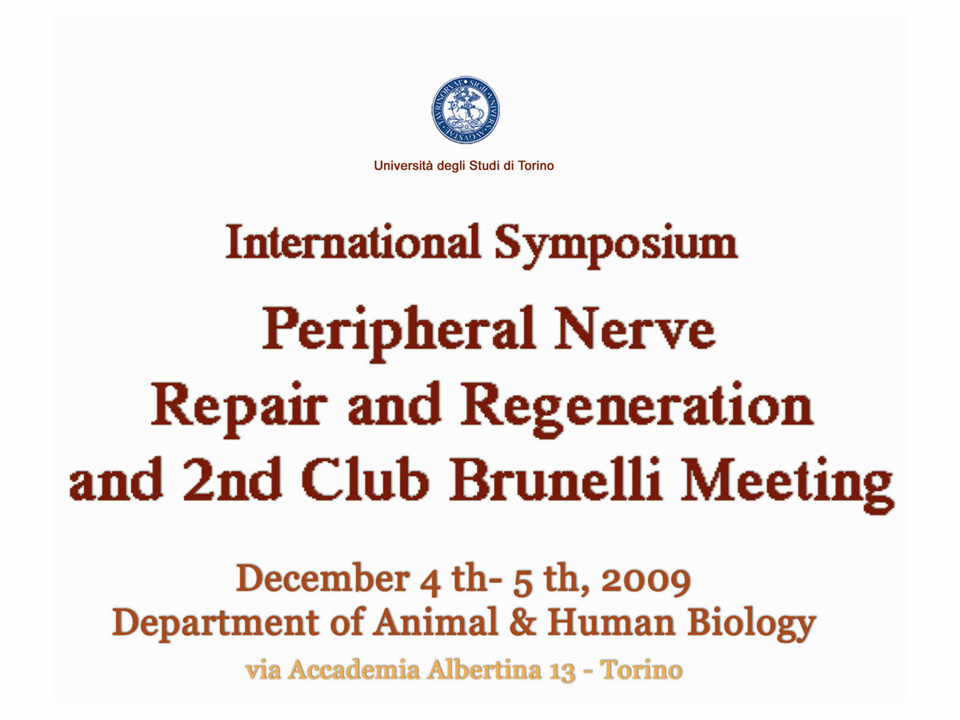
**Logo of the symposium**.

In line with this growing interest, the International Symposium "Peripheral Nerve Repair and Regeneration and 2nd Club Burnelli Meeting" was held at the Department of Animal and Human Biology of the University of Turin, Italy, on December 4-5, 2009 (figure 1) [1]. The topics covered along the symposium were: neurobiology of peripheral nerve regeneration, glial cells, tissue engineering, innovative strategies for promoting nerve regeneration, biomaterials and artificial conduits for nerve reconstruction, and clinical applications, such as tubulization and end-to-side neurorrhaphy. The international and multidisciplinary panel of papers addressed, from many points of view, the current knowledge on nerve repair and regeneration, from the basic mechanisms to the perspectives for defining innovative treatment strategies for improving nerve recovery in human and veterinary medicine. Both basic and clinical scientists with different background (including neurobiologists, neuroanatomists, neurologists, biomaterial engineers, neurosurgeons, plastic and reconstructive surgeons, hand surgeons and orthopedists) attended to the Symposium and a total of 47 oral talks were given by lecturers coming from various countries, including Austria, Croatia, Czech Republic, Germany, Greece, Israel, Italy, Portugal, Slovenia, Spain, Switzerland, The Netherlands, Turkey, and United Kingdom. Yet, a keynote lecture entitled "Holistic and Epistemologic Review of Peripheral Nerve Repair and Regeneration" was given by Professor Giorgio Brunelli who provided an overview about past, present and future of the most challenging topics in nerve repair and regeneration. In addition to oral presentations, a total of 15 poster presentations were exhibited.

The additional file 1 is the compressed PDF of the proceedings of the symposium [see additional file 1]. Furthermore, the additional file 2 contains the slides of the keynote lecture from Professor Giorgio Brunelli [see additional file 2].

## Conclusion

The symposium was organized as a *low cost event *and without registration fees, in order to facilitate the participation of younger scientists (PhD students, in-training clinicians, etc.), and total final attendance was much higher than expected reaching about 200 people. All scientific sessions were very active and the overall appreciation of the meeting was high among participants. There are thus plans to organize further events of this kind on a biannual basis.

## Competing interests

The authors declare that they have no competing interests.

## Authors' contributions

Both authors (MT & SG) contributed to the creation of the manuscript and have read/approved the final manuscript.

## Supplementary Material

Additional file 1 Compressed PDFs of the proceedings of the symposium. Click here for file

Additional file 2 Slides of oral presentation from the keynote lecture from Professor Giorgio Brunelli. Click here for file
