# Supplementary material for: International symposium on peripheral nerve repair and regeneration and 2nd club Brunelli meeting
Source: J Brachial Plex Peripher Nerve Inj. 2010 Mar 9;5:5. doi: 10.1186/1749-7221-5-5 (PMC2848046; doi:10.1186/1749-7221-5-5)
Supplement: Additional file 1 — Compressed PDFs of the proceedings of the symposium. [file 1749-7221-5-5-S1.PDF]

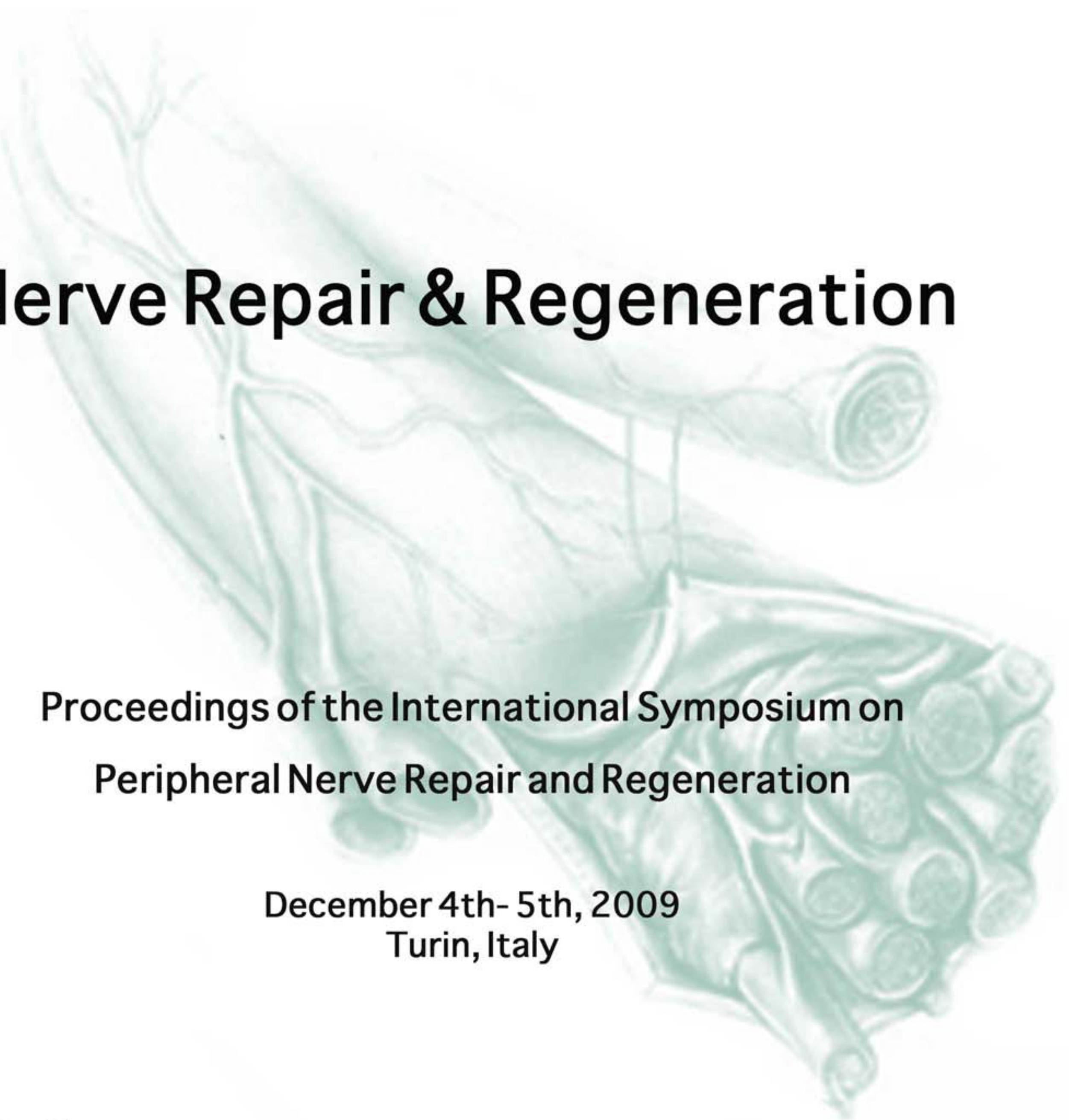

Nerve Repair & Regeneration

Proceedings of the International Symposium on Peripheral Nerve Repair and Regeneration

December 4th- 5th, 2009
Turin, Italy

Edited by:

Bruno Battiston
Stefano Geuna
Isabelle Perroteau
Pierluigi Tos

Università degli Studi di Torino

TABLE OF CONTENTS

THE NEUROBIOLOGY OF PERIPHERALNERVE REGENERATION

6 *"Modulated expression of antioxidant molecules during peripheral nerve regeneration"* C. Lanza, G. Ronchi, A. Voci, L. Vergani, N. Catena, F. Sénès, P. Tos, S. Geuna

7 *"Comparative proteomic profile of rat sciatic nerve and gastrocnemius muscle tissues in ageing by 2-D DIGE"* D. Capitanio, M. Vasso M, C. Fania, M. Moriggi, A. Viganò, P. Procacci, V. Magnaghi, C. Gelfi

8 "*Inhibition of RhoA promotes peripheral axon regeneration"* M. Auer, S. Frauscher, E. Udina, X. Navarro, L. Klimaschewski

9 *"JNK-AP1 pathway is involved in axonal growth potential and neuroregulation of CGRP dynamics in mice DRG after peripheral nerve injury"* G. Manassero, I. Repetto, F. Rossi, A. Vercelli

10 *"FGF signaling in adult sensory neurons promotes long-distance regeneration after axotomy"* L. Klimaschewski, B. Hausott

11 *"Dorsal root ganglia neuronal population increases over 40% as a consequence of a crush lesion of brachial plexus nerves in adult rats"* M. Fornaro, G. Ronchi, S. Raimondo, L. Muratori, I. Perroteau, S. Geuna, MG. Giacobini-Robecchi

12 *"Axonal regeneration interplay between GAP-43 and receptors of myelin inhibitory molecules"* S. Foscarin, S. Gianola, D. Carulli, P. Fazzari, S. Mi, L. Tamagnone, F. Rossi

14 "*Glutamate: a possible role in peripheral nerve damage?*" G. Cavaletti

GLIAL CELLS

14 *"Characterization of the Neonatal Olfactory Bulb Ensheathing Cell Line (NOBEC) in the perspective of its employment for experimental nerve tissue engineering"* R. Pellitteri, S. Geuna, S. Raimondo, C. Audisio, G. Gambarotta, D. Zaccheo, I. Perroteau

15 *"Isolation method for a stem cell population with neural potential from skin and adipose tissue in the perspective of peripheral nerve reconstruction"* V. Vindigni, B. Zavan, L. Lancerotto, F. Bassetto, G. Abatangelo

16 "*GABA receptors in adult stem cells after differentiation into Schwann-like cells: a possible target to promote nerve repair?"* A. Faroni, V. Magnaghi, G. Terenghi

17 *"Schwann-cell transplantation and electrical stimulation to increase peripheral nerve regeneration across long gaps"* K. Haastert, D. Klode, R. Schmitte, N. Korte, C. Grothe

18 *"Schwann cell like differentiated adipose derived stem cells enhance peripheral nerve regeneration in vivo"* P.G. Di Summa, P.J. Kingham, W. Raffoul, M. Wiberg, G. Terenghi, D.F. Kalbermatten

20 *"Transplantation of olfactory ensheathing cells enhances peripheral nerve regeneration after microsurgical nerve repair"* C. Radtke, A.A. Aizer, S.K. Agulian, K.L. Lankford, P.M. Vogt, J.D. Kocsis

TISSUE ENGINEERING AND STRATEGIES FOR PROMOTING NERVE REGENERATION

20 *"Rodent sciatic nerve-crush injuries: injury and recovery assessment"* R. Pavic, M. Pavic

20 *"Sciatic nerve injury: functional recovery and split plot differences in rat and mouse models"* R. Pavic, M. Pavic

21 *"Forelimb vs hindlimb nerve regeneration experimental models"* P. Tos, I. Papalia, G. Ronchi, I. Perroteau, B. Battiston, S. Geuna

22 *"Promoting regeneration of the injured peripheral nerve by combining neurosurgical repair with gene therapy"* J. Verhaagen, M. Tannemaat, R. Eggers, M. Mason, M. Malessy

23 *"Gene therapy for promoting nerve regeneration in rats"* F. Novati, G. Papa, L. Zentilin, S. Zacchigna, S. Moimas, M. Giacca, F. Fregnan, G. Gambarotta, I. Perroteau, S. Geuna, S. Raimondo, Z.M. Arnež

24 *"Activity-dependent strategies to enhance peripheral nerve regeneration and neural plasticity"* E. Udina, E. Asensio-Pinilla, X. Navarro

25 *"New insights on neuroactive steroids in the peripheral nerve"* R.C. Melcangi

26 *"The effect of melatonin and platelet gel on sciatic nerve repair: an electrophysiological and stereological study"* S. Kaplan, A. Piskin, M. Ayyildiz, A. Aktaş, B. Koksal , M. Basak-Ulkay, A.P. Turkmen, A. Turkmen, F. Bakan

27 *"Des-acyl ghrelin promotes peripheral nerve regeneration in a transgenic mouse model"* A. Graziani, N. Filigheddu, P. Porporato, C. Audisio, I. Perroteau, G. Ronchi, S. Geuna, S. Raimond

27 *"Phototherapy in peripheral nerve injury for muscle preservation and nerve regeneration"* S. Rochkind, S. Geuna, A. Shainberg

28 *"Changes in inhibitory cortical neurons induced by electrical stimulation of a transected peripheral nerve"* C. Herrera-Rincon, A. Sanchez-Jimenez, C. Avendaño, F. Panetsos

29 *"Reconnecting skeletal muscle to health spinal cord"* M. Pizzi, M. Francolini, S. Barlati, B. Guarneri, M. Buffelli, P. Spano, F. Clementi, G. Brunelli

BIOMATERIALS AND ARTIFICIAL CONDUITS FOR NERVE RECONSTRUCTION

30 *"Bridging peripheral short nerve defects with diverse sort of conduits. An overview of experimental novelties and clinical examples"* I.A. Ignatiadis

31 *"Natural based materials as scaffold for peripheral nerve regeneration"* C. Tonda-Turo, C. Audisio, P. Gentile, V. Chiono, S. Geuna, I. Perroteau, G. Ciardelli

33 *"Development of a new multichannel biodegradable conduit for peripheral nerve regeneration using nano-composite polymer"* A. Pabari, T. Sedaghati, S. Yu Yang, A. Mosahebi, A.M. Seifalian

34 *"In vivo evaluation of polysialic acid as candidate substance for the development of new nerve graft materials"* C. Grothe, Schaper-Rinkel, B. Rode, R. Gerardy-Schahn, T. Scheper, K. Haastert

35 *"Polymeric micro-channel electrodes for improved recording in peripheral nerve implant"* S.P. Lacour, J.J. FitzGerald, N. Lago, S. Benmerah, E. Tarte, S. McMahon, J. Fawcett

36 *"Biomaterials and cellular systems used for nerve regeneration"* M.J. Simões, S. Amado, A. Gärtner, P.A.S. Armada da Silva, S. Raimondo, M. Vieira, A.L. Luís, A.P. Veloso, A.S.P. Varejão, S. Geuna, A.C. Maurício

37 *"Biodegradable hydrogels as scaffolds for nerve regeneration"* V. Magnaghi, E. Ranucci, F. Fenili, P. Procacci, G. Pivato, P. Cortese, P. Ferruti

38 *"Tissue engineered guided regenerative gel for recovery of peripheral nerve injury with massive loss defect"* S. Rochkind, M. Alon, M. Graif, A. Shahar, Z. Nevo

CLINICAL APPLICATIONS: BIOLOGICAL TUBULIZATION AND END-TO-SIDE NERVE REPAIR

38 *"Ciliary neurotrophic factor induces more extensive collateral sprouting of motor than of afferent axons associated with improved functional reinnervation of the biceps muscle in an experimental model of end-to-side neurorrhaphy"* P. Dubový, P. Haninec, O. Raska, L. Stejskal, P. Celakovský

40 *"Longitudinal retrograde tracer study of sensory axonal ingrowth into end-to-side coapted nerve stump in the rat"* T. Žele, U. Kovačič, J. Sketelj, F.F. Bajrović

41 *"End-to-side nerve repair in brachial plexus and digital nerves: clinical experience"* B. Battiston, S. Artiaco, L.G. Conforti, P. Cartesegna, P. Tos

41 *"Use of end-to-side anastomosis in brachial plexus injury"* P. Haninec, P. Dubový, L. Mencl, R. Kaiser, L. Houšťava

42 *"Nerve repair by denatured muscle autografts promotes sustained sensory recovery in leprosy"* J.H. Pereira, D.D. Palande, T.S. Narayanakumar, A.S. Subramanian, S. Gschmeissner, M. Wilkinson

42 *"Nerve repair by fresh muscle-vein-combined nerve guides: clinical results and actual indications"* P. Tos, S. Artiaco, D. Ciclamini, E. Boux, L.G. Conforti, B. Battiston

43 *"Baby sitting procedures in nerve repair: a case of a proximal ulnar lesion"* G**.** Delia, M. Galeano, G. Risitano, F. Stagno d'Alcontres, M.R. Colonna

43 *"Late reinnervation in obstetrical brachial plexus palsy"* F.M. Senes, N. Catena

44 *"Neuroprotective effects of Melatonin on peripheral nerve regeneration"* M. Turgut

44 *"Patho-morphosis of the upper limb from nervous damage to the light of system theory and principle of regression"* G.M. Grippi

45 *"Aging impairs the reinnervation after end-to-side nerve repair in the rat"* U. Kovačič, T. Žele, J. Sketelj, F.F. Bajrović.

46 *"Tissue engineering of peripheral nerves: The need for an interdisciplinary approach"* S. Raimondo, G. Ronchi, P. Tos, S. Geuna, M. Fornaro, G. Gambarotta, I. Perroteau, M.G. Giacobini-Robecchi, B. Battiston

47 *"Bridging laboratory data to clinical applications for promoting peripheral nerve regeneration: Pitfalls and perspectives" S.* Geuna, P. Tos, I. Papalia, I. Perroteau, B. Battiston

POSTER PRESENTATIONS

47 *"In vitro comparison of motor and sensory neurons outgrowth in a 3D collagen type I matrix*" I. Allodi, M. Guzman-Lenis, X. Navarro, E. Udina

49 *"Nerve decompression does not allow sciatic function reestablishment after chronic constriction injury in the rat"* S. Amado, A. Mazzuco, A.C. Maurício, A. Veloso, S. Raimondo, S. Geuna, P. Armada-da-Silva

50 "*Effects of mesenchymal stem cells on peripheral nerve repair"* B. Ayas, P. Gürgör, Z. Erişgin, A. Korkmaz, M. Çifci

51 "*Morphological and functional modifications of the nervous system in a rat model of oxaliplatin-dependent neuropathy*" L. Bonaccini, L. Di Cesare Mannelli, C. Ghelardini, A. Pacini

52 *"Cyanoacrylate glue versus microsuturation in peripheral nerve anastomosis using histomorphometric and tensiometric analysis"* Y.A. Burhanoglu, K.A. Sargin, B. Can, O. Evirgen, I. Karabulut, M.N. Koc, D. Balkanci

53 *"Layer-by-layer coating with photoactive copolymers in biomedical applications"* I. Carmagnola, V. Chiono, C. Tonda-Turo, P. Gentile, F. Boccafoschi, G. Georgiev, V. Georgieva, G. Ciardelli

55 *"Short-lasting but not long-lasting treadmill exercise counteracts neuropathic pain and speed-up functional recovery after peripheral nerve injury"* S. Cobianchi, S. Luvisetto, S. Marinelli, F. Florenzano, F. Pavone ***(Roma, ITALY)****.*

56 *"Ultrastructural study of the effects of FK506 administration to the rat sciatic nerve"* O. Evirgen, F. Topal, O. Semiz, M. Akbari, A. Köse Sargın, B. Can

58 *"Axonotmesis-related changes in sensory neurons"* L. Muratori, A. Cunotto, S. Raimondo, G. Ronchi, S. Geuna, M. Fornaro

58 *"Significance of gp130 signaling in neuronal regeneration"* S. Quarta, N. Scherbakov, M. Andratsch, M. Kress

59 *Peripheral glial cell differentiation from neurospheres derived from adipose mesenchymal stem cells.* C. Radtke, B. Schmitz, M. Spies, J.D. Kocsis, P.M. Vogt

59 *"Median nerve regeneration is increased in ErbB2 transgenic mice"* P. Salamone, F. Di Scipio, G. Ronchi, A.E. Sprio, P. Tos, S. Geuna, G.N. Berta

60 *"Oxidative stress and effect of antioxidant treatment in an animal model of compressive neuropathy"* D. Tomassoni, L. Di Cesare Mannelli, C. Ghelardini, F. Amenta

61 *"Nanofibrous matrices for nerve regeneration"* C. Tonda-Turo, V. Chiono, E. Cipriani, C. Audisio, S. Geuna, M. Zanetti, I. Perroteau, G. Ciardelli

63 *"A new technique of autogenous conduits for bridging short nerve defects. An experimental study in the rabbit***"** V. Tsiampa, I. Ignatiadis, S. Galanakos, A. Avram, A. Papalois

Modulated expression of antioxidant molecules during peripheral nerve regeneration

C. Lanza,[1] G. Ronchi,[2] A. Voci,[1] L. Vergani,[1] N. Catena,[3] F. Sénès,[3] P. Tos,[4] S. Geuna[2]

[1]Department of Biology, University of Genoa, Genoa, [2]DSCB, University of Turin, [3]Orthopedics Unit, IRCCS Giannina Gaslini Institute, Genoa, and [4]Reconstructive Microsurgery Unit, CTO Hospital, Torino, Italy

This study focused on the expression of molecules orchestrating the response of peripheral nerves to axonal injury in the mouse model system. Peripheral nerve injury produces functional changes in lesioned neurons in which oxidative stress is considered to be the main cause of neuronal damage (Zochodne & Levy, 2005). The competence of neurons to regenerate depends on their ability to initiate a program of gene expression supporting growth, but also on the activity of glial cells in the distal stump of the injured nerve (De la Hoz et al., 2003). Cells and tissues are equipped with a variety of enzymatic and non-enzymatic antioxodants to remove the excess of ROS and protect against oxidative injury (Hensley, 2000). Among the non-enzymatic systems, metallothioneins (MTs) are the most important in nervous system. MTs are a class of low-molecular weight (6-7 kDa) metal-binding proteins involved in scavenging of free radicals (Chung and West, 2004), storage and metabolism of essential metals and detoxification of toxic metals (Vergani et al., 2009).

In the present study changes in mRNA expression of antioxidant and stress-associated molecules have been evaluated during regeneration of the injured rat peripheral median nerve. Yet, the nerve morphology in the different experimental conditions was assessed by high resolution light microcopy.

The median nerve was cut and the transcriptional changes were studied at day 6 and day 12 after injury in both the proximal and distal stumps, in the absence or in the presence of end-to-end microsurgical repair. The expression profiles of three metallothionein isoforms (MT-1, MT-2 and MT-3) as well as of the main antioxidant enzymes (catalase, superoxide dismutase and glutathione-S-transferase) and of typical markers of cellular damage (poly(ADP-ribose) polymerase-1-PARP-1) were evaluated.

Results indicated that different patterns of gene expression emerged after surgical repair in comparison to unrepaired nerve. In the proximal nerve portion w/o surgical repair, a marked increase in mRNA expression of MT-3 was observed at both day 6 and day 12 with respect to controls. By contrast, MT-3 mRNA expression showed a marked decrease when the proximal region was sutured with the distal portion. Expression of the two non-neuronal MT isoforms decreased at all times in all conditions in the proximal nerve portion. In the absence of microsurgical repair, at day 6, expression of MT-1 and MT-2 markedly decreased with respect to control as well as at day 12. After microsurgical repair at day 6, the expression of MT-1 and MT-2 markedly decreased with respect to control, but at day 12 the expression rose towards values near to control.

In the distal nerve portion, expression of MT-3 showed changes specular to those observed in the proximal portion. In the absence of suture, the MT-3 mRNA level showed a dramatic decrease at day 6 that was only slightly reversed at day 12 with respect to control. By contrast, when the proximal region was sutured with the distal portion, the MT-3 expression increased with respect to control. In the distal nerve portion in the absence of suture, MT-1 mRNA expression was down-regulated both at day 6 and at day 12 with respect to control. Conversely, MT-2 expression did not change significantly at day 6 and slightly increased at day 12. After microsurgical repair, expression of MT-1 and MT-2 markedly decreased at day 6, but at day 12 the decrease in mRNA expression was reduced.

The transcript levels of the main antioxidant enzymes involved in oxyradical detoxification, catalase-CAT and Cu,Zn-Superoxide dismutase-SOD, as well as glutathione transferase-GST, which is involved in detoxification of various substrates through consumption of the main soluble cellular thiol glutathione (GSH), were also evaluated.

No significant changes in the mRNA expression of the three antioxidant enzymes were observed in the proximal nerve portion w/o surgical repair at day 6, while at day 12 a significant decrease could be appreciated only for CAT expression. No significant changes in GST expression could be appreciated at all times. When the proximal region was sutured with the distal portion the mRNA expression of all the three enzymes was down-regulated. At day 6 expression of GST, CAT and SOD markedly decreased with respect to control, but at day 12, GST and SOD expression slightly rose towards the values of controls, while CAT expression was still very low.

In the distal nerve portion w/o surgical repair at day 6, expression of GST and SOD increased with respect to control, while CAT expression was down-regulated. At day 12 mRNA expression of GST and SOD decreased. GST and CAT expression returned to values of control, while SOD expression was reduced with respect to control. When the proximal region was sutured with the distal, expression of GST and SOD was markedly up-regulated at day 6 with respect to control while CAT expression was still rather low.

PARP-1 is a nuclear enzyme that contributes to both neuronal death and survival under stress conditions. In the proximal nerve portion w/o surgical repair at day 6 PARP-1 expression was up-regulated with respect to control and further increased at day 12. On the other hand, an opposite trend was observed when the proximal region was sutured with the distal portion. At day 6, PARP-1 mRNA expression was markedly down-regulated and further decreased at day 12.

In the distal nerve portion w/o surgical repair mRNA expression of PARP-1 decreased at day 12 with respect to control, while a marked increase occurred at day 6 after suture with respect to control and slightly decreased at day 12.

Peripheral nerve injury produces functional changes in lesioned nerves in which oxidative stress is considered to be the main cause of neuronal damage. Results of these experiments show a modulation of gene expression in both distal and proximal portions of the nerve at 6 and 12 days after dissection in the presence or absence of sutures. Advancing knowledge about the nature and function of these signals may find applications in the development of specific therapeutic approaches for the repair of lesions of peripheral nerves.

References

Chung R.S. and West A.K. (2004) A role for extracellular metallothioneins in CNS injury and repair. Neuroscience 123, 595-599.

De la Hoz C, Oliveira A, De S. Queiroz L and Langone F. Wallerian degeneration in C57BL/6J and A/J mice: differences in time course of neurofilament and myelin breakdown, macrophage recruitment and iNOS expression. J. Anat. 2003, 203:567–578

Hensley K, Robinson KA, Gabbita SP et al. Reactive oxygen species, cell signaling, and cell injury. Free Radic Biol Med 2000, 28:1456–1462.

Vergani L. "Metallothioneins in aquatic organisms: fish, crustaceans, molluscs and echinoderms" in Metallothioneins and Related Chelators (eds A. Sigel, H. Sigel, and R.K.O. Sigel) Royal Society of Chemistry, Cambridge, UK 2009

Zochodne DW, Levy D. Nitric oxide in damage, disease and repair of the peripheral nervous system. Cell Mol Biol 2005, 51:255–267.

Comparative proteomic profile of rat sciatic nerve and gastrocnemius muscle tissues in ageing by 2-D DIGE

D. Capitanio,[1-2] M. Vasso,[1,2] C. Fania,[1,2] M. Moriggi,[1,2] A. Viganò,[1,2] P. Procacci,[3] V. Magnaghi,[4] C. Gelfi[1,2]

[1]Department of Sciences and Biomedical Technologies, University of Milan, [2]Institute of Bioimaging and Molecular Physiology, National Research Council, [3]Department of Human Morphology and Biomedical Sciences, University of Milan, [4]Department of Endocrinology, Physiopathology and Applied Biology, University of Milan, Italy

Background

Ageing induces a progressive functional decline affecting the entire organism, therefore, the loss of muscle mass and function (sarcopenia) contributes significantly to a loss of functional autonomy, increased prevalence of falls, and greater morbidity. In muscles, the age-related degenerative changes produce alteration of morphology associated to muscle fiber atrophy [1], loss of satellite cells [2], and remodelling of neuronal structures [3]. The mechanism of muscle wasting is poorly understood but it could be related to a protein synthesis/degradation imbalance, to the post mitotic nature of myofibers or to a dependency of myofibers on motoneurons innervation [4]. Morphological changes induced by ageing indicate a decrement of muscle fiber size, a change in fiber type distribution and appearance of mitochondrial aggregates with an increment of lipofuscin. The functional and morphological changes in muscles are observed in a parallel manner in nerves. Actually, the ageing process in peripheral nerves induces important biochemical, morphological and functional variations both in myelin and in axons.

Methods

In the present study we characterized sciatic nerves and gastrocnemius muscles by light and electron microscopy. Samples were oriented for cross sectioning of the nerves and for longitudinal or cross sectioning of the muscles. Semi-thin section from each sample were stained with 0.5% toluidine blue in 1% sodium borate and examined by light microscopy (Zeiss Axiophot Photomicroscope). Ultrathin sections were examined under the electron microscope (Zeiss EM 10). The proteomic profile of gastrocnemius muscles and sciatic nerves from six 8- and six 22-month-old rats was examined by 2-D DIGE and mass spectrometry. 2-D DIGE was conducted on protein extracts from each animal, labelled with Cy5 dye, while the internal standards, generated by pooling together an aliquot of each nerve and muscle extracts respectively, were labelled with Cy3 dye. The minimal labelling was performed according to manufacturer's recommendations. CyDye labelled gels were visualized using a Typhoon 9200 laser scanner (GE Healthcare). Spot detection was performed using DeCyder DIA module V. 6.5 (difference in-gel analysis, GE Healthcare). For protein identification, semipreparative gels containing 400 µg of total protein extract per strip, were loaded with unlabelled sample;

electrophoretic conditions were the same as 2-D DIGE, except that gels were stained with a protein fluorescent stain. Tandem electrospray mass spectra were recorded using a HCT Ultra mass spectrometer (Bruker Daltonics) interfaced to a MDLC capillary chromatograph (GE Healthcare). Proteins were identified by correlation of uninterpreted tandem mass spectra to Rattus entries in NCBInr database, using MASCOT software. No mass and pI constraints were applied. One missed cleavage per peptide was allowed, and the fragment ion tolerance window was set to 0.3 Da.

Results

In young rats the muscle fibers present physiological morphology with regular spaces between fibers, while in old rats a number of abnormalities indicative of a degenerative, paraphysiological process were observed. The old muscular fibers showed signs of atrophy, with myofibrillar loss and degeneration, internal migration of nuclei and longitudinal fiber splitting, as previously observed in old and senescent muscles [5]. The electron microscopy analysis clearly supported an increase in subsarcolemmal mitochondrial aggregates and an increased number of lipofuscin granules. Moreover, the myofibrillar degeneration seemed to be mainly related to the disappearance of sarcomeres and was accompanied by the dilatation of sarcoplasmic reticulum. According to alterations described in peripheral nerves of old rats [6], the sciatic nerve showed typical age-associated abnormalities of myelin, like enfoldings, invaginations and presence of onion bulbs. In addition, the electron microscopy revealed some interesting details: the onion bulbs, a typical sign of nerve degeneration, were present at different stages. Some myelinated fibers appeared completely degenerated with myelin breakdown, side axonal atrophy and enfolded loops of myelin. Differential proteomics of aged gastrocnemius was achieved by analyzing 3130 spots per gel. Among them, 319 were differentially expressed (t-test, $p < 0.01$) in old vs. young rat muscles indicating a large (10%) protein reassessment induced by the ageing process, according to a previous study [7]. Relevant changes were found in proteins involved in metabolism, contractile machinery and cytoskeletal organization, signal transduction, stress control and transport. Differential proteomics of aged sciatic nerve detected 2566 spots, 101 of them were differentially changed (t-test, $p < 0.01$) in young vs. old male animals, suggesting that rearrangement induced by ageing in sciatic nerve involves 4% of the total detected spots. Deregulated functional categories included anaerobic metabolism, transport, cell structure and calcium homeostasis.

Conclusions

In this study we confirmed that ageing induces several morphological changes both in the skeletal muscle and in the peripheral nerves, which might affect the physiology of these tissues. These changes are accompanied by deregulation of specific proteins directly related to morphological and functional adaptation; some of them are specific to muscle function, while others are typical of the nerve ageing process. Moreover, with this study, we can conclude that there are many proteins that could be monitored as indicators of nerve and muscle degeneration. Further investigation and validation in human samples will be required to demonstrate that muscle tissue can be a target for the diagnosis and follow up of both neurodegenerative and regenerative processes.

References

[1] Jubrias, S.A., Odderson, I.R., Esselman, P.C., Conley, K.E., 1997. Pflugers Arch. 434, 246-253.
[2] Renault, V., Thornell, L.E., Eriksson, P.O., Butler-Browne, G., Mouly, V., 2002. Aging Cell. 1, 132-139.
[3] Kamel, H.K., 2003. Nutr Rev. 61, 157-167.
[4] Carlson, B.M., 2004. Basic Appl. Myol. 14, 135-139.
[5] Alnaqeeb, M.A., Goldspink, G., 1987. J. Anat. 153, 31-45.
[6] Verdu, E., Ceballos, D., Vilches, J.J., Navarro, X., 2000. J. Peripher. Nerv. Syst. 5, 191-208.
[7] Piec, I., Listrat, A., Alliot, J., Chambon, C., et al., 2005. Faseb J. 19, 1143-1145.

Inhibition of RhoA promotes peripheral axon regeneration

M. Auer,[1] S. Frauscher,[1] E. Udina,[2] X. Navarro,[2] L. Klimaschewski[1]

[1]*Division of Neuroanatomy, Medical University Innsbruck, and* [2]*Institut de Neurosciencies, Universitat Autònoma de Barcelona, Spain*

The small GTPase RhoA is an inhibitor of axonal regeneration. Activation of Rho and its effectors mediates growth cone collapse and retraction via phosphorylation of myosin light chain and interference with actin turnover. In contrast, inhibition of RhoA or expression of a dominant negative construct result in process outgrowth by rat pheochromocytoma cells and cultured hippocampal neurons. Increased RhoA activation was observed in sensory neurons following peripheral nerve lesion. The aim of this study was to assess possible effects of Rho inhibition and Rho down-regulation on peripheral axon regeneration in primary sensory neuron cultures and in the sciatic nerve lesion model.

Dissociated dorsal root ganglia (DRG) neurons obtained from adult rats were transfected with RhoA siRNA plasmids also encoding EGFP. Pharmacologically, Rho activation was inhibited by Clostridium botulinum toxin C3 [10 ng/ml], which inactivates Rho by ADP-ribosylation. Axon

growth of cultured neurons was determined by measurement of the total axonal length. For the in vivo experiments the right sciatic nerve of adult rats was transected and a 12 mm-silicon tubule bridging the nerve stumps was implanted. It was filled with a collagen solution containing C3 [0.5 mg/ml or 0.25 mg/ml]. Controls received collagen or an enzymatically inactive mutant version of C3. Functional sciatic nerve regeneration was assessed monthly using the walking track (testing locomotor function), thermal algesimetry (assessing pain sensation), and electrophysiological recording of digital sensory nerve action potentials (assessing regenerated nerve conduction).

RhoA siRNA transfected or C3 treated DRG neurons exhibited a significant increase in total axonal length within a 24h culture period. After C3 application in vivo, locomotor function improved in the group that received C3 [0.5 mg/ml] at 60 days post operation (dpo). Animals treated with either C3 concentration regained normal sensation at 90 dpo. Sciatic nerve action potentials at the digital level could be detected first in the C3 [0.5 mg/ml] treated group.

Taken together, our results indicate a growth promoting effect of Rho down-regulation or Rho inactivation in adult primary neuron culture and a functional improvement in response to Rho inactivation following peripheral nerve injury.

JNK-AP1 pathway is involved in Axonal Growth Potential and neuroregulation of CGRP dynamics in mice DRG after peripheral nerve injury

G. Manassero,[1] I. Repetto,[1] F. Rossi,[2] A. Vercelli[1]

[1]Department of Anatomy, Pharmacology and Forensic Medicine, University of Turin, Turin, Italy, and [2]National Institute of Neuroscience, University of Turin, Turin, Italy. Emails: giusi.manassero@unito.it, ivan.china86@gmail.com, ferdinando.rossi@unito.it, alessandro.vercelli@unito.it

Background

Proinflammatory cytokine interleukin-1 (IL-1)-β modulates the events caused by the nerve damage. IL-1β can activate C-Jun N-terminal kinase (JNK) that phosphorylate c-Jun to form an Activator Protein 1 (AP1) complex (Hou et al., 2003). The JNK-AP1 pathway remains active for long periods after axotomy, but its activity diminishes during target contact, suggesting an important role in regeneration (Kenney and Kocsis, 1998). In DRG neurons, JNK can regulate the synthesis of the neuropeptide Calcitonin Gene-Related Peptide (CGRP) after stimulation of IL-1β. Furthermore, CGRP, whose expression dynamic in primary sensory neurons of L4-L5 DRGs is influenced by sciatic nerve transaction (SNT) (Zheng et al., 2008), has been recognized as a nerve regeneration-promoting peptide in vivo.

This study aims i) to examine the JNK-related effect of neuronal injury on the axonal growth potential of nociceptive DRG neurons and its relationship with CGRP neuroregulation; ii) to clarify the role of the different JNK isoforms in these mechanisms.

Methods

Adult male 2-4 months-old ko mice for the different JNK isoforms and age-matched wild types (wt) underwent sciatic nerve transaction (SNT) (N wt=11, N ko1=6, N ko2=6, N ko3=6) and were perfused after 72h. Ipsi- and contralateral L4 DRGs were analysed by CGRP and GAP43 IHC. In a separate group of wt animals, to investigate whether JNK was involved in SNT-induced CGRP and GAP43 expression, DJNKI1 (0.3 mg/kg) was i.p. injected 30 min before surgery. Quantitative analysis of the CGRP and GAP43 immunoreactivities (IR) was assessed with the Neurolucida software. Our controls were naive mice. Before lesion we excluded differences between wt and ko animals in i) the footprint patterns ii) CGRP and GAP43 basal expression.

Results

Treatment reversed CGRP downregulation ($P<0.05$), which was elevated after transection in wt mice ($P<0.001$) (Fig.1). 72h post-lesion, we found a marked decrease in the percentage of CGRP-IR neurons both in wt and JNK3 ko mice ($P<0.001$, vs control) whereas its downregulation was absent both in JNK1 and JNK2 ko. Furthermore, SNT induced GAP-43 upregulation, related to axonal growth potential ($P<0.01$), which was significantly prevented by DJNKI1 treatment ($P<0.05$) (Fig.2). The number of GAP43-IR neurons on the ipsilateral side was unchanged both in JNK1 and JNK3 ko vs control, whereas in JNK2 ko mice was similar to the wt. SNT affected also contralateral CGRP-IR neurons that showed similar changes to the axotomized side.

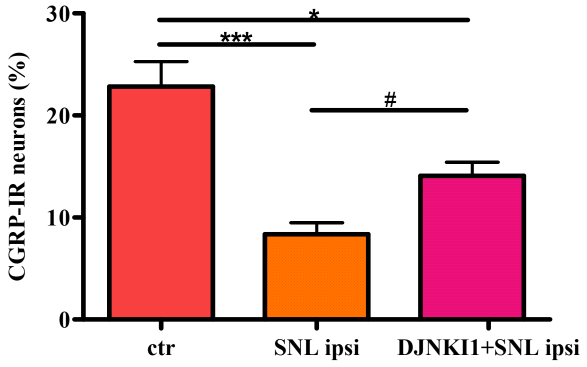

Fig. 1 Effect of i.p DJNKI1 on SNL-induced CGRP expression in L4 DRG neurons. *p<0.05,***p<0.001 by tTest compared with

control; n=6. #p<0.05 by tTest compared with SNL ipsi; n=6. Value are mean ± SEM.

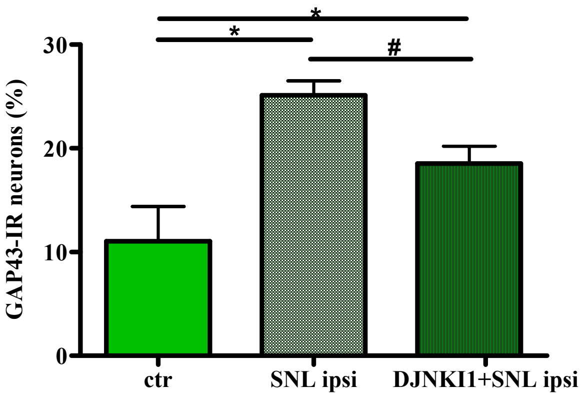

Fig. 2 Effect of i.p DJNKI1 on SNL-induced GAP43 expression in L4 DRG neurons. *p<0.05 by tTest compared with control; n=6. #p<0.05 by tTest compared with SNL ipsi; n=6. Value are mean + SEM.

Conclusions

These findings suggest that JNK-AP-1 pathway is involved in SNT neuroregulation of both GAP43 and CGRP in DRG neurons and that different isoforms are responsible for this interaction. Sensory axon regeneration process may require different JNKs depending on promotion or sprouting: the JNKs pair 1 and 2 are responsible for the interaction with CGRP, whereas knockout of two others JNKs together 1 and 3 may impair axonal growth potential. Our data also support the idea that contralateral side cannot be considered as a baseline, although it has been widely used as reference. Contralateral DRGs seem to be affected by the reactive program of axotomized side: they might sense the lesion by diffusive agents produced in the ipsilateral spinal cord.

References

Hou, L., Li, W., Wang, X., 2003. Mechanism of interleukin-1 beta-induced calcitonin gene-related peptide production from dorsal root ganglion neurons of neonatal rats. J. Neurosci. Res. 73, 188-197.

Kenney, A.M., Kocsis J.D., 1998. Peripheral axotomy induces long-term c-Jun amino-terminal kinase-1 activation and activator protein-1 binding activity by c-Jun and junD in adult rat dorsal root ganglia in vivo. J. Neurosci. 18, 1318- 1328.

Zheng, L.F., Wang, R., Xu, Y.Z., Yi, X.N., Zhang, W.J., Zeng, Z.C., 2008. Calcitonin gene-related peptide dynamics in rat dorsal root ganglia and spinal cord following different sciatic nerve injuries. Brain Res. 1187, 20-32.

FGF signaling in adult sensory neurons promotes long-distance regeneration after axotomy

L. Klimaschewski, B. Hausott

Department of Anatomy and Histology, Division of Neuroanatomy (www.neuroanatomy.at), Muellerstrasse 59, A-6020 Innsbruck,
Email: lars.klimaschewski@i-med.ac.at

Peripheral nerve lesions are common. They cause motor and sensory deficits with often serious clinical consequences such as prolonged paralysis, anaesthesia and neuropathic pain. Therefore, improvement of long-distance axonal regeneration is important for fast elongation of axons into target muscles which atrophy in the absence of reinnervation.

Primary neurons derived from dorsal root ganglia are particularly suitable to study regeneration-associated neuronal plasticity. Their axons are elongating and branching after lesion mainly because of the permissive environment provided by Schwann cells, extracellular matrix and neurotrophic activities in vivo. Various families of neurotrophic factors exist, for example, the neurotrophins and members of the fibroblast growth factor (FGF) family. Some of the different FGF proteins and their receptors play a prominent role in axon growth during brain development and axon regeneration in the adult nervous system.

FGF-2 (basic fibroblast growth factor) is up-regulated in response to nerve injury and has been shown to promote neuronal survival and neurite outgrowth. FGFs mediate their response by activation of four types of high affinity tyrosine kinase receptors (FGFR1-4). Novel negative feedback regulators of FGFR signalling have been described, but their significance for axonal growth has not been investigated so far.

My laboratory focuses on the signalling pathways activated by FGFR1 to influence different modes of regeneration, such as axon elongation, branching and maintenance. FGFR1 overexpression and inhibition of receptor degradation strongly stimulate the neuronal ERK pathway and promote elongative axon growth by adult sensory neurons. Degradation of FGFR1 was inhibited by the lysosomal inhibitor leupeptin and by the proteasomal inhibitor lactacystin. FGFR1 overexpression enhanced FGF-2-induced axon growth by adult sensory neurons, which was further increased by co-treatment with leupeptin.

Therefore, lysosomal inhibition of receptor degradation concomitant with ligand stimulation of neurons overexpressing FGFR1 represents a new mechanism of tyrosine kinase receptor mediated promotion of axon regeneration and demonstrates that adult sensory neurons express sub-optimal levels of tyrosine kinase receptors for neurotrophic factors.

Furthermore, Sprouty proteins act as negative feedback inhibitors of the ERK pathway. We analyzed the expression of Sprouty1, -2 and -4 in adult sensory neurons as well as the effects of Sprouty inhibition on axon growth by small interfering RNAs (siRNAs). In DRG cultures, Sprouty2 revealed the highest expression level. Down-regulation of Sprouty2 promoted elongative axon growth by adult sensory neurons. In response to Sprouty2 knockdown, enhanced FGF-2-induced activation of ERK and Ras could be observed, but phosphorylation of Akt and p38 remained unaffected. Our results imply that Sprouty2 is highly expressed in adult peripheral neurons and its down-regulation strongly promotes elongative axon growth by activation of the Ras/Raf/ERK pathway suggesting novel novel therapeutic strategies to promote rapid and specific peripheral axon elongation in vivo (supported by FWF, MUI, COST).

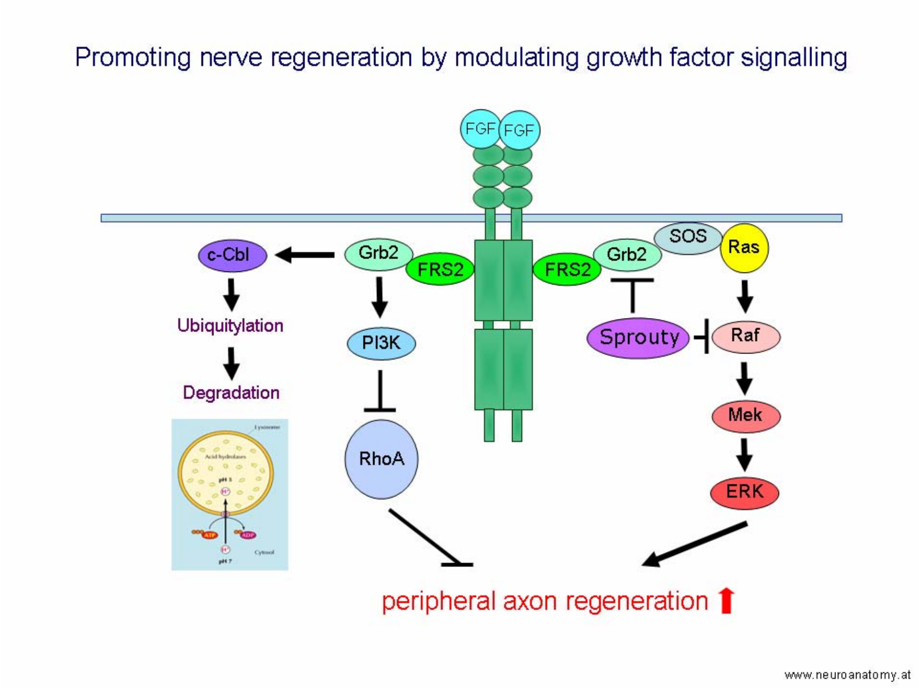

References

1. Klimaschewski L, Nindl W, Feurle J, Kavakebi P, Kostron H. (2004) Basic fibroblast growth factor isoforms promote axonal elongation and branching of adult sensory neurons in vitro. NEUROSCIENCE, 126, 347-353
2. Klimaschewski L, Hausott B, Ingorokva S, Pfaller K. (2006) Constitutively expressed catalytic proteasomal subunits are up-regulated during neuronal differentiation and required for axon initiation, elongation and maintenance. JOURNAL OF NEUROCHEMISTRY, 96, 1708-1717
3. Kavakebi P, Hausott B, Tomasino A, Ingorokva S, Klimaschewski L. (2005) The N-end rule ubiquitin conjugating enzyme, HR6B, is up-regulated by nerve growth factor and required for neurite outgrowth. MOLECULAR AND CELLULAR NEUROSCIENCE, 29, 559-568
4. Hausott B, Schlick B, Vallant N, Dorn R, Klimaschewski L. (2008) Promotion of neurite outgrowth by FGFR1 overexpression and lysosomal inhibition of receptor degradation in PC12 cells and adult sensory neurons. NEUROSCIENCE, 153, 461-473
5. Hausott B, Vallant N, Auer M, Yang L, Dai F, Brand-Saberi B, Klimaschewski L. (2009) Sprouty2 down-regulation promotes peripheral axon regeneration by adult sensory neurons. MOLECULAR AND CELLULAR NEUROSCIENCE, in press.

Dorsal root ganglia neuronal population increases over 40% as a consequence of a crush lesion of brachial pleus nerves in adult rats

M. Fornaro, G. Ronchi, S. Raimondo, L. Muratori, I. Perroteau, S. Geuna, MG. Giacobini-Robecchi
Department of Clinical and Biological. Sciences, University of Turin, Orbassano (To), Italy

Many experimental works have suggested that new-generated neurons could be added to the DRG (dorsal root ganglia) postnatally. Some authors have pointed out the possible existence of neurogenesis in adult DRGs, others

have suggested that these new neurons could instead originate from the maturation or growth of preexisting immature cells (Ciaroni et al., 2000; Lagares et al., 2007). However adult DRG neurogenesis has not been confirmed in vivo.

In the present study, we investigated, using different experimental approaches, the effect of a nerve crush lesion on DRGs sensory neurons in adult rat. The crush injury was applied to the median, ulnar and radial nerves at their point of origin from the brachial plexus using a non-serrated clamp that could guarantee standardized and reproducible method. Animals were then sacrificed at 1, 5, 10 and 30 days after the injury and DRG corresponding only to the level C5-T1, that give rise to the fibers of the radial, ulnar and median nerves, were extracted.

Our morphological analysis in optical and electron microscopy reveals an unusual number of small size cells morphologically different from the glial satellite cells. FACS analysis performed at 1, 5, 30 days post-damage confirmed that there is no significant loss of sensory neurons.

Neurogenesis was then investigated by injecting rats with bromodeoxyuridine (BrDU). Immunohistochemical analysis in laser confocal revealed a relevant number of cells BrDU-positive. Most of the BrDU positive cells belong to the glial family although, some BrDU colocalized with neuronal markers suggesting that neurogenesis occurs in adult DRGs neurons that undergo peripheral nerve injury. These data are supported by evidence of neuronal progenitor's markers as nestin, Sox-2 expressed within the crushed DRGs 1 and 5 days after damage.

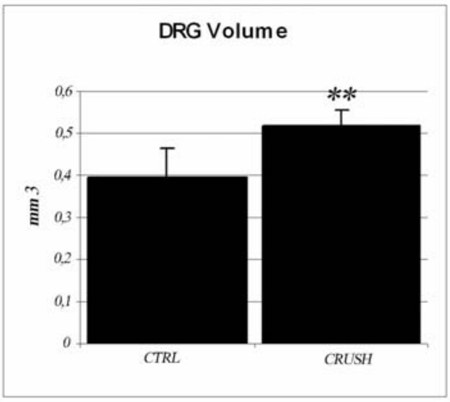

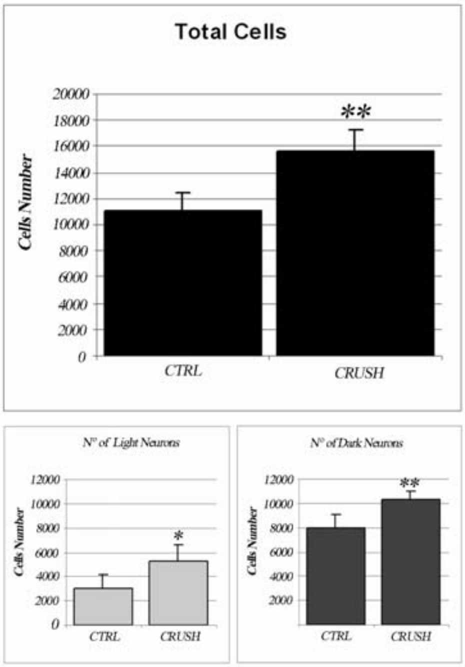

Finally, a stereological analysis, using the physical dissector method, showed a significant increase in number (42%) of DRGs sensory neurons 1 month after nerve-crush injury compared to control. All together our data support the idea that the population of DRG's neurons increased as a consequence of the nerve damage. Evidence of morphological changes in the population of cells surrounding neurons and the immunopositivity for neuronal progenitor markers, suggested the hypothesis that the increased number of neurons is due to undifferentiated precursors localized within the adult DRG.

References

Ciaroni S, Cecchini T, Cuppini R, Ferri P, Ambrogini P, Bruno C, Del Grande P (2000) Are there proliferating neuronal precursors in adult rat dorsal root ganglia? Neurosci Lett 281:69-71.

Lagares A, Li HY, Zhou XF, Avendano C (2007) Primary sensory neuron addition in the adult rat trigeminal ganglion: evidence for neural crest glio-neuronal precursor maturation. J Neurosci 27:7939-7953.

Axonal regeneration: Interplay between GAP-43 and receptors of myelin inhibitory molecules

S. Foscarin,[1] S. Gianola,[1] D. Carulli,[1] P. Fazzari,[2] S. Mi,[3] L. Tamagnone,[2] F. Rossi[1]

[1]Department of Neuroscience and ''Rita Levi Montalcini Centre for Brain Repair'', Neuroscience Institute of Turin (NIT), University of Turin, I-10125 Turin, Italy; [2]Division of Molecular Oncology, Institute for Cancer Research and Treatment (IRCC), University of Turin Medical School, Candiolo, Turin, Italy; [3]Biogen Idec, 14 Cambridge Center, Cambridge, MA, USA

Overexpression of GAP-43 modifies the distribution of NgR and PlexinB1 in injured Purkinje axons. Neurons with enhanced intrinsic growth capabilities can elongate their axons into non-permissive territories, but the mechanisms that enable the outgrowing processes to overcome environmental inhibition are largely unknown. To address this issue, we examined adult mouse Purkinje cells that overexpress the axonal growth-associated protein GAP-43.

After injury, these neurons exhibit sprouting along the intracortical neuritic course and at the severed stump in the white matter. To determine whether GAP-43-overexpressing Purkinje cells are responsive to extrinsic inhibitory cues, we investigated the content and subcellular localization of major receptors for myelin-associated inhibitory proteins, PlexinB1 and the Nogo receptor (NgR) with the related co-receptors LINGO-1 and p75. Expression of these molecules, estimated by measuring perikaryal immunostaining intensity and Western blot, was not different in wild-type or transgenic mice, and it was not overtly modified after axotomy. Following injury, however, the content of PlexinB1 was significantly reduced in GAP-43-overexpressing neurites. Furthermore, in the same axons the distribution of both PlexinB1 and NgR was altered, being inverse to that of GAP-43. Labelling for the two receptors was conspicuously reduced on the axonal surface and it was almost undetectable in the outgrowing sprouts, which showed strong GAP-43 immunoreactivity. These observations indicate that although GAP-43 overexpression does not modify the expression of receptors for myelin-associated inhibitory factors, it interferes with their subcellular localization and exposure on the neuritic membrane. Therefore, GAP-43 promotes axon growth by multiple synergistic mechanisms that potentiate the intrinsic motility of the elongating processes, while reducing their sensitivity to environmental inhibition.

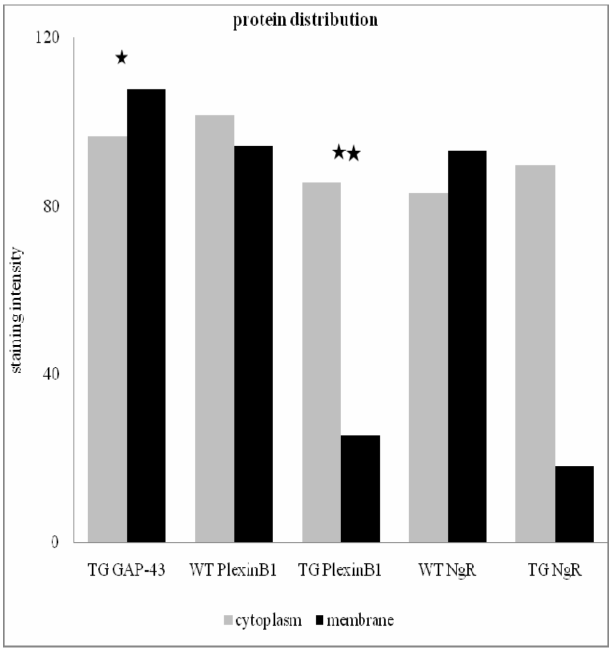

Analysis of the subcellular distribution of GAP-43, PlexinB1 and NgR immunostaining in Purkinje axon torpedoes or terminal bulbs (1 month after axotomy). In GAP-43-overexpressing mice, GAP-43 staining intensity is higher on the cell membrane, in contrast with PlexinB1 and NgR, which present the highest staining intensity in the cytoplasmic compartment. On the contrary, in wild-type animals, both PlexinB1 and NgR present the same staining intensity in the two cellular compartments. (n = 51 for GAP-43; n = 24 for PlexinB1; n = 27 for NgR; mean ± SEM. Paired t-test, *P < 0.05, **P < 0.001).

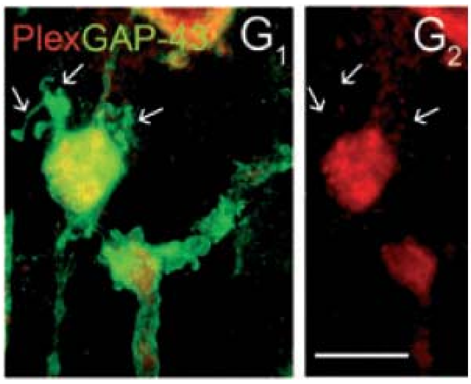

Morphology of injured Purkinje axons (1 month after axotomy) and pattern of PlexinB1 immunoreactivity (red) in intracortical neurites (TG). Arrows point to sprouts budding from TG axons; note that these sprouts show strong GAP-43 staining, whereas PlexinB1 is undetectable

References

Buffo, A., Holtmaat, A.J.D.G., Savio, T., Verbeek, J.S., Oberdick, J., Oestreicher, A.B., Gispen, W.H., Verhaagen, J., Rossi, F. & Strata, P. (1997) Targeted overexpression of the neurite Growth-Associated Protein B-50 / GAP-43 in cerebellar Purkinje cells induces sprouting after growth-permissive transplants. J. Neurosci., 17, 8778–8791.

Fazzari, P., Penachioni, J., Gianola, S., Rossi, F., Eickholt, B.J., Maina, F., Alexopoulou, L., Sottile, A., Comoglio, P.M., Flavell, R.A. & Tamagnone, L. (2007) Plexin-B1 plays a redundant role during mouse development and in tumor angiogenesis. BMC Dev. Biol., 7, 55.

Fournier, A.E., Grand-Pre`, T. & Strittmatter, S.M. (2001) Identification of a receptor mediating Nogo-66 inhibition of axonal regeneration. Nature, 409, 341–346.

Gianola, S. & Rossi, F. (2004) GAP-43 overexpression in adult mouse Purkinje cells overrides myelin-derived inhibition of neurite growth. Eur. J. Neurosci., 19, 819–830.

Rossi, F., Jankovsky, A. & Sotelo, C. (1995) Differential regenerative response of Purkinje cell and inferior olivary axons confronted with embryonic grafts: environmental cues versus intrinsic neuronal determinants. J. Comp. Neurol., 359, 663–677.

Worzfeld, T., Puschel, A.W., Offermanns, S. & Kuner, R. (2004) Plexin-B family members demonstrate non-redundant expression patterns in the developing mouse nervous system: an anatomical basis for morphogenetic effects of Sema4D during development. Eur. J. Neurosci., 19, 2622–2632.

Glutamate: A possible role in peripheral nerve damage?

G. Cavaletti

Dipartimento di Neuroscienze e Tecnologie Biomediche, Università di Milano-Bicocca, Monza, Italy

Glutamate is the major mediator of excitatory signalling in the mammalian central nervous system, but it has recently been shown to play a role in the transduction of sensory input at the periphery and in peripheral neuropathies as well. New advances in research have demonstrated that rat peripheral sensory terminals and Dorsal Root Ganglia (DRG) express molecules involved in glutamate signalling, including “high-affinity” membrane bound glutamate transporters (EAATs), and that alterations in their expression and/or functionality can be implicated in several models of peripheral neuropathy, neuropathic pain and hyperalgesia.

In order to get a deeper knowledge into the possible role of glutamate in peripheral nerve damage we have investigated, through immunoblotting, immunofluorescence assays and β-counter analysis of [H3] L-glutamate uptake, the expression, the distribution and the activity of the EAATs in in vitro cultures of neuronal and neuronal+satellite cells of embryonic DRG.

Our results demonstrated that EAATs are expressed in both cell cultures with a peculiar pattern of distribution. The EAATs immunolabelling was observed in the cytoplasm of neuronal or satellite cells with spots of strong labelling underneath the cellular membranes. Moreover, both cell cultures showed a strong sodium-ATP-dependent glutamate uptake activity and the net glutamate transport was more marked in neurons alone.

These results demonstrating that functionally active EAATs can be studied in DRG cell cultures give further evidence for a role of glutamatergic transport in the peripheral nervous system and will be useful to test if any change occur in in vitro models of peripheral nervous system damages.

Characterization of the Neonatal Olfactory Bulb Ensheathing Cell Line (NOBEC) in the perspective of its employment for experimental nerve tissue engineering

R. Pellitteri,[1] S. Geuna,[2] S. Raimondo,[2] C. Audisio,[3] G. Gambarotta,[3] D. Zaccheo,[4] I. Perroteau[3]

[1]Institute of Neurological Sciences,CNR, Catania, [2]Department of Clinical and Biological Sciences, [3]Department of Animal and Human Biology, University of Turin, [4]Department of Experimental Medicine, University of Genoa, Italy

Olfactory Ensheathing Cells (OECs), an unusual glial population of the olfactory system, have raised great interest over recent years for their exceptional plasticity and for supporting olfactory neurogenesis. Several studies reveal that OECs produce both various growth factors and adhesion molecules, exerting a positive influence on the survival and axonal outgrowth of CNS neurons in vitro (Pellitteri et al., 2007, 2009). Here, we provide an in vitro

characterization of the NOBEC (Neonatal Olfactory Bulb Ensheathing Cells) line that was obtained from primary cells dissociated from rat neonatal olfactory bulb (OB) and immortalized by retroviral transduction of SV40 large T antigen (Goodman et al., 1993). Light and electron microscopy investigation showed that NOBECs are a homogeneous cell population both at structural and ultrastructural level. RT-PCR, Western blotting and immunocytochemistry releaved that NOBECs express the glial markers S100, GFAP (Glial Fibrillar Acid Protein) and p75NGFR as well as NRG1 (neuregulin-1) and ErbB1-2-3 receptors while they are negative for ErbB4 (Audisio et al., 2009). In addition, NOBECs show immunopositivity for other markers, such as calponin, nestin, CD133 and vimentin. In particular, vimentin (its expression is modulated during cellular maturation) showed a higher labelling expression compared to the other markers (Fig.1).

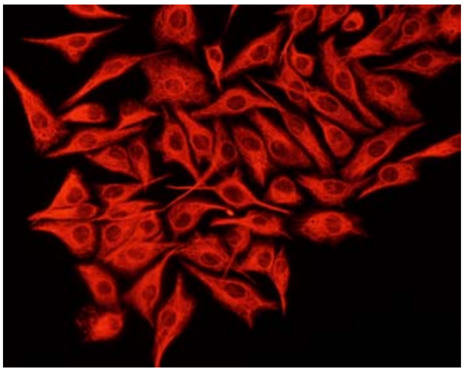

Figure 1

Moreover, in immunocytochemical studies, we examined the expression of some neuronal markers, such as Protein Gene Product (PGP 9.5) and Microtubule Associate Protein-2 (MAP-2) in NOBECs grown both in serum containing medium and in serum-free medium added with different growth factors (NGF or bFGF or GDNF). We observed a strong immunoreaction to PGP 9.5 and MAP-2 increased in NOBECs grown in serum-free medium both treated with growth factors and without them. These results suggest that the highest expression of neuronal markers in serum-free medium might be explained by serum containing molecules that inhibit the effect of growth factors on the PGP 9.5 and MAP-2 expression.

Yet, NOBECs exhibit a high proliferation and migration basal activity and can be transducted with vectors carrying GFP (Green Fluorescent Protein) and NRG1 cDNA. Functional stimulation by means of NRG1-III-β3 overexpression through viral transduction induced a significant increase in cell proliferation rate while it had no effect on cell migration (Audisio et al., 2009).

It could be concluded that NOBEC cell line retains glial features both morphologically and functionally and could thus be used for both in vitro assays of glial cell manipulation and in vivo experimental studies of glial cell transplantation in the nervous system. These properties might render them potential clinical agents and of trophic support to CNS injury.

References

Pellitteri, R., Spatuzza, M., Russo, A., Stanzani, S., 2007. Olfactory ensheathing cells exert a trophic effect on the hypothalamic neurons "in vitro". Neurosci. Lett. 417, 24-29.

Pellitteri, R., Spatuzza, M., Russo, A., Zaccheo, D., Stanzani, S., 2009. Olfactory Ensheathing Cells represent an optimal substrate for hippocampal neurons. An in vitro study. Int. J. Develop. Neurosc. 27, 453-458.

Goodman, M.N., Silver, J., Jacobberger, J.W., 1993. Establishment and neurite outgrowth properties of neonatal and adult rat olfactory bulb glial cell lines. Brain Res. 619, 199–213.

Audisio, C., Raimondo, S., Nicolino, S., Gambarotta, G., Di Scipio, F., Macrì, L., Montarolo, F., Giacobini-Robecchi, M.G., Porporato, P., Filigheddu, N., Graziani, A., Geuna, S., Perroteau, I. 2009. Morphological and biomolecular characterization of the neonatal olfactory bulb ensheathing cell line. J. Neurosci. Methods 185, 89-98.

Isolation method for a stem cell population with neural potential from skin and adipose tissue in the perspective of peripheral nerve reconstruction

V. Vindigni,[1] B. Zavan,[2] L. Lancerotto,[1] F. Bassetto,[1] G. Abatangelo[2]

[1]*Clinic of Plastic and Reconstructive Surgery, University of Padova;* [1]*Dept of Histology, Microbiology and Medical Biothecnology, University of Padova, Italy*

Objective

In recent years, research on stem cells has been focused on the development of personalized cell-based therapies. Owing to their homing properties, adult human stem cells are a promising source of autologous cells to be used as therapeutic vehicles[1-3]. Multiple potential sources for clinically useful stem and progenitor cells have been identified, including autologous and allogenic embryonic, fetal and adult somatic cells from neural, adipose and mesenchymal tissue. In particular neural stem cells are immature self-renewing cells that can generate neurons and glia, which could make them useful in many clinical applications[4]. However, ethical and practical issues constrain the availability of neural stem cells derived from human embryonic tissue[4]. In the present report, we describe a simple protocol to obtain an enriched culture of adult stem cells organized in neurospheres from two post-natal tissues: skin and adipose tissue and we propose our future aim to combine these adult stem cells with a tridimensional scaffold.

Methods

Adult stem cells isolated from skin and adipose tissue derived from the same adult donor were amplified under varying conditions related to the coating of the chamber slide and the presence of serum and/or growth factors, such as with EGF and FGF2. Neurospheres were then expanded and evaluated in terms of proliferation and gene expression.

Results

Adipose and skin derived neurospheres were comparable in size, quantity of cells and genes expressed. Cells from both types of tissue grew optimally without slide coating, in the presence of serum and with the combined addition of FGF2 and EGF. After 7 days, most cells of both tissue origins adhered to the tissue culture plastic assuming a fibroblast-like phenotype: large, flat and spindle shaped cells with a small population of expanded cells organized into spheres growing in suspension. At day 7, neurospheres present in the medium were collected and their number analysed. Total cells forming neurospheres were determined from a standard curve (micrograms DNA versus cell number).The presence of a coating condition is not able to generate neurospheres: only in the absence of any attachment factor the presence of neurospheres is detectable. In this proliferative phase, serum also contributed in an important way to neurosphere production: the highest number of neurospheres was produced in cells cultured in serum supplemented medium in both skin and adipose tissue derived cell cultures. Similarly, the combined presence of EGF and FGF ensured the highest number of neurospheres. Although EGF alone and FGF alone enhanced neurosphere production, results were greatly improved when the combination was added to the medium. With regard to gene expression, tubulin III and nestin were detected in all neurospheres obtained with all medium conditions. Skin and adipose tissue derived neurospheres demonstrated no difference in cell number or gene expression, confirming their homogeneity regardless of their diverse origin.

Discussion

We described a method of obtaining a population of neural crest-related precursor cells organized in structures known as neurospheres from two highly accessible tissue sources: adult mammalian dermis and adipose tissue. This autologous adult stem cell population could be used for cell replacement or cell therapies. The next step will be to combine this cell population with a biocompatible scaffold. For *in vivo* peripheral nerve regeneration, three-dimensional distribution and growth of cells within the porous scaffold are of clinical significance. We have previously tested a hyaluronic acid based biomaterial as a scaffold for different cell nerve components [5]. Then, our future purpose will be to test *in vivo* a tissue engineered nerve conduit made by a hyaluronic acid based tubular conduit (HYAFF-11) enriched with adipose and skin derived neurospheres, following the main pillars of tissue engineering: namely (1) microsurgery, (2) cell and tissue transplantation, (3) material science, and (4) gene transfer[6].

References

1. Chalfoun CT, Wirth GA, Evans GR. Tissue engineered nerve constructs: Where do we stand? J Cell Mol Med 2006; 10: 309–317
2. Fernandes KJL, McKenzie IA, Mill P, et al. A dermal niche for multipotent adult skin-derived precursor cells. Nat Cell Biol 2004;6: 1082–1093
3. Fernandes KJ, Kobayashi NR, Gallagher CJ, et al. Analysis of the neurogenic potential of multipotent skin-derived precursors. Exp Neurol 2006; 201: 32–48
4. Walsh S, Midha R. Practical considerations concerning the use of stem cells for peripheral nerve repair. Neurosurg Focus. 2009 Feb;26(2):E2.
5. Zavan B, Abatangelo G, Mazzoleni F, Bassetto F, Cortivo R, Vindigni V. New 3D hyaluronan-based scaffold for in vitro reconstruction of the rat sciatic nerve. Neurol Res. 2008 Mar;30(2):190-6.
6. Battiston B, Raimondo S, Tos P, Gaidano V, Audisio C, Scevola A, Perroteau I, Geuna S. Tissue engineering of peripheral nerves. Int Rev Neurobiol. 2009;87:227-49.

GABA receptors in adult stem cells after differentiation into Schwann-like cells: A possible target to promote nerve repair?

A. Faroni,[1] V. Magnaghi,[2] G. Terenghi[1]

[1]Blond McIndoe Laboratories, Tissue Injury and Repair Group, The University of Manchester, Manchester, UK; [2]Department of Endocrinology, Physiopathology and Applied Biology, University of Milan, Milan, Italy

Gamma amino-butyric acid (GABA) is the main inhibitory neurotransmitter in the adult central nervous system (CNS). GABA interacts with different receptor types, respectively the ligand-gated ion channel GABA-A and the metabotropic GABA-B receptor. Both GABA-A and GABA-B receptor types are expressed in glial cells of the central and peripheral nervous system (PNS), where they are involved in the biology of myelin (Magnaghi, 2007). Previous in vitro studies showed that GABA receptor ligands modulate the proliferation and expression of myelin proteins in Schwann cells (SC) cultured from neonatal rat sciatic nerves (Magnaghi, et al., 2004, Magnaghi, et al., 2006).

Furthermore, the importance of GABA-B receptors in peripheral myelination has been analysed in vivo in GABA-B1 deficient mice (Magnaghi, et al., 2008). SC play an essential role in peripheral nerve regeneration, but due to their slow growth rate, they are not profitable to a tissue engineering approach for nerve regeneration (Terenghi, et al., 2009). Adult stem cells, derived either from adipose tissue (ADSC) or from bone marrow (BM-MSC), can be differentiated into a SC-like phenotype and thus potentially be used as SC replacement (Caddick, et al., 2006, di Summa, et al., 2009). The aim of this study was to determine the expression pattern of GABA receptors in these cells following differentiation and to assess the similarity with SC. By means of immunocytochemistry, RT-PCR and western blot analysis we found that both BM-MSC and ADSC express several subunits of GABA-A and B receptors. Specifically, GABA-A subunits α2 and β3, as well as GABA B1 and B2 subunits are expressed in both BM-MSC and ADSC. Interestingly, GABA-A α2 mRNA and protein levels appear to be upregulated in SC-like differentiated cells. Furthermore, the localisation of these proteins observed by immunocytochemistry analysis is consistent with previous observations made on SC (Magnaghi, et al., 2004, Magnaghi, et al., 2006). Altogether, our data show that GABA receptors are present in BM-MSC and ADSC stem cells, where they might be functionally active regulating key processes such as SC-like differentiation and proliferation. Further studies, using in vitro and in vivo models of PNS regeneration, are direct to analyze whether GABAergic drugs and SC-like stem cells may be a valid tool to improve peripheral nerve repair after injury.

References

Caddick, J., Kingham, P. J., Gardiner, N. J., Wiberg, M., and Terenghi, G., 2006. Phenotypic and functional characteristics of mesenchymal stem cells differentiated along a Schwann cell lineage. Glia 54, 840-849.

di Summa, P. G., Kingham, P. J., Raffoul, W., Wiberg, M., Terenghi, G., and Kalbermatten, D. F., 2009. Adipose-derived stem cells enhance peripheral nerve regeneration. J Plast Reconstr Aesthet Surg.

Magnaghi, V., 2007. GABA and Neuroactive Steroid Interactions in Glia: New Roles for Old Players? Curr Neuropharmacol 5, 47-64.

Magnaghi, V., Ballabio, M., Camozzi, F., Colleoni, M., Consoli, A., Gassmann, M., Lauria, G., Motta, M., Procacci, P., Trovato, A. E., and Bettler, B., 2008. Altered peripheral myelination in mice lacking GABAB receptors. Mol Cell Neurosci 37, 599-609.

Magnaghi, V., Ballabio, M., Cavarretta, I. T., Froestl, W., Lambert, J. J., Zucchi, I., and Melcangi, R. C., 2004. GABAB receptors in Schwann cells influence proliferation and myelin protein expression. Eur J Neurosci 19, 2641-2649.

Magnaghi, V., Ballabio, M., Consoli, A., Lambert, J. J., Roglio, I., and Melcangi, R. C., 2006. GABA receptor-mediated effects in the peripheral nervous system: A cross-interaction with neuroactive steroids. J Mol Neurosci 28, 89-102.

Terenghi, G., Wiberg, M., and Kingham, P. J., 2009. Chapter 21: Use of stem cells for improving nerve regeneration. Int Rev Neurobiol 87, 393-403.

Schwann-cell transplantation and electrical stimulation to increase peripheral nerve regeneration across long gaps

K. Haastert,[1,2] D. Klode,[1] R. Schmitte,[1] N. Korte,[1] C. Grothe[2]

[1]Hannover Medical School, Institute of Neuroanatomy, Hannover; [2]Center for Systems Neurosciences (ZSN) Hannover, Hannover, Germany

Objective

During the last years we investigated several ways to increase peripheral nerve regeneration across long gaps. We demonstrated before that regeneration promoting growth factors like fibroblast growth factor-2 can be increased at the site of nerve reconstruction by ex vivo gene therapy (3, 6). Electrical stimulation of the proximal peripheral nerve stump prior to end-to-end coaptation or tubular bridging of small nerve gaps has been reported to increase preferential motor reinnervation and functional motor recovery (1). Here we investigated the effects of electrical stimulation on regeneration across 13 mm peripheral nerve gaps in rats. Three paradigms were evaluated, (I) reconstruction using autotransplants, (II) transplantation of differentially filled silicone tubes and (III) evaluation of ES combined to gene therapy with fibroblast growth factor-2 (FGF-221/23kD).

Materials and Methods

Adult female Sprague Dawley rats were separarted into control condition without and test condition with electrical stimulation for 1h (20Hz, 0.3mA) prior to nerve gap reconstruction.

Autotransplantation **(I)** was done by transecting the sciatic nerve at mid-thigh once proximal and 13 mm distal again and resuturing both transection sites. Differentially filled silicone tubes **(II)** contained: (A) matrigel alone or (B) neonatal, naïve rat Schwann cells. To evaluate paradigm **(III)**, silicone tubes were filled with (C) naïve Schwann cells, (D) empty vector transfected Schwann cells, (E) Schwann cells over-expressing FGF-$2^{21/23kD}$. For histomorphometrical analysis, regenerated nerve tissue was stained for myelin and epon-embedded.

Semithin cross sections at defined points distal to the proximal nerve stump were digitalized and semi-automatically analysed (7) for the number of regenerated myelinated axons, nerve fiber density as well as g-ratio of regenerated myelinated axons. To detect functional motor

recovery, electrodiagnostical measurements and evaluation of the nerve conduction velocity were performed by the end of the observation period.

Results

(I) Two and 8 weeks after surgery electrical stimulation did significantly increase the nerve density at midtransplanat level and at the distal gap end, respectively, in autotransplanted sciatic nerves. Furthermore, electrical stimulation did significantly increase the nerve conduction velocity (NCV) ratio ($NCV_{ipsilateral}/NCV_{contralateral}$) 8 weeks after surgery. Analysis of experimental paradigm **(II)** revealed better macroscopic tissue regeneration through silicone tubes after electrical stimulation and transplantation of naïve Schwann cells. **(III)** A combination of electrical stimulation with FGF-2$^{21/23kD}$ gene therapy did not further improve macroscopic tissue regeneration. However, this combination did result in a high rate of regenerated nerves that did already functionally reconnect to the target muscle 8 weeks after surgery.

Outlook

Further evaluation will elucidate if electrical stimualtion did also increase precision of motor end plate reinnervation.

We did demonstrate before, that adult human Schwann cells can be highly enriched and non-virally transfected (4, 5). Therefore in follow-up experiments, we will combine transplantation of genetically modified brain-derived neurotrophic factor (BDNF)-overexpressing human Schwann cells with electrically stimulation which is known to increase expression of the high affinity neurotrophin receptor trkB (2).

Acwnowlegments – Financial support: Stiftung neurochirurgische Forschung to KH and Hochschul-interne Leistungsförderung (HiLF) to KH.

References

1. Ahlborn, P., Schachner, M., and Irintchev, A. 2007. One hour electrical stimulation accelerates functional recovery after femoral nerve repair. Exp Neurol 208: 137-144.
2. Al-Majed, A. A., Brushart, T. M., and Gordon, T. 2000. Electrical stimulation accelerates and increases expression of BDNF and trkB mRNA in regenerating rat femoral motoneurons. Eur J Neurosci 12: 4381-4390.
3. Haastert, K., Lipokatic, E., Fischer, M., Timmer, M., and Grothe, C. 2006. Differentially promoted peripheral nerve regeneration by grafted Schwann cells over-expressing different FGF-2 isoforms. Neurobiol Dis 21: 138-153.
4. Haastert, K., Mauritz, C., Chaturvedi, S., and Grothe, C. 2007. Human and rat adult Schwann cell cultures: fast and efficient enrichment and highly effective non-viral transfection protocol. Nat Protoc 2: 99-104.
5. Haastert, K., Mauritz, C., Matthies, C., and Grothe, C. 2006. Autologous adult human Schwann cells genetically modified to provide alternative cellular transplants in peripheral nerve regeneration. J Neurosurg 104: 778-786.
6. Haastert, K., Ying, Z., Grothe, C., and Gomez-Pinilla, F. 2008. The effects of FGF-2 gene therapy combined with voluntary exercise on axonal regeneration across peripheral nerve gaps. Neurosci Lett 443: 179-183.
7. Timmer, M., Robben, S., Muller-Ostermeyer, F., Nikkhah, G., and Grothe, C. 2003. Axonal regeneration across long gaps in silicone chambers filled with Schwann cells overexpressing high molecular weight FGF-2. Cell Transplant 12: 265-277.

Schwann cell-like differentiated adipose-derived stem cells enhance peripheral nerve regeneration *in vivo*

P.G. di Summa,[1-2] P.J. Kingham,[2-3] W. Raffoul,[1] M. Wiberg,[3] G. Terenghi,[2] D.F. Kalbermatten[1]

[1]Chirurgie Plastique et Reconstructive CHUV, Université de Lausanne, CH; [2]Blond McIndoe Research Laboratories. The University of Manchester, Manchester, UK; [3]Departments of Surgical & Perioperative Science (Hand surgery) and Integrative Medical Biology (Anatomy), Umea University, Umea, Sweden

Traumatic injuries resulting in peripheral nerve lesions often require a graft to bridge the gap. Although autologous nerve auto-graft is still the first choice strategy in reconstructions, it has the severe disadvantage of the sacrifice of a functional nerve. Cell transplantation in a bioartificial conduit is an alternative strategy to create a favorable environment for nerve regeneration. We decided to test new fibrin nerve conduits seeded with various cell types (primary Schwann cells and adult stem cells differentiated to a Schwann cell-like phenotype) for repair of sciatic nerve injury. Two weeks after implantation, conduits were removed and examined by immunohistochemistry for axonal regeneration (evaluated by PGP 9.5 expression) and Schwann cell presence (detected by S100 expression). The results show a significant increase in axonal regeneration in the group of fibrin seeded with Schwann cells compared with the empty fibrin conduit. Differentiated adipose derived stem cells also enhanced regeneration distance in a similar manner to differentiated bone marrow mesenchymal stem cells. These observations suggest that adipose derived stem cells may provide an effective cell population, without the limitations of the donor site morbidity associated with isolation of Schwann cells, and could be a clinically translatable route towards new methods to enhance peripheral nerve repair.

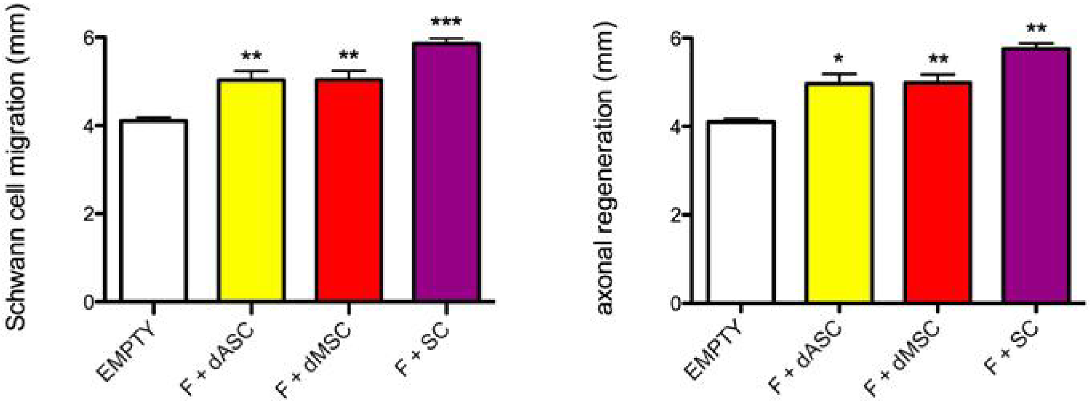

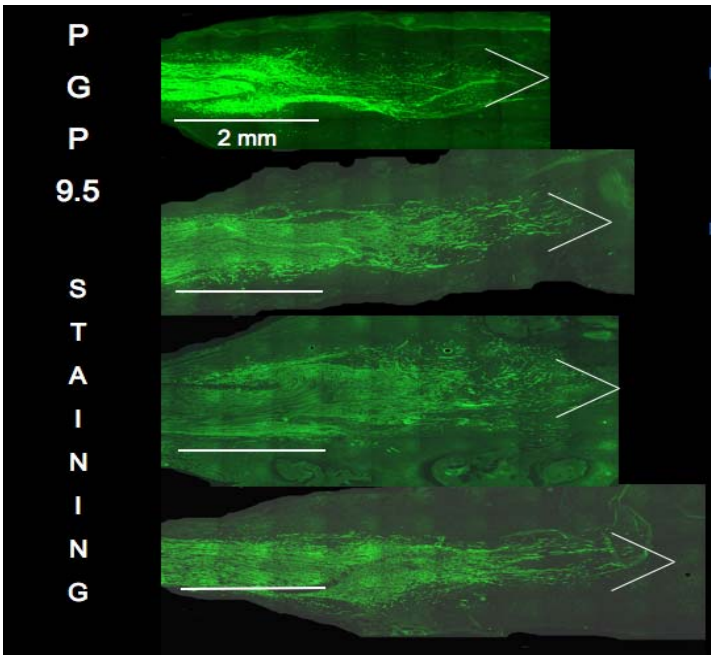

References

Caddick, J., Kingham, P. J., Gardiner, N. J., Wiberg, M., and Terenghi, G., 2006. Phenotypic and functional characteristics of mesenchymal stem cells differentiated along a Schwann cell lineage. Glia 54, 840-849

Dezawa, M., Takahashi, I., Esaki, M., Takano, M., and Sawada, H., 2001. Sciatic nerve regeneration in rats induced by transplantation of in vitro differentiated bone-marrow stromal cells. Eur J Neurosci 14, 1771-1776.

Kalbermatten, D. F., Pettersson, J., Kingham, P. J., Pierer, G., Wiberg, M., and Terenghi, G., 2009. New fibrin conduit for peripheral nerve repair. J Reconstr Microsurg 25, 27-33.

Kingham, P. J., Kalbermatten, D. F., Mahay, D., Armstrong, S. J., Wiberg, M., and Terenghi, G., 2007. Adipose-derived stem cells differentiate into a Schwann cell phenotype and promote neurite outgrowth in vitro. Exp Neurol 207, 267-274.

Tohill, M., and Terenghi, G., 2004. Stem-cell plasticity and therapy for injuries of the peripheral nervous system. Biotechnol Appl Biochem 40, 17-24.

Kalbermatten, D. F., Kingham, P. J., Mahay, D., Mantovani, C., Pettersson, J., Raffoul, W., Balcin, H., Pierer, G., and Terenghi, G., 2008. Fibrin matrix for suspension of regenerative cells in an artificial nerve conduit. J Plast Reconstr Aesthet Surg 61, 669-675.

Gimble, J. M., Katz, A. J., and Bunnell, B. A., 2007. Adipose-derived stem cells for regenerative medicine. Circ Res 100, 1249-1260.

Transplantation of olfactory ensheathing cells enhances peripheral nerve regeneration after microsurgical nerve repair

C. Radtke,[1,2,3] A.A. Aizer,[1,2] S.K. Agulian,[1,2] K.L. Lankfor,[1,2] P.M. Vogt,[3] J.D. Kocsis[1,2]

[1]Department of Neurology and Center for Neuroscience and Regeneration Research, Yale University School of Medicine, New Haven, CT 06510, USA; [2]Rehabilitation Research Center, Veterans Affairs Connecticut Healthcare System, West Haven, CT 06516, USA; [3]Department of Plastic, Hand- and Reconstructive Surgery, Hannover Medical School, 30659 Hannover, Germany

While axonal regeneration is more successful in peripheral nerve than in the central nervous system, it is by no means complete and research to enhance peripheral nerve regeneration is clinically important. Olfactory ensheathing cells (OECs) are known to enhance axonal regeneration and to produce myelin after transplantation. In contrast to Schwann cells their migratory potential and ability to penetrate glial scars is higher. This study evaluated the effect of OEC transplantation on microsurgically repaired sciatic nerves. Rat sciatic nerves were transected followed by microsurgical repair and transplantation of OECs or injection of medium without cells. Twenty-one days later the nerves were removed and prepared for either histology or electrophysiological analysis. Footprint analysis was carried out at 7, 14 and 21 days. The OECs survived and integrated into the repaired nerves as indicated by eGFP-expressing cells aligned with neurofilament identified axons bridging the repair site. Moreover, regenerated axons were myelinated by the transplanted OECs and nodes of Ranvier were formed. Conduction velocity in the OEC transplant group was increased in comparison to the microsurgical repair alone, and improved stepping was observed in the transplant group. These results suggest that presentation of OECs at the time of nerve injury enhances regeneration and improves functional outcome. Even a modest improvement in nerve regeneration could have significant clinical implications for reconstructive nerve surgery.

Rodent sciatic nerve-crush injuries: injury and recovery assessment

R. Pavic, M.L. Pavic

University Hospital Osijek, Medical School, JJ Strossmayer University, Osijek, Croatia; Email: roman.pavic@os.t-com.hr

The study of nerve regeneration markers after the deliverance of a crush injury allows us to study the changes and possibly enhance the environment to decrease inhibition of nerve regeneration. However, the importance of muscle ˝splitting˝ surgery to produce the experimental sciatic nerve crushes in both rat and mice models can not be underestimated. The original infliction of the crush injury and the subsequent wound healing have a strong impact upon an experiment. Following a crush injury to the sciatic nerve, rats and mice can be monitored in various ways in their functional recovery (1,2). The simultaneous tracking of changes found in the spinal cord segments following injury and during recovery offer an insight into the environment necessary for nerve regeneration, and further emphasize the differences in nerve recovery between these two rodent models.

References

1 Pavic R, Tvrdeic A, Tot OK, Heffer-Lauc M. 2007. Activity cage as a method to analyze functional recovery after sciatic nerve injury in mice. Somatosens Mot Res 24(4):213–219.

2 Pavic R, Pavic ML, Tot OK, Bensic M, Heffer-Lauc M. 2008. Side distinct sciatic nerve recovery differences between rats and mice Somatosens Mot Res, 25(3):163–170.

Sciatic nerve injury: functional recovery and split plot differences in rat and mouse models

R. Pavic, M.L. Pavic

University Hospital Osijek, Medical School, JJ Strossmayer University, Osijek, Croatia; Email: roman.pavic@os.t-com.hr

The use of sciatic functional indexing following sciatic nerve damage in rodent models has become a standard since the presentation by DeMedinaceli in 1982 (1). Although it has gone through revisions and improvements

the traditional SFI experiments typically show recovery within 3 weeks following a crush injury. Because of its relative ease both rat and mouse models were developed. During several nerve regeneration experiments with rats and mice the sciatic functional index was used to show recovery of each species. However as we proceeded the fact that there must be some sort of compensation involved following injury was continually being asked. To eliminate this from the data so that we could see the injured leg of each animal and how it progressed in its return to full function we developed a split plot which used the preoperative value for the unoperated leg throughout the sciatic functional index calculations. What was found was that the rats and mice reacted quite differently during the recovery of the sciatic crush injury (2). While the mouse model remained fairly true to the standard SFI plot when it was split, the rat model revealed that the unoperated leg compensated for the injury and that the compensation slowly decreased until the recuperation was complete (3).

References

1 De Medinaceli L, Freed WJ, Wyatt RJ. 1982. An index of the functional condition of rat sciatic nerve based on measurements made from walking tracks. Exp Neurol 77:634–643.

2 Dellon ES, Dellon AL. 1991. Functional assessment of neurologic impairment: Track analysis in diabetic and compression neuropathies. Plast Reconstr Surg Oct;88(4):686–694.

3 Pavic R, Pavic ML, Tot OK, Bensic M, Heffer-Lauc M. 2008. Side distinct sciatic nerve recovery differences between rats and mice Somatosens Mot Res, 25(3):163–170.

Forelimb vs hindlimb nerve regeneration experimental models

P. Tos,[1] I. Papalia,[2] G. Ronchi,[3] I. Perroteau,[4] B. Battiston,[1] S. Geuna[3]

[1]Department of Orthopaedics and Traumatology, CTO Hospital, Turin, Italy, [2]Department of Department of Surgical Disciplines, University of Messina [3]Department of Clinical and Biological Sciences, [4]Department of Animal and Human Biology, University of Turin

Peripheral nerve regeneration research is usually based on the employment of the sciatic nerve model which still represents a valid experimental approach due to the several behavioral functional tests available (Varejão et al., 2004) and specifically the computerized gait analysis (Luis et al., 2007; Bozkurt et al., 2008).

Nevertheless, the use of the rat median nerve has been recently sustained mainly because animal welfare is more preserved (Papalia et al., 2003;2006;2007; Tos et al., 2009). Moreover, experimental results are more likely to be translated to the clinical practice (the main goal of most studies on experimental surgery) since the majority of surgical interventions for repairing a damaged human nerve are performed at the upper limb level. Additionally, the grasping function requires fine, skilled finger movement and the behavior is quite similar between rodents and humans (Whishaw et al., 1992).

The main limitation of this model is the small size of the median nerve which requires advanced microsurgical skills for performing epineurial suturing without causing any epineurial damage.

Therefore, it should be emphasized that our present knowledge not allow us to conclude that one of these two models is superior to the other and that researchers must choose the experimental model based on their specific requirements and expertise, knowing each model's limitations and using the results within those limitations, rather than hewing to a more rigid point of view about which model is best.

References

Bozkurt A, Deumens R, Scheffel J, O'Dey DM, Weis J, Joosten EA, Führmann T, Brook GA, Pallua N. CatWalk gait analysis in assessment of functional recovery after sciatic nerve injury. J. Neurosci. Methods, 2008a;173:91-8.

Luis AL, Amado S, Geuna S, Rodrigues JM, Simoes MJ, Santos JD, Fregnan F, Raimondo S, Veloso AP, Ferreira AJ, Armada-da-Silva PA, Varejao AS, Mauricio AC. Long-term functional and morphological assessment of a standardized rat sciatic nerve crush injury with a non-serrated clamp. J. Neurosci. Methods, 2007;163:92-104.Nichols et al., 2005

Papalia I, Tos P, Stagno d'Alcontres F, Battiston B, Geuna S. On the use of the grasping test in the rat median nerve model: a re-appraisal of its efficacy for quantitative assessment of motor function recovery. J. Neurosci. Methods, 2003;127:43-7.

Papalia I, Tos P, Scevola A, Raimondo S, Geuna S. The ulnar test: a method for the quantitative functional assessment of posttraumatic ulnar nerve recovery in the rat. J. Neurosci. Methods, 2006;154:198-203.

Papalia I, Cardaci A, d'Alcontres FS, Lee JM, Tos P, Geuna S. Selection of the donor nerve for end-to-side neurorrhaphy. J. Neurosurg., 2007;107:378-82. Varejão et al., 2004;

Tos P, Ronchi G, Papalia I, Sallen V, Legagneux J, Geuna S, Giacobini-Robecchi MG. Methods and protocols in peripheral nerve regeneration experimental research: part I-experimental models. Int. Rev. Neurobiol., 2009;87:47-79.

Varejão ASP, Cabrita AM, Meek MF, Bulas-Cruz J, Melo-Pinto P, Raimondo S, Geuna S, Giacobini-Robecchi MG. Functional and morphological assessment of a standardized rat sciatic nerve crush injury with a non-serrated clamp. J. Neurotrauma, 2004;21:1652-70.

Whishaw IQ, Pellis SM, Gorny BP. Skilled reaching in rats and humans: evidence for parallel development or homology. Behav. Brain Res., 1992;47:59-70.

Promoting regeneration of the injured peripheral nerve by combining neurosurgical repair with gene therapy

J. Verhaagen, M. Tannemaat, R. Eggers, M. Mason, M. Malessy

Netherlands Institute for Neuroscience, Amsterdam and Leiden University Medical Center, Leiden, The Netherlands

Despite great advancements in surgical repair techniques, a considerable degree of functional impairment remains in most patients after reconstructive peripheral nerve surgery. New approaches to promote peripheral nerve regeneration are needed since surgical repair has probably reached its optimal technical refinement. At least four major challenges exist to improve the clinical outcome of nerve repair. These can be summarized as follows:

1. It is necessary to enhance the number of regenerating axons and the velocity of axon growth. If this would be possible this would allow for more axons to rapidly grow over larger distances in shorter periods of time, preventing them from being trapped in chronically denervated nerve stumps that are non-permissive for axon growth.

2. Following severe injuries the lesion has to be bridged by sural nerve transplants. Although these sural nerve bridges are now the golden standard in neurosurgical repair, axon growth through these bridged is suboptimal. Therefore axon regeneration through nerve bridges has to be improved.

3. The formation of a growth-inhibiting scar or neuroma at the site of the lesion or at the coaptation site prevents axon regeneration. This inhibition has to be overcome.

4. The misrouting of sensory and motor axons leads to inappropriate innervation of target cells. Targeting sensory and motor axons to their appropriate and preferably their original target is a major challenge.

Here we will discuss efforts to develop gene therapy as an adjunct strategy to promote peripheral nerve regeneration following neurosurgical repair. Gene therapy can be defined as the introduction of genetic material into living cells with the aim of treating a disease, and the inserted gene is referred to as the transgene. Compared to the delivery of a therapeutic protein gene therapy has three main advantages:

1. Transduced cells will continuously express the therapeutic protein, transforming them in effect in "biological minipumps", and ensuring that the therapeutic protein molecules can act over extended periods of time.

2. Local expression of the transgene by transduced cells mimics the physiological situation (at least to some extent) and this may prevent effects on more distant cells that are not the target cells.

3. The effects of genes encoding for proteins with an intracellular function (e.g. transcription factors, intraneuronal growth-associated proteins) can be studied.

There are several methods to introduce genetic material in cells, but the use of viral vectors has emerged as the most efficient way to express a potentially therapeutic gene into the nervous system. Adeno-associated viral vectors (AAV) and lentiviral vectors (LV) are the two most commonly used vectors. AAV and LV vectors have very different cellular transduction profiles. AAV vectors predominantly transduce neurons. Moreover, at least 11 naturally occurring AAV serotypes have now been transformed into vectors that display very interesting differential and predominantly neuronal tropism. LV vectors transduce Schwann cells, astrocytes and fibroblasts but are quite inefficient when it comes to genetic modification of neurons. We have used AAV as well as LV vectors to investigate whether gene therapy could be a strategy to promote repair of peripheral nerves. These studies are in an initial stage, but we now have solid evidence that neurotrophic factor expression can be enhanced for several months in avulsed reimplanted ventral nerve roots and in injured rat peripheral nerves (Eggers et al. 2008, Tannemaat et al. 2008). We also have shown that the expression of NGF in human sural nerve segments can be enhanced by lentiviral vector-mediated introduction of the NGF gene (Tannemaat et al. 2007). Current studies first of all focus on refining the temporal and spatial delivery of neurotrophic factor genes to injured peripheral nerves (Eggers et al. 2009). Moreover we are investigating what would be the best way to promote the intrinsic growth-program of injured sensory and motor neurons. As a first step to achieve this goal a comparative study with seven different AAV serotype vectors (AAV1-6 and AAV8) demonstrated that up to 90% of lumbar sensory neurons can be transduced by a single intraganglionic injection of AAV5.

References

Tannemaat M., Boer G.J., Verhaagen, J., Malessy M. (2007) Genetic modification of human sural nerve segments results in enhanced expression of nerve growth factor. Neurosurgery 61: 1286-1296

Tannemaat, M.R., Eggers, R., Hendriks, W.T.J., De Ruiter, G.C.W., Van Heerikhuize, J.J., Pool, C.W., Malessy. M.J.A., Boer, G.J., Verhaagen, J. (2008) Differential effects of lentiviral vector-mediated overexpression of NGF and GDNF on regenerating

sensory and motor axons in the transected peripheral nerve. Eur. J. Neurosci. 28: 1467-1479

Eggers, R., Hendriks W.T.J., Tannemaat, M., Hoeben, R., Boer, G.J., Carlstedt, T.D., Verhaagen J. (2008) Neuroregenerative effects of the reimplantation of genetically modified spinal roots following avulsion of the spinal motor nerve. Molec. Cell. Neurosci. 39: 105-117

Eggers, R., Tannemaat, M.R., Ehlert, E.M., Verhaagen, J. (2009) A spatio-temporal analysis of motoneuron survival, axon regeneration and neurotrophic factor expression after lumbar ventral root avulsion and reimplantation. Exp. Neurol. (in press)

Gene therapy for promoting nerve regeneration in rats

F. Novati,[1] G. Papa,[1] S. Moimas,[2] S. Zacchigna,[2] L. Zentilin,[2] M. Giacca,[2] F. Fregnan,[3] G. Gambarotta,[3] I. Perroteau,[3] S. Geuna,[4] S. Raimondo,[4] Z.M. Arnež[1]

[1]Department of Plastic Surgery, University of Trieste; [2]Molecular Medicine Laboratory, ICGEB, Trieste; [3]Department of Animal and Human Biology, University of Turin; [4]Department of Clinical and Biological Sciences, Univeristy of Turin, Italy

Gene therapy is opening very promising perspectives for improving nerve regeneration (Tannemaat et al., 2008; Zacchigna and Giacca, 2009) The scope of our study was to verify the effects of VEGF on nerve and muscle. We developed an experimental model in rat consisting of creating a 1 cm gap in the median nerve in the forearm, which we then bridged by a scaffold consisting of a vein filled with non vascolarized muscle (muscle-vein-combined conduit). To deliver VEGF to the scaffold we took advantage of the AAV-VEGF vector. At 3 months, first the functional recovery of the repair was tested by the grasping test. Then the rats were sacrificed and the nerve ends and the scaffold were included in resin and taken to trial and analysis in high-definition optical microscopy, electron microscopy and molecular biological analysis. The results validated the delivery of AAV-VEGF vector to the scaffold. However, results showed that this type of vector had an anti-atrophic effect on the grafted muscle thus hindering successful nerve fiber regeneration.

We then decided to study the antiatrophic effect of the same carrier in a rat experimental model aimed to prevent muscle atrophy after denervation. The experimental model that we used was based on transection, under general anesthesia, of the median nerve leading to denervation of the deep and superficial flexor muscles of the fingers resulting in progressive muscle atrophy. In this study we evaluated the effect of injection of the superficial flexor muscles with AAV-VEGF and AAV-LacZ.

One month after injection the animals were sacrificed under general anesthesia, the muscles were then removed, immediately weighed and then processed for inclusion in resin for light microscopy and analysis in high definition. In a second experiment, the denervated muscles treated with AAV vector have been used for biomolecular analysis.

The results showed that the muscles treated with VEGF had a significant increase in weight (mean = 121mg, SD = 15mg) compared with controls (mean = 96mg, SD = 7mg). Histological and ultrastructural evaluation confirmed the improved trophism in muscles treated with AAV-VEGF. The assesment confirmed the biomolecular expression of the transgene in the treated muscles.

In conclusion, our results, although still preliminary, are very promising and indicate a possible innovative therapeutic strategy to prevent muscle atrophy caused by denervation in patients with peripheral nerve injuries. Our data also point to the muscle-vein-combined nerve conduits as a potentially effective mean for delivering AAV-VEGF during nerve regeneration, though the selection of the gene to be delivered should be done avoiding those genes which have an effect on muscle trophism.

References

Tannemaat, M.R., Eggers, R., Hendriks, W.T.J., De Ruiter, G.C.W., Van Heerikhuize, J.J., Pool, C.W., Malessy. M.J.A., Boer, G.J., Verhaagen, J. (2008) Differential effects of lentiviral vector-mediated overexpression of NGF and GDNF on regenerating sensory and motor axons in the transected peripheral nerve. Eur. J. Neurosci. 28: 1467-1479

Zacchigna, S., Giacca, M. (2009) Gene therapy perspectives for nerve repair. Int. Rev. Neurobiol. 87: 381-92

Activity-dependent strategies to enhance peripheral nerve regeneration and neural plasticity

E. Udina, E. Asensio-Pinilla, X. Navarro

Group of Neuroplasticity and Regeneration, Faculty of Medicine, Institut of Neurosciences, Universitat Autonoma Barcelona and CIBERNED, Bellaterra, Spain

Severe lesions to the peripheral nervous system lead to important functional deficits in the patients, due to the limited capacity of the nervous system to regenerate and to the misdirection of axonal growth. In parallel to the discontinuity of the axons, nerve injuries produce marked plastic changes in the spinal cord and the brain, that can interfere with functional recovery. Nerve lesions induces facilitation of spinal reflexes in relation to the severity of the lesion and to the reinnervation of the targets (Navarro et al., 2007). These changes last long time after the reinnervation of the targets and disturb the functional recovery after severe lesions. Maintenance of the activity in the neural circuits after lesions can be a key to modulate the plastic changes that neurons suffer due to the loss of synaptic and neurotrophic inputs.

A therapeutical option with a promising potential to improve functional recovery is the electrical stimulation of the injured nerve. Application of electrical stimulation proximal to the injury, at 20Hz for one hour after the nerve repair, accelerates axon growth across the suture (Al-Majed et al., 2000) .

The pro-regenerative effects of electrical stimulation in the peripheral nerve after lesions have been related to increased expression of neurotrophins in neuronal somas. Axotomized neurons receive signals through antidromic activation of the axon (Brushart et al., 2002). Electrical stimulation mimics, partially, the cellular response triggered by axotomy, as it accelerates the stimulated neuron switch from a neurotransmitter state to a pro-regenerative state, thus facilitating regeneration. However, electrical stimulation does not lead to spinal changes as strong as axotomy, neither in axonal elongation (Udina et al., 2008) nor in the cell reaction. Interestingly, however, electrical stimulation reverses the hyperreflexia provoked by axotomy (Vivo et al., 2008).

The advantage of electrical stimulation over other strategies to promote nerve regeneration is its feasible clinical application; indeed, a clinical study has shown the capacity to accelerate regeneration of the median nerve after carpal compressions (Gordon et al., 2007). Moreover, electrical stimulation shares some similarities with physical exercise, that promotes plasticity in the nervous system after lesions (Vaynman and Gomez-Pinilla, 2005) and accelerates axonal regeneration in peripheral nerve models (Molteni et al., 2004). Electrical stimulation is an artificial way to apply activity in the neuron, whereas exercise would be the natural way to provoke activity.

The aim of this study is to elucidate the role of neuronal activity, induced by nerve electrical stimulation and by exercise, in promoting axonal regeneration and modulating plasticity in the spinal cord after nerve injury. We further assessed the effects of electrical stimulation applied only acutely or daily during the regeneration phase after sciatic nerve injury and suture repair in rats. Moreover, we compared the effects of electrical stimulation with those of active exercise on a treadmill, and the possible additive effect when combining both activity-dependent treatments.

Three groups of animals received electrical stimulation at 3 V, 0.1 ms at 20 Hz just after lesion for 1h (ES1h), for 4h (ES4h) or 1h daily during 4 weeks (chronic; ESc). A fourth group was submitted to treadmill running during 4 weeks (5 m/min, 1h twice daily; TR). Another group received a combined treatment, 1h electrical stimulation after the lesion and treadmill exercise for the following 4 weeks (ES+TR). An untreated group served as control (C). Nerve conduction, H reflex and algesimetry tests were performed at 1, 3, 5, 7 and 9 weeks after surgery, to assess muscle reinnervation and changes in excitability of spinal cord circuitry. Histological analysis was made at the end of the follow-up. Groups that received acute ES and/or were forced to exercise in the treadmill showed higher levels of muscle reinnervation and increased numbers of regenerated myelinated axons when compared to control animals or animals that received chronic ES. Increasing the amount of ES received, by enlarging the time of stimulation (4h vs 1h) increased the improvement of muscle reinnervation. In contrast, daily ES was detrimental and the enhanced muscle reinnervation observed after the acute treatment was lost; muscle reinnervation and number of myelinated axons distal to the suture site were similar to the control group in animals that received ES chronically. Combining ESa with treadmill training significantly improved muscle reinnervation during the initial phase, showing an synergic effect. Interestingly, the facilitation of the monosynaptic H reflex in the injured limb was reduced in all groups with increased activity (exercise, acute and even chronic ES). Since chronic ES effectively modulates hyperreflexia but does not improve muscle reinnervation, electrical stimulation would modulate the hyperreflexia post-injury by a mechanism different to the one that “primes” the intact neuron leading to accelerated regeneration.

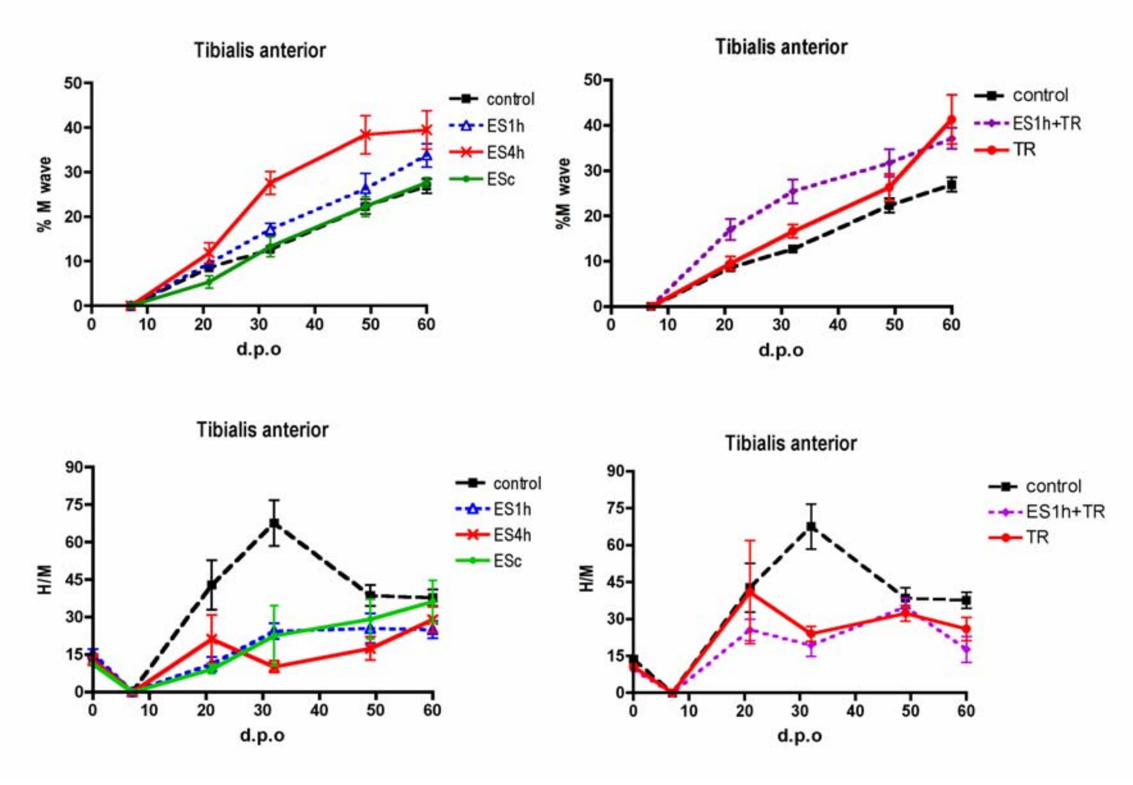

Recovery of the M wave amplitude in percentage with respect to contralateral control values for the tibialis anterior muscle (top pannels) and H/M ratio during follow-up (bottom pannels) in rats untreated (C), treated with 1h (ES1h), 4h (ES4h) of acute electrical stimulation or with daily 1h ES (ESc), and with treadmill training (TR) or combination of both (ES1h+TR) compared to control.

References

Al-Majed AA, Neumann CM, Brushart TM, Gordon T (2000) Brief electrical stimulation promotes the speed and accuracy of motor axonal regeneration. J Neurosci 20:2602-2608.

Brushart TM, Hoffman PN, Royall RM, Murinson BB, Witzel C, Gordon T (2002) Electrical stimulation promotes motoneuron regeneration without increasing its speed or conditioning the neuron. J Neurosci 22:6631-6638.

Molteni R, Zheng JQ, Ying Z, Gomez-Pinilla F, Twiss JL (2004) Voluntary exercise increases axonal regeneration from sensory neurons. Proc Natl Acad Sci U S A 101:8473-8478.

Navarro X, Vivo M, Valero-Cabre A (2007) Neural plasticity after peripheral nerve injury and regeneration. Prog Neurobiol 82:163-201.

Udina E, Furey M, Busch S, Silver J, Gordon T, Fouad K (2008) Electrical stimulation of intact peripheral nerve promotes the axonal regeneration of cut sensory fibers within the spinal cord. Exp Neurol 210(1):238-47.

Vaynman S, Gomez-Pinilla F (2005) License to run: exercise impacts functional plasticity in the intact and injured central nervous system by using neurotrophins. Neurorehabil Neural Repair 19:283-295.

Vivo M, Puigdemasa A, Casals L, Asensio E, Udina E, Navarro X (2008) Immediate electrical stimulation enhances regeneration and reinnervation and modulates spinal plastic changes after sciatic nerve injury and repair. Exp Neurol, 211(1):180-93.

New insights on neuroactive steroids in the peripheral nerve

R.C. Melcangi

Department of Endocrinology, Pathophysiology, and Applied Biology - Center of Excellence on Neurodegenerative Diseases, University of Milan, Via Balzaretti 9, 20133 Milano, Italy, FAX: +39-02-50318204; Tel. +39-02-50318238; Email: roberto.melcangi@unimi.it

Peripheral nerves are able to synthesize and metabolize neuroactive steroid and are also a target for these molecules, since they express classical and non-classical steroid receptors. Neuroactive steroids, such as progesterone, testosterone and their derivatives modulate the expression of key transcription factors for Schwann cell

function, regulate Schwann cell proliferation and promote the expression of myelin proteins involved in the maintenance of myelin multilamellar structure, such as myelin protein zero and peripheral myelin protein 22. These actions may result in the protection and regeneration of peripheral nerves affected by different forms of pathological alterations. Indeed, these neuroactive steroids are able to counteract biochemical, morphological and functional alterations of peripheral nerves in different experimental models of neuropathy, including the alterations caused by aging, chemotherapy, diabetes and physical injury (Leonelli, et al., 2007, Roglio, et al., 2009, Roglio, et al., 2007, Roglio, et al., 2008). Moreover, an interesting therapeutic strategy could be to increase the levels of neuroactive steroids directly in the nervous system by ligand of translocator protein-18 kDa (TSPO). TSPO is mainly present in the mitochondrial outer membrane, where it promotes the translocation of cholesterol to the inner mitochondrial membrane, and, as demonstrated it allows the transformation of cholesterol into pregnenolone and the increase of steroid levels. Indeed, in an experimental model of diabetic neuropathy we have recently demonstrated that a TSPO ligand, such as Ro5-4864, was effective in reducing the severity of diabetic neuropathy through a local increase of neuroactive steroid levels (Giatti, et al., 2009). Furthermore, we have recently observed that the levels of neuroactive steroids in the peripheral nerves show sex differences under basal conditions and are differently affected by diabetes in male and female rats (Pesaresi, et al., 2009). In agreement, neuroactive steroid levels are also sex different in an experimental model of peripheral neuropathy Charcot-Marie-Tooth type 1. Because peripheral neuropathies show sex differences in their incidence, symptomatology and nerodegenerative outcome (Melcangi and Garcia-Segura, 2009), findings here reported may have strong implications for the development of new sex-oriented therapies based on the use of neuroactive steroids, pharmacological agents able to increase their local synthesis or synthetic ligands for their receptors.

References

Giatti, S., Pesaresi, M., Cavaletti, G., Bianchi, R., Carozzi, V., Lombardi, R., Maschi, O., Lauria, G., Garcia-Segura, L. M., Caruso, D., and Melcangi, R. C., 2009. Neuroprotective effects of a ligand of translocator protein-18kDa (Ro5-4864) in experimental diabetic neuropathy. Neuroscience 164, 520-529.

Leonelli, E., Bianchi, R., Cavaletti, G., Caruso, D., Crippa, D., Garcia-Segura, L. M., Lauria, G., Magnaghi, V., Roglio, I., and Melcangi, R. C., 2007. Progesterone and its derivatives are neuroprotective agents in experimental diabetic neuropathy: a multimodal analysis. Neuroscience 144, 1293-1304.

Melcangi, R. C., and Garcia-Segura, L. M., 2009. Sex-specific therapeutic strategies based on neuroactive steroids: In search for innovative tools for neuroprotection. Horm Behav. doi:10.1016/j.yhbeh.2009.06.001

Pesaresi, M., Maschi, O., Giatti, S., Garcia-Segura, L. M., Caruso, D., and Melcangi, R. C., 2009. Sex differences in neuroactive steroid levels in the nervous system of diabetic and non-diabetic rats. Horm Behav. doi:10.1016/j.yhbeh.2009.04.008

Roglio, I., Bianchi, R., Camozzi, F., Carozzi, V., Cervellini, I., Crippa, D., Lauria, G., Cavaletti, G., and Melcangi, R. C., 2009. Docetaxel-induced peripheral neuropathy: protective effects of dihydroprogesterone and progesterone in an experimental model. J Peripher Nerv Syst 14, 36-44.

Roglio, I., Bianchi, R., Giatti, S., Cavaletti, G., Caruso, D., Scurati, S., Crippa, D., Garcia-Segura, L. M., Camozzi, F., Lauria, G., and Melcangi, R. C., 2007. Testosterone derivatives are neuroprotective agents in experimental diabetic neuropathy. Cell Mol Life Sci 64, 1158-1168.

Roglio, I., Bianchi, R., Gotti, S., Scurati, S., Giatti, S., Pesaresi, M., Caruso, D., Panzica, G. C., and Melcangi, R. C., 2008. Neuroprotective effects of dihydroprogesterone and progesterone in an experimental model of nerve crush injury. Neuroscience 155, 673-685.

The effect of melatonin and platelet gel on sciatic nerve repair: An electrophysiological and stereological study

S. Kaplan,[1] A. Piskin,[2] M. Ayyildiz,[3] A. Aktaş,[4] B. Koksal,[2] M. Basak-Ulkay,[4] A.P. Turkmen,[1] A. Turkmen,[1] F. Bakan[1]

[1]Dept. of Histology and Embryology, Ondokuz Mayis University School of Medicine, Samsun, Turkey, [2]Dept. of Orthopedic and Trauma Surgery, Ondokuz Mayis University School of Medicine, Samsun, Turkey, [3]Dept. of Physiology, Ondokuz Mayis University School of Medicine, Samsun, Turkey, [4]Dept. of Histology and Embryology, Istanbul University School of Veterinary Medicine, Istanbul, Turkey

Nerve regeneration after surgical reconstruction is far from being optimal and thus effective strategies for improving the outcome of nerve repair are being sought. In this experimental study, we verified if postoperative intraperitoneal melatonin administration after intraoperative platelet gel application improves peripheral nerve regeneration using collagen conduit sciatic nerve repair in the rat. In adult male rats, 1-cm long sciatic nerve defects were repaired using four different strategies: autologous nerve graft repair followed by melatonin administration, collagen conduit repair followed by melatonin administration, platelet gel-enriched collagen conduit repair followed by melatonin administration, and platelet gel-enriched collagen conduit repair followed by no substance administration. Sham operated animals were used as controls. Ninety days after surgery, the outcome of

nerve regeneration was comparatively assessed by means of electrophysiological and stereological analysis. Electrophysiology revealed no significant differences among the experimental groups and the sham control group. Stereology showed no significant differences among the experimental groups regarding axon size and myelin thickness. On the other hand, axon number was significantly lower in the melatonin group compared to the control and nerve graft + melatonin group. Moreover, there was no significant difference between the number of myelinated axons in the platelet gel and the control groups, between the platelet gel + melatonin and melatonin groups, and between the melatonin and the platelet gel groups. This study suggests that local platelet gel administration without postoperative melatonin administration has a positive effect on nerve repair. The combination of melatonin with platelet gel or melatonin alone did not show the same effect on the nerve repair.

Des-acyl ghrelin promotes peripheral nerve regeneration in a transgenic mouse model

A. Graziani,[1] N. Filigheddu,[1] P. Porporato,[1] C. Audisio,[2] I. Perroteau,[2] G. Ronchi,[2,3] S. Geuna,[3] S. Raimondo[3]

[1]Department of Clinical and Experimental Medicine, University of Piemonte Orientale "A. Avogadro", Novara; [2]Department of Animal and Human Biology, University of Torino, Torino; [3]Department of Clinical and Biological Sciences, University of Torino, Orbassano, Italy

The hormone Ghrelin (GHR) and its unacylated form des-acyl ghrelin (D-GHR) are receiving increasing attention because of their wide range of activities which include inhibition of apoptosis, regulation of cell differentiation, and stimulation or inhibition of proliferation of several cell types. Recently, interest about these hormones has raised also among neuroscientists because of their effect on the nervous system, especially the stimulation of neurogenesis in spinal cord, brain stem and hippocampus. Little is known regarding the role of these hormones on neural repair. To partially fill this gap, the aim of this study was to assess the effect of D-GHR on peripheral nerve regeneration in the mouse.

We used a strain of transgenic mice (aMHC/GHRL) in which overexpression of the ghrelin gene results in a 50-fold increase in circulating D-GHR levels without affecting acylated GHR circulating levels. Regeneration was assessed by both behavioral evaluation (grasping test) and stereological analysis of regenerated myelinated axons.

While no differences were detected, in normal conditions, in the peripheral nerve morphology and function between transgenic and wild type mice, results showed that, after nerve lesion, the increased presence of circulating D-GHR induced a significant faster functional recovery that was accompanied by a significant increase in the number and density of regenerated myelinated nerve fibers.

Altogether these results unveil a novel role of D-GHR as an agent which promotes posttraumatic neural regeneration in the Peripheral Nervous System.

Phototherapy in peripheral nerve injury for muscle preservation and nerve regeneration

S. Rochkind,[1,3] S. Geuna,[2] A. Shainberg[3]

[1]Division of Peripheral Nerve Reconstruction, Department of Neurosurgery, Tel Aviv Sourasky Medical Center, Tel Aviv University, Israel; [2]Department of Clinical and Biological Sciences, University of Turin, Italy; [3]Faculty of Life Science, Bar-Ilan University, Israel

Background

Posttraumatic nerve repair and prevention of muscle atrophy represent a major challenge of restorative medicine. Considerable interest exists in the potential therapeutic value of laser phototherapy for restoring or temporary preventing denervated muscle atrophy as well as enhancing regeneration of severely injured peripheral nerve.

Methods

Low power laser irradiation (laser phototherapy) was applied for treatment of rat denervated muscle in order to estimate biochemical transformation on cellular and tissue levels, as well as on rat sciatic nerve model after crush injury, direct or side-to-end anastomosis and neurotube reconstruction. Nerve cells' growth and axonal sprouting were investigated on embryonic rat brain cultures. The animal outcome allowed clinical double-blind, placebo-controlled randomized study which measured the effectiveness of 780-nm laser phototherapy on patients suffering from incomplete peripheral nerve injuries for 6 months up to several years.

Results

In denervated muscle, *animal study* suggests that function of denervated muscles can be partially preserved

by temporary prevention of denervation–induced biochemical changes. The function of denervated muscles can be restored, not completely but to a very substantial degree, by laser treatment, initiated at the earliest possible stage post-injury.

In peripheral nerve injury, laser phototherapy has a protective and immediate effect, it maintains functional activity of the injured nerve, decreases scar tissue formation at the injury site, decreases degeneration in corresponding motor neurons of the spinal cord and significantly increases axonal growth and myelinization.

In cell cultures, laser irradiation accelerates migration, nerve cell growth and fiber sprouting.

In a pilot, clinical, double-blind, placebo-controlled randomized study in patients with incomplete long-term peripheral nerve injury, 780-nm laser irradiation can progressively improve peripheral nerve function, which leads to significant functional recovery.

Conclusion

Laser phototherapy temporarily preserves the function of a denervated muscle, accelerates and enhances axonal growth and regeneration after peripheral nerve injury or reconstructive procedures. Laser activation of nerve cells, their growth and axonal sprouting can be considered as potential treatment of neuronal injury. Animal and clinical studies show the promoting action of phototherapy on peripheral nerve regeneration, which makes it possible to suggest that the time for broader clinical trials has come.

Changes in inhibitory cortical neurons induced by electrical stimulation of a transected peripheral nerve

C. Herrera-Rincon,[1] A. Sanchez-Jimenez,[1] C. Avendaño,[2] F. Panetsos[1]

[1]Neurocomputing and Neurorobotics Research Group, Universidad Complutense de Madrid, 28037 Madrid (e-mails: celia.herrer@opt.ucm.es, abelsanchez@bio.ucm.es, fivos.panetsos@opt.ucm.es); [2]Department of Anatomy, Histology and Neuroscience, Universidad Autonoma de Madrid, 28029 Madrid, Spain (e-mail: carlos.avendano@uam.es)

Introduction

Limb amputation brings about an irrecoverable loss of both motor and sensory functions for the patient. For an artificial member to be fully effective, therefore, it should ideally be interfaced to the sensory nerves involved through a neural prosthetic device, whereby coded information might be fed to the appropriate sensory processing domains of the Central Nervous System (CNS).

The present report is part of an ongoing study devoted to investigate the anatomical and electrophysiological effects that electrical signals generated by a neural prosthesis exert on relevant CNS regions, in an effort to help determine the limits of future neuroprostheses directly connected to CNS. Specifically, here we report on the consequences of a sustained electrical stimulation of the transected infraorbital nerve (IoN) on certain morphological characteristics of the trigeminal representation within the primary somatosensory cortex (SmI). The choice of the trigeminal tactile system in the rat is due to its highly topographic organization and the broad knowledge gathered on its anatomy, physiology, plasticity and behavior throughout the last few decades.

In previous works, we have demonstrated that the electrical stimulation applied to the proximal stump of the transected IoN through an implanted electrode reduces by more than 50% the volume loss of SmI subsequent to the massive deprivation caused by the transection, and helps maintain normal or nearly normal patterns of cytochrome oxidase (CyO) activity. Now, we investigate further the possible mechanisms of cortical reorganization induced by electrical stimulation by focussing on the population of cortical inhibitory interneurons that express the calcium-binding proteins (CaBPs), parvalbumin (PV) or calbindin D28k (Calb).

Materials and Methods

We used 12 adult female Wistar rats divided in three experimental groups according to the manipulation of the sensory input: 4 animals with intact nerve (Control or C-group); 4 animals with complete left infraorbital nerve (IoN) transection (Amputated or A-group) and 4 deafferented animals with complete left IoNtransection and subsequent electrical stimulation (square pulses of 100µs, 3.0V, at 20Hz, 12 hours/day) of the transected IoN (Prosthetic or P-group). After 4 weeks, animals were perfusion-fixed and their brains were removed and serially sectioned for immunohistochemical studies.

To evaluate the alterations in each animal, we quantified, by stereological methods, the number of neurons expressing PV or Calb in the granular and supragranular layers of the subregion of SmI representing the mystacial vibrissae (cortical layers I-IV of the posteromedial barrel subfield, or PMBSF). The statistical distribution of the immunoreactive cells was checked for normality (Shapiro-Wilks test) and homocedasticity (Levene test). The results were then examined by ANOVA and the least significant difference (LSD) test was used for post hoc multiple comparisons. Significance was set at 0.05.

Results

Estimates of PV-stained neurons in control animals (Table 1) revealed an average of 9,500 labeled neurons in layers I-IV of the PMBSF, with neurons in the left hemisphere exceeding by a non-significant 4% those in the right. In the A-group there was a significant loss of nearly 25% PV-stained neurons in the deafferented cortex (right; contralateral to the transection) vs. the "intact" (left; ipsilateral to the transection) cortex. In contrast, animals with sectioned peripheral nerve submitted to electrical stimulation (P-Group) showed a clear maintenance of PV-containing interneurons (8730.0±1388.5 for the ipsilateral versus 8889.5±1081.2 for the contralateral cortex, with a ratio in percentage of 102.6). Variance analysis show significant differences ($p<0.05$) between Control-Amputated and Amputated – Prosthetic Groups.

Immunostaining for Calb (Table 2) showed an average of 4,600 positive neurons in controls, with a nonsignificant 7% higher number in the left over the right cortex. In the A-group we found a very significant 31% loss of Calb-positive neurons in the hemisphere contralateral to the IoN transection. Rats in the P-group also revealed fewer Calb-immunoreactive cells in the contralateral cortex, but this loss was of only 13%, and this difference did not reach statistical significance when compared with the opposite hemisphere. Variance analysis show significant differences ($p<0.05$) only between Control and Amputated.

	Table 1. Number of PARV-immunoreactive neurons in the posteromedial whisker barrel subfield (PMBSF, layers I-IV) of the three experimental groups. (C) control group; (A) amputated; (P) prosthetic group. Data show intragroup mean ± S.D.			Table 2. Number of CALB-immunoreactive neurons in the posteromedial whisker barrel subfield (PMBSF, layers I-IV) of the three experimental groups. (C) control group; (A) amputated; (P) prosthetic group. Data show intragroup mean ± S.D.		
Experimental Group	Ipsilateral* cortex	Contralateral cortex	Contra/Ipsi in %	Ipsilateral* cortex	Contralateral cortex	Contra/Ipsi in %
Control	9728.0±1220.0	9341.5±1429.8	96.4	4463.8±454.4	4714.0±4957	107.1
Amputated	96278.0±1477.4	7394.0±2439.0	75.5	4198.8±862.1	2939.3±855.4	69.2
Prosthetic	8730.0±1388.5	8889.5±1081.3	102.6	4528.8±974.3	3809.3±188.1	87.4

* Ipsilatelaly to the transected IoN

Discussion

Our results show that, for a relatively short post-implantation survival time, extended sessions of electrical stimulation to a transected sensory nerve have measurable and significant effects on the CaBPs expression in the sensory input-deprived cortex. Cortical deafferentation is known to result in a local loss of inhibition, more or less temporary and reversible, depending on the deafferentation conditions. Our findings suggest that chronic electrical stimulation of the trasected nerve stump prevents the expected reduction of GABAassociated molecules and therefore the dampening of intracortical inhibitory circuits. This effect was particularly noticeable in the GABAergic PARV-positive neurons, the dominant GABAergic subclass in the barrel cortex, and also occurred, to a lesser degree, in GABAergic Calb-positive neurons.

Short of restoring the sensory capabilities, advanced neuroprostheses should help to maintain the cortical activity (and structure) near its normal values by preventing, or at least lessening, the deleterious effects of the deafferentation.

In addition, we are currently studying the effects of varying a number of stimulation parameters, using longer-term postlesion and stimulation periods, and testing the effects of neural stimulation with delays between amputation and electrode implants.

Reconnecting skeletal muscle to health spinal cord

M. Pizzi,[1] M. Francolini,[2] S. Barlati,[3] B. Guarneri,[4] M. Buffelli,[6] P.F. Spano,[1] F. Clementi,2 G. Brunelli[5]

[1]Div. of Pharmacology and Experimental Therapeutics of the Dept. of Biomedical Sciences and Biotechnologies, School of Medicine, University of Brescia; [2]Dept. of Medical Pharmacology - University of Milan and CNR Neuroscience Institute, Milan; [3]Div. of Biology and Genetics, [4]Div. of Neurophysiology of the Spedali Civili of Brescia; [5]Foundation for Experimental Spinal Cord Research; [6]Dept. of Neurological and Vision Sciences, Section of Physiology, University of Verona, Italy

Spinal cord injury bringing about traumatic paraplegia is still incurable as injured axons cannot regenerate into the CNS due the "non-permissive" milieu (1), and the lack of growth-promoting factors at the neuronal growth cone or

at the somata. However, evidence that PNs directly grafted into the CNS allow the severed central axons to extend throughout the peripheral endoneural tubes suggests that the latter could constitute a suitable environment for nerve regeneration (2). Thus, in order to bypass a spinal cord lesion, muscular nerve branches were inserted into the severed lateral bundle of spinal cord in primate (3). This procedure proved able to restore muscle innervation and motor function and raised the possibility that the re-growth of axons descending from central non-cholinergic neurons and cut during the grafting procedure could be responsible for functional muscle reinnervation. We recently tested this hypothesis (4).

An autologous sural nerve graft was implanted into the acutely severed lateral white matter of the spinal cord and connected to the transected cholinergic motor neuron of the internal obliquus abdominis muscle. This procedure produced functional muscle reinnervation after two months. Most interestingly, the restored neuromuscular activity became resistant to common curare blockers, but sensitive to a glutamate AMPA receptor antagonist. Analysis of the regenerated nerve disclosed the presence of markers for new glutamatergic axons and the disappearance of markers for cholinergic fibres. Regenerated axons derived from supraspinal neurons located in red nucleus and brainstem nuclei.

Finally, the innervated muscle displayed high expression and clustering of glutamate AMPA receptors. We studied the ultrastructure of reinnervated NMJs and the subunit composition of the AChRs. We detected higher glutamate-like immunoreactivity in the presynaptic terminals of the reinnervated NMJs. The postsynaptic apparatus of the NMJs innervated by glutamatergic axons expressed markers of glutamatergic synapses and, in particular, the AMPA receptor subunits partially colocalized with the AChRs and coimmunoprecipitate with rapsyn (5). Overall, these data suggest that under appropriate surgical manipulation supraspinal neurons can target skeletal muscle, while the latter retains the plasticity to generate a functional glutamatergic NMJ located at the preexisting cholinergic endplates.

References

1. Silver J, Miller JH: Regeneration beyond the glial scar. Nat Rev Neurosci 2004, 5:146-156.
2. Richardson PM, McGuinness UM, Aguayo AJ: Axons from CNS neurons regenerate into PNS grafts. Nature 1980, 284:264-265.
3. Brunelli GA, Brunelli GR: Experimental surgery in spinal cord lesions by connecting upper motoneurons directly to peripheral targets. J Peripher Nerv Syst 1996, 1:111-118.
4. Brunelli G, Spano PF, Barlati S, Guarneri B, Barbon A, Bresciani R, Pizzi M: Glutamatergic reinnervation through peripheral nerve graft dictates assembly of glutamatergic synapses at rat skeletal muscle. Proc. Natl. Acad. Sci. USA 2006, 102: 8752-8757
5. Francolini M., Brunelli G., Cambianica I., Barlati S., Barbon A., Luca L., Guarneri B., Boroni F., Lanzillotta A., Baiguera C., Ettorre M., Buffelli M., Spano P., Clementi F., Pizzi M. Glutamatergic reinnervation and assembly of glutamatergic synapses in adult rat skeletal muscle occurs at cholinergic endplates. J Neuropathol Exp Neurol. 2009, 68 (10) 1103-1115

Bridging peripheral short nerve defects with diverse sort of conduits. an overview of experimental novelties and clinical examples

I.A.Ignatiadis

Hand-Upper limb Surgery and Microsurgery Dept, KAT Hospital, Athens, Greece.

Nerve regeneration can occur through neural[1] and non-neural tissue used as conduits [2,3.]

In several experimental models the search for an optimal nerve conduit material led to the use of autogenous and more recently artificial materials in association without or with local administration of regeneration promoting factors called neurotrophic factors (local use of NPFs factors, NGF factor, factors promoting Schwann cells proliferation, treatment with TH) . Clinical implementation of conduits has focused on the use of autogenous tissue (veins[4], arteries, pseudoseaths[5], Frozen and thawed muscle, fresh muscle in vein grafts, nerve grafts) and occasionally of artificial conduits [6](silicone chambers, polyglicans and polyglactine mesh). Up to now, conduit materials does not seem to improve significantly the outcome compared to nerve grafting. After some series with patients treated by veins or muscles for bridging short defects with satisfactory results clinical case using successfully epineural sleeves have been recently reported.

The epineurium[7] may also serve as an autologous conduit, which may facilitate bridging of nerve defects. An autologous epineural tube has been used to bridge very short nerve defects. Positive Results have been obtained after studies concerning epineural flaps techniques to bridge a short nerve defect in the rabbit sciatic nerve defect model and another similar series combined with NGF s7 local administration.

More recently decellularized human allograft graft was used for the repair and replacement of damaged peripheral nerves. These grafts retain naturally occurring growth promoters and maintain the three-dimensional nerve structure. Of course the most important advantage of using the above artificial conduits, the epineurium flap and the nerve allograft is that we avoid to cause a donor site graft morbidity. Both artificial conduits and nerve

allografts costs a lot, while there is not at all cost by using epineural conduits for bridging nerve defects.

The major obstacle in the use of conduits is the limitation in the defect size that can be successfully bridged and is in humans approximately 2.5 to 3, 5 cm. In case of a larger defect typical nerve grafting with a native nerve graft (sural, brachial or antebrahial cutaneous autologous nerves) is necessary.

References

1. Millesi H. The nerve gap. Hand Clinics 1986;2:651–663.
2. Suematsu N. Tubulation for peripheral nerve gap: Its history and possibility. Microsurgery 1989;10:71–74.
3. Strauch B. Use of nerve conduits in peripheral nerve repair. Hand Clinics 2000;16:123–130.
4. Malizos KN, Dailiana ZH, Anastasiou EA, Soucacos PN. Neuromas and gaps of sensory nerves of the hands: Management using vein conduits. Am J Orthop 1997;26:481–485.
5. Mackinon SE, Dellon AL. A comparison of nerve regeneration across a sural nerve graft and a vascularised pseudosheath. J Hand Surgery A 1988;13:935–942.
6. Williams LR, Longo FM, Powell HC, Lundborg G, Varon S. Spatial temporary progress of peripheral nerve regeneration with a silicone chamber: Parameters for a bioassay. J Comp Neurol 1983;218:46–70.
7. Ignatiadis IA, Diverse types of epineural conduits for bridging short nerve defects. An experimental study in the rabbit. Microsurgery. 2007;27(2):98-104.

Natural based materials as scaffold for peripheral nerve regeneration

C. Tonda-Turo,[1] C. Audisio,[2] P. Gentile,[1] V. Chiono,[1] S. Geuna,[3] I. Perroteau,[2] G. Ciardelli[1]

[1]Politecnico di Torino, Department of Mechanics, Corso Duca degli Abruzzi 24, 10129 Turin, e-mail: chiara.tondaturo@polito.it, gianluca.ciardelli@polito.it; [2]Department Of Biological and Clinical Science, University of Turin; [3]Department Of Human and Animal Biology, University of Turin, Turin, Italy

Introduction

Nerve guides based on biodegradable and biocompatible polymers are one of the most promising strategies for peripheral nerve repair [1]. The inner tube part can be filled using specific matrices containing functional moieties to direct and accelerate the regeneration process. In this contest, the current trend of nerve tissue engineering is the realization of biomimetic NGCs, providing chemotactic, topological and haptotactic signalling to cells, respectively by surface functionalization with cell binding domains, the use of internal oriented matrices/fibres and the sustained release of neurotrophic factors[2,3]. Recently, natural-based biomaterials have been applied to artificial skin, bone grafts, and scaffolds for tissue engineering [4,5]. The main limitation of natural materials for application in tissue engineering is its solubility in aqueous solution; therefore, crosslinking is necessary to increase their stability in biological environment . In this study, crosslinked gelatin and chitosan substrates were studied to be used as materials for guiding the axonal growth.

Materials and Methods

Gelatin (GL, type A from porcine skin) and chitosan (CH, medium molecular weight) were supplied from Aldrich. Genipin (GP) was purchased from Challenge Bioproducts. GL was dissolved in demineralised water at 50°C to obtain a 2.5% (wt/v) solution. CH was dissolved in a 0.5 M acetic acid solution in water to obtain a 2.5% (wt/v) solution and its acid-insoluble fraction was removed by filtration. GP was added to the GL and CH solution at a 2.5 % wt/wt amount with respect to GL or CH. The resulting solution was kept under stirring at 50°C for 15 minutes, then cast films, porous sponges or electrospun substrates were obtained with the following procedure:

- Films were prepared by solvent casting;
- Porous scaffolds were fabricated by freeze-drying the solution after a freezing step at −20 °C overnight.

The physico-chemical properties of films, sponges and nanofibrous scaffolds were evaluated by swelling and dissolution tests, differential scanning calorimetry (DSC), infrared spectroscopy (FT-IR), stress-strain tests, scanning electron microscopy (SEM) and static contact angle measurements.

In vitro cells adhesion tests were performed using neonatal olfactory bulb ensheathing cells (NOBEC). Cell morphology was studied by SEM and fluorescence microscopy. Cells on the three different substrates (film, porous scaffolds and nanofibers) were quantified after nuclear staining using DAPI (4′,6-Diamidino-2-phenylindole dihydrochloride).

The GL and CH solutions were also prepared to fabricate hollow guide for peripheral nerve regeneration using the rotating mandrel technique.

Results and discussion

Crosslinking with GP increased GL and CH stability in water media, as shown by the decrease in the dissolution and swelling behaviour. Wettability of crosslinked materials was similar to that of uncrosslinked one (table 1). This result is within the optimal range for cell adhesion (50-70°).

Sample	GL	GL/GP	CH	CH/GP
Static Contact Angle	63° ± 1,4°	67,4° ± 6,1°	76° ± 5,6°	77,8° ± 1,7°

Table 1. Static contact angle values

The thermal properties of GL/GP and CH/GP were evaluated by DSC analysis, showing an increase in the denaturation temperature and a decrease in the denaturation enthalpy of crosslinked samples as compared to the uncrosslinked counterpart. The elastic modulus of films and porous scaffolds increased by GP crosslinking. Porous scaffolds showed interconnected pores with an average diameter in the range of 30-40µm. As regards the *in vitro* cells adhesion, GP crosslinked scaffolds supported NOBEC cells adhesion and proliferation: the number of adhered cells was similar as compared to the control (TCPS). Hollow guide for peripheral nerve regeneration were obtained using a rotating mandrel with a diameter of 1.3 mm (figure 1).

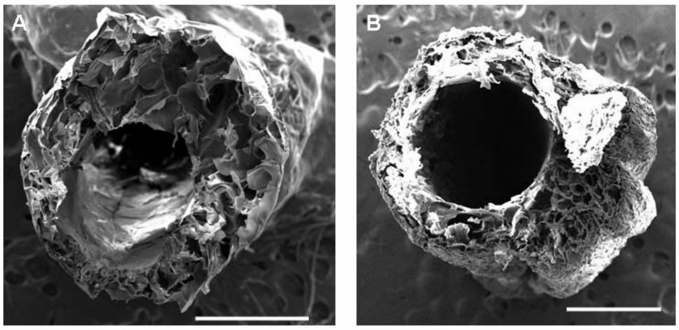

Fig.1. SEM images of (A) GP crosslinked GL and (B) GP crosslinked CH hollow guide (bar 1mm)

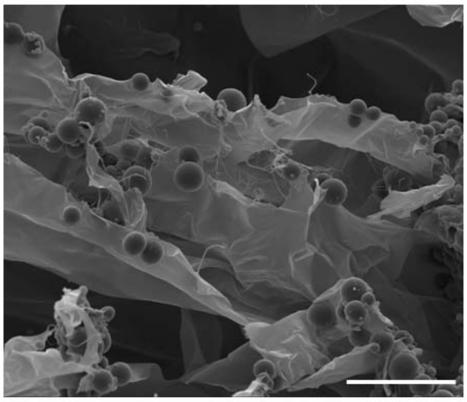

Fig.2. SEM images of nanoparticles embedded in CH/GL scaffold (bar 10 µm)

Conclusion

Crosslinked GL and CH are promising materials to be used as internal matrix to support and direct nerve regeneration process in the form of porous sponge. Moreover different techniques can be applied to obtain hollow tube as implantable devices for nerve regeneration. Future works will be focused on the delivery of active substance, such as growth factor, to accelerate the regeneration process. The factors can be encapsulated into nanoparticles and loaded onto the polymeric scaffold (fig 2).

References

[1] Ciardelli, G. and Chiono, V., 2006. Materials for peripheral nerve regeneration. Macromol. Biosci. 6,13–26

[2] Dodla, M. C., and Bellamkonda, R. V. , 2008. Differences between the effect of anisotropic and isotropic laminin and nerve growth factor presenting scaVolds on nerve regeneration across long peripheral nerve gaps. Biomaterials 29, 33–46.

[3] Ghasemi-Mobarakeh, L., Prabhakaran, M. P., Morshed, M., Nasr-Esfahani, M. H., and Ramakrishna, S., 2008. Electrospun poly(e-caprolactone)/gelatin nanofibrous scaVolds for nerve tissue engineering. Biomaterials 29, 4532–4539.

[4] Muzzarelli, R. A. A. , 2009. Chitins and chitosans for the repair of wounded skin, nerve, cartilage and bone. Carbohydr. Polym. 76, 167–182.

[5] Chen, Y. S., Chang, J. Y., Cheng, C. Y., Tsai, F. J., Yao, C. H., and Liu, B. S., 2005. An in vivo evaluation of a biodegradable genipin-cross-linked gelatin peripheral nerve guide conduit material. Biomaterials 26, 3911–3918.

Development of a new multichannel biodegradable conduit for peripheral nerve regeneration using nano-composite polymer

A. Pabari,[1,2] T. Sedaghati,[1] S.Y. Yang,[1] A. Mosahebi,[1,2] A.M. Seifalian[1*]

[1]*Centre for Nanotechnology, Biomaterials & Tissue Engineering, Division of Surgery & Interventional Sciences, University College London, London;* [2]*Department of Plastic Surgery, Royal Free Hampstead NHS Trust Hospital, London, United Kingdom;*
**Correspondence to: a.seifalian@medsch.ucl.ac.uk*

Introduction and Aims

Traumatic peripheral nerve injuries constitute a major source of chronic disability. Functional improvement following peripheral nerve injuries has plateaued.(Hart, Terenghi, and Wiberg, 2008;Wiberg and Terenghi, 2003) Advancement in tissue engineering and nanotechnology has made significant contribution to the medical devices industry by translating our understanding of basic sciences and pathophysiology. Management of peripheral nerve injuries has seen similar progress with the evolution of synthetic nerve tubes from infancy stage to commercially available conduits that have gained U.S. Food and Drug Administration (FDA) and Conformit Europe (CE) approval.(Meek and Coert, 2008;Schlosshauer, Dreesmann, Schaller, and Sinis, 2006) Although autologous nerve grafts are the current gold standard for bridging peripheral nerve defects,(Millesi, 1984) the morbidity associated with this technique, their limited supply and potential fascicular mismatch has sparked a huge interest amongst researchers to seek for suitable alternatives.

Current commercially available conduits are synthesised from either type I collagen or aliphatic polyesters. They are extruded as hollow tubes and do not mimic the natural architecture of the peripheral nerve. The natural architecture plays an important role in regulating cellular behaviours by influencing cells with biochemical signals and topographical cues and this may be one of the reasons for the current synthetic conduits not being able to support nerve regeneration in gaps longer than 30 mm and in large diameter nerve defects.

We have developed a multichannel, biodegradable nerve conduit from a new nano-composite polymer that is developed and patented by us. The aim of creating this nanofiber conduit is twofold. Firstly, the nanostructured scaffold will increase the cell-material interaction thus encouraging Schwann cell attachment and supporting its proliferation over longer nerve gap. Secondly, a combination of aligned nanofiber scaffold and macroscopic multiple channels will provide guidance for the axonal elongation thus supporting nerve regeneration in large diameter defects. Neither of these important features are present in current commercially available conduits.

Materials and Methods

The multi channel nerve conduit is made from a novel nanocomposite polymer by incorporating polyhedral oligomeric silsesquioxane (POSS) into polyhexanolactone and poly(carbonate-urea) urethane (PCU). The resultant biodegradable polymer has been awarded an international patent with a trade name of UCL-NanoBio™.(Seifalian, Handcock, and Salacinski, 2005) We have extruded this novel conduit by utilising the liquid ultrasonic atomization technology of Sono-Tek® to coat the polymer on a rotating mandrel with nano/micro channel mould pathway.

The cytocompatability of the novel nanocomposite polymer was evaluated by culturing rat Schwann cells on the casted polymer. Structural morphology of the polymer was studied by atomic force microscopy (AFM). Scanning electron microscopy was undertaken to study the morphology of the cultured Schwann cells on the nanofiber scaffold.

Results

The aligned nanofiber nerve conduit can be easily fabricated to any diameter, thickness and length by adjusting the diameter, length and the collecting time on the mandrel. AFM showed a “pebble-stone” blend throughout the scaffold which would increase the surface area for cell-material interaction. SEM revealed highly aligned nanofiber structure. Preservation of spindle-shaped cellular morphology of SC was observed. Immunochemistry confirmed the alignment of SCs along the length of the conduit. Furthermore, SCs had differentiated into myelinated lineage demonstrating their ability to support nerve regeneration.

Discussion

Bionanocomposites form a fascinating interdisciplinary area that brings together biology, material science and nanotechnology. Nanoscale features like in our new scaffold have the potential to improve the specificity of the biomaterial. The “pebble-stone” blend of our scaffold increases the surface area for the cells to anchorage on to the biomaterial. Furthermore, because extracellular molecules like collagen and fibrinogen assemble into nanofibers naturally, our nanocomposite biomaterial is as physiologically relevant to the Schwann cells as the nanoscale roughness of the polymer. With these two important features, we aim for guided axonal regeneration through our conduit and support for nerve regeneration over long gaps and in large diameter nerve injuries.

Conclusion

Nanofiber scaffolds can serve as powerful tools in the tissue regeneration processes. The fibrous structure closely resembles the natural environment that cells grow in and provides appropriate physical cues for manipulating cellular functions. The nanoscale features of our scaffold can interface more intimately with individual SC by mimicking the native extracellular environment thus making it potential candidate for the next generation of nerve conduits.

References

1. Hart, A.M., Terenghi, G., Wiberg, M., 2008. Neuronal death after peripheral nerve injury and experimental strategies for neuroprotection. Neurol. Res. 30, 999-1011.
2. Meek, M.F., Coert, J.H., 2008. US Food and Drug Administration /Conformit Europe- approved absorbable nerve conduits for clinical repair of peripheral and cranial nerves. Ann. Plast. Surg. 60, 466-472.
3. Millesi, H., 1984. Nerve grafting. Clin. Plast. Surg. 11, 105-113.
4. Schlosshauer, B., Dreesmann, L., Schaller, H.E., Sinis, N., 2006. Synthetic nerve guide implants in humans: a comprehensive survey. Neurosurgery 59, 740-747.
5. Seifalian, A.M., Handcock, S., Salacinski, H.J. Polymer for Use in Conduits and Medical Devices. WO2005070998. 2005.
6. Wiberg, M., Terenghi, G., 2003. Will it be possible to produce peripheral nerves? Surg. Technol. Int. 11, 303-310.

In vivo evaluation of polysialic acid as candidate substance for the development of new nerve graft materials

C. Grothe,[1,2] J. Schaper-Rinkel,[1,2] B. Rode,[3] R. Gerardy-Schahn,[2,4] T. Scheper,[3] K. Haastert[1,2]

[1]Hannover Medical School, Institute of Neuroanatomy, Hannover; [2]Center for Systems Neurosciences (ZSN) Hannover; [3]Leibnitz Universität Hannover, Institute of Technical Chemistry, Hannover; [4]Hannover Medical School, Institute of Cellular Chemistry, Hannover, Germany;
Contacts: Grothe.Claudia@mh-hannover.de; Haastert.kirsten@mh-hannover.de

Objective

The work presented here prospects towards the use of polysialic acid (polySia) as basis material for the development of nerve conduits. PolySia is a relatively simple molecule consisting of a linear chain of alpha2,8 linked 5-N-acetylneuraminic acid residues and occurs in mammals as a unique posttranslational modification of the neural cell adhesion molecule NCAM (6). PolySia is one of the major guidance cues in the developing nervous system. While nerves that have made stable contacts lose polySia-expression, the molecule re-appears after nerve injury and accounts for selective reinnervation of motor targets (1). In a series of recent in vivo studies it has been demonstrated that artificial induction or down-regulation, respectively, of polySia-expression at the nerve lesion site modifies peripheral nerve regeneration (2, 7). We have, in a systematic screen, evaluated the substrate properties of the soluble and immobilized polySia analog colominic acid and of degradation products thereof. Our data clearly demonstrate that polySia provides a favourable substrate for primary neurons and glia cells in vitro (4, 5). Here we show first in vivo results after incorporation of soluble polySia into cell-free or Schwann-cell containing silicone tubes.

Materials and Methods

(I) In a first study, 10 mm sciatic nerve gaps in adult female Sprague Dawley rats were bridged by silicone tubes filled with growth factor reduced matrigelTM (matrigel) (A) alone, (B) plus soluble polySia, (C) plus Schwann cells, (D) plus polySia and Schwann cells. The regeneration of gap bridging tissue as well as the quantity and quality of regenerated myelinated axons was analyzed with histological and semi-automated morphometrical methods (3). Furthermore, electrodiagnostical measurements were utilized to determine the status of motor recovery 8 weeks after surgery. In two additional groups we transplanted Schwann cells which have been pre-labeled with the fluorescent cell-linker PKH26-GL in matrigel (E) or in matrigel plus polySia (F) and analyzed the survival of these cells 6 weeks after surgery in longitudinal cryosections through the regenerated tissue. (II) In a second study a 13 mm nerve gap was either bridged by tubes filled with Matrigel plus polySia plus Schwann cells like in experimental group D (see above) or by autologous nerve grafts (clinical standard). Over 10 weeks the functional sensory recovery was monitored using the pinch-test. And electrodiagnostical measurements revealed functional motor recovery at 10 weeks post surgery.

Results

(I) Eight weeks after surgery, polySia treated animals showed significantly enhanced numbers of regenerated myelinated axons. Furthermore, presence of polySia did not interfere with the outcome of motor recovery (electrodiagnostic measurements). Pre-labeling of transplanted Schwann cells and retrograde tracing experiments revealed no negative effects of exogenous polySia on Schwann cell survival and regenerating motor and sensory neurons. (II) With regard to functional recovery, a higher speed of sensory recovery after transplantation of exogenous polySia–containing nerve bridges compared to autotransplantation conditions was determined over 10 weeks post surgery within the pinch-

test. Finally, electrodiagnostic measurements revealed 10 weeks post surgery reinnervation of the gastrocnemius muscle in 30% of the polySia-grafted animals but 100% in the group implanted with autologous grafts.

Outlook

We demonstrate here biocompatibility of exogenous polySia. In the next step chemically immobilized polySia-preparations will be developed, e.g. nanofibers electrospun on a polySia-basis or polySia immobilized on silica nanoparticles. New polySia based materials will then be tested for their suitability to bridge peripheral nerve gaps and to provide a container for transplanted Schwann cells.

Acknowledgments – Financial support: German Research Foundation (DFG – FOR 548-GR-857/20-3&24-1) to CG.

References

1. Franz, C. K., Rutishauser, U., and Rafuse, V. F. 2005. Polysialylated neural cell adhesion molecule is necessary for selective targeting of regenerating motor neurons. J Neurosci 25: 2081-2091.
2. Gravvanis, A. I., Lavdas, A. A., Papalois, A., Tsoutsos, D. A., and Matsas, R. 2007. The beneficial effect of genetically engineered Schwann cells with enhanced motility in peripheral nerve regeneration: review. Acta Neurochir Suppl 100: 51-56.
3. Haastert, K., Lipokatic, E., Fischer, M., Timmer, M., and Grothe, C. 2006. Differentially promoted peripheral nerve regeneration by grafted Schwann cells over-expressing different FGF-2 isoforms. Neurobiol Dis 21: 138-153.
4. Haile, Y., Berski, S., Drager, G., Nobre, A., Stummeyer, K., Gerardy-Schahn, R., and Grothe, C. 2008. The effect of modified polysialic acid based hydrogels on the adhesion and viability of primary neurons and glial cells. Biomaterials 29: 1880-1891.
5. Haile, Y., Haastert, K., Cesnulevicius, K., Stummeyer, K., Timmer, M., Berski, S., Drager, G., Gerardy-Schahn, R., and Grothe, C. 2007. Culturing of glial and neuronal cells on polysialic acid. Biomaterials 28: 1163-1173.
6. Johnson, C. P., Fujimoto, I., Rutishauser, U., and Leckband, D. E. 2005. Direct evidence that neural cell adhesion molecule (NCAM) polysialylation increases intermembrane repulsion and abrogates adhesion. J Biol Chem 280: 137-145.
7. Jungnickel, J., Bramer, C., Bronzlik, P., Lipokatic-Takacs, E., Weinhold, B., Gerardy-Schahn, R., and Grothe, C. 2009. Level and localization of polysialic acid is critical for early peripheral nerve regeneration. Mol Cell Neurosci 40: 374-381.

Polymeric micro-channel electrodes for improved recording in peripheral nerve implant

S.P. Lacour,[1] J.J. FitzGerald,[2] N. Lago,[3] S. Benmerah,[4] E. Tarte,[4] S. McMahon,[4] J. Fawcett[2]

[1]Nanoscience Centre, University of Cambridge (spl37@cam.ac.uk), [2]Centre for Brain Repair, University of Cambridge, [3]Neurorestoration Group, King's College London, [4]Department of Electrical Engineering, University of Birmingham, UK

We have developed a novel design of peripheral nerve interface. By placing axons into polymeric micro-channels hosting embedded electrodes the extracellular amplitude of action potentials is greatly increased, allowing for robust recording, noise suppression and efficient stimulation.

Figure 1 presents a sketch of the concept and pictures of current prototypes. The neural interface is a three dimensional bundle of parallel micro-channels in an insulating substrate (Fig. 1b) hosting embedded micro-electrodes that are exposed to the interior of the channels. It is housed within a silicone tube, into the ends of which the proximal and distal stumps of the severed nerve are sutured.

The micro-channel nerve interface is made initially as a flat plastic strip with open-topped channels, using standard planar photolithographic processes to integrate metallic electrodes directly on compliant polyimide films [2,3]. The strip is then rolled up into a cylinder so that the base layer of each turn of the roll roofs over the channels in the previous turn, yielding an array of closed micro-channels. The embedded electrodes (Fig. 1b-inset) are connected by conductive tracks within the substrate to contact pads at the side of the device that can be linked to external electronic circuitry using a compliant cable. Fig. 1c shows a picture of 2 complete rolled implants with zif (zero insertion force) connector inserted on the array contact pads.

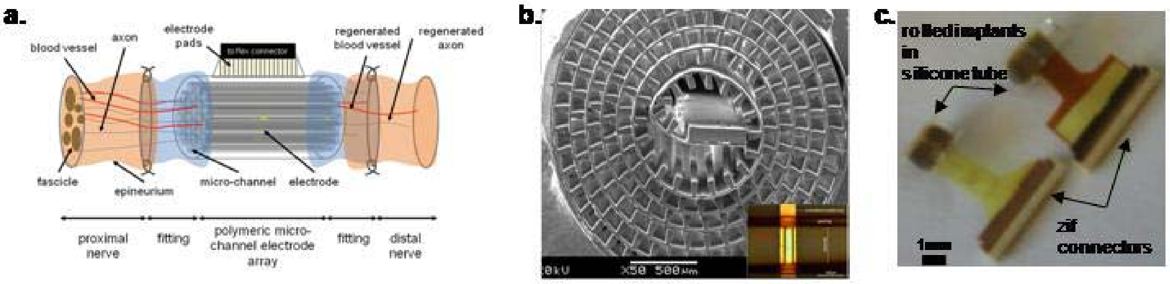

Figure 1. Micro-channel electrode implants. (a) 3D sketch of the device. (b) Cross-section of a polyimide roll micro-channels. Each channel is 0.1mm side. Inset: Electrode embedded in polyimide. (c) Picture of two complete rolls interfaced to zif connector [1].

We have demonstrated that micro-channel electrode arrays with 100 μmx100 μm cross-section (or 110 μm in diameter) channels support axon regeneration well, and that micro-channels of similar calibre and up to 5mm long can support axon regeneration and vascularisation. Electron microscopy images (Fig. 2) taken from the distal nerve immediately after the implant illustrate regenerated "mini-nerves" along with blood vessel and regenerated myelinated axons. Robust vascularisation is observed through most micro-channels.

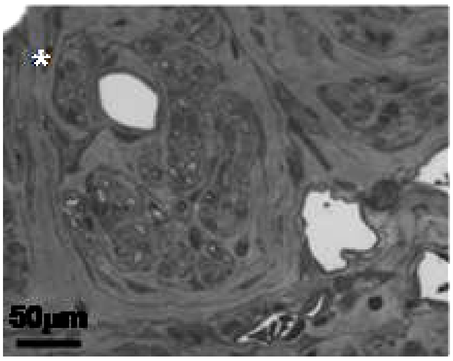

Figure 2. EM section of a mini-nerve regenerating through a 100μmx100μm, 2.5mm long rolled implant, 30 days post-implantation. EM sections of the distal nerve [1].

Action potentials can be reliably recorded extracellularly using the novel implant. Micro-channel confinement of axons results in significant amplification of the recorded extra-cellular signal, and free positioning of the electrodes in the channels (no longer at the nodes of Ranvier) [4]. Not only is the extracellular signal small but it can easily be submerged by electrical interference, mostly EMG from surrounding muscles. Tripolar electrode set-ups, similar to whole-nerve cuff electrode arrangements, provide an efficient means of noise reduction, allowing the recovery of AP signals from EMG noise of much higher amplitude. Micro-channels were also found to be efficient stimulators [5]. Stimulation was achieved using extremely small currents and short duration pulses, typically 1μA for 50μs, equivalent to a charge injection around ten times lower than existing intraneural stimulation devices. Using an asymmetric tripole arrangement, uni-directional stimulation of APs was also demonstrated. This is particularly important for neuroprosthesis applications where it may highly desirable to stimulate only afferent or efferent fibres.

Acknowledgments – This work is supported in part by the EPSRC-MRC Basic Technology Program (EP/C52330X) on 'Bioelectronic interfaces for peripheral nerve repair', by an MRC/Royal College of Surgeons of England fellowship awarded to JJF, and a University Research Fellowship from the Royal Society to SPL.

References

[1] S. P. Lacour, J. FitzGerald, N. Lago, E. Tarte, S. McMahon, and J. Fawcett, "Long micro-channel electrode arrays: a novel type of regenerative peripheral nerve interface," IEEE Trans. on Neural Systems and Rehabilitation Engineering, vol. on line, 2009.

[2] S. P. Lacour, R. Atta, J. FitzGerald, M. Blamire, E. Tarte, and J. Fawcett, "Polyimide micro-channel arrays for peripheral nerve regenerative implants," Sensors and Actuators A, vol. 147, pp. 456-463, 2008.

[3] S. Benmerah, L. S.P., and T. E, "Design and fabrication of neural implant with thick micro-channels based on flexible polymeric materials" in IEEE EMBS Conference Minneapolis: IEEE, 2009.

[4] J. FitzGerald, S. P. Lacour, S. McMahon, and J. Fawcett, "Microchannels as axonal amplifiers," IEEE Transactions on Biomedical Engineering, vol. 55, pp. 1136-1146, 2008.

[5] J. FitzGerald, S. P. Lacour, S. McMahon, and J. Fawcett, "Microchannel electrodes for recording and stimulation: in vitro evaluation," IEEE Transactions on Biomedical Engineering, vol. 56, pp. 1524-1534, 2009.

Biomaterials and cellular systems used for nerve regeneration

M.J. Simões,[1] S. Amado,[2] A. Gärtner,[1] P.A.S. Armada da Silva,[2] S. Raimondo,[3] M. Vieira,[1] A.L. Luísa,[4] A.P. Veloso,[2] A.S.P. Varejão,[5] S. Geuna,[3] A.C. Maurício[1,4*].

*[1]Centro de Estudos de Ciência Animal (CECA), Instituto de Ciências e Tecnologias Agrárias e Agro-Alimentares (ICETA), Universidade do Porto (UP); [2]Faculdade de Motricidade Humana (FMH), Universidade Técnica de Lisboa (UTL), Portugal; [3]Department of Clinical and Biological Sciences, University of Turin, Italy; [4]Departamento de Clínicas Veterinárias, Instituto de Ciências Biomédicas Abel Salazar (ICBAS), Universidade do Porto (UP); [5] Departamento de Ciências Veterinárias, CETAV, Universidade de Trás-os-Montes e Alto Douro (UTAD), Portugal. * Presenting author's email: ana.colette@mail.icav.up.pt; ana.colette@hotmail.com*

Recent advances in nerve Tissue Engineering have greatly promoted the generation of biomaterials, which may be implanted empty, or may be filled with growth factors, and / or stem cell auto or allografts for nerve regeneration. Mesenchymal stem cells (MSCs) comprise a rare population of multipotent progenitors capable of supporting hematopoiesis and differentiating into several lineages (osteogenic, neurogenic, myogenic, among others). Due to this ability confirmed by the results of both in vitro experiments and in vivo studies, MSCs appear to be an attractive tool in the context of Tissue Engineering and cell-based therapy. Currently, bone marrow represents the

main source of MSCs for both experimental and clinical applications. However the number of bone marrow MSCs significantly decreases with age and the bone marrow HLA compatible donors are very difficult to find, which makes the search for adequate alternative sources of these cells necessary for autologous and allogenic use. The stem cells obtained from the blood and Wharton´s jelly of the umbilical cord are a promising source of stem cells: i) the number of stem cells per volume is higher than in bone marrow, ii) a complete or high HLA profile match for allogenic use is not necessary, which permits to greatly enlarge the number of available donors iii) the stem cells are easier to obtain, manipulate and criopreserve, and their collection in ethically approved by national and international laws.

A multidisciplinary team, including Veterinaries, Engineers, Medical doctors like neurologists and surgeons through Experimental Surgery has a crucial role in the development of biomaterials associated to these cellular systems, and in testing the surgical techniques that involve their application, always considering animal welfare and the most appropriate animal model. Several biomaterials developed by our research group (including PLGA with a novel proportion 90:10 of the two polymers, poly(L-lactide):poly(glycolide), hybrid chitosan and collagen) have been tested associated to cellular systems to promote nerve regeneration after axonotmesis and neurotmesis injuries in the sciatic nerve experimental model. The cellular systems that have been studied in this context include an immortalized neural cell line N1E-115, stem cells obtained from the Wharton´s jelly of the umbilical cord, CD34+ stem cells from the umbilical cord blood, and MSCs from umbilical cord matrix. The tube-guides associated to one of the cellular systems are tested in the rat sciatic nerve across a 10 mm-gap (neurotmesis) or in a 3 mm axonotmesis lesion. The cells introduced are able to produce growth factors in the local of the nerve injury, during the necessary healing period.

Under general anesthesia, the sciatic nerve is unilaterally exposed. After nerve mobilization, a transection injury is performed, just above the terminal nerve ramification (neurotmesis). For the crush injury, a non-serrated clamp exerting a force of 54N is used for a period of 30 seconds to create a 3 mm long crush injury, 10 mm above the bifurcation. For reconstruction, the biodegradable tubes/membranes covered by the cellular system are used, for a nerve gap of 10 mm or to involve the axonotmesis lesion area, respectively. Motor functional recovery after the sciatic nerve reconstruction is assessed serially using video recording of the gait for biomechanical analysis, by measuring extensor postural thrust (EPT), sciatic functional index (SFI) and sciatic functional index under static conditions (SSI). The sensitive recovery is tested by the withdrawal reflex latency (WRL) and von Frey filaments. The repaired nerves are processed for light and electronic microscope analysis, imunohistochemistry, confocal microscopy and stereological studies. Functional and morphologic results obtained with these different biomaterials and cell lines are presented and discussed in terms of better improvement of nerve regeneration after axonotmesis and neurotmesis injuries in rat sciatic model.

Biodegradable hydrogels as scaffolds for nerve regeneration

V. Magnaghi,[1] E. Ranucci,[2] F. Fenili,[2] P. Procacci,[3] G. Pivato,[4] P. Cortese,[4] P. Ferruti[2]

[1]Department of Endocrinology, Physiopathology, Applied biology, Via Balzaretti 9, University of Milan, 20133 Milan; [2]Department of Organic and Industrial Chemistry, University of Milan, Via Venezian 21, 20133 Milan,; [3]Department of Human Morphology and Biomedical Sciences - Citta' Studi, University of Milan, Via Mangiagalli 31, 20133 Milan; [4]Hand Surgery Unit, IRCCS Multimedica, Via Milanese 300, 20099 Sesto San Giovanni, Italy

Transected peripheral nerves are typically reconnected by direct end-to-end surgery or by autologous nerve graft. However, artificial synthetic guide are a successful alternative which may prevent neuroma formation (1). Among biodegradable conduits a novel approach is represented by use of tuneable polyamidoamine (PAA)-based hydrogels, with specific diameters, different shapes and/or dimensions. Depending by their crosslinking degree, hydrogels made by PAAs are tough material which may absorb large amounts of water. PAA hydrogels are biocompatible and biodegradable in vitro to non-toxic low molecular weight products over a period of time varying from few weeks to months (2). In order to evaluate their ability to promote nerve regeneration, PAA hydrogels scaled as scaffold conduits (10mm lenght, 1mm internal diameter) were studied by using an experimental model of rat nerve transection. A conduit was used to join a gap of 4-5 mm in the sciatic nerve, and a longitudinal analysis was made at 30, 45, 60, 90 days post-surgery. We performed the gait analysis to evaluate locomotor coordination, the plantar test to study nociception and pain sensitivity, and the morphological-morphometric analysis to evaluate the nerve recovery. Preliminary results indicate that nerve ends can be successfully joined by these PAA-based hydrogel conduits.

One month after surgery, in fact, the regeneration is appreciable inside the conduit and the nerve is resistant to mechanical traction, without signs of inflammation or serum infiltrate. In the implanted rats 45 days after surgery the footprints analysis reveals a trail similar to sham-operated animals, while the thermal hypersensitivity tend to normalize to the control levels at later times. The

morphological evaluation of the explanted conduit at 90 days after surgery shows normal myelin structures, confirming nerve regeneration and complete scaffold re-absorption. In conclusion, our results demonstrate that PAA hydrogels might be a promising scaffold tube for nerve regeneration. Further studies on the hydrogels functionalization for drug delivery, with growth factors or hormones, are in progress in our labs.

References

1. 1. Yannas, I.V., Hill, B.J., 2004. Selection of biomaterials for peripheral nerve regeneration using data from the nerve chamber model. Biomaterials 25, 1593-1600.
2. Jacchetti, E., Elimitri, E., Rodighiero, S., Indrieri, M., Gianfelice, A., Lenardi, C., Podestà, A., Ranucci, E., Ferruti, P. Milani, P., 2008. P. Biomimetic poly(amidoamine) hydrogels as synthetic materials for cell culture. J. Biothecnol. 6, 14

Tissue engineered guided regenerative gel for recovery of peripheral nerve injury with massive loss defect

S. Rochkind, M. Alon, M. Graif, A. Shahar, Z. Nevo

Division of Peripheral Nerve Reconstruction, Departments of Neurosurgery, Rehabilitation and Radiology, Tel Aviv Sourasky Medical Center, Tel Aviv University; NVR labs, Ness Ziona; Department of Human Molecular Genetics and Biochemistry, Sackler School of Medicine, Tel Aviv University, Israel

Background

Guided Regeneration Gel (GRG) was developed to simulate the extracellular milieu, support growth and activity of axons and cells in vitro and in vivo upon implantation, as well as destined to serve as a regenerative and repair source for nerve tissue reconstruction.

Purpose

Evaluation of the efficacy of GRG based on tissue-engineering technology for the treatment of complete peripheral nerve injury with significant loss defect.

Methods

Rat sciatic nerve was completely transected and a 2 cm segment of the peripheral nerve was removed. Composite transplant, containing a guiding tube filled with GRG, which is an excellent milieu for growth of axons, was placed between the proximal and the distal parts of the transected nerve for reconnection of 2cm long distance.

Results

The post-operative follow-up (up to 4 months) of the operated rats showed re-establishment of active foot movements. The tube had dissolved and the nerve showed complete reconnection. Histological observation of the nerve showed growth of myelinated axons in the place where nerve defect was replaced by composite nerve transplant, and continuation of axonal sprouting through the place of the tube to the distal part of the nerve.

Conclusion

Utilization of an innovative composite implant to bridge a gap resulting from removal of a 2cm peripheral nerve segment shows promise, suggesting the feasibility of this approach for reconstruction of peripheral nerve lesions. Such an implant may serve as a vital bridging station in peripheral nerve injuries with massive loss of tissue.

Ciliary neurotrophic factor induces more extensive collateral sprouting of motor than of afferent axons associated with improved functional reinnervation of the biceps muscle in an experimental model of end-to-side neurorrhaphy

P. Dubový,[1] P. Haninec,[2] O. Raška,[2] L. Stejskal,[2] P. Čelakovský,[2]

[1]Department of Anatomy, Division of Neuroanatomy, Faculty of Medicine, Brno, and [2]Department of Neurosurgery, 3rd Faculty of Medicine, Charles University In Prague, Czech Republic

Introduction

End-to-side neurorrhaphy is based on collateral sprouting of an intact axon. The aim of the present study was to quantitatively assess collateral sprouts sent out by intact motor and sensory axons. End-to-side neurorrhaphy of the distal stump of transected musculocutaneous nerve (MCN) with intact ulnar nerve (UN) was performed in a rat model.

Material and Methods

The experimental model was used to evaluate the efficacy of Cerebrolysin and ciliary neurotrophic factor (CNTF) treatment in promoting the reinnervation of MCN stump by collateral sprouts of intact afferent and motor axons of the UN. CNTF, Cerebrolysin and PBS (control) were

administered intrathecally with a brain infusion cannula connected with an ALZET 2002 osmotic minipump (rate 0.5 µl/h, ALZA, Palo Alto, USA) for 2 weeks, and all animals were left to survive for 2 months from operation. Collateral sprouts of UN axons were quantitatively evaluated by counting spinal motoneurons and DRG neurons following their retrograde labeling by Fluoro-Ruby and Fluoro-Emerald applied to the UN and MCN, respectively. The pool of retrogradely labeled neurons of rats operated on end-to-side neurorrhaphy was detected by distinct red, green and orange (mixed) fluorescence on longitudinal sections through both the spinal cord segments (C6–Th1) and DRG of the same levels. In addition, the functional reinnervation of biceps brachii muscles was evaluated by EMG measurement, a behavioral (grooming) test, and morphological features of regenerated axons (their diameter and myelin sheath thickness).

Results

CNTF treatment resulted in a significantly higher behavioral test score in comparison with PBS- or Cerebrolysin-treated groups of rats. Intrathecal administration with vehicle (PBS) or Cerebrolysin for 2 weeks and survival for 2 months after surgery resulted in a significantly higher number of myelinated axons regenerated into MCN stump in comparison with CNTF treatment. However, the mean diameter of the myelinated axons regenerated into the MCN stump and their myelin sheath thickness of Cerebrolysin- or CNTF-treated animals were larger than for those of rats treated with vehicle (PBS). The mean axon diameter and thickness of myelin sheaths were larger in the group of Cerebrolysin than CNTF-treated rats, but mean scores of the behavioral (grooming) test was significantly the best in CNTF-treated animals.

The percentage of double to all labeled motoneurons was very similar whether following intrathecal administration of PBS or Cerebrolysin, but it was significantly higher after CNTF administration. In contrast, the percentage of double to all labeled DRG neurons was very similar in all experimental groups following intrathecal administration of PBS, Cerebrolysin as well as CNTF. Generally, Cerebrolysin like PBS does not support preferential collateral sprouting from motor or afferent axons in the model of end-to-side anastomosis of the UN and MCN. However, intrathecal application of CNTF supports formation of collateral sprouts much more by motor than afferent axons of the donor UN.

Discussion and Conclusion

A double-labeling method using different fluorescent tracers has been applied to obtain morphological evidence of collateral reinnervation following end-to-side neurorrhaphy (Zhang et al., 1999; Kanje et al., 2000). Morphological evidence of collateral sprouts sent by intact sensory and motor axons of the UN has also been obtained by retrograde labeling of the neurons using one type of molecule (dextran) conjugated with two different fluorophores. This approach to neuronal labeling based on one type of molecule (e.g., Fluoro-Ruby, Fluoro-Emerald) is suitable for quantitative morphological evaluation of collateral sprouting (Kubek et al., 2004; Šámal et al., 2006).

The present experimental study confirms end-to-side neurorrhaphy as a suitable method of nerve reconstruction. Our results revealed that CNTF application results in significantly increased capacity of motoneurons relative to that of DRG neurons to send out collateral sprouts. Simultaneously, CNTF increased the growth of motor axons directly regenerated into the MCN stump that contributed to functional reinnervation associated with better behavioral test results. An injury to a small amount of UN axons during surgical manipulation led to no EMG denervation elements (fibrillations, positive waves) in the flexor carpi ulnaris muscles innervated by the donor nerve.

Acwnowlegments – This work was supported by grants NS10496-3/2009, MSM0021622404 and MSM0021620816.

References

Kanje, M., Arai, T., Lundborg, G., 2000. Collateral sprouting from sensory and motor axons into an end to side attached nerve segment. Neuroreport 11, 2455-2459.

Kubek, T., Kyr, M., Haninec, P., Samal, F., Dubovy, P., 2004. Morphological evidence of collateral sprouting of intact afferent and motor axons of the rat ulnar nerve demonstrated by one type of tracer molecule. Ann. Anat. 186, 231-234.

Samal, F., Haninec, P., Raska, O., Dubovy, P., 2006. Quantitative assessment of the ability of collateral sprouting of the motor and primary sensory neurons after the end-to-side neurorrhaphy of the rat musculocutaneous nerve with the ulnar nerve. Ann. Anat. 188, 337-344.

Zhang, Z.J., Soucacos, P.N., Bo, J.Y., Beris, A.E., 1999. Evaluation of collateral sprouting after end-to-side nerve coaptation using a fluorescent double-labeling technique. Microsurgery 19, 281-286.

Longitudinal retrograde tracer study of sensory axonal ingrowth into end-to-side coapted nerve stump in the rat

T. Žele, U. Kovačič, J. Sketelj, F.F. Bajrović

Institute of Pathophysiology, Faculty of Medicine, University of Ljubljana, Zaloška 4, SI-1000 Ljubljana, Slovenia; tilen.zele@gmail.com

Background

After end-to-side nerve repair the donor nerve axons are able to grow into the recipient nerve stump. It is unclear however; to what extent these ingrowing axons represent either collateral sprouts of uninjured donor nerve axons or regenerating axons of injured donor nerve. As shown by double retrograde labeling studies, the percentage of all sensory neurons projecting to the recipient nerve as well as to the donor nerve was highly variable (10-61%). These studies were performed at different single time points after end-to-side neurorrhaphy. Therefore, it is not clear if the recipient nerve is primarily reinnervated by the regenerating donor nerve axons or by the collateral sprouts of uninjured donor nerve axons which later prune their parent branch from the donor nerve.

Methods

The distal stump of transected peroneal nerve (recipient) was sutured by four epineurial sutures to the side of uninjured ipsilateral sural nerve (donor) in rat. There was no epi/perineurial windowing of the donor nerve at the site of coaptation. At the time of coaptation (control group), 7 days (group I), 28 days (group II), 84 days (group III) or 196 days later (group IV; n=8 for each group), retrograde tracers 1,1'-dioctadecyl-3,3,3',3'-tetramethylindocarbocyanine perchlorate (DiI) and Fluoro Gold (FG) were applied to recipient and donor nerves, respectively, just distally from the coaptation site. After 10 days of recovery, dorsal root ganglia (DRGs) L4-L6 were harvested, cut on freezing microtome, and all single and double retrogradely labeled neurons on sections were counted.

Results

The numbers of all DRG neurons retrogradely labeled from the recipient nerve by DiI were 112 ± 55 (mean ± SD) in the control group, 223 ± 95 in the group I, 332 ± 177 in the group II, 383±138 in the group III and 409±140 in the group IV, respectively. The numbers of all labeled neurons were statistically significantly smaller ($p < 0.05$) in the control group than in groups II, III or IV, respectively. There was no statistically significant difference between groups III and IV in this regard ($p < 0.05$). Double labeled DRG neurons were expressed as the percentages (mean ± SD) of the all neurons labeled from the recipient nerve. The percentages of double labeled neurons were 51% ± 15% in the control group and 61% ± 15%, 31% ± 8%, 24% ± 10% and 24% ± 5% in groups I- IV, respectively. The percentage of double labeled neurons in the control group and group I was statistically significantly larger ($p < 0.05$) than in groups II, III and IV, respectively.

Conclusion

The results of our longitudinal study suggest that early after an end-to-side nerve repair both the collateral sprouting of uninjured and the regeneration of injured donor nerve sensory axons take about equal portion in reinnervation of the recipient nerve stump. Later, however, the number of regenerating axons from the donor nerve or dying back of parental branches of collateral sprouts in the donor nerve increases.

References

Sananpanich, K., Galea, M.P., Morrison, W.A., Messina, A., 2007. Quantitative characterization of regenerating axons after end-to-side and end-to-end coaptation in a rat brachial plexus model: a retrograde tracer study. J. Neurotrauma 24, 864-875.

Sámal, F., Haninec, P., Raska, O., Dubovỳ, P., 2006. Quantitative assessment of the ability of collateral sprouting of the motor and primary sensory neurons after the end-to-side neurorrhaphy of the rat musculocutaneous nerve with the ulnar nerve. Ann. Anat. 188, 337-344.

Bontioti, E., Kanje, M., Lundborg, G., Dahlin, L.B., 2005. End-to-side nerve repair in the upper extremity of rat. J. Peripher. Nerv. Syst. 10, 58-68.

End-to-side nerve repair in brachial plexus and digital nerves: clinical experience

B. Battiston, S. Artiaco, L.G. Conforti, P. Cartesegna, P. Tos
Department of Orthopaedics and Traumatology, CTO Hospital, Turin, Italy

Literature from the last two decades has reported an increasing number of studies on clinical application of end-to-side (ETS) neurorraphy. At present digital nerve lesions and brachial plexus injuries are the most common indications for end-to-side nerve repair.

Regarding digital nerves injuries, our clinical experience and analysis of the literature supports the view that ETS nerve suture can be a reliable technique for recovery of distal sensory reinnervation. During 6 years, we performed ETS nerve coaptation in eight patients with traumatic or postsurgical digital nerve injuries

. All patients had sensory recovery, graded as S3+ in seven cases and S3 in one case, according to the classification of British Medical Research Council modified by Mackinnon and Dellon (1985).

The average two-point discrimination distance was 13 mm. From five retrospective clinical studies reported in the literature a comprehensive number of 26 further patients have been described and in all but one case a successful result has been documented.

As for brachial plexus injury the results reported in the literature are inconstant. We used ETS nerve sutures, associated with standard neurotizations, in 11patients with brachial plexus injuries. In this case series the ETS coaptations showed successful results (M4) in one case, partially successful (M3) in two cases, and poor or absent result (M0-M1-M2) in five cases. We did not observe a significant benefit from ETS neurotization in primary reconstructive surgery for patients with traumatic closed brachial plexus injuries.

Currently, we believe that this technique should not substitute standard neurotizations which are more reliable in adult brachial plexus surgery. Occasionally, ETS coaptation may support standard reconstructive procedures in case of severe brachial plexus injuries when few undamaged donor nerves are available.

References

Battiston B, Artiaco S, Conforti LG, Vasario G, Tos P. End-to-side nerve suture in traumatic injuries of brachial plexus: Review of the literature and personal case series. J Hand Surg Eur Vol, 2009, in press

Artiaco S, Tos P, Conforti LG, Geuna S, Battiston B. Termino-lateral nerve suture in lesions of the digital nerves: clinical experience and literature review. J Hand Surg Eur Vol, 2009 in press.

Tos P, Artiaco S, Papalia I, Marcoccio I, Geuna S, Battiston B. End-to-side nerve regeneration: from the laboratory bench to clinical applications. Int Rev Neurobiol, 2009;87:281-94.

Use of end-to-side anastomosis in treatment of brachial plexus injury

P. Haninec, P. Dubový, L. Mencl, R. Kaiser, L. Houšťava
Department of Neurosurgery, 3rd Faculty of Medicine, Charles University In Prague and Department of Anatomy, Division of Neuroanatomy, Faculty of Medicine, Brno, Czech Republic

In our series of patients the results of the neurotization procedure showed dependence on the type of the donor nerve used. The intraplexal nerves (motor branches of brachial plexus) were significantly (p=0.043) more successful donors of motor fibers (74%) than extraplexal nerves (54%). Functional recovery after the nerve transfer strongly depended also on the individual type of donor and the medial pectoral nerve was the most successful donor (88%). Because of poor functional results of the axillary nerve neurotization via the extraplexal nerves (60%), we used end-to-side neurorrhaphy in 23 cases with incomplete avulsion.

The overall success rate of end-to-side neurorrhaphy was 64, 5% and the success rate for the axillary nerve as a recipient was 62% (follow-up > 2 years). The results of end-to-side neurorrhaphy in neurotization of the axillary nerve were similar to those using intraplexal (67%) or extraplexal (60%) nerves in our group of patients. However, general results of Oberline procedure (donor – ulnar nerve) are nearly 80%. For this reason in present time we are using more Oberline technique than end-to-side anastomosis.

We see the advantage of end-to-side neurorrhaphy over that of "classical" neurotization in that there is no need to sacrifice neither any of the surrounding nerves nor fascicles of the ulnar nerve. Typical synkinesis of muscle contraction innervated by the recipient nerve with contraction of muscles innervated by the donor was observed in patients after the end-to-side neurorrhaphy.

Nerve Repair by Denatured Muscle Autografts Promotes Sustained Sensory Recovery in Leprosy

J.H. Pereira, D.D. Palande, T.S. Narayanakumar, A.S. Subramanian, S. Gschmeissner, M. Wilkinson
James Paget University Hospitals NHS Foundation Trust and University of East Anglia Medical School, Norfolk, England

A total of 38-patients with leprosy and localised nerve damage (11 median at the wrist and 37-posterior tibial at the ankle) were treated by 48 freeze-thawed skeletal muscle autografts ranging between 2.5 cm. and 14 cm. in length. Sensory recovery was noted in 34-patients (89%) and was maintained during a mean period of follow-up of 12.6 years (4 - 14).

After grafting the median nerve in all patients remained free of ulcers and blisters, ten demonstrated perception of texture and eight recognised weighted pins. In the posterior tibial nerve group, 24 of 30 repairs (80%) resulted in improved healing of ulcers and 26 (87%) demonstrated discrimination of texture. Quality of life and hand and foot questionnaires showed high levels of improvement and patient satisfaction.

This study demonstrates that nerve/muscle interposition grafting in leprosy patients results in consistent levels of protective sensory recovery.

References

Millesi H. .Surgery on muscles in consequence of peripheral nerve lesions. Acta Neurochir Suppl. 2007;100:179-81.

Schmidhammer R, Nógrádi A, Szabó A, Redl H, Hausner T, van der Nest DG, Millesi H. Synergistic motor nerve fiber transfer between different nerves through the use of end-to-side coaptation. Exp Neurol. 2009 Jun;217(2):388-94

Nerve repair by fresh muscle-vein-combined nerve guides: Clinical results and actual indications

P. Tos, S. Artiaco, D. Ciclamini, E. Boux, L.G. Conforti, B. Battiston
Department of Orthopaedics and Traumatology, CTO Hospital, Turin, Italy

Introduction: Although autogenous nerve grafting is still considered the best method for bridging nerve defects, several alternative types of conduits (biological and synthetic) have been studied. We have demonstrated in previous experimental research that a graft made using a vein (providing a guide for nerve regeneration) filled with fresh skeletal muscle (to prevent vein collapse and support axon regeneration) gave similar results to traditional nerve grafts, in the rat. On this basis, we decided to use the muscle-vein-combined grafts in clinical cases not only for sensory nerves but also for mixed nerves. Despite continuous researches and surgical innovations, the treatment of peripheral nerve injuries remains a complex problem particularly in non sharp lesions where this kind of reconstruction is a good option of treatment. We report our case series and results.

Matherial and Methods: Mixed nerves: we reviewed 23 patients operated from 1993 to 2004 with this technique. The mean follow up was 26 months (minimum 14 months – maximum 58 months). The mean length of conduits was 2.5 cm (0.5 to 6 cm). Case series: 4 radial nerve at the elbow level, 9 median nerve at the distal third of the forearm, 6 ulnar nerve at the forearm, 1 ulnar nerve at the wrist, 1 ulnar nerve at the arm, 2 proximal cord of the brachial plexus. Sensory nerves: we operated 13 patients for sensory nerve reconstruction at the hand and wrist level.About these patients 13 were operated in emergency for crush injuries of sensory and mixed nerves.

We evaluated our results by the criteria of the Nerve Injuries Committee of the BMRC modified by Mackinnon-Dellon. We classified the results in three groups with the grading system proposed by Sakellarides. Very Good: [illegible] M4 / [illegible] S3+; Good: M3 / S3-S2+; Poor: < M2 / < S2+.

Results: Mixed nerves: In 12 (52%) cases we had a good and very good results. In 6 cases (26%) a good sensory restoration has been not accompanied by a good motor recovery. In 2 cases (8.5%) we had a good motor recovery and a fair sensory recovery. In the last 3 cases (13%), in gap longer than 3 cm, we had a fair results both for sensory and motor recovery.

Sensory nerves : In the muscle-vein-combined group , 10 patients (76.9%) showed Very Good results while only 3 patients (23.1%) showed Good results.

Conclusions: The clinical employment of tubes as an alternative to autogenous nerve grafts is mainly justified by the limited availability of donor tissue for nerve autograft and its related morbidity. Indication, in this little series of patients operated in ten years, had been very restricted :

treatment in emergency, not enough nerve graft, no will of the patient on harvesting a healthy nerve.

Our retrospective study demonstrated that favourable results can be achieved either for primary repair of crush injured nerves when a short gap is present or in secondary procedures mainly for sensory nerves. An attempt of reconstruction in emergency with muscle-vein combined graft or alternative conduits is justified considering the possible advantages offered by this kind of nerve repair.

References

Battiston B, Raimondo S, Tos P, Gaidano V, Audisio C, Scevola A, Perroteau I, Geuna S. Tissue engineering of peripheral nerves. Int Rev Neurobiol, 2009;87:227-49

Battiston B, Tos P, Conforti LG, Geuna S. Alternative techniques for peripheral nerve repair: conduits and end-to-side neurorrhaphy. Acta Neurochir Suppl. 2007;100:43-50.

Battiston B, Geuna S, Ferrero M, Tos P.Nerve repair by means of tubulization: literature review and personal clinical experience comparing biological and synthetic conduits for sensory nerve repair. Microsurgery. 2005;25(4):258-67

Battiston B, Tos P, Cushway TR, Geuna S. Nerve repair by means of vein filled with muscle grafts I. Clinical results. Microsurgery. 2000;20(1):32-6.

Baby sitting procedures in nerve repair: a case of a proximal ulnar lesion

G. Delia, M. Galeano, G. Risitano, F. Stagno d'Alcontres, M.R. Colonna

Dept of Surgical Specialties, Clinical Unit of Plastic Surgery (Head Prof F Stagno d'Alcontres), University of Messina Medical School, Italy

A case of distal reinnervation of proximally transected ulnar nerve in a young man is presented.

In the distal third of the forearm, a small nerve graft was interposed between the median and the ulnar nerve through two end-to-side windows. A three months FU is presented, showing promising results.

This procedure is compared to other baby- sitting techniques.

References

Millesi H. .Surgery on muscles in consequence of peripheral nerve lesions. Acta Neurochir Suppl. 2007;100:179-81.

Schmidhammer R, Nógrádi A, Szabó A, Redl H, Hausner T, van der Nest DG, Millesi H. Synergistic motor nerve fiber transfer between different nerves through the use of end-to-side coaptation. Exp Neurol. 2009 Jun;217(2):388-94

Late nerve repair in obstetrical brachial plexus palsy

F.M. Sénès, N. Catena

UOS di Chirurgia della Mano e Microchirurgia, UOC di Ortopedia e Traumatologia, IRCCS G. Gaslini, Genova, Italy

Microsurgical repair of obstetrical brachial plexus injuries is the method of choice for affected children doomed to an unfavourable motor and sensory outcome. At present, the exact timing for primary nerve surgery is still controversial (from 3 months to 9 months).

According to Gilbert-Tassin indications, the lack of spontaneous recovery of biceps brachialis muscle later than the third month of life is the indicator to proceed to microsurgical revision of injuried brachial plexus and repair by means of nerve grafts or neurotization (intra or extraplexual) on the analogy of the brachial plexus lesions of adulhood.

The choice of which patients should undergo primary nerve surgery is often difficult. In addition to children bound to an unfavorable outcome, identified within the third month of life, there is a group of patients presenting doubtful signs of spontaneous recovery or showing recovery of only some muscles. Therefore, a different surgical approach should be identified for a selective repair of deficient muscular functions.

Undoubtedly, it is mandatory to operate on children affected by total palsy without any sign of recovery, even if they exceed the maximum age established for primary nerve surgery. Selective neurotization of muscular groups can yield interesting results for those patients presenting incomplete recovery of motion during early childhood.

Neurotization of single muscles (ie deltoid, external rotators of the shoulder, triceps, serratus anterior etc.) can be obtained using fascicles or some branches of donor nerves (namely ulnar, accessory spinal, intercostal or thoracodorsal nerves).

The child's good potential for nerve regeneration allows a later use of these procedures compared to the established timing of nerve surgery, with considerable functional recovery.

The Authors illustrate their experience and propose technical indications for late repair of obstetrical brachial plexus injury.

References

Gilbert A, Pivato G, and Kheiralla T. Long term results of primary repair of brachial plexus lesions in children. Microsurgery. 2006;26(4):334-42.

Oberlin C. et al. Nerve transfer to biceps muscle using a part of ulnar nerve for C5-C6 avulsion of the brachial plexus: anatomical study and report of four cases. J Hand Surg Am.1994;19:232-237

Pondaag W et al. External rotation as a result of suprascapular nerve neurotisation in obstetric brachial plexus lesions. Neurosurgery;2005;Sep;57(3):530-537

Kawabata H, Shibata T, Matsui Y, Yasui N. Use of intercostal nerve for neurotization of the musculocutaneous nerve in infants with birth related brachial plexus palsy. J Neurosurgery;2001;Mar;94(3):386-391

Neuroprotective effects of melatonin on peripheral nerve regeneration

M.Turgut

Department of Neurosurgery, Adnan Menderes University School of Medicine, Aydın, Turkey

Despite surgical repair, peripheral nerve damage does not frequently result in complete recovery due to neuroma formation, lipid peroxidative damage, ischemia and other factors. Unfortunately, there is no optimal treatment method enhancing peripheral nerve regeneration. On the other hand, widespread interest has grown among researchers regarding the neuroprotective, free radical scavenging, antioxidative, and analgesic properties of melatonin, the main hormone of the pineal gland in recent years.

Some studies using various experimental injury models clearly suggest various positive effects of melatonin on axon length and sprouting following traumatic events to peripheral nerves. Further experimental and clinical studies are necessary to determine the clinical utility of melatonin hormone. With its possible beneficial effects at macroscopic and microscopic levels, however, it is evident that exogen melatonin administration support peripheral nerve reconstruction and neural plasticity following peripheral nerve injury in clinical practice.

This is a review of the available literature in terms of the effects of melatonin on nerve recovery, especially the sciatic nerve, based on a critical analysis of electrophysiological, biochemical and histopathological findings as well as functional observations.

Patho-morphosis of the upper limb from nervous damage to the light of system theory and principle of regression

G.M. Grippi

SOC di Ortopedia e Traumatologia, Ospedale San Lazzaro di Alba – ASL CN2 Alba-Bra, Italy

Subject of this treatment is the secondary patho-morphosis of the upper limb in the peripheral nervous damage. Or rather, as the development and the trofic-structural (the form) maintenance of the bodily (inclusive upper limb) segments it is known they are influenced for the whole life by efficiency of the nervous system. So the nervous lesions - with the appearance of paralysis and/or muscular contractures - end with to modify its conformation (naturally expressed by the genetic-environmental information) in pathological sense, with real dys-morphism (in general sense).

In dependence from the quality/entity of the damage (to 1° or 2° motoneurones, to the plexus, to the peripheral nerve etc.) and/or from the moment in which this has happened (in born, in the infancy, in the adult, etc.) diversified patho-morphologic orders (f.e. hypoplasia-shortening, adduction or intra-rotation, forearm pronation or supination, flexed or lax elbow-wrist, fingers deviation, atrophies, hand of "monkey", etc.) are verifiable in the clinic. These generally allow the recognition (of dys-morphism) in a neurological context.

In such sense, it is independently amazing to ascertain as from the type of lesion there is often the tendency to a generic isomorphism, that recalls phases of upper limb onto-morphogenesis.

The same likewise, seems to happen in microscopic structure and neuronal physiology and in the metabolism of injured peripheral nerve (s.c. Wallerian degeneration).

The explanation of all of this is to consider within Systems Theory (of Cybernetic matrix) and in the conceptual tool of the s.c. Principle of Regression, according to which " the damage structural curtains to take on a shape as local recalling of onto-morphogenesis ", in adaptative sense.

To support, some cases of common clinical evidence are showed.

References

Bateson G.: Mente e natura (Mind and Nature). Adelphi ed. S.p.A., Mila¬no, 1984.

Bonola A., Caroli A., Celli L.: La Mano – Piccin editore Padova: 3-42, 1981.

Bonner J. T.: Le idee della Biologia. Biblioteca della EST., Milano, 1964.
Brunelli GA: Lesioni nervose dell'arto superiore. Ed. Intern. EDIMES EMS 2004 Pavia
Canepa G., Pelizza A., Pietrogrande V.: Le malattie dello scheletro in età evolutiva – Vol 1, Piccin Nuova Libraria SPA - 1987, Padova
Darwin C.: L'origine delle specie (intr. Montalenti G.), Editore Boringhieri s.p.a., Milano, 1967.
Goethe J.W.: Teoria della natura. Boringhieri, Torino,1968
Grippi G.M.: Medicina e cibernetica: implicazioni metodologiche della Teoria dei Sistemi applicati al piede. Chir. del piede, vol. 7, n. 1, 1983
Grippi G.M.: "Atti del 7° Seminario Albese di Chirurgia del Polso e della Mano" – Alba – 3/07/20 Tipografia l'Artigiana (aut. e reg. Trib. di Alba)
Haeckel E.: Generelle Morphologie der Organismen. Berlino, t. 11, 300, 1866.
Margaria R.: Fisiologia e meccanica del movimento. Biblioteca EST, Milano, 1975.
Miller J. G.: La Teoria Generale dei Sistemi Viventi. Collana Scientifica Fran¬co Angeli, Milano, 1978.
Montalenti A.: Compendio di embriologia. Idelson, Napoli, 1981.
Rossi P.A.: Cibernetica e teoria del¬l'informazione. Editrice La Scuola, Bre¬scia, 1978.
Tanner J.M.: Auxologia: dal feto al¬l'uomo, la crescita fisica dal concepi¬mento alla maturità. Ed. UTET, Torino, 1981.
Von Bertalanffly L.:Teoria generale dei Sistemi. Mondadori Studio, 1983.
Waddington H. C.: Strumenti per pen¬sare, un approccio globale ai sistemi complessi, Biblioteca EST, Milano, 1977.
Wiener N.: Introduzione alla ciber¬netica. Boringhieri, Torino, 1966 (The Human Use of Human Beings, Hugton a Miffin Co., Boston, 1950).

Aging impairs the reinnervation after end-to-side nerve repair in the rat

U. Kovačič, T. Žele, J. Sketelj, F.F. Bajrović

Institute of Pathophysiology, Faculty of Medicine, University of Ljubljana, Zaloška 4, SI-1000 Ljubljana, Slovenia; uros.kovacic@mf.uni-lj.si

Background

Numerous and extensive functional, structural and biochemical changes characterize intact aged peripheral nervous system (Peters, 2002; Verdú et al., 2000). Functional recovery after peripheral nerve injury depends on survival of injured neurons and functional reinnervation of target tissue by regeneration of injured axons and collateral sprouting of uninjured (intact) adjacent axons. The rate of axonal regeneration becomes slower and its extent (density and number of regenerating axons) decreases in aged animals. Aging also impairs terminal sprouting of regenerated axons and collateral sprouting of intact adjacent axons, thus further limiting target reinnervation and its functional recovery (for review see Kovačič et al., 2009a). In particular, the following review will be focused on the impact of aging on sprouting of nociceptive axons and on recovery of cutaneous nociception after end-to-side nerve repair in the rat. Possible mechanisms of impaired reinnervation after end-to-side nerve repair will be discussed.

Methods and Results

In aged rats, collateral sprouting-related expansion of the cutaneous nociceptive territory of the uninjured sural nerve into the adjacent denervated skin, as detected by the pinch test, was slower than in young adult rats, and the absolute reinnervated area after 8 weeks of recovery was about 50% smaller in aged than in young adult rats (Kovačič et al., 2008). In addition, sprouting of sural nerve nociceptive afferents into the end-to-side coapted peripheral nerve was less abundant in aged than in young adult rats (Kovačič et al., 2008; Kovačič et al., 2009b). This conclusion is supported by the hystomorphometric analysis, which showed that the total number of thin myelinated axons in the recipient nerve 8 and 19 weeks after the end-to-side nerve repair was about 6-fold and 7.6-fold, respectively, lower in aged than in young adult rats. Furthermore, functional recovery of cutaneous mechano-nociception after end-to-side nerve repair was about 20% larger in young adult than in aged rats. It was found in 66% and 16% of young and aged rats, respectively (Kovačič et al., 2009b). These age-related effects were similar to those that had been found in functional recovery by collateral axon sprouting after peripheral nerve injury without end-to-side nerve repair.

Decreased survival of aged non-injured and injured neurons, limited intrinsic growth potential of neuron, alteration in its responsiveness to stimulatory or inhibitory environmental factors, and changes in the peripheral neural pathways and target tissues are possible reasons for impaired reinnervation after peripheral nerve injury in old age. We found that age-related differences in the collateral sprouting of nociceptive axons in rats is mostly due to the age-related changes in the peripheral neural pathways and target tissues, and not due to the limited intrinsic growth capacity of neurons or their reduced responsiveness to trophic factors (Kovačič et al., 2008). Namely, cross-transplantations of the end-to-side coapted nerve grafts between young and aged rats demonstrated that average number of myelinated sensory axons in the nerve grafts from young rats coapted to the side of the sural nerves of aged rats (host) was a) 2.4-fold higher than that in the aged nerve grafts coapted to the side of the young host sural nerves, and b) not statistically significantly smaller than that in the end-to-side coapted grafts in the control group of young rats (Kovačič et al., 2008). The levels of laminin polypeptides, as detected by Western blot analysis, in peripheral nerves were 50-100% higher in young adult than

in aged rats. However, the levels of peripherin, NGF isoforms and TrkA in skin, peripheral nerves and DRG, respectively, were not significantly reduced in aged rats. Therefore, impaired sprouting of nociceptive axons in aged rats is due rather to the alterations in peripheral neural pathways (i.e. decreased levels of extracellular matrix components), than to the limited sprouting capacity of aged sensory neurons.

Conclusion

Axon sprouting from the spared sural nerve, both into adjacent denervated skin and into end-to-side coapted nerve graft, was significantly higher in young rats than in aged rats. In addition, axon sprouting from young recipient sural nerves into aged donor nerve grafts was significantly deteriorated, whereas the axon sprouting from aged recipient sural nerves into young donor nerve grafts was not statistically significantly affected. Age-related alterations in the non-soluble extra-cellular matrix components of neural pathways, like laminin, might be important in this respect (see also Gavazzi and Cowen, 1993; Gavazzi et al., 1995).

References

Gavazzi, I., Cowen, T., 1993. Axonal regeneration from transplanted sympathetic ganglia is not impaired by age. Exp. Neurol. 122, 57-64.
Gavazzi, I., Boyle, K.S., Edgar, D., Cowen, T., 1995. Reduced laminin immunoreactivity in the blood vessel wall of ageing rats corelates with reduced innervation in vivo and following transplantation. Cell Tissue Res. 281, 23-32.
Kovačič, U., Sketelj, J., Bajrović, F.F., 2009a. Chapter 26: Age-related differences in the reinnervation after peripheral nerve injury. Int. Rev. Neurobiol. 87, 465-482.
Kovačič, U., Sketelj, J., Bajrović, F.F., 2009b. Effect of aging on recovery of cutaneous nociception after end-to-side nerve repair in the rat. Ann. Plast Surg. 62, 439-445.
Kovačič, U., Žele, T., Marš, T., Sketelj, J., Bajrović F.F., 2008. Aging impairs collateral sprouting of nociceptive axons in the rat. Neurobiol. Aging doi:10.1016/j.neurobiolaging.2008.03.020.
Peters, A., 2002. The effects of normal aging on myelin and nerve fibers: a review. J. Neurocytol. 31, 581-593.
Verdú, E., Ceballos, D., Vilches, J.J., Navarro, X., 2000. Influence of aging on peripheral nerve function and regeneration. J. Peripher. Nerv. Syst. 5, 191-208.

Tissue engineering of peripheral nerves: The need for an interdisciplinary approach

S. Raimondo,[1] G. Ronchi,[1] P. Tos,[2] S. Geuna,[1] M. Fornaro,[1] G. Gambarotta,[3] I. Perroteau,[3] M.G. Giacobini-Robecchi,[1] B. Battiston[2]

[1]Department of Clinical and Biological Sciences, University of Torino, San Luigi Gonzaga Hospital, Orbassano (TO); [2]Reconstructive Microsurgery Division, C.T.O. Hospital, Torino; [3]Department of Animal and Human Biology, University of Torino, Italy

Tissue engineering of damaged peripheral nerve is a challenging branch of regenerative medicine with a very high potential clinical impact.

The investigation of peripheral nerve techniques of repair by tissue engineering has seeing an increasing interest from both clinical and basic researchers (Battiston et al. 2009, de Ruiter et al. 2009). To optimize the strategy for the tissue engineering of the peripheral nerves in the clinical view, researchers have to strive for a new level of innovation which will bring together (in a multi-translational approach) the main pillars of tissue engineering, namely 1) Microsurgery (Tos et al. 2009), 2) Transplantation (organs, tissues, cells, genes)(Radtke et al. 2009), 3) Material science (Chiono et al. 2009), 4) Physical therapy (Rochkind et al. 2009).

In order to obtain good results it's necessary an integrated and translational overview on these four key approaches to peripheral nerve tissue engineering.

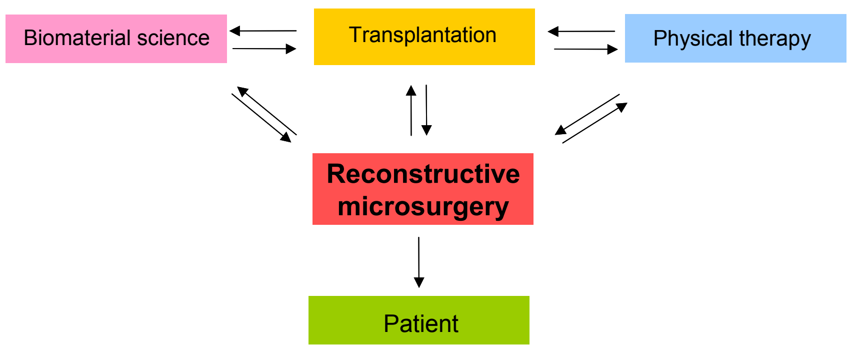

In this presentation we will provide a critical overview of the future perspective in combining the different approaches of tissue engineering focusing not only on the promises and expectations but also on the possible pitfalls, which may arise for complex biotechnological interventions.

Acwnowlegments – Financial support: MIUR and Regione Piemonte

References

Battiston B, Raimondo S, Tos P, Gaidano V, Audisio C, Scevola A, Perroteau I, Geuna S. Tissue engineering of peripheral nerves. Int Rev Neurobiol. 2009; 87:227-49.

Chiono V, Tonda-Turo C, Ciardelli G. Artificial scaffolds for peripheral nerve reconstruction. Int Rev Neurobiol. 2009; 87:173-98.

de Ruiter GC, Malessy MJ, Yaszemski MJ, Windebank AJ, Spinner RJ. Designing ideal conduits for peripheral nerve repair. Neurosurg Focus. 2009; 26:E5.

Radtke C, Kocsis JD, Vogt PM. Transplantation of olfactory ensheathing cells for peripheral nerve regeneration. Int Rev Neurobiol. 2009; 87:405-15.

Rochkind S, Geuna S, Shainberg A. Phototherapy in peripheral nerve injury: effects on muscle preservation and nerve regeneration. Int Rev Neurobiol. 2009;87:445-64.

Tos P, Artiaco S, Papalia I, Marcoccio I, Geuna S, Battiston B. End-to-side nerve regeneration: from the laboratory bench to clinical applications. Int Rev Neurobiol. 2009; 87:281-94.

Bridging laboratory data to clinical applications for promoting peripheral nerve regeneration: Pitfalls and perspectives

S. Geuna,[1] P. Tos,[2] I. Papalia,[3] I. Perroteau,[4] B. Battiston[2]

[1]Department of Clinical and Biological Sciences, University of Torino, San Luigi Gonzaga Hospital, Orbassano (TO); [2]Traumatology Division, C.T.O. Hospital, Torino; [3]Department of Department of Surgical Disciplines, University of Messina; [4]Department of Animal and Human Biology, University of Torino, Italy

Usually experimental nerve regeneration studies are carried out on “ideal” subjects, i.e. young and healthy animals. This might represent a problem when researchers seek to translate experimental results to the clinics since patients often do not match these two characteristics (i.e. they are not young and/or concurring diseases are present). Since this discrepancy is likely to represent one of the causes of the failure in translating laboratory bench results to the patient bed, the employment experimental models with old animals and/or concurring diseases (such as infections, diabetes, etc.) should be also taken into consideration to verify the effectiveness of a new technique for improving nerve regeneration.

A further factor which might cause differences between experimental studies and clinical applications is represented by delayed nerve regeneration since it has been shown a delay in surgical nerve repair results in impaired nerve regeneration and functional recovery both in rodents and humans (Richardson, 1997, Saito and Dahlin, 2008).

Finally, sexual dimorphism should also taken into consideration since there is evidence that nerve regeneration is more pronounced in females because of the neuroprotective effects of sex hormones.

In this presentation, we will provide an overview on these nd other critical issues in translational nerve research.

References

Battiston B, Raimondo S, Tos P, Gaidano V, Audisio C, Scevola A, Perroteau I, Geuna S. Tissue engineering of peripheral nerves. Int Rev Neurobiol. 2009; 87:227-49.

In vitro comparison of motor and sensory neurons outgrowth in a 3D collagen type I matrix

I. Allodi, M.S. Guzman-Lenis, X. Navarro, E. Udina

Group of Neuroplasticity and Regeneration, Faculty of Medicine, Universitat Autonoma Barcelona, and CIBERNED, Bellaterra, Spain

Specificity of nerve regeneration, in terms of motor and sensory fibers reinnervation of appropriate targets, is still poorly understood. An increased amount of regenerating nerve fibers does not always mean enhanced functional recovery, so that after a peripheral nerve injury, patients are still not able to reach again the normal motor control and sensibility. For that reason, it is important to understand deeper the molecular mechanisms implicated in axonal growth and target reinnervation after injury, taking into account the complex interactions between neurotrophic factors, extracellular matrix molecules and their receptors. These important cues are involved in cells

chemotaxis, migration and nerve elongation during development, and their signalling pathways can promote cell survival or apoptosis. Nowadays, it is becoming clear that these molecular cues pathways maintain important connections between each other.

The aim of this work is to set up an in vitro model for the comparative investigation of motor and sensory neurons outgrowth and to apply this knowledge as a tool to reduce random reinnervation of target organs. We modified two well known in vitro methods: the organotypic spinal cord slice and the dorsal root ganglia (DRG) explant. The tissues were cultured for a few days at 37° C in a three dimensional matrix of collagen type I. Collagen gels can be easily manipulated maintaining the same concentration, density and pH, and allow to add molecules to assess changes in neurites outgrowth related to the modified environment. Finally, a collagen matrix can also be used to fill artificial neural guides used for nerve repair in vivo, so that our findings in vitro could be easily translated to in vivo model.

Samples of spinal cord and DRG were obtained from postnatal day 7 rats, and freed from meningeal layers under dissecting microscope in sterile conditions. Spinal cord segments were cut with a McIlwain chopper at 350 µm, whereas whole DRGs were used. Both tissues were embedded within a collagen type I solution gelified at 3 mg/ml (BD Biosciences), and maintained at 37ºC under CO_2 atmosphere in a culture cabine.

By immunolabeling motoneurons and myelinated sensory neurons with neurofilament antibody (RT97), we observed that the 3D matrix allowed both types of neurites to grow outside the explant after a few days in vitro. Thus, the collagen matrix creates a permissive environment for regeneration. Both sensory and motor neurons elongated their neurites into the matrix, but the pattern of regeneration was different depending on the tissue: motor neurites followed the remaining parts of spinal roots attached to the spinal cord slices and, at the same time S100-positive glial cells migrated outside the slice, following also the roots and around the new neurites growing out. In the DRG explants S100 positive cells were also supporting neurites outgrowth, but, in most cases, the outgrowth had a 360°-like pattern, all around the explant. Sensory DRG neurons appear to have a higher intrinsic capacity of outgrowth compared with spinal motorneurons but we should also keep in mind the constitutive differences between the explants.

We changed the environmental conditions by adding different neurotrophic factors to the matrix and comparing the results with control conditions of both assays. We added GDNF (glial cell line-derived neurotrophic factor), BDNF (brain derived neurotrophic factor) and NGF (nerve growth factor) to assess the reliability of the in vitro models to study basic insight of axonal regeneration and to screen the specificity of different factors for motor or sensory nerve growth.

We first compared the effects of different trophic factors on neuronal survival and regeneration. Both BDNF and GDNF enhanced motorneurons outgrowth, while NGF treatment did not improve growth when compared to control spinal cord slices. On the other hand, the three neurotrophic factors significantly improved neurite outgrowth in DRG explants.

In conclusion, the in vitro organotypic culture models used allow a reliable comparison under the same conditions of the effects of different molecules with potentially specific capacity to promote outgrowth of sensory or motor neurons, and also to manipulate the environement (for example, by adding other neurotropic guidance cues or other trophic factors at the collagen matrix). Further studies are needed to search for the interactions of axonal tips with extracellular factors, and to find new strategies to improve specific reinnervation of target organs.

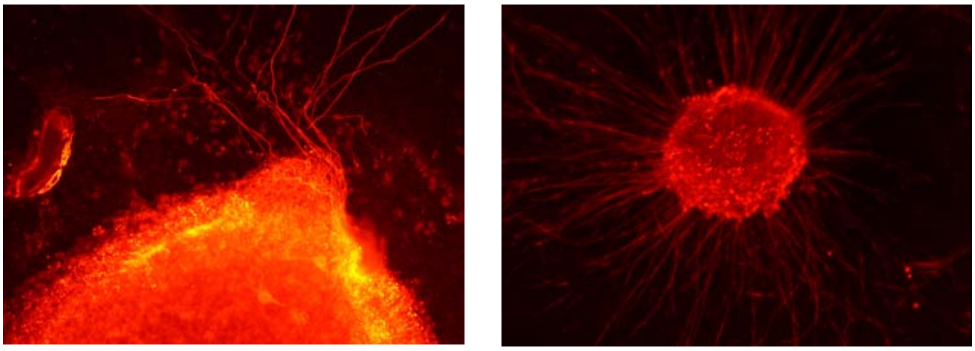

Figure 1. Outgrowth of motor and sensory neurons after in vitro culture in a 3D collagen matrix (control conditions); neurons and neurites are labelled with RT97 antibody

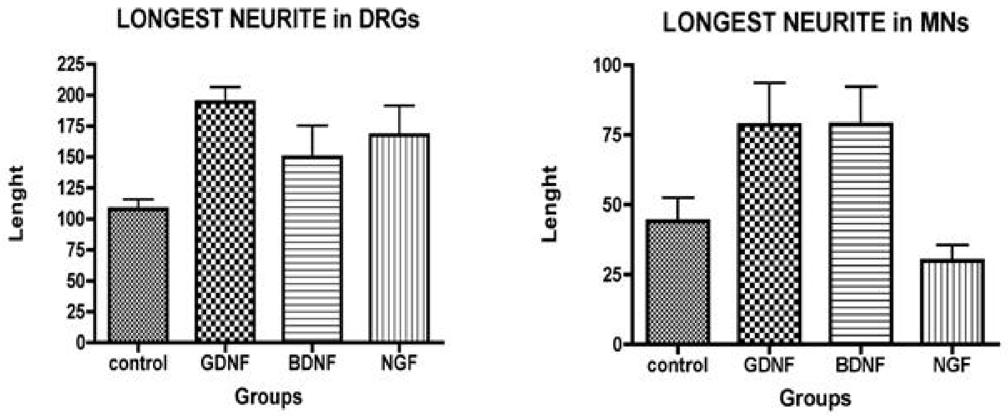

Figure 2. Measurements of the longest neurites in DRGs explants after 2 days in vitro and in spinal cord slices after 4 days in vitro, in different environmental conditions

References

Gahwiler BH, Capogna M, Debanne D, McKinney RA, Thompson SM (1997) Organotypic slice cultures: a technique has come of age. TINS 20(10):471-477

Guzman-Lenis MS, Navarro X, Casas C (2009a) Drug screening of neuroprotective agents on an organotypic-based model of spinal cord excitotoxic damage. Restor Neurol Neurosci 27:335-349.

Hoke A, Redett R, Hameed H, Jari R, Zhou C, Li ZB, Griffin JW, Brushart TM (2006) Schwann cells express motor and sensory phenotypes that regulate axon regeneration. J Neurosci 26:9646-9655.

Ide C (1996) Peripheral nerve regeneration. Neurosci Res 25:101-121.

Labrador RO, Butí M, Navarro X (1998) Influence of collagen and laminin gels concentration on nerve regeneration after resection and tube repair. Exp Neurol 149: 243-52

Tucker A, Lumsden A, Guthrie S (1996) Cranial motor axons respond differently to the floor plate and sensory ganglia in collagen gel co-cultures. Eur J Neurosci 8:906-916

Nerve decompression does not allow sciatic function reestablishment after chronic constriction injury in the rat

S. Amado,[1] A. Mazzuco,[2] A.C. Maurício,[3] A. Veloso,[1] S. Raimondo,[2] S. Geuna,[2] P. Armada-da-Silva[1]

[1]CIPER/FMH, Technical University of Lisbon, Portugal, [2]Dipartimento di Scienze Cliniche e Biologiche, Universitá di Torino, Italy, [3]UMIB/ICBAS University of Oport;, parmada@fmh.utl.pt

Chronic constriction injury (CCI) of the rat sciatic nerve is considered a suitable experimental model of neurophatic pain with injured animals developing mechanical allodynia and thermal hyperalgesia after a few day of the lesion. The injury is described as causing marked nerve damage mainly but not exclusively to large diameter axons, and incomplete denervation of the peripheral field. Only a few attempts have been made to study recovery from this type of injury by surgical removal of constriction. The aim of the present study was to assess the severity of motor and sensory loss in CCI and its recover with nerve decompression. Methods: Twenty male Sasco Sprague-Dawley rats (250-300g) were separated in an unoperated control group and three groups with CCI with one of them using resorbable suture. In one CCI group, the non-resorbable ligatures were removed after 4 weeks. Animals were sacrificed after 8 weeks. CCI was performed in deep anesthetized animals after exposing and encircling the right sciatic nerve with 4 loose ligatures using 0/4 thread. Motor deficit was assessed by the extensor postural thrust (EPT) and expressed as percentage of the contralateral side. Thermal algesia was evaluated by the withdrawal reflex latency (WRL) measured by the time taken by the animal to remove its paw from a hotplate. The injured sciatic nerves were removed and kept for histomorphometry of nerve regeneration. Results: Pre-operatively the EPT scores were similar in both sides and in all groups but quickly dropped to near null values at week 1 post constriction and at week 8 percentage motor deficit in CCI groups was between 92 and 93% contrasting to 0% in the control group. No differenced existed for motor deficit between the three sciatic nerve constricted animals. All animals displayed normal WRL response pre-operatively but at week 1 all constricted animals reach the cutoff time of 12 sec. Unlike EPT, there was recovery of WRL scores with mean values in the CCI groups at week 8 between 4.0 ± 1.1 sec and 2 ± 0.0 sec, comparable to values in the control group (2.0 ± 0.0 sec). No statistical differences were observed between the CCI groups. At levels below the

constriction point, gross nerve morphological changes were noticed in all groups, large decrease in axon and myelin sheet thickness. In some deconstruction-group animals, signs of nerve regeneration and a more close to normal nerve morphology could be observed. In the animals sustaining constriction up to 8 weeks, nerve fibres were closely packed with decrease in interstitial space. A large number of cells were seen intermingled with nerve fibers, suggesting ongoing inflammatory/proliferative activity. Discussion: Sciatic nerve chronic constriction causes swelling and inflammation at constriction site and axon Wallerian degeneration. Axonal damage has been reported to be more severe in large myelinated motor axons [1] and in this study we confirm that CCI injury produces severe loss of muscle function. Thermal sensation was also deeply affected within one week of injury but unlike motor function it recovered to normality within 8 weeks. Surprisingly, nerve decompression had no beneficial effect on motor or sensory function which is in contrast to a previous study showing a positive effect of nerve decompression on allodynia and molecular markers of inflammatory response at the dorsal horn of the spinal cord [2]. Histological changes in the constricted sciatic nerves will enable us to at least partly explain the present functional data.

Acknowledgments – This study was supported by grant PTDC/CVT/64220/2006 from Fundação para a Ciência e a Tecnologia, Ministério para a Ciência e o Ensino Superior.

References

1. Daemen, M.A., H.A. Kurvers, P.H. Bullens, D.W. Slaaf, G. Freling, P.J. Kitslaar, and F.A. van den Wildenberg, Neurosci Lett, 1998. 247(2-3): p. 204-8.
2. Tseng, T.J., C.C. Chen, Y.L. Hsieh, and S.T. Hsieh, Neuroscience, 2008. 156(3): p. 758-68.

Effects of mesenchymal stem cells on peripheral nerve repair

B. Ayas,[1] P. Gürgör,[1] Z. Erişgin,[1] A. Korkmaz,[1] M. Çifci[2]

[1]Ondokuz Mayıs University School of Medicine, Histology and Embryology Samsun Turkey, [2]Plastic, Reconstructive and Aesthetic Surgery Samsun, Turkey; ayasb@omu.edu.tr

Introduction

Bone marrow stromal cells (BMSC), a form of multipotential mesenchymal stem cells (MSC), are capable of differentiating into several types of cells under appropriate conditions. Schwann cells, which play a key role during myelinization as well as peripheral nerve regeneration, can also be formed in the same way (1). The interaction of these cells with the growing axons is a crucial issue during peripheral nerve regeneration. The BMSC have been utilized in a variety of peripheral nerve injury experiments (2,3) with the hope of helping this process. These studies, where different methodologies have been applied, show that Schwann cells derived from BMSC have great potential on promoting regeneration of peripheral nerves. In the present study we have used the BMSC by employing a recently used (4) reconstruction approach that is said to be more efficient, namely the end-in-end nerve repair method. Restriction of neurotrophic factors in the repair area, keeping out inflammmatory mediators and minimizing neuroma formation are some of its benefits.

Methods

Five male adult white New Zealand rabbits were used, weighing 2500 – 3000 g. After exposing the sciatic nerves, a 5 mm long part was transversely dissected out on either side. On both sides, the epineurium of the proximal stump has then been sutured around the relevant distal stump according to the aforementioned method. Formerly harvested and cultured autologous MSC were injected into the gap on the right side only. The sutured nerve on the other side was left untreated and received no cell injection. Four weeks later, repair areas were removed and processed for routine histology. Paraplast blocks were cut with a nominal section thickness of 6 μm, mounted on polylysine coated slides and stained with the S100 antibody to demonstrate Schwann cells. The repair areas were cut exhaustively and sampled in a systematic random fashion, which yielded a total of about 10 sections. Stained areas were considered to be Schwann cells, whether existing or newly derived. Evaluations of the left and right sides were made by comparing their Schwann cell density determined by estimating their volume fractions (5).

Conclusions

The repair areas on both sides showed no significant differences as to the degree of immunoreactivity for S100. But the volume fraction of the Schwann cells within the repair area was estimated to be increased on the cell-treated nerves. This result supports the findings already reported in the literature, in that the increased Schwann cell density will probably promote regeneration of the peripheral nerve. We are planning to expand this issue by adding further age groups to study and using more markers like GFAP and CD 31

Acknowledgments – Supported by the Project Management Office of the Ondokuz Mayıs University (Project No. T573).

References

1. Ishikawa N et. al., 2009. Peripheral nerve regeneration by transplantation of BMSC-derived Schwann cells as chitosan gel

sponge scaffolds Schwann cells as chitosan gel sponge scaffolds. Journal of Biomedical Materials Research
2. Tohill M. et al., 2004. Rat bone marrow mesenchymal stem cells express glial markers andstimulate nerve regeneration. Neuroscience Letters. 362, 200–203.
3. Dezawa M., 2002. Central and peripheral nerve regeneration by transplantation of Schwann cells and transdifferentiated bone marrow stromal cells. Anatomical Science International. 77, 12–25.
4. Siemionow M. et al. 2002. Epineural sleeve neurorrhaphy: surgical technique and functional results--a preliminary report.. Ann Plast Surg.. 48(3), 281-5
5. Howard C.V. and Reed M.G., 1998. Unbiased stereology; Three dimensional measurement in microscopy., BIOS Scientific Publishers.

Morphological and functional modifications of the nervous system in a rat model of oxaliplatin-dependent neuropathy

L. Bonaccini,[1] L. Di Cesare Mannelli,[2] C. Ghelardini,[2] A. Pacini[1]

[1]*University of Florence, Dept. of Anatomy Hystology and Forensic Medicine, Viale Morgagni 85, 50139, Florence,* [2]*University of Florence, Dept. of Preclinical and Clinical Pharmacology, Viale Pieraccini 6, 50139, Florence, Italy.*

Oxaliplatin, a platinum-based chemotherapeutic agent, has become a standard treatment for advanced colorectal cancer [Andre et al., 2004] and a valid option for patients in the adjuvant setting [Petrioli et al., 2008]. Unlike other platinum derivatives, oxaliplatin does not result in significant renal impairment or ototoxicity, and it has only mild hematological and gastrointestinal toxicity, while neurotoxicity is the limiting side effect. Oxaliplatin's dose-limiting toxicity is related to the development of peripheral neuropathy. Typically, the clinical presentation reflects an axonal peripheral neuropathy with glove-and-stocking distribution sensory loss, combined with features suggestive of nerve hyperexcitability including paresthesia, dysesthesia, and pain. These symptoms may be disabling, adversely affecting activities of daily living and thereby quality of life [Kieman and Krishnan, 2006].

In order to delucidate the morphological and molecular alterations that occur in the peripheral and central nervous systems during neuropathy, we daily injected rats with clinically relevant doses of oxaliplatin (2,4 mgkg-1 intraperitoneally). Twenty-one days later, the behavioural paw-pressure test evidenced a significantly reduced pain threshold consisting in mechanical hyperalgesia. This behavioural aspect was accompanied by no evident morphological and morphometric alterations in the peripheral nerves: the histochemical study did not highlight neither oedema or inflammatory infiltrate nor variations in myelin thickness or axonal diameter. On the contrary, the oxaliplatin treatment increased the incidence of eccentric nucleoli and multinucleolated neurons in the lumbar dorsal root ganglia (DRG). Moreover, a significant reduction in the somatic area of small (<600μm2) and medium (600-1200μm2) neurons between controls and the oxaliplatin groups was observed.

The immunohistochemical analysis showed an oxaliplatin-induced alteration of gene expression profile. In the peripheral nervous system the expression of the cellular injury marker ATF3 (activating transcription factor 3) was enhanced both in axons and in myelinating Schwann cells of the sciatic nerve as well as in neurons and satellite cells of DRG. The expression of the structural protein NF200 (200 kDa neurofilament) was unaltered is the nerve, whereas the majority of DRG neurons displayed a dramatic decrease.

In the central nervous system a general glia activation was provoked by oxaliplatin administration. Both in the spinal cord and in pain-related cerebral areas, treated rats displayed a microglial activation, consisting in a round-shaped body and very few extremely short processes. Moreover, the astrocytes showed an increased density and signs of activation, such as increase in the body size, thickening of the processes and labelling intensity.

An understanding of the factors involved in the development and maintenance of neuropathy and the study of the neuron vs glia signalling may lead to mechanism based therapies that prevent/treat the neuropathic pain face and improve neurorestoration.

References

T. Andre, C. Boni, L. Mounedji-Boudiaf, Oxaliplatin, fluorouracil, and leucovorin as adjuvant treatment for colon cancer; Multicenter International Study of Oxaliplatin/5-Fluorouracil/Leucovorin in the Adjuvant Treatment of Colon Cancer (MOSAIC) Investigators, J. Clin. Oncol. 15 (2004), pp. 229-237.

M.C. Kiernan and A.V. Krishnan, The pathophysiology of oxaliplatin-induced neurotoxicity, Curr. Med. Chem. 13 (2006), pp. 2901-2907.

R. Petrioli, A. Pascucci, E. Francini, Neurotoxicity of FOLFOX-4 as adjuvant treatment for patients with colon and gastric cancer: A randomized study of two different schedules of oxaliplatin, Cancer Chemoth. Pharm. 61 (2008), pp. 105-111.

Cyanoacrylate glue versus microsuturation in peripheral nerve anastomosis using histomorphometric and tensiometric analysis

Y.A.D. Burhanoglu,[1] K.A. Sargin,[2] B. Can,[3] O. Evirgen,[4] I. Karabulut,[5] M.N. Koc,[6] D. Balkanci[7]

[1]*Department of Plastic and Reconstructive Surgery, Ankara Numune Training and Research Hospital, Ankara, asudeniz@gmail.com;* [2]*Infertility Clinic, Suleymaniye Maternity and Women Health Research and Training Hospital, Istanbul, drayse1980@yahoo.com;* [3]*Department of Histology and Embryology, Ankara University School of Medicine, Ankara, belgincan@yahoo.com;* [4]*Department of Histology and Embryology, Ankara University School of Medicine, Ankara, oya.evirgen@gmail.com;* [5]*Department of Physiology, Hacettepe University School of Medicine, Ankara, ikbulut2001@yahoo.com;* [6]*Department of Plastic and Reconstructive Surgery, Ankara Numune Training and Research Hospital, Ankara, mnihat@yahoo.com;* [7]*Department of Physiology, Hacettepe University School of Medicine, Ankara, Turkey, zbalkanc@hacettepe.edu.tr*

Nerve injuries and repair are still controversial and functional recovery after severe nerve injuries are often poor, resulting in permanent disabilities. Although the ideal technique is yet to be developed, several principles for nerve repair have been set (Nishihira et al., 1989). These principles include: 1) The repair should limit fibrosis and inflammation, which may interfere with axon sprouting and produce misdirection of the nerve fibers; 2) accurate anatomic approximation of the nerve stumps should be achieved without tension or gapping to permit regenerating axons to cross the anastomosis; and 3) the orientation of the fibers should be maintained until regeneration is complete. Different techniques have been used to repair the nerves such as; laser, fibrin glue, suture (Tomas et al, 2001; Wieken et al., 2003), grafting (allograft, autograft) (Auba et al., 2006), tubulation (Spector et al., 1997). A major focus of nerve repair research has been the development of procedures with which to avoid or minimize the use of sutures and prevent fibrous ingrowths at the repair site (Tomas et al., 2001; Wieken et al., 2003).

The aim of this study was to evaluate the benefits and functional outcome of repairing nerves with cyanoacrylate adhesive. The tensinometric and histomorphometrical analysis after repair of lesions to the sciatic nerve was studied in 32 adult Wistar Albino rats to compare the conventional microsuturing with cyanoacrylate adhesive. The right sciatic nerve of 32 female, Wistar rats was sectioned and repaired. The rats were randomized into 2 experimental groups: Cyanoacrylate group (n 16) and suture group (n 16). In the control group the sciatic nerve of the left side was exposed but was not sectioned. The sciatic nerve was transected and immediately after transection readapted with cyanoacrylate in the cyanocrylate group and 9/0 nylon sutures in the suture group. The rats were sacrificed and sciatic nerves were harvested bilaterally at 5, 7, 9, 12, 15, 21, 50, 90 days postanastomosis. For each day, two of the sciatic nerves were analyzed tensinometrically for the tensile strength with a device named Biopac MP 30 and the rest of them were examined under light microscope by counting the number of axons in the proximal and distal nerve segments.

The repair sites in cyanoacrylate glue group were more hypertrophic than those repaired with microsutures. Increase in the epineural thickness and neural constriction occured in cyanoacrylate group (fig 1a, 1b, 1c). The degree of adhesion at the anastomosis was greater in cyanoacrylate group. There was evidence of reduction in the axons' number, existence of fine fibrosis, Wallerian degeneration, and regenerative changes in the distal segments of the nerves of cyanoacrylate group. Suture group showed a minimal foreign body reaction and fairly good axonal regeneration. Both repaired groups had smaller axonal diameters, an increase in fiber density, and in total number of myelinated axons compared with that of control nerves (fig 2 a, 2b, 2c).

The histomorphometric assessment showed statistically superior results in suture group than cyanoacrylate group in the mean diameter and the number of regenerated myelinated axons distal to the repair site for all time points ($p<0,05$). After the 21st day there was statistically significant difference between cyanoacrylate and control groups ($p<0,05$) but not in between groups B and C ($p>0,05$). Cyanoacrylate has the poorest results for all time points.

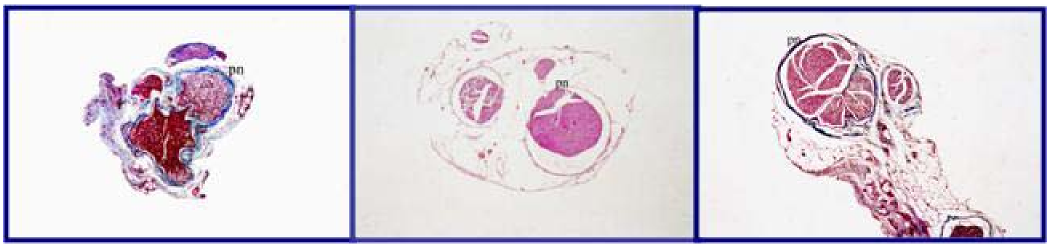

Figure 1a, b, c: Although neural integrity and epineurium continuity was not corrupted in a (control group) b (microsuture group) and c (cyanoacrylate group) at x12,5 zoom, increase in the epineural thickness and neural constriction were observed in c (x12.5, a: Hematoxylen – Eosin Staining, b,c: Mallory Azan Staining)

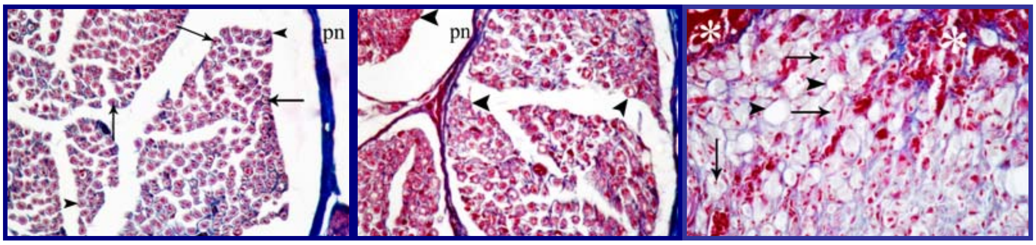

Figure 2a, b, c: Cross sections taken from the distal of sciatic nerve repair line are shown. a: 5th day control group; b: 5th day microsuture group and c: 5th day cyanoacrylate group are monitored on images at x40 zoom for myelinated axon counting.(x100 Hematoxylen – Eosin Staining, b, c: Mallory Azan Staining)

The tensiometric analysis showed statistically significant difference between control group and cyanoacrylate, suture groups (p<0,05) until the day 21. After the day 21 there was no statistically significant difference between all groups (p>0,05). The values for the study groups reached that of the the control group.

In conclusion anastomosis of nerve with cyanoacrylate adhesive does not improve morphological and functional recovery compared to that of conventional epineural sutures, contrary to the recent publications.

References

Auba, C., Hontanilla, B., Arcocha, J., Gorria, O. Peripheral Nerve Regeneration Through Allografts Compared with Autografts in Fk506-Treated Monkeys. J. Neurosurg. 105: 602, 2006.

Hamm, K.D., Steube, D., Pothe, H., et al. Experimental Studies In Animals On The Use Of A Fibrin Glue From The Human Plasma Fraction Cohn I In Nerve Reconstruction. Folia Haematol. Int. Mag. Klin. Morphol. Blutforsch. 115: 208, 1988.

Marshall, D.M., Grosser, M., Stephanides, M.C., et al. Sutureless Nerve Repair At The Fascicular Level Using A Nerve Coupler. J. Rehabil. Res. Dev. 26: 63, 1989.

Nishihira, S. and McCaffrey, T.V. Repair of Motor Nerve Defects: Comparison of Suture and Fibrin Adhesive Techniques. Otolaryngol. Head Neck Surg. 100: 17, 1989.

Spector, J.G. Neural Repair in Facial Paralysis: Clinical and Experimental Studies. Eur. Arch. Otorhinolaryngol. 254 (Suppl. 1): 68, 1997.

Tomas, M. and Johan, F. B. Laser, Fibrin Glue, or Suture Repair of Peripheral Nerves: A Comparative Functional, Histological, And Morphometric Study in the Rat Sciatic Nerve. J. Neurosurg. 95: 694, 2001.

Wieken, K., Angioi-Duprez, K., Lim, A., Marchal, L., Merle, M. Nerve Anastomosis with Glue: Comparative Histologic Study of Fibrin and Cyanoacrylate Glue. J. Reconstr. Microsurg. 19: 17, 2003.

Layer-by-layer coating with photoactive copolymers in biomedical applications

I. Carmagnola,[1] V. Chiono,[1] C. Tonda-Turo,[1] P. Gentile,[1] F. Boccafoschi,[1] G. Georgiev,[2] V. Georgieva,[2] G. Ciardelli[1]

[1]Department of Mechanics, Politecnico di Torino, Corso Duca Degli Abruzzi 24, 10129 Torino, Italy, e-mail: irene.carmagnola@polito.it; gianluca.ciardelli@polito.it; [2]Laboratory of Water-Soluble Polymers, Polyelectrolytes and Biopolymers, Faculty of Chemistry, University of Sofia, 1 J. Bourchier Ave., 1164 Sofia, Bulgaria, e-mail: Georgs@chem.uni-sofia.bg

Introduction

Biomaterial surface plays an important role in biological system due to the biological reactions occurring at material/biological environment interface. The improvement in biocompatibility of biomaterials for tissue engineering by directed surface modification at nanoscale is an important contribution to biomaterials development [1]. Two techniques dominated the research in this area: Langmuir Blodgett deposition and self-assembled monolayers (SAMs). An alternative of Langmuir Blodgett deposition and SAMs is layer-by-layer (LbL) assembly, introduced by Decher and Hong for preparing structured controlled thin films for biological applications [2]. The LbL process, driven by electrostatic interactions, involves sequentially dipping of charged substrate into solution of oppositely charged polyelectrolytes and allowing the polymer to absorb and reverse the charge of the substrate surface. A rinsing step is included between one adsorption process and another, to remove excess and to prevent cross-contamination of the polyelectrolyte solutions.

The LbL technique is versatile and offers the benefit of solvent-free processing as well as the ability to coat all available surfaces of virtually any material with uniform ultrathin films of precisely controlled thickness [3]. In this work, nanostructured multilayer photoactive films have been produced on model substrates for innovative

applications in tissue engineering using photoactive amphiphilic copolymers.

The photoactive amphiphilic copolymers (photozymes) may be statistical, block or graft copolymers and they are generally water soluble. They consist of hydrophobic chromophoric and hydrophilic monomer units, in water solution they assume a pseudo-micellar conformation with a hydrophobic core. The hydrophobic domains may solubilise hydrophobic guest molecules and play the role of nanoreactors with photochemical activity [4].

These novel copolymers are advantageous for tissue engineering applications, including the engineering of peripheral nerves, as they may reduce the risks of bacterial infections by proper irradiation [5].

Materials and Methods

A graft-copolymer photozyme, chitosan-g-fluorescein (code CHFL; 0.5 mol.% FL; produced from chitosan FG 90 with Mn:210±10 kDa and Mw/Mn: 2.17, Primex Ingredients ASA) and a stat-copolymer zwitterionic photozyme, poly(sodium styrene-stat-vinyl naphtalene-stat-sulphonate-co-3-dimethyl(methacryloylethyl) ammonium propane sodium sulfonate) (PSSS-stat-VN-stat-DMAPS; code ZI; with 20 mol.% VN, 70 mol.% SSA; 10 mol.% DMAPS) were synthesized and supplied by the Laboratory of Water-Soluble Polymers, Polyelectrolytes and Biopolymers, Faculty of Chemistry, University of Sofia. Chitosan highly viscous (code CH) and poly(sodium 4-styrene-sulfonate) and (code PSS) (Mw 70.000) were purchased from Sigma Aldrich (Milano, Italy). Cast films based on gelatin (type A; Sigma-Aldrich) crosslinked with 2.5% w/w genipin (Challenge Bioproducts) were used as substrates for the coating (G_GP). LbL was realized by alternative incubation (20 min) into 0.1% (w/v) polyelectrolyte solutions (pH 4) with intermediate washing steps (5 min at pH 4).

The procedure was repeated and 10 layers were deposited, pre-coating (6 layers) of CH/PSS and 4 layers of photozymes were realized. At the end, samples were further rinsed in bi-distilled water (10 min). LbL coatings on G_GP were characterized by scanning electron microscopy-energy dispersive x-ray analysis (SEM-EDX; FEI QUANTA INSPECT 200 apparatus, equipped with EDAX Genesis Software), FTIR-ATR analysis (Perkin Elmer Spectrum One Spectrometer), UV-VIS spectroscopy (Perkin Elmer), fluorescence microscopy (Axiovert, Zeiss, Oberkochen), static water contact angle (CAM 200 KSV Instrument). MTS ([3-(4,5-dimethylthiazol-2-yl)-5-(3-carboxymethoxyphenyl)-2-(4-sulfophenyl)-2H- tetrazolium, inner salt) viability tests were performed culturing mesenchymal stem cells (MSCs) isolated from bone marrow into solutions of pure photozymes in culture medium, at various concentration (0.01-, 2.0 $mg{\cdot}ml^{-1}$), after 3 days culture time.

Results and Discussion

The presence of PSS and ZI on G_GP substrate was confirmed by FTIR-ATR and EDS analysis; the presence of CHFL was demonstrated by fluorescence microscopy. Through morphological analysis (SEM) it was observed that pre-coating of commercial polymers was homogeneous on the substrate surface, whereas surface roughness increased and agglomerates were detected on surface samples when photozymes were deposited. After the 4th layer, contact angle displayed alternate values suggesting the beginning of the layer-by-layer assembly.

The contact angles of the layers with ZI were higher than the contact angle of pure ZI photozyme, suggesting the formation of nano- and micro-aggregates on the surface based on the ZI photozyme. Biocompatibility of CHFL and ZI was evaluated through MTS test: both photozymes resulted biocompatible. The biocompatibility only slightly decreased with increasing the photozyme concentration. The ability of ZI towards singlet oxygen formation could be exploited for tailored photochemical reactions, e.g. with the purpose to obtain materials able to kill bacterials [5], increasing the well-known anti-fouling properties of zwitterionic polymers.

Conclusion

The main result of this work was the development of innovative nanostructured multifunctional coatings in which the photosensitizing properties of the different types of photozymes were combined. This coating could be proposed for implantable devices to reduce the risks of infections by irradiation during the sterilisation step (near-UV irradiation stimulating ZI copolymer) and during surgical insertion (visible light stimulating CH-FL copolymer).

The LbL technique is advantageous for the coating of nanofibres, used as functional filler for nerve guidance channels. Future works will be focused on the realization of biomimetic multilayered coatings based on layers of ZI copolymer and proteins (laminin or fibronectin). This coating will allow a combination of the antifouling and antimicrobial properties of the zwitterionic photozyme and the biomimetic properties of laminin or fibronectin, that both increase adhesion, growth, proliferation and migration of Schwann cells.

Acknowledgments – The Photonanotech project (Contract Number: 033168) is acknowledged.

References

1. Cui, F.Z., Jiao Y.P., 2007, Surface modification of polyester biomaterials for tissue engineering. Biomed.Mater.2 R24-R37.
2. Kotov, N.A., Podsiadlo, P., Tang, Z., Wang, Y., 2006, Biomedical applications of Layer-by-Layer assembly: from biomimetics to tissue engineering. Adv. Mater.6, 18, 3203-3224.
3. Channasanon, S., Graisuwan, W., Hoven, V.P., Kiatkamjornwong, S., 2007, Alternating bioactivity of multilayer thin films assembled from charged derivates of chitosano. Journal of Colloid an Interface Science 316, 331-343.
4. Burke, N.A.D., Guillet, J.E., Nowakowska, M., Paone, S., 1998, Polymer catalysts for important photoelectron transfer reactions. Macromol. Symp., vol. 134, pp. 41-49.

5. Decraene, V., Pratten, J., Wilson, M., 2006, Cellulose acetate containing toluidine blue and rose Bengal is an effective antimicrobial coating when exposed to while light, Applied And Environmental Microbiology, p. 4436–4439.
6. Sherrill, J., Michielsen, S., Stojiljkovic, I., 2003, Grafting of Light-Activated Antimicrobial Materials to Nylon Films. Journal of Polymer Science: Part A: Polymer Chemistry, Vol. 41, 41–47.

Short-lasting but not long-lasting treadmill exercise counteracts neuropathic pain and speed-up functional recovery after peripheral nerve injury

S. Cobianchi,[1] S. Luvisetto,[1] S. Marinelli,[1] F. Florenzano,[2] F. Pavone[1]

[1]*CNR, Neuroscience Institute, Rome;* [2]*CNR-EBRI-S.Lucia Foundation, Confocal Microscopy Unit, Rome, Italy.*

Introduction

Exercise is a common and necessary component of rehabilitative therapy to improve functional recovery after traumatic injuries of nervous system. Treadmill locomotion is widely used for physical rehabilitative therapy. Treadmill running show beneficial effects in animal models of peripheral injury such as the crush of sciatic nerve (Byun et al., 2005; Seo et al., 2006, 2009) or the transection of either peroneal or common fibular nerve (Marqueste et al., 2004; Sabatier et al., 2008).

Although several studies demonstrate positive effects of Treadmill running on the functional recovery after a nerve injury, the effects on pain symptoms have not been investigated, and there are not sufficient evidences to conclude which Treadmill running strategies are more effective in improving motor function after peripheral nerve injuries. In this study we analyzed which Treadmill protocol could be effective both on alleviation of neuropathic pain symptoms and on peripheral nerve regeneration and functional recovery.

Methods

Chronic Constriction Injury (CCI) model was used to induce neuropathy in CD1 male mice (Bennet and Xie, 1988). We measured the onset of mechanical allodynia in mice undergoing short- (1 week after CCI) or long-lasting (8 weeks after CCI) daily sessions (1 hour, 5 days/week) of Treadmill (running from 20cm/sec to 54cm/sec) exercise. Functional recovery of the injured paw was examined by analyzing weight bearing of hind limb, walking track analysis and sciatic static index (Baptista et al., 2007). Behavioral data were correlated with data from immunofluorescence staining of biological markers for cellular proliferation (Cdc2, GAP-43) in injured nerves and for activated glial cells (Cd11b, GFAP) in lumbar spinal cord.

Results

An early mild and short-lasting Treadmill exercise was effective in counteracting the development of mechanical allodynia induced by CCI and to speed up the functional recovery of injured paw, as demonstrated by the normalization of the weight bearing and of walking pattern of mice. On the contrary, long-lasting Treadmill exercise did not show substantial beneficial effects compared to animals that were not subjected to Treadmill. Behavioural data strongly correlated with nerve regeneration immunofluorescence markers. Only the short-lasting Treadmill exercise was coupled with an increased expression of Cdc2 and GAP-43, proteins associated with regenerative processes, in injured sciatic nerves. In the same mice group, a reduced expression of Cd11b and GFAP-labeled glial cells was also observed in the lumbar spinal cord.

In conclusion, our results show that the duration of Treadmill exercise is fundamental for modulating painful symptoms induced by neuropathy and for accelerating the functional recovery of the injured hind paw. We suggest that only an immediate short-lasting exercise induces pain relief, stimulates nerve regeneration, and speeds up the complete functional recovery.

References

Baptista AF, de Souza Gomes JR, Oliveira JT, Santos SMG, Vannier-Santos MA, Martinez AMB, 2007. A new approach to assess function after sciatic nerve lesion in the mouse – Adaptation of the sciatic static index. J Neurosci Meth 161:259-264.

Bennet GJ, Xie YK, 1988. A peripheral mononeuropathy in rat that produces disorders of pain sensation like those seen in man. Pain 33:87-107.

Byun YH, Lee MH, Kim SS, Kim H, Chang HK, Lee TH, Jang MH, Shin MC, Shin MS, Kim CJ, 2005. Treadmill running promotes functional recovery and decreases brain-derived neurotrophic factor mRNA expression following sciatic crushed nerve injury in rats. J Sports Med Phys Fitness 45:222-228.

Marqueste T, Alliez JR, Alluin O, Jammes Y, Decherchi P, 2004. Neuromuscular rehabilitation by treadmill running or electrical stimulation after peripheral nerve injury and repair. J Appl Physiol 96:1988-1995.

Sabatier MJ, Redmon N, Schwartz G, English AW, 2008. Treadmill training promotes axon regeneration in injured peripheral nerves. Exp Neurol 211:489-493.

Ultrastructural study of the effects of FK506 administration to the rat sciatic nerve

O. Evirgen,[1] F. Topal,[1] O. Semiz,[2] M. Akbari,[3] AA.K. Sargın,[1] B. Can[1]

[1]Department of Histology and Embryology, Ankara University Faculty of Medicine, Ankara,, oya.evirgen@gmail.com, belgincan@yahoo.com, ferdatopal@gmail.com, drayse1980@yahoo.com; [2]Sakarya University School of Health Esentepe Campus, Sakarya, Turkey, osemiz@sakarya.edu.tr

Introduction

FK506 (Tacrolimus) derived from streptomyces tsukubaensis is a widely used immunosuppressive drug for prevention of graft rejection in patients following organ transplantations. Like Cyclosporin A (CyA), its immunosuppressive effect is achieved by blocking calcineurin activity that inhibits T-cell activity (Tan, Timothy C. et al., 2006). During FK506 treatment neurotoxicity is a common side effect and more commonly seen in peripheral neurvous system (PNS) than central nervous system. In PNS Schwann cells are sensitive to neurotoxicity and showed demyelinating and axonal damage. Peripheral neuropathy is reversible after the withdrawal of the drug (Wijidicks, Eelco F.M., 2001). Beside its immunosuppressive effect it has been reported in literature that FK506 also improve the rate of axonal regeneration after nerve injury (Gold,Bruce G. et al., 1995). A wide range of different dosages (1,2,5 mg/kg to 10mg/kg) of FK506 have been used for nerve regeneration in literature , but there is no consensus about its optimal regenerative dosage and administration route (Yang ,Roberta K. et al., 2003; Albert Pan,Y. et al., 2003). In this study we aimed to study the ultrastructural effects of FK506 administrated (2 mg/kg/day) orally for 7,14,21 days on normal rat sciatic nerve.

Material and Methods

A total number of 20 male Wistar Albino rats (weighing 200-250gr) were randomly divided into four groups; group-1 control and FK506-treated groups as group-2 for 7 days, group-3 for 14 days and group-4 for 21 days. All procedures were approved by Animal care and Usage Ethics Committee of Ankara University (2008-29-139).

Drug administration FK506 (Prograf 5mg hard capsules Astellas Pharma Co. Ltd., Dublin 22) 2mg/kg/day was given via orogastric route by a feeding canulla.

Tissue fixation and preparation: The rats were sacrificed by an ether overdose and bilaterally removed sciatic nerves were sampled for each group. For electron microscopy tissue samples fixed in 2,5 % gluteraldehyde were processed and embedded in araldite. Semithin sections were stained with Toluidine Blue- Azul II and observed under a light microscope. Ultra thin sections stained with uranyl acetate and lead citrate were observed under LEO 906 E Transmission electron microscope

Results

In the control group the sciatic nerve light and electron microscopy showed normal morphology (figure: 1 A, a, B, C). The examination of semithin sections from rats of group-2 revealed the appearance of axonal dilatation (figure: 1 D, E). In group-3 light microscopy showed normal and pathologic areas in the same nerve. Pathologic areas presented loosely laminated myelin sheaths, demyelination and degenerating axons. Few number of mast cells were observed within the nerve between the myelinated and unmyelinated axons (figure:1 F, f, ff, G). Ultrastructurally there were sings of myelin degradation forming myelin debris within the endoneurium and small to large vacuols in myelin (figure: 1 H, I, J).In group-4 semithin sections there were supernumerary mast cells between the axons and also in the connective tissue surrounding the nerve fascicle (figure:1 K, k, kk). Ultrastructural analysis showed redundant myelin loops and degenerated regions in myelin sheaths, there were a moderate connective tissue increase between the axons .(figure: 1 L, M, N, O).

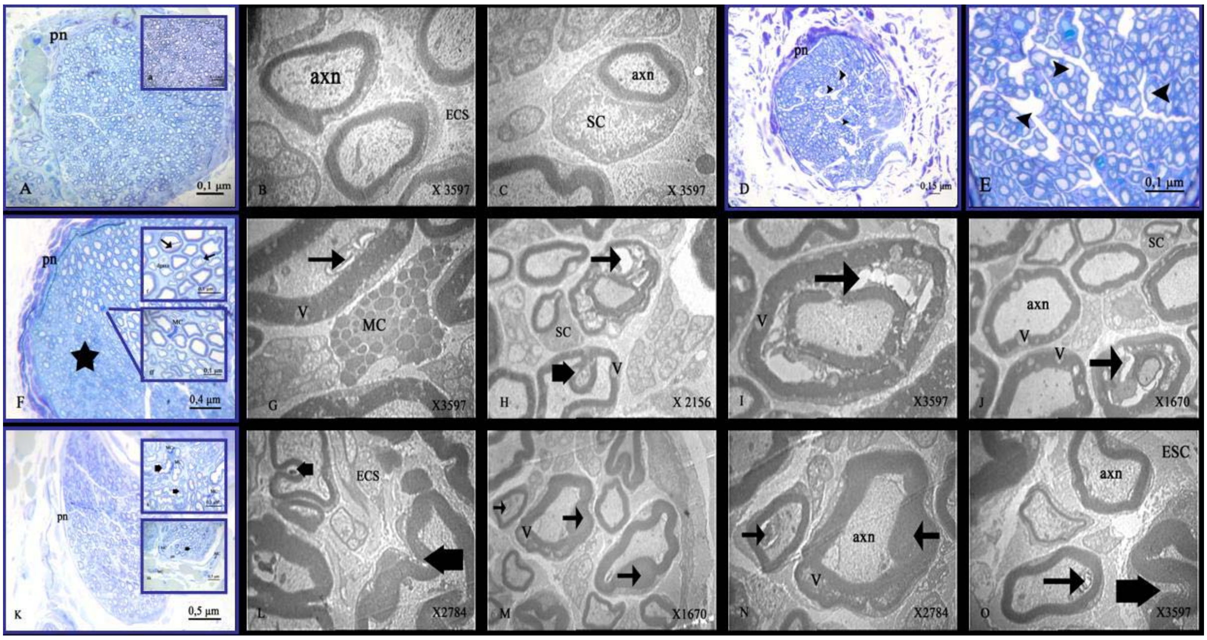

Figure 1: pn: perineurium, axn: axon, ECS: extra cellular space, arrow heads: dilated axons, dgaxn: degenerated axon, MC: mast cell, star: pathologic area, thin arrows: loose myelin lamella, irregular myelin, SC: Schwann cell, V: vacuols, thick arrows: redundant myelin loops.

Discussion

One of the major side effect during the treatment with FK506 is neurotoxicity. Neurotoxicity can be attributed to prolonged drug exposure of neuronal tissue and the effect of calcineurin inhibition on neuronal function. Beside its neurotoxicity several studies have demonstrated that FK506 has beneficial effects on neuroregeneration after nerve injury when used at low subimmunosuppresive doses.

The exact mechanism , dose and the administration route of drug to promote the neurodegenerative effects is still unclear. In the present study we administered minimal immunosuppressive dose of FK506 orally instead of subcutaneous injections that has been shown to prevent skin allograft rejection in rats. (Yang,Roberta K. et al., 2003). Morphological sings of neurotoxicity such as myelin disintegration, axonal degeneration was much evident in rats of group-3 receiving the drug for 14 days. 21 days after in group-4 ultrastructurally sciatic nerve showed remyelinating axons and redundant myelin loops suggesting an adaptive neuroregeneration.

Increased mast cell numbers in group-3 indicate that these cells might play an important role and regulation in neuroregeneration by releasing growth factors, proteolitic enzymes, tumor necrosis factor alpha (TNF-a),interleukins and cytokines. Also histamin may be important in opening the blood nerve barrier (Leon, A. et al., 1994; Naidu, M., 2009).

However alterations in mast cell functions may lead pathological conditions such as neurofibromas by providing mitogenic secretions that induce cell proliferation(Viskochil, David H., 2003).

References

Albert Pan,Y., Misgeld,T.,Lichtman,JeffW.,Sanes,JoshuaR.,2003. Effects of neurotoxic and neuroprotective agents on peripheral nerve regeneration assayed by time-lapse imaging in vivo. The Journal of Neuroscience.23(36),11479-11488.

Gold,Bruce G., Katoh,Kiyoshi., Storm-Dickerson. Toni., 1995. The immunosuppressant FK506 increases the rate of axonal regeneration in rat sciatic nerve. The Journal of Neuroscience. 15(11),7509-7516.

Leon,A., Buriani,A., Dal Toso,R., Fabris, M., 1994. Mast cells synthesize, store, and release nerve growth factor. Neurobiology. 91,3739-3743.

Naidu,M.,2009.The role of cells, neutrophils, extracellular matrix and cell surface molecules in peripheral nerve regeneration. Malaysian Journal of Medical Sciences. 16(2),10-14.

Tan,Timothy C., Robinson, Phillip J.,2006. Mechanisim of calcineurin inhibitor-induced neurotoxicity. Transplantation Reviews.20,49-60.

Viskochil, DavidH., 2003. It takes two to tango: mast cell and Schwann cell interactions in neurofibromas. The Journal of Clinical Investigation. 112(12),1791-1793.

Wijidicks, EelcoF.M., 2001. Neurotoxicity of immunosuppressive drugs. Liver Transplantation. 7(11),937-942.

Yang,Roberta K., Lowe,James B., Sobol, Julia B., 2003. Dose-dependent effects of FK506 on neuroregeneration in a rat model. Plastic and Reconstructive Surgery. 112(7), 1832-1840.

Axonotmesis-related changes in sensory neurons

L. Muratori, A. Cunotto, S. Raimondo, G. Ronchi, S. Geuna, M. Fornaro
Department of Clinical and Biological Sciences, University of Turin, Orbassano (TO), Italy

The evidence of new-generated neurons in sensory ganglia of adult animals has been a matter of controversy since it was first reported (Devor and Govrin-Lippmann, 1985, 1991; La Forte et al., 1991). Although some have pointed out the possible existence of neurogenesis in adult DRGs, others have suggested that these new neurons could instead derive from the maturation or growth of preexisting immature cells.

In the present study, we investigate using different experimental approaches, the effect of a nerve crush lesion on DRGs sensory neurons in adult rat. The crush injury was applied to the median, ulnar and radial nerves at their point of origin from the brachial plexus using a non-serrated clamp that could guarantee standardized and reproducible method. Animals were then sacrificed at several time points after the injury and DRG corresponding only to the level C5-T1, that give rise to the fibers of the radial, ulnar and median nerves, were extracted.

Our morphological data in optical and electron microscopy show no evidences of cell sufferance as a consequence of the nerve injury. FACS analysis confirmed that there is no significant lost of sensory neurons. However, a stereological analysis using the physical dissector method showed a significant increase in number (42%) of sensory neurons 1 month after injury. BrDU-immunopositive neuronal nuclei strongly reinforce our findings suggesting that neurogenesis occurs in DRG as a consequence of nerve crush injury. Further investigating the fate of the overpopulation of neurons, crush injury was performed and DRG harvested after 2, 3 and 6 months.

Already after 2 months, the number of neurons decreased to a number similar to control. Immunohistochemistry and western blot analysis confirmed that apoptosis occurs between 1 and 2 months from the nerve lesion.

The results of this study suggested the following hypothesis: DRG neuronal population increases at first as a consequence of the peripheral nerve damage. Right after lesion, the neuronal population start growing new generated axons towards the peripheral targets. Functional tests of muscle-recovery suggested that this process usually occurs in about 1 month. Whenever the fibers regeneration is completed, the overpopulation of neurons that never reached the periphery undergoes to apoptosis.

References

Devor M, Govrin-Lippmann R (1985) Neurogenesis in adult rat dorsal root ganglia. Neurosci Lett 61:189-194.

Devor M, Govrin-Lippmann R (1991) Neurogenesis in adult rat dorsal root ganglia: on counting and the count. Somatosens Mot Res 8:9-12.

La Forte RA, Melville S, Chung K, Coggeshall RE (1991) Absence of neurogenesis of adult rat dorsal root ganglion cells. Somatosens Mot Res 8:3-7.

Significance of gp130 signaling in neuronal regeneration

S. Quarta, N. Scherbakov, M. Andratsch, M. Kress
Division of Physiology and Medical Physics, Innsbruck Medical University, Austria; E-mail: serena.quarta@i-med.ac.at

Background

The cytokine interleukin-6 (IL-6) activates target genes involved in differentiation, survival, apoptosis and proliferation as well as in inflammatory processes. It acts by binding to plasma membrane receptor complexes containing the common signal transducing receptor subunit gp130. Subsequently and by activation of JAK, STAT and MAPK signaling pathways, IL-6 regulates various target genes leading to its broad effects. Still, it is poorly understood how IL6/gp130 signals participate in the regeneration of peripheral neurons. In the present work, we have investigated the role of gp130 in the regeneration of peripheral sensory neurons using conditional knock-out mice (SNS-gp130-/-) (Andratsch et al., J. Neurosci., 2009 Oct; 29 (43): 13473-13483).

Methods

Biochemical approaches, quantitative PCR and fluorescence microscopy were used. A nerve injury model, obtained by a one minute crush of the sciatic nerve, and sensory and motor behavioral tests were used to monitor the recovery process in vivo.

Results

Dorsal root ganglion neurons (DRGs) from SNS-gp130-/- mice showed a significantly reduced neurite extension and number of neurite bearing neurons in culture as compared to gp130fl/fl and wild-type animals. In vivo, after

nerve crush injury regeneration of the sciatic nerve was monitored for 25 days by determining mechanical and heat thresholds and motor capabilities in control and knock-out mice. Recovery of sensitivity was similar in wt and gp130fl/fl mice but significantly delayed in SNS-gp130-/- mice. No significant difference was observed in recovery of motor capabilities between the groups.

In order to elucidate the effects of IL-6 and gp130 on peripheral regeneration and to find potential signaling partners we used Affymetrix® gene expression analysis of DRG explants from SNS-gp130-/- mice and gp130fl/fl controls. We found down-regulation of two regeneration-associated genes (RAGs), Atf3 and Sprr1a. Using L4-L6 DRG explants from non-injured animals the mRNA expression in SNS-gp130-/- mice was determined by quantitative PCR. mRNA of both Atf3 and Sprr1A were lower in SNS-gp130-/- DRG explants compared to gp130fl/fl mice. qRT-PCR showed an increase in mRNA expression of both genes after sciatic nerve injury at 3 and 7 days.

Conclusion

Our data suggest that regeneration of neurons is significantly inhibited in SNS-gp130 in vitro and in vivo. We found that genes that are associated with nerve injury or regulation of neurite outgrowth are down-regulated in SNS-gp130-/- mice. Experiments addressing the functional importance of gp130/IL6 signaling in the outgrowth process of peripheral neurons are ongoing.

Acknowledgments – Supported by FWF (P18444) and DK SPIN.

References

Andratsch et al., The Journal of Neuroscience 29 (43), October 2009; 13473-13483.
Ernst et al., TRENDS in Genetics 20(1), January 2004; 23-32.
Cafferty et al., The Journal of Neuroscience 24(18), May 2004; 4432-4443.
Seijffers et al., The Journal of Neuroscience 27(30), July 2007; 7911-7920.
Starkey et al., The Journal of Comparative Neurology 513, 2009; 51-68.

Peripheral glial cell differentiation from neurospheres derived from adipose mesenchymal stem cells

C. Radtke,[1,2,3] B. Schmitz,[1] M. Spies,[4] J.D. Kocsis,[2,3] P.M. Vogt[1]

[1]Department of Plastic, Hand- and Reconstructive Surgery, Hannover Medical School, 30659 Hannover; [2]Department of Neurology and Center for Neuroscience and Regeneration Research, Yale University School of Medicine, New Haven, CT 06510, USA; [3]Rehabilitation Research Center, Veterans Affairs Connecticut Healthcare System, West Haven, CT 06516, USA; [4]Department of Plastic, Hand- and Reconstructive Surgery, Barmherzige Brüder Regensburg, Germany

Mesenchymal stem cells derived from bone marrow and adipose tissue are being considered for use in neural repair because they can differentiate after appropriate induction in culture into neurons and glia.

The question we asked was if neurospheres could be harvested from adipose-derived stem cells and if they then could differentiate in culture to peripheral glial-like cells. Here, we demonstrate that adipose-derived mesenchymal stem cells can form nestin-positive non-adherent neurosphere cellular aggregates when cultured with basic fibroblast growth factor and epidermal growth factor. Dissociation of these neurospheres and removal of mitogens results in expression of the characteristic Schwann cell markers S100 and p75 nerve growth factor receptor and GFAP. The simultaneous expression of these glia markers are characteristic features of Schwann cells and olfactory ensheathing cells which have unique properties regarding remyelination and enhancement of axonal regeneration. When co-cultured with dorsal root ganglion neurons, the peripheral glial-like cells derived from adipose mesenchymal stem cells aligned with neuritis and stimulated neuritic outgrowth.

These results indicate that neurospheres can be generated from adipose-derived mesenchymal stem cells, and upon mitogen withdrawal can differentiate into peripheral glial cells with neurotrophic effects.

Median nerve regeneration is increased in ErbB2 transgenic mice

P. Salamone, F. Di Scipio, G. Ronchi, A.E. Sprio, P. Tos, S. Geuna, G.N. Berta

Department of Clinical and Biological Sciences, San Luigi Gonzaga Hospital, University of Turin, Italy

Since peripheral nerves injuries have high worldwide prevalence and incidence, in the last decade the experimental investigation about nerve function recovery is rising. Unlike central nervous tissue, peripheral nerve fibers are able to functionally regenerate due to their permissive environment. The understanding of nerve regeneration mechanisms is interesting to indentify new molecular targets to facilitate and speed up the physiological injury recover and to improve the post-operative outcome. Schwann cells are key elements in this process but the

biomolecular signals driving the regeneration have not been completely characterized. Recent data shown that neuregulin-mediated HER family (Human Epidermal growth factor Receptor, transmembrane receptor with tyrosine kinase activity), in particular ErbB2, is involved in the regulation of Schwann cells activity, and hence in the molecular response to peripheral nerve injuries.

To study the involvement of ErbB2 in the biological process of spontaneous nerve regeneration, we analyzed the condition after a crush injury in genetically modified mice over-expressing this receptor compared to wild type mice used as controls.

Postoperative recovery was regularly evaluated by the grasping test: results indicate that functional recovery of the transgenic mice is significantly higher than the control ones starting from the 1st week.

Nerve fibers regeneration was assessed by quantitative stereology of myelinated ones: the transgenic mice show a very evident increase in the total fiber number which is more than sixty percent, and it is significant compared to WT mice (see figure).

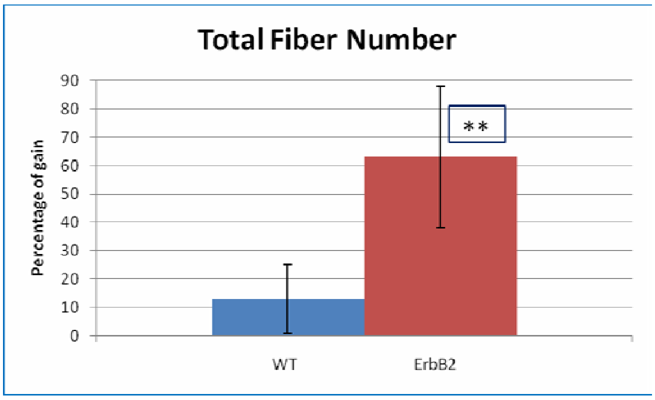

Preliminary results show that over-expression of ErbB2 induces a faster recovery after crush injury, and morphological analysis demonstrates that this is probably due to an increased number of regenerated axons. This study may provide the biological basis for an innovative clinical strategy for promoting nerve regeneration after lesion through the pharmacological manipulation of Erb system.

Oxidative stress and effect of antioxidant treatment in an animal model of compressive neuropathy

D. Tomassoni,[1] L. Di Cesare Mannelli,[2] C. Ghelardini,[1] F. Amenta[3]

[1]*Dipartimento di Scienze Morfologiche e Biochimiche Comparate, Università di Camerino;* [2]*Dipartimento di Farmacologia Preclinica e Clinica, Università di Firenze, Firenze;* [3]*Dipartimento di Medicina Sperimentale e Sanità Pubblica, Università di Camerino, Camerino.*

Entrapment and compressive neuropathies of the upper and lower extremities are frequently encountered disorders. Treatment of these disorders is still problematical, lacking relevant information about their etiology and pathophysiology. Loose ligation of the rat sciatic nerve represents a model of peripheral neuropathy. The purpose of the present investigation was to assess if similarly as reported in other neuropathies, compression of the sciatic nerve is accompanied by an increased oxidative stress and if damage induced by nerve ligation is countered by treatment with the antioxidant thioctic or alpha-lipoic acid (ALA). Analysis was performed in an obvious model of increased oxidative stress represented by spontaneously hypertensive rats (SHR).

Loose ligation of the right sciatic nerve was performed in 20-week-old and SHR and age-matched normotensive Wistar-Kyoto rats (WKY) used as a reference group. The left sciatic nerve served as control. Another group of both SHR and WKY rats was sham-operated. Animals with sciatic nerve ligation were left untreated or were treated with ALA (25 and 50 mg/Kg/day i.p. for 14 days), (R+)-ALA-treated (25 mg/Kg/day i.p. for 14 days) and pregabalin (50 mg/Kg/day for 14 days) rats.

At the end of experiments paw-pressure test for assessing analgesic effect elicited by different treatments was used. Animals were then killed by decapitation under anaesthesia, blood was collected and serum obtained. Malondialdehyde (MDA) as a marker of oxidative stress was then assayed. Both ligated and control sciatic nerves were dissected out and investigated microanatomically using Masson's trichromic technique for evaluating sciatic nerve microanatomy, phosphorylated 200-kDa neurofilament (NFP) and myelin basic like-protein (MBP) immunohistochemistry.

In SHR either control or treated pharmacologically, significantly higher values of systolic and diastolic blood pressure compared to WKY rats were found. Different treatments did not change blood pressure values. Analysis of MDA revealed an increased plasma oxidative stress in sham-operated SHR compared to WKY rats. Oxidative stress was more pronounced in SHR and WKY with sciatic nerve ligated compared with sham-operated rats. Treatment with (R+)-ALA and with the higher dose of ALA and to a lesser extent with ALA 25 mg/Kg/day or pregabalin countered the increase of MDA.

Repeated ALA (50 mg/Kg/day) or (R+)-ALA treatment increased the nociceptive threshold in the right paw. The activity of the higher dose of ALA and of (R+)-ALA was comparable with that of pregabalin. No significant differences in nociceptive threshold were observed between WKY rats and SHR with right sciatic nerve ligation. Microanatomical analysis revealed revealed that treatment

with the higher dose of ALA and (R+)-ALA countered nerve injury.

The above data indicate an increased oxidative stress in this animal model of compressive neuropathy. The demonstration of an activity of ALA on both stress and peripheral nerve injury suggests that antioxidant strategy may represent an approach in the treatment of compressive neuropathies.

Nanofibrous matrices for nerve regeneration

C. Tonda-Turo,[1] V. Chiono,[1] E. Cipriani,[2] C. Audisio,[3] S. Geuna,[4] M. Zanetti,[2] I. Perroteau,[3] G. Ciardelli[1]

[1]Department of Mechanic, Politecnico di Torino, e-mail: chiara.tondaturo@polito.it, gianluca.ciardelli@polito.it; [2]Nanostructured Interfaces and Surfaces (NIS) Centre of Excellence, Department of Chemistry IFM, University of Turin, e-mail: marco.zanetti@unito.it; [3]Department Of Human and Animal Biology, University of Turin, e-mail: chiara.audisio@unito.it; [4]Department of Clinical and Biological Science, University of Turin, Italy, e-mail: stefano.geuna@unito.it

Introduction

Fibrous matrices mimic the complex biological structures and provide the mechanical support to allow the cells of the damaged tissue to remodel and repair forming three-dimensional tissue structures that resemble the original tissue. In recent years, electrospinning has been extensively used to construct tissue-engineered scaffolds because it is a simple fabrication process that can easily produce nano- and micro-sized polymeric fibres[1].

Various biomaterials including natural and synthetic polymers (non-degradable and degradable) have been investigated as nerve conduits. Biodegradable synthetic polymers such as aliphatic polyesters and copolyesters can degrade during the tissue regeneration process; variations in their chemical or engineering properties may change biocompatibility, degradation behaviour, porosity and mechanical strength. On the other hand, natural polymers are advantageous materials for tissue engineering of nerves as they are biocompatible, favour the migration of supporting cells and avoid the occurrence of toxic affects. Natural materials are usually hydrophilic and possess good cell compatibility and interaction, but they may suffer from batch-to-batch variability and need extensive purification and characterization[2]. Furthermore most materials of natural origin lack adequate mechanical strength and must therefore be crosslinked to meet mechanical requirements. Gelatin-based biomaterials have been applied as artificial skin, bone grafts, and scaffolds for tissue engineering. The main limitation of gelatin (GL) for application in tissue engineering is its solubility in aqueous solution; therefore, crosslinking is necessary to increase gelatin stability in biological environment. Genipin (GP) is a naturally occurring crosslinking agent, its biocompatible, and less-toxic than traditional chemical agents, such as formaldehyde, glutaraldehyde and epoxy compounds [3].

The aim of this study was to prepare and characterize GP crosslinking GL matrices prepared by electrospinning to be applied as a substrate for peripheral nerve regeneration.

Material and Methods

GL (type A from porcine skin) was supplied from Aldrich. GP was purchased from Challenge Bioproducts LTD. GL was dissolved in demineralised water at 50°C to obtain a 2.5% (wt/v) solution.
Crosslinked GL films were prepared by casting. GP were added to the GL solution at a 2.5% wt/wt amount with respect to GL. Nanofibrous scaffolds were prepared by electrospinning technique using a flat aluminium plate to collect randomly oriented nanofibers (table 1). The nanofibrous matrices obtained were stabilized using a dehydrothermal process (DHT) and finally the fiber mats were crosslinked by immersion in GP solutions in ethanol at 2.5% (w/v).

Table 1. Electrospinning conditions of the different GL samples

Spinning parameters sample A	Solution: 8%(%w/w)<br>Flow rate: 10 µl/min<br>Needle-collector distance: 15cm<br>Applied Voltage: 30 kV
Spinning parameters sample B	Solution: 10%(%w/w)<br>Flow rate: 10 µl/min<br>Needle-collector distance: 15cm<br>Applied Voltage: 30 kV
Spinning parameters sample C	Solution: 12%(%w/w)<br>Flow rate: 10 µl/min<br>Needle-collector distance: 15cm<br>Applied Voltage: 30 kV

Spinning parameters sample D	Solution: 15%(%w/w)<br>Flow rate: 10 µl/min<br>Needle-collector distance: 15cm<br>Applied Voltage: 30 kV
Spinning parameters sample E	Solution: 18%(%w/w)<br>Flow rate: 10 µl/min<br>Needle-collector distance: 15cm<br>Applied Voltage: 30 kV

The obtained scaffolds were characterized in terms of their morphology and porosity (scanning electron microscopy), stability (swelling and degradation), chemical (infrared spectroscopy) and thermal properties (differential scanning calorimetry) and mechanical behaviour. The matrices biocompatibility were tested using different cell lines such as neonatal olfactory bulb ensheathing cells (NOBEC).

Results and Discussion

The GP crosslinked GL films increased their weight of about eight times after 24 hours in aqueous medium showing a high degree of water uptake. The dissolution test reported a loss of half of the initial weight after one week.

- Solution concentration:
  - lower than 8% (w/w): no fiber formation and droplets of different size were obtained
  - higher than 18% (w/w): the solution was highly viscous and spinning was not feasible
- The fiber diameter increased with increasing solution concentration
- The pore dimension did not show any trend versus GL solution concentration.

The sample D (figure 1) was selected for the crosslinking process.

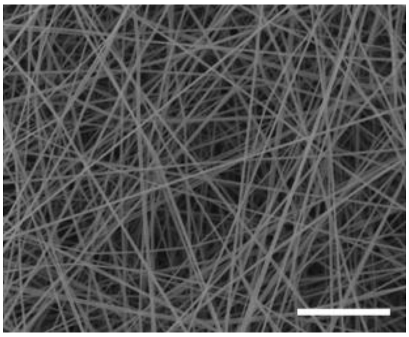

Figure 1. SEM micrography of the sample D before the crosslinking process.

The fiber structure was fixed by a preliminary DHT process, followed by immersion in a GP solution. However, the second treatment caused fiber aggregation. The high degree of swelling of GL induced a new fiber distribution after the crosslinking process.
Cell tests showed that the GP-crosslinked GL samples supported NOBEC cells adhesion and proliferation: the density of cells on the substrate was similar to the control.

Conclusion

Operative conditions for GL electrospinning at mild conditions were found. A method for successful crosslinking of GL was developed using a not-cytotoxic agent, GP, after a stabilisation pre-treatment by DHT. GP-crosslinked samples were found to be suitable for NOBEC adhesion and proliferation. The preliminary evaluation of this work suggested the potential of GP-crosslinked nanofibers for applications in the field of nerve repair.

References

[1] Agarwal, S., Wendorff, J.H., Greiner, A., 2008. Use of electrospinning technique for biomedical applications. Polymer 49, 5603–5621
[2] Ciardelli, G., and Chiono, V., 2006. Materials for peripheral nerve regeneration. Macromol. Biosci. 6,13–26
[3] Muzzarelli, R.A.A., 2009. Chitins and chitosans for the repair of wounded skin, nerve, cartilage and bone. Carboh. Polym. 76, 167–82.

A new technique of autogenous conduits for bridging short nerve defects: An experimental study in the rabbit

V.Tsiampa,[3] I.Ignatiadis,[1] S.Galanakos,[1] A.Avram,[1] A. Papalois[2]

[1]Hand Surgery and Microsurgery Department, KAT Hospital, Athens; [2]Experimental Research Unit ELPEN PHARMA, Athens, Greece; [3]Second Orthopaedic Dept, Athens University Medical School, Greece

Aim

Nerve grafting, veins and muscle[1] are commonly used autogenous conduits[2] to repair short neural gaps, providing good results but associated with donor site morbidity. In this study we try to evaluate peripheral nerve regeneration through epineural conduits. Artificial conduits[3,4] have been used both experimentally and clinically for bridging short nerve defects, obtaining as well similar with the above results. The epineurium until today was considered as a taboo concerning its use in peripheral nerve surgery because of its high contribution in nerve vascularisation. Therefore just a few studies have been reported about epineurium possibilities in nerve gap bridging[5,6,7].

Material and Methods

126 white New Zealand rabbits, weighing between 3 and 3.5 kg respectively were allocated to 4 Groups. In each of the 3 study groups (Groups A, B and C) 36 animals were included, while the rest 18 animals served as control (Group D). In all groups a 10-mm sciatic nerve defect was created and bridged either with 3 variations of an epineural flap (Groups A, B and C) or with a nerve graft (Group D).

An advancement epineural flap harvested from the proximal nerve stump and from the distal nerve stump were employed in Groups A and B respectively. In Group C a specially designed reversed epineurial flap harvested from the proximal stump was employed while in the control group the defect was bridged using the excised portion of the sciatic nerve, which was sutured in its original site.

Results

Following sacrifice all animals were examined using light microscopy and immunocytochemistry. Nerve regeneration was studied in transverse sections at 3, 6 and 9 mm from the proximal stump. Immunohistochemical, histochemical and functional evaluation morphometrically analyzed showed nerve regeneration closed to the grafted nerve control series, especially for the group A.

Conclusions

The epineurium may serve as an autologous conduit, which facilitates nerve defects bridging.

References

1. Whitworth IH, Dore CJ, Green CJ, Terenghi G. Increased axonal regeneration over long nerve gaps using autologous nerve-muscle sandwich grafts. Microsurgery 1995;16:772–778.
2. Suematsu N. Tubulation for peripheral nerve gap: Its history and possibility. Microsurgery 1989;10:71–74.
3. Strauch B. Use of nerve conduits in peripheral nerve repair. Hand Clinics 2000;16:123–130.
4. Urabe T, Zhao Q, Danielsen N, Lundborg G. Regeneration across a partial defect in rat sciatic nerve encased in a silicon chamber. Scand J Plast Reconstruct Hand Surg 1994;30:7–15.
5. Ayhan S, Markal N, Siemionow K, Araneo B, Siemionow M. Effect of subepineurial dehydroepiandrosterone treatment on healing of transected nerves repaired with the epineurial sleeve technique. Microsurgery 2003;23:49–55.
6. Siemionow M, Tetik C, Ozer K, Ayhan S, Siemionow K, Browne E. Epineural sleeve neurorrhaphy: Surgical technique and functional results—A preliminary report. Ann Plast Surg 2002;48:281–285.
7. Tetik C, Ozer K, Ayhan S, Siemionow K, Browne E, Siemionow M. Conventional versus epineural sleeve neurorhaphy technique: Functional and histomorphometric analysis. Ann Plast Surg 2002;49:397–403.

We wish to thank for sponsoring this Symposium:

Leica
MICROSYSTEMS

Nikon

2B
2BIOLOGICAL INSTRUMENTS S.N.C.

SIGMA-ALDRICH®
