# Supplementary material for: International symposium on peripheral nerve repair and regeneration and 2nd club Brunelli meeting
Source: J Brachial Plex Peripher Nerve Inj. 2010 Mar 9;5:5. doi: 10.1186/1749-7221-5-5 (PMC2848046; doi:10.1186/1749-7221-5-5)
Supplement: Additional file 2 — Slides of oral presentation from the keynote lecture from Professor Giorgio Brunelli. [file 1749-7221-5-5-S2.PPT]

## Slide 1
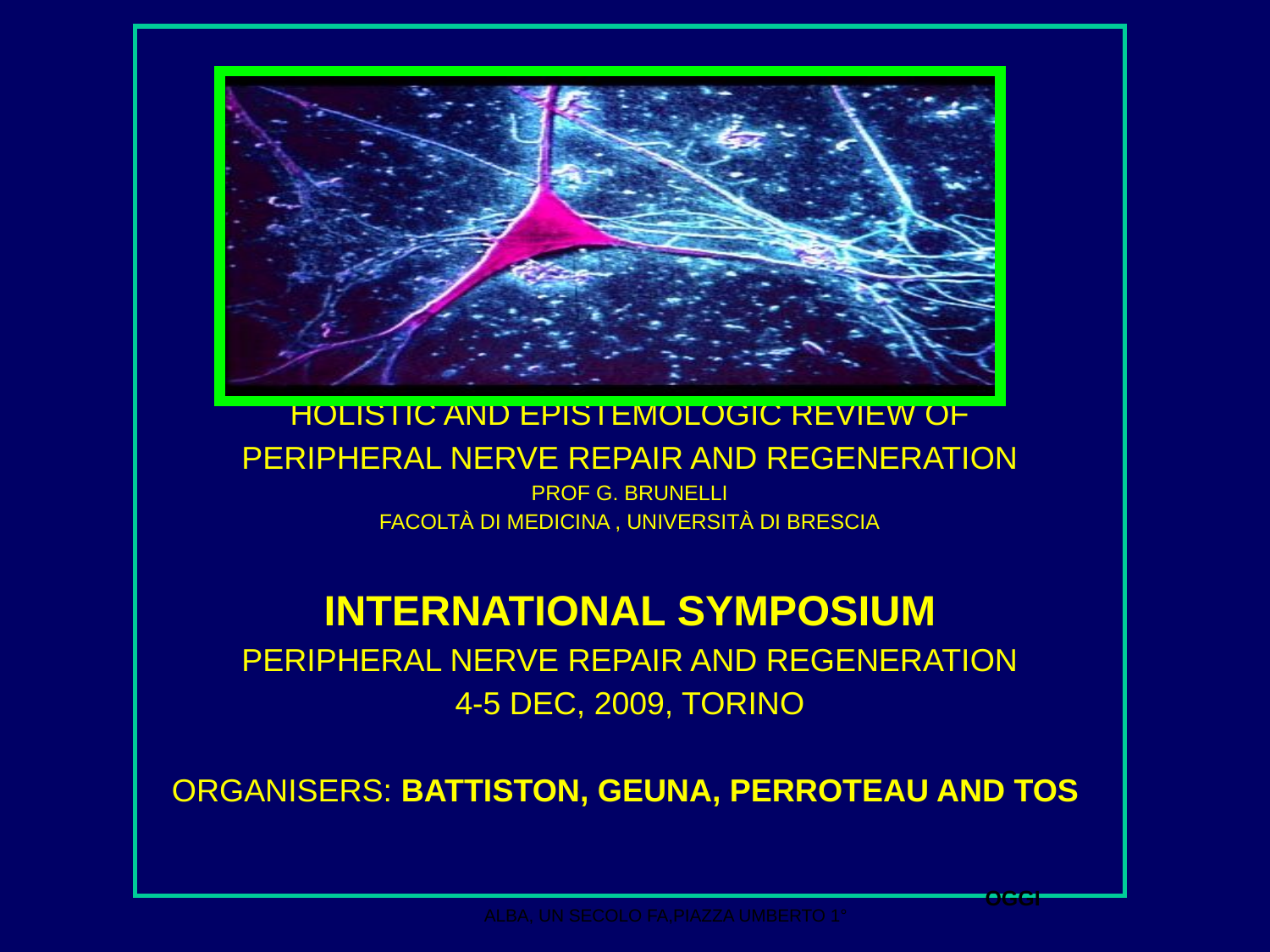

| HOLISTIC AND EPISTEMOLOGIC REVIEW OF PERIPHERAL NERVE REPAIR AND REGENERATION PROF G. BRUNELLI FACOLTÀ DI MEDICINA , UNIVERSITÀ DI BRESCIA INTERNATIONAL SYMPOSIUM PERIPHERAL NERVE REPAIR AND REGENERATION 4-5 DEC, 2009, TORINO ORGANISERS: BATTISTON, GEUNA, PERROTEAU AND TOS |
| --- |
 ALBA, UN SECOLO FA,PIAZZA UMBERTO 1°
OGGI

## Slide 2
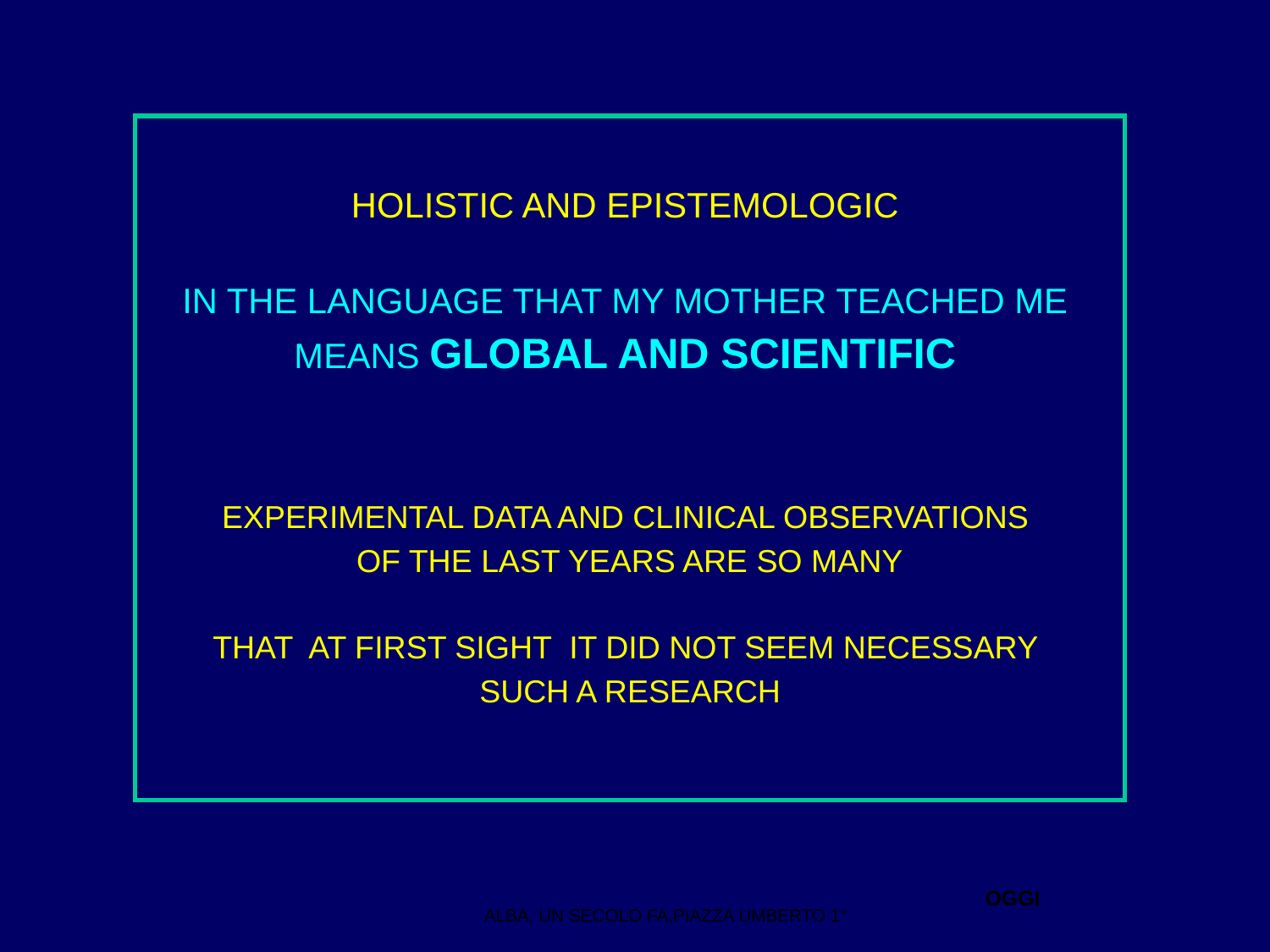

| HOLISTIC AND EPISTEMOLOGIC IN THE LANGUAGE THAT MY MOTHER TEACHED ME MEANS GLOBAL AND SCIENTIFIC EXPERIMENTAL DATA AND CLINICAL OBSERVATIONS OF THE LAST YEARS ARE SO MANY THAT AT FIRST SIGHT IT DID NOT SEEM NECESSARY SUCH A RESEARCH |
| --- |
 ALBA, UN SECOLO FA,PIAZZA UMBERTO 1°
OGGI

## Slide 3
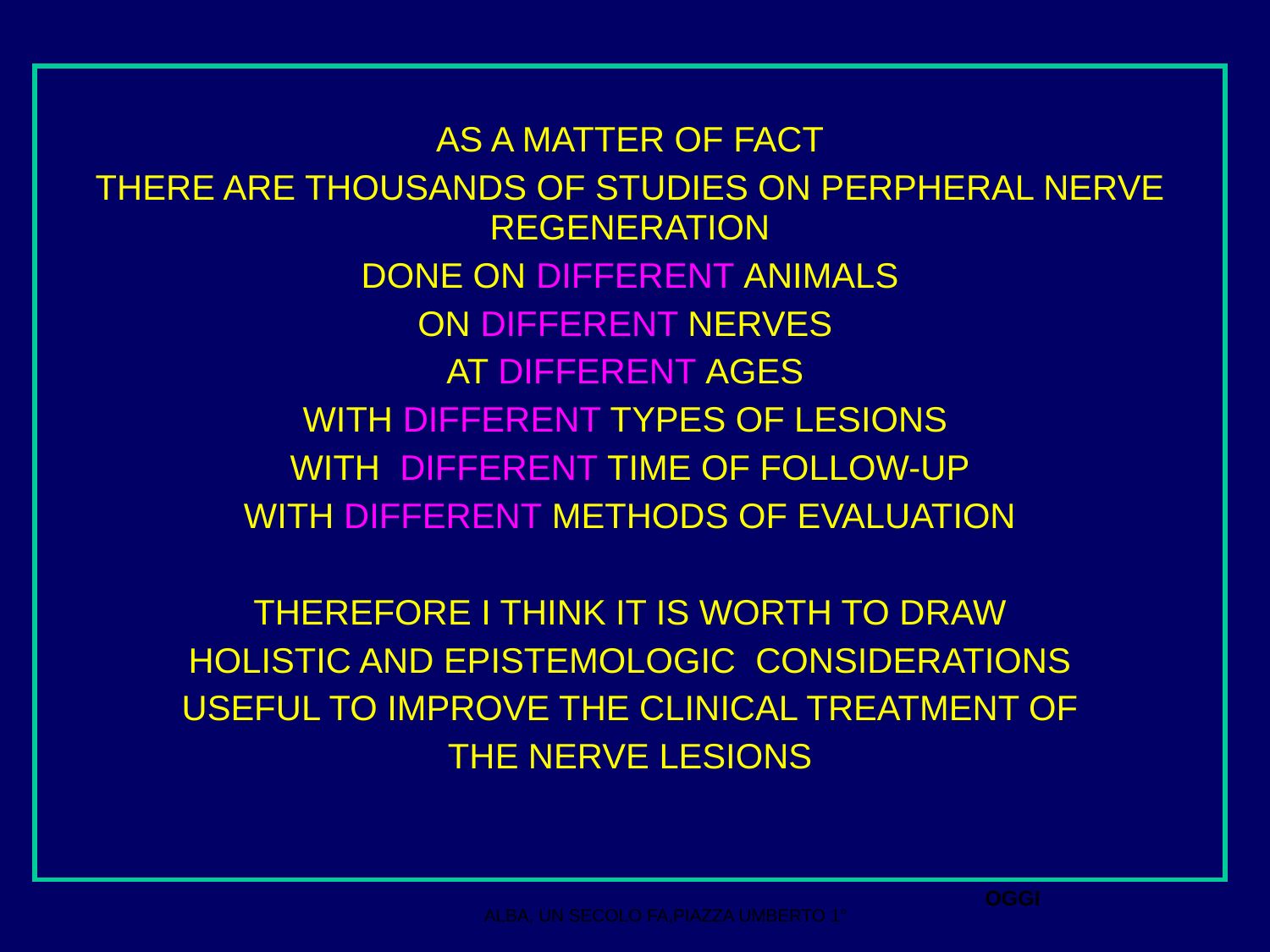

| AS A MATTER OF FACT THERE ARE THOUSANDS OF STUDIES ON PERPHERAL NERVE REGENERATION DONE ON DIFFERENT ANIMALS ON DIFFERENT NERVES AT DIFFERENT AGES WITH DIFFERENT TYPES OF LESIONS WITH DIFFERENT TIME OF FOLLOW-UP WITH DIFFERENT METHODS OF EVALUATION THEREFORE I THINK IT IS WORTH TO DRAW HOLISTIC AND EPISTEMOLOGIC CONSIDERATIONS USEFUL TO IMPROVE THE CLINICAL TREATMENT OF THE NERVE LESIONS |
| --- |
 ALBA, UN SECOLO FA,PIAZZA UMBERTO 1°
OGGI

## Slide 4
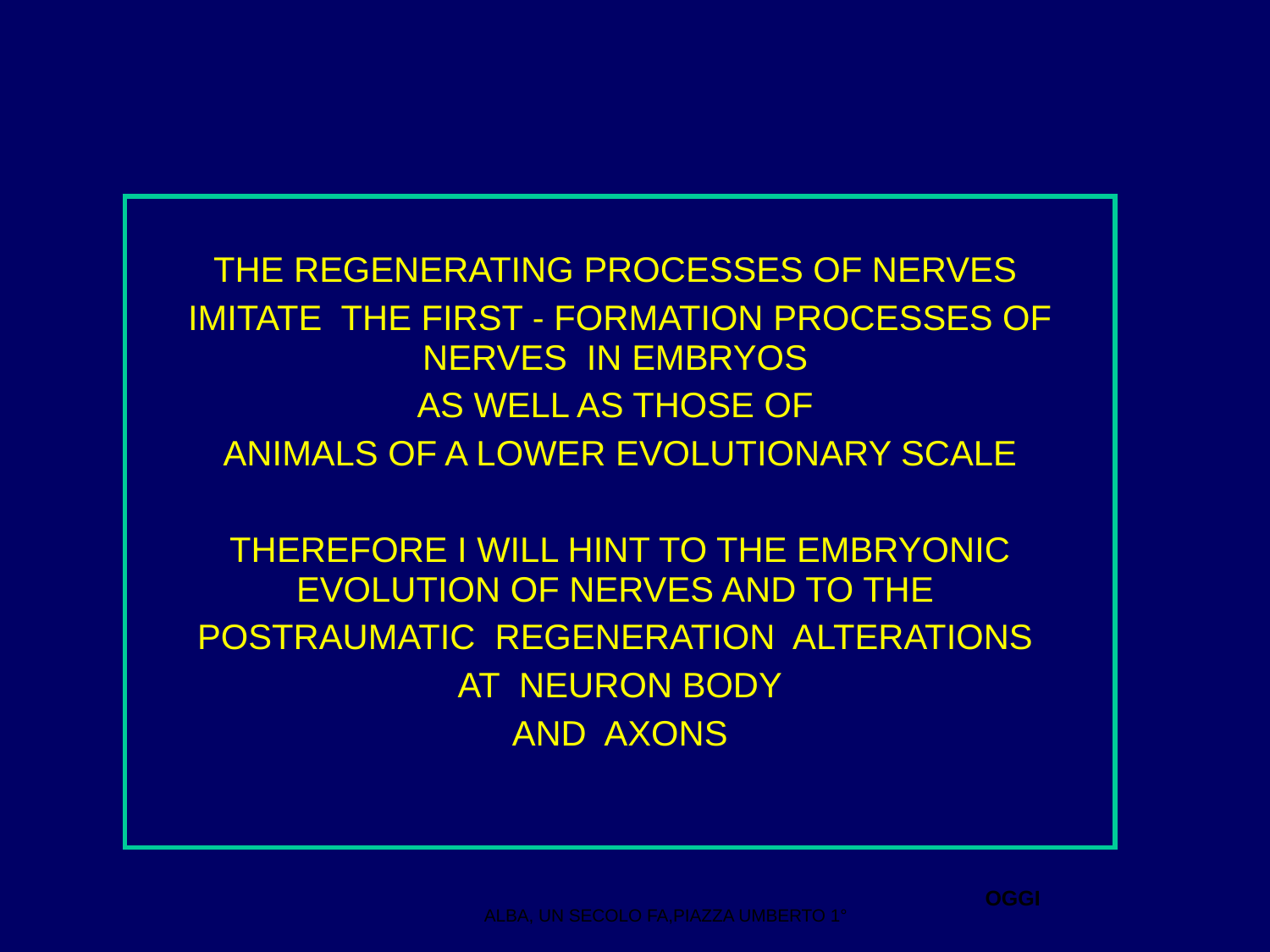

| THE REGENERATING PROCESSES OF NERVES IMITATE THE FIRST - FORMATION PROCESSES OF NERVES IN EMBRYOS AS WELL AS THOSE OF ANIMALS OF A LOWER EVOLUTIONARY SCALE THEREFORE I WILL HINT TO THE EMBRYONIC EVOLUTION OF NERVES AND TO THE POSTRAUMATIC REGENERATION ALTERATIONS AT NEURON BODY AND AXONS |
| --- |
 ALBA, UN SECOLO FA,PIAZZA UMBERTO 1°
OGGI

## Slide 5
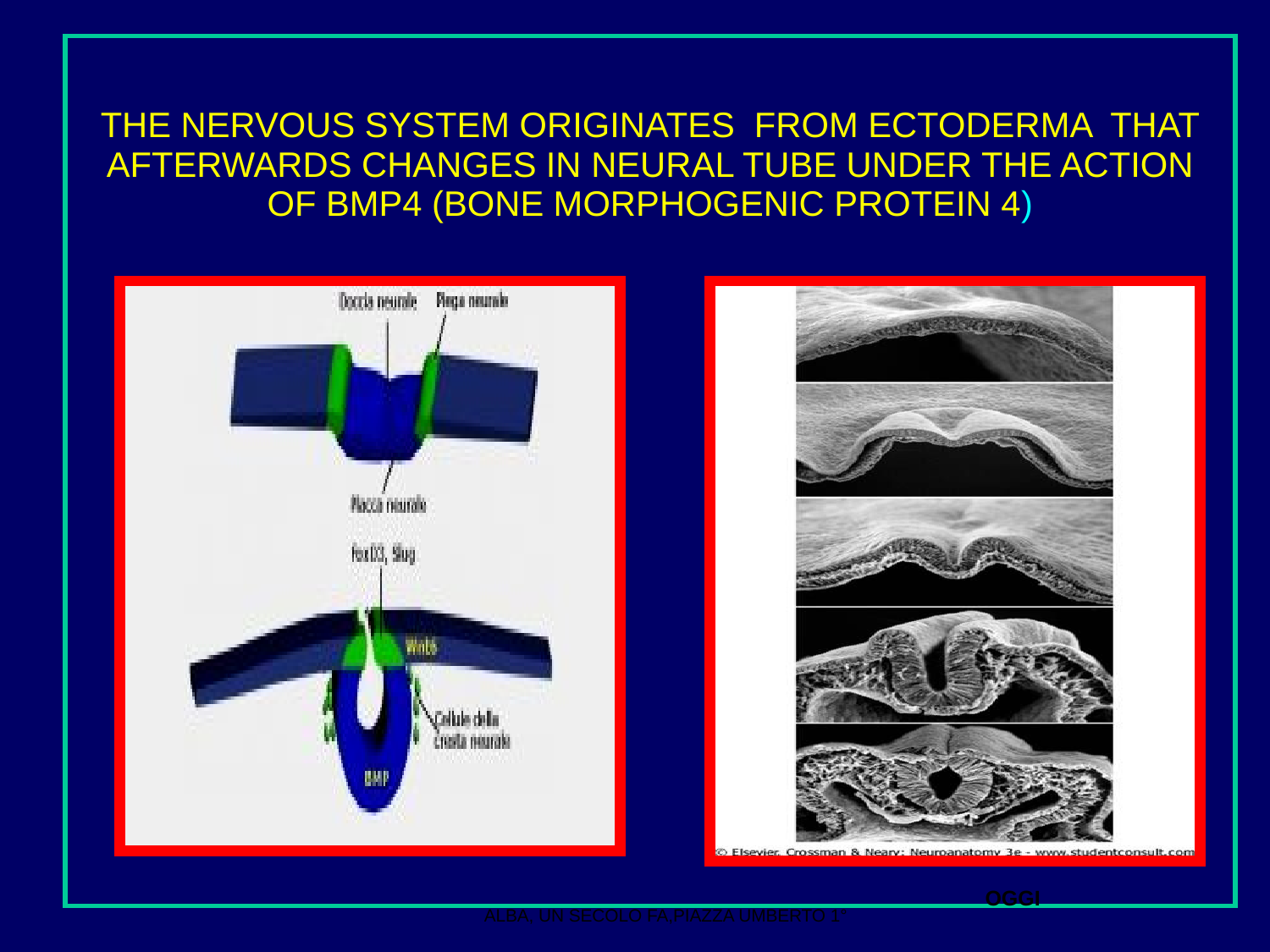

| THE NERVOUS SYSTEM ORIGINATES FROM ECTODERMA THAT AFTERWARDS CHANGES IN NEURAL TUBE UNDER THE ACTION OF BMP4 (BONE MORPHOGENIC PROTEIN 4) |
| --- |
 ALBA, UN SECOLO FA,PIAZZA UMBERTO 1°
OGGI

## Slide 6
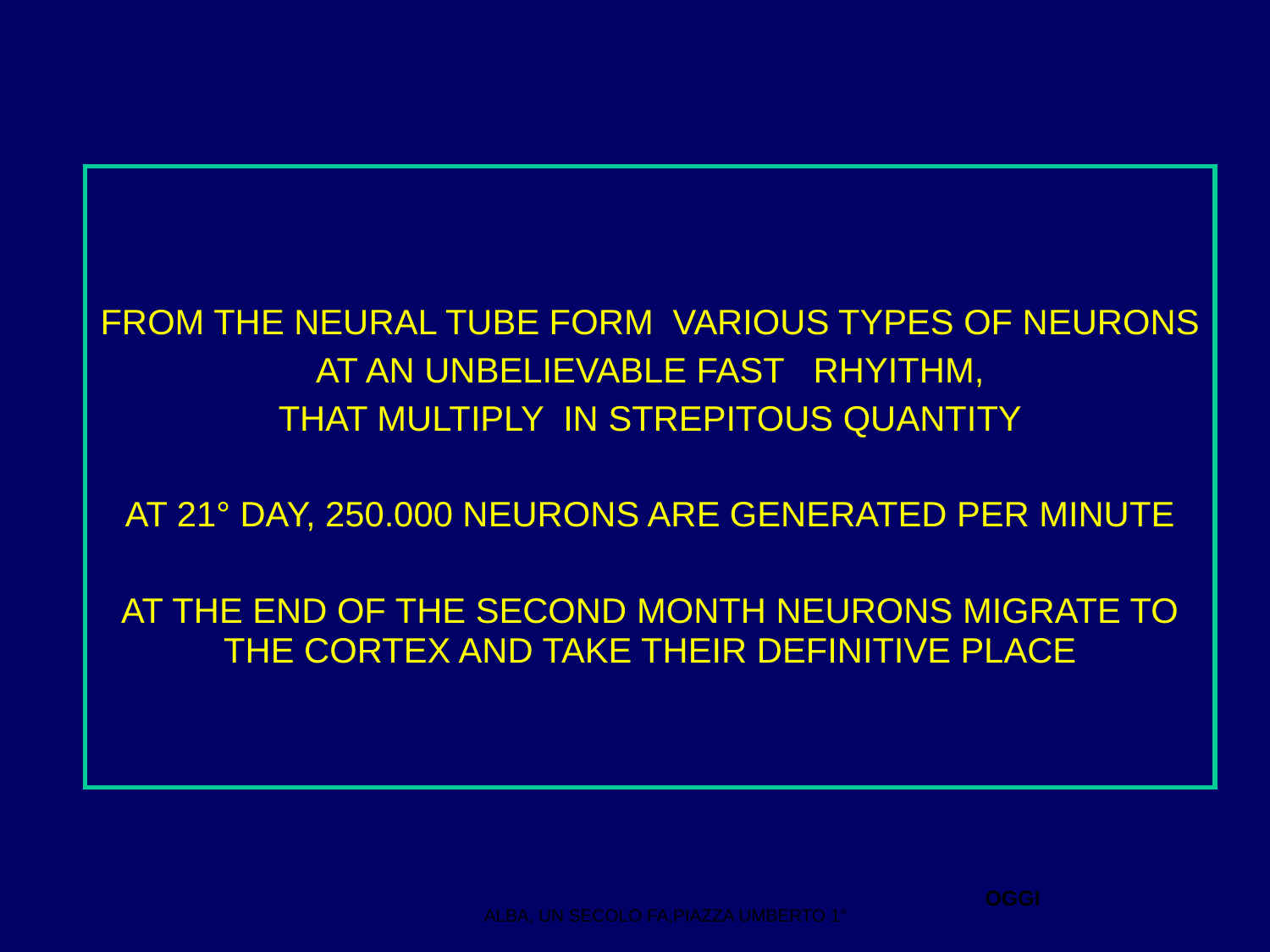

| FROM THE NEURAL TUBE FORM VARIOUS TYPES OF NEURONS AT AN UNBELIEVABLE FAST RHYITHM, THAT MULTIPLY IN STREPITOUS QUANTITY AT 21° DAY, 250.000 NEURONS ARE GENERATED PER MINUTE AT THE END OF THE SECOND MONTH NEURONS MIGRATE TO THE CORTEX AND TAKE THEIR DEFINITIVE PLACE |
| --- |
 ALBA, UN SECOLO FA,PIAZZA UMBERTO 1°
OGGI

## Slide 7
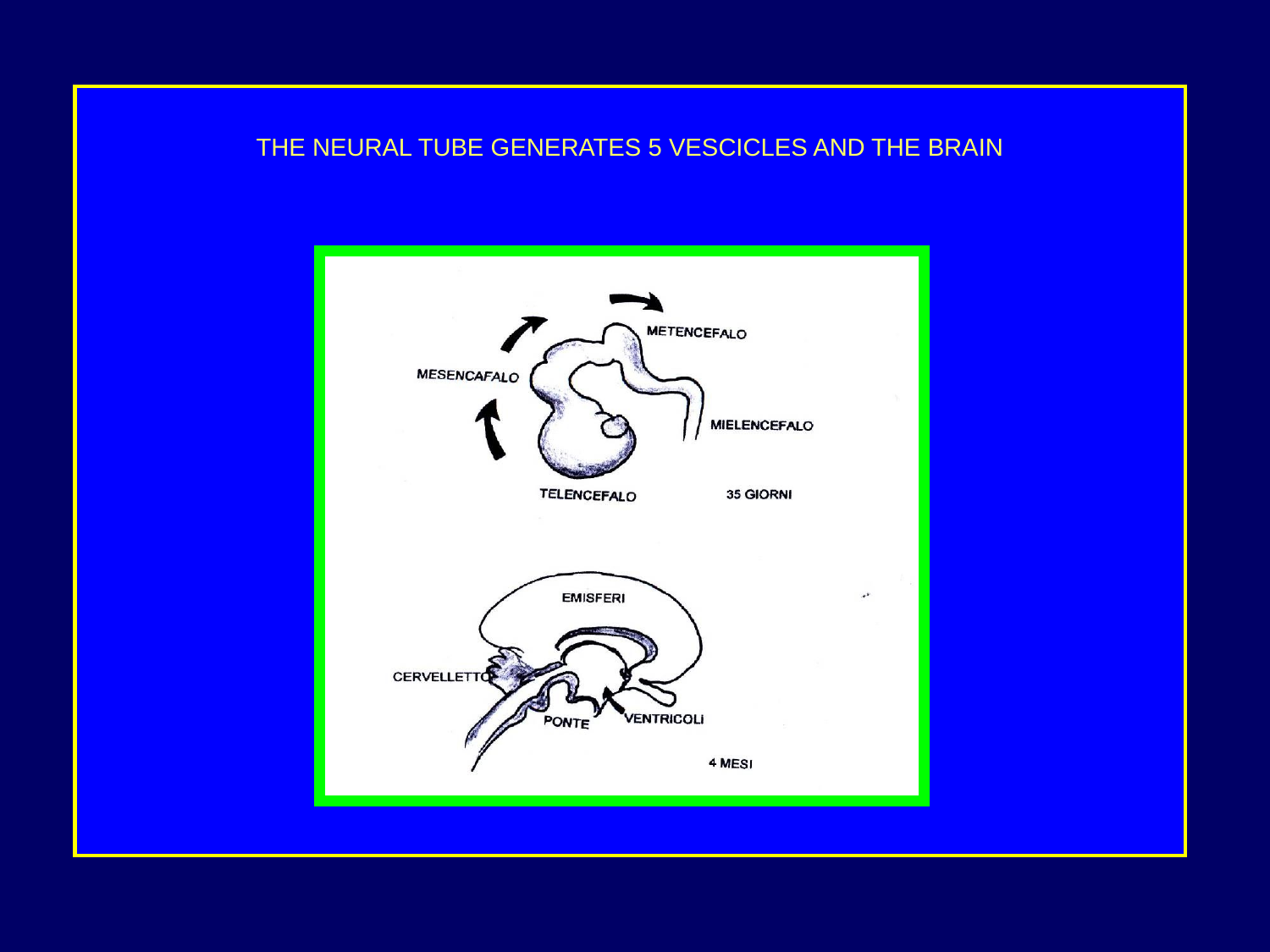

#
| THE NEURAL TUBE GENERATES 5 VESCICLES AND THE BRAIN |
| --- |

## Slide 8
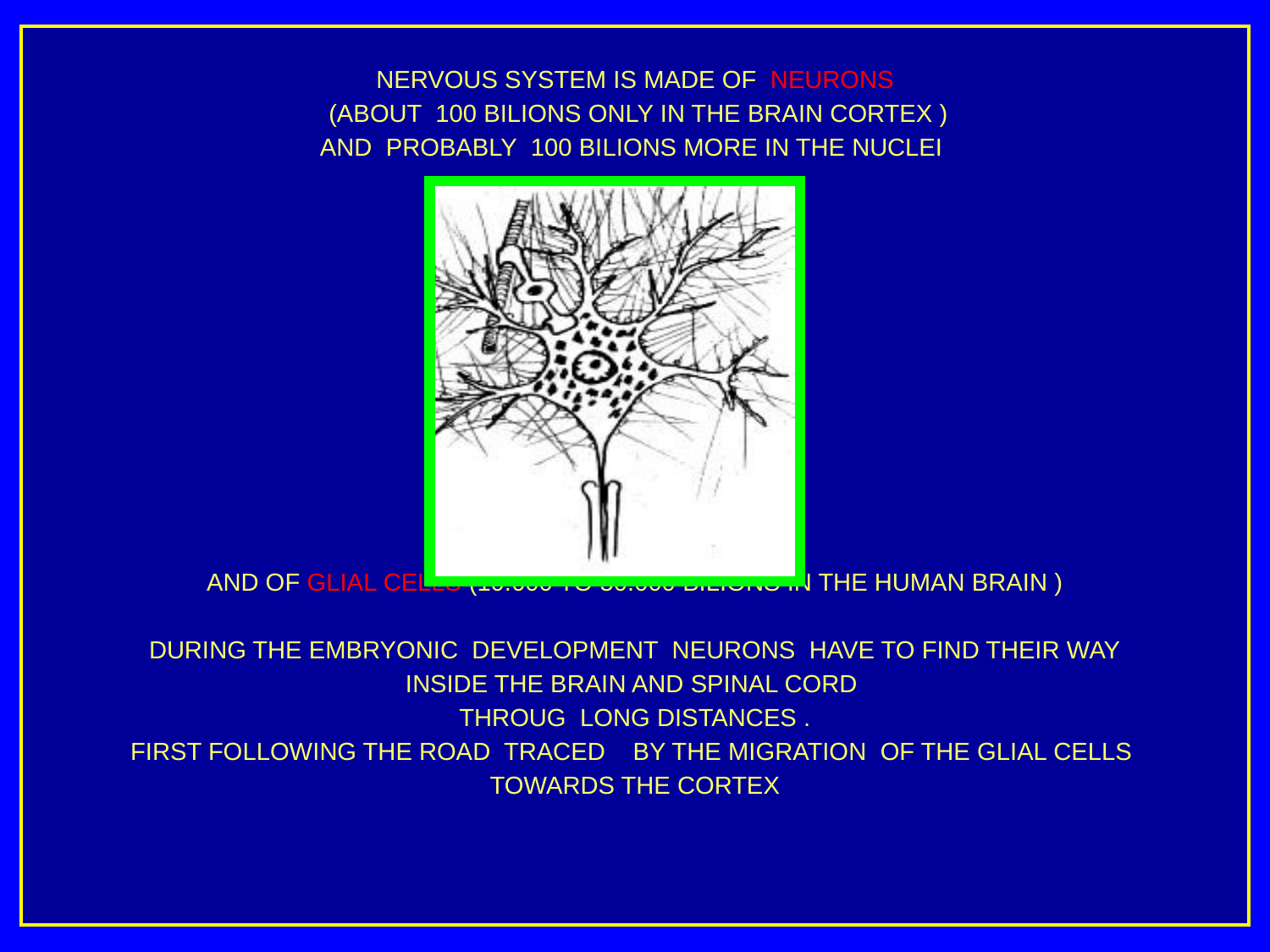

| NERVOUS SYSTEM IS MADE OF NEURONS (ABOUT 100 BILIONS ONLY IN THE BRAIN CORTEX ) AND PROBABLY 100 BILIONS MORE IN THE NUCLEI AND OF GLIAL CELLS (10.000 TO 50.000 BILIONS IN THE HUMAN BRAIN ) DURING THE EMBRYONIC DEVELOPMENT NEURONS HAVE TO FIND THEIR WAY INSIDE THE BRAIN AND SPINAL CORD THROUG LONG DISTANCES . FIRST FOLLOWING THE ROAD TRACED BY THE MIGRATION OF THE GLIAL CELLS TOWARDS THE CORTEX |
| --- |

## Slide 9
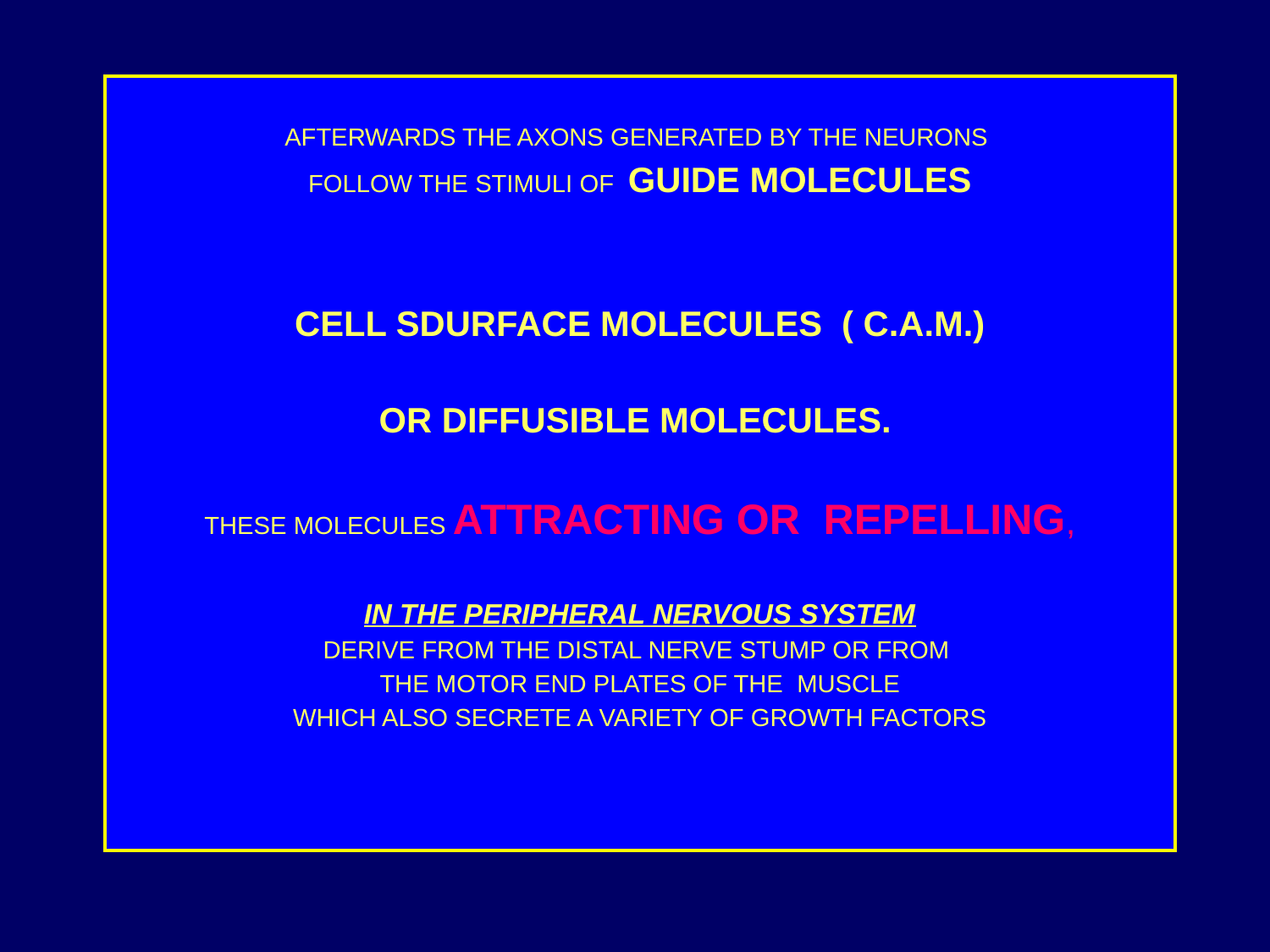

| AFTERWARDS THE AXONS GENERATED BY THE NEURONS FOLLOW THE STIMULI OF GUIDE MOLECULES CELL SDURFACE MOLECULES ( C.A.M.) OR DIFFUSIBLE MOLECULES. THESE MOLECULES ATTRACTING OR REPELLING, IN THE PERIPHERAL NERVOUS SYSTEM DERIVE FROM THE DISTAL NERVE STUMP OR FROM THE MOTOR END PLATES OF THE MUSCLE WHICH ALSO SECRETE A VARIETY OF GROWTH FACTORS |
| --- |

## Slide 10
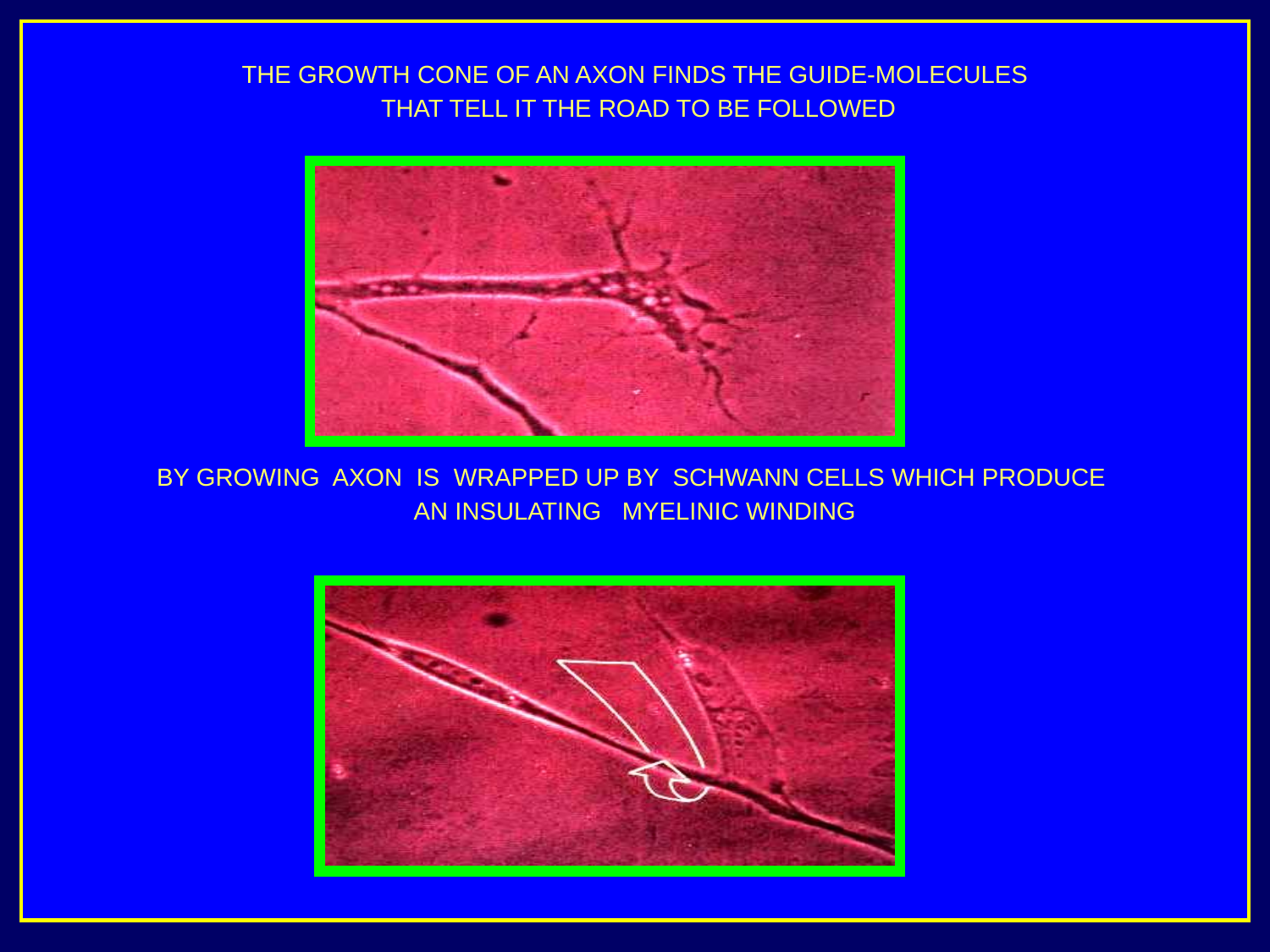

| THE GROWTH CONE OF AN AXON FINDS THE GUIDE-MOLECULES THAT TELL IT THE ROAD TO BE FOLLOWED BY GROWING AXON IS WRAPPED UP BY SCHWANN CELLS WHICH PRODUCE AN INSULATING MYELINIC WINDING |
| --- |

## Slide 11
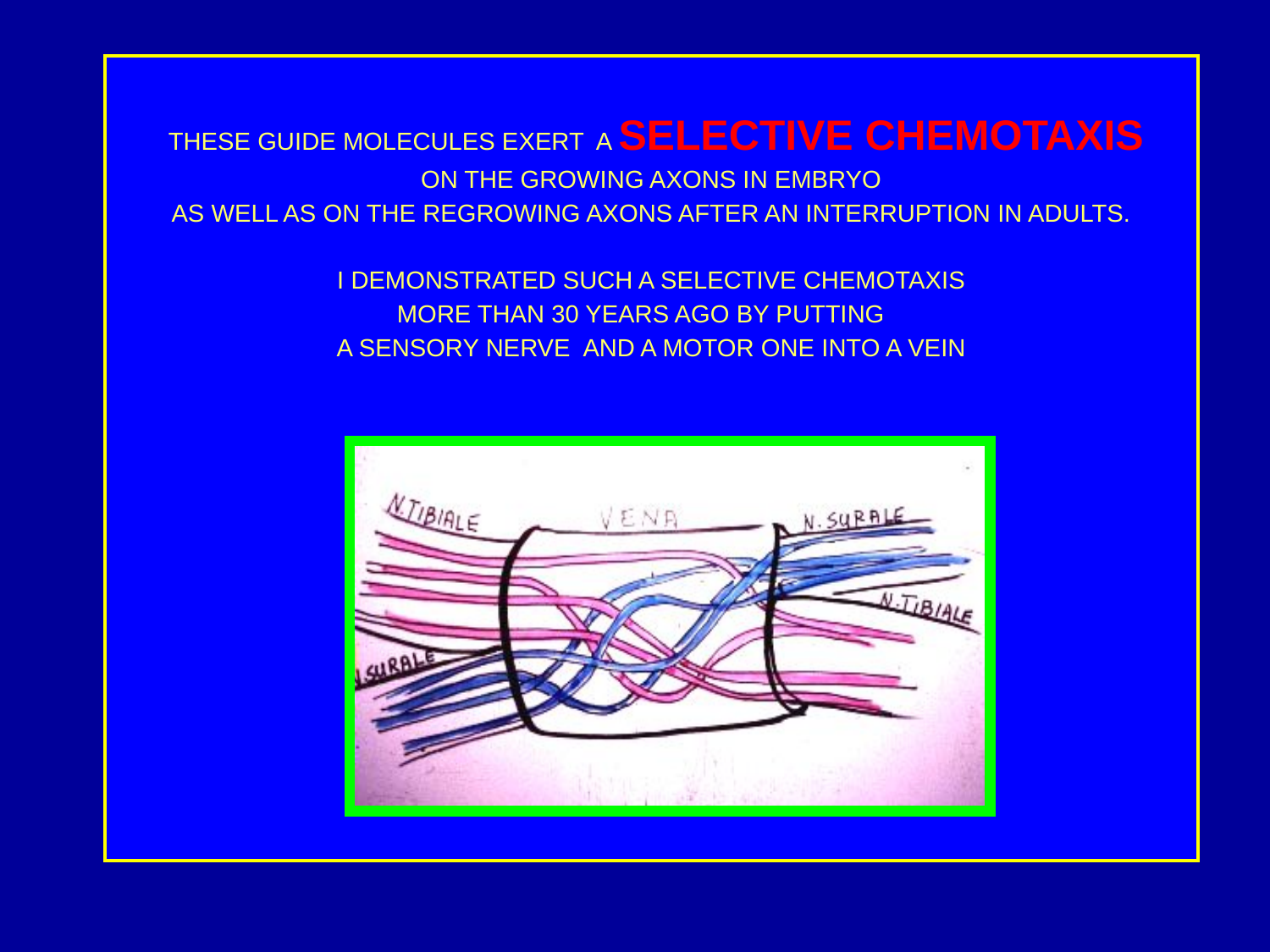

| THESE GUIDE MOLECULES EXERT A SELECTIVE CHEMOTAXIS ON THE GROWING AXONS IN EMBRYO AS WELL AS ON THE REGROWING AXONS AFTER AN INTERRUPTION IN ADULTS. I DEMONSTRATED SUCH A SELECTIVE CHEMOTAXIS MORE THAN 30 YEARS AGO BY PUTTING A SENSORY NERVE AND A MOTOR ONE INTO A VEIN |
| --- |
 NELLA VENA

## Slide 12
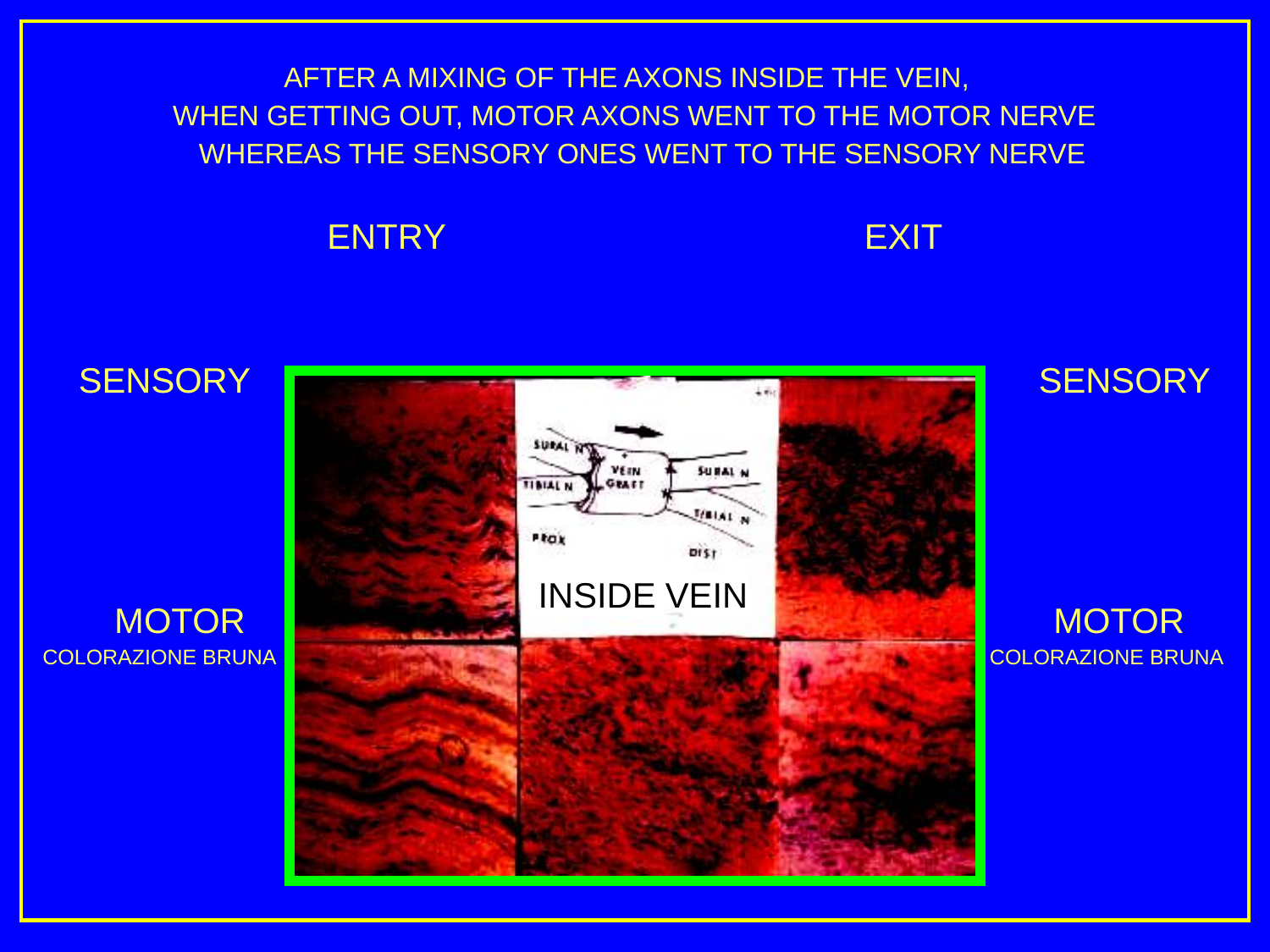

| AFTER A MIXING OF THE AXONS INSIDE THE VEIN, WHEN GETTING OUT, MOTOR AXONS WENT TO THE MOTOR NERVE WHEREAS THE SENSORY ONES WENT TO THE SENSORY NERVE ENTRY EXIT SENSORY SENSORY MOTOR MOTOR COLORAZIONE BRUNA COLORAZIONE BRUNA |
| --- |
 INSIDE VEIN

## Slide 13
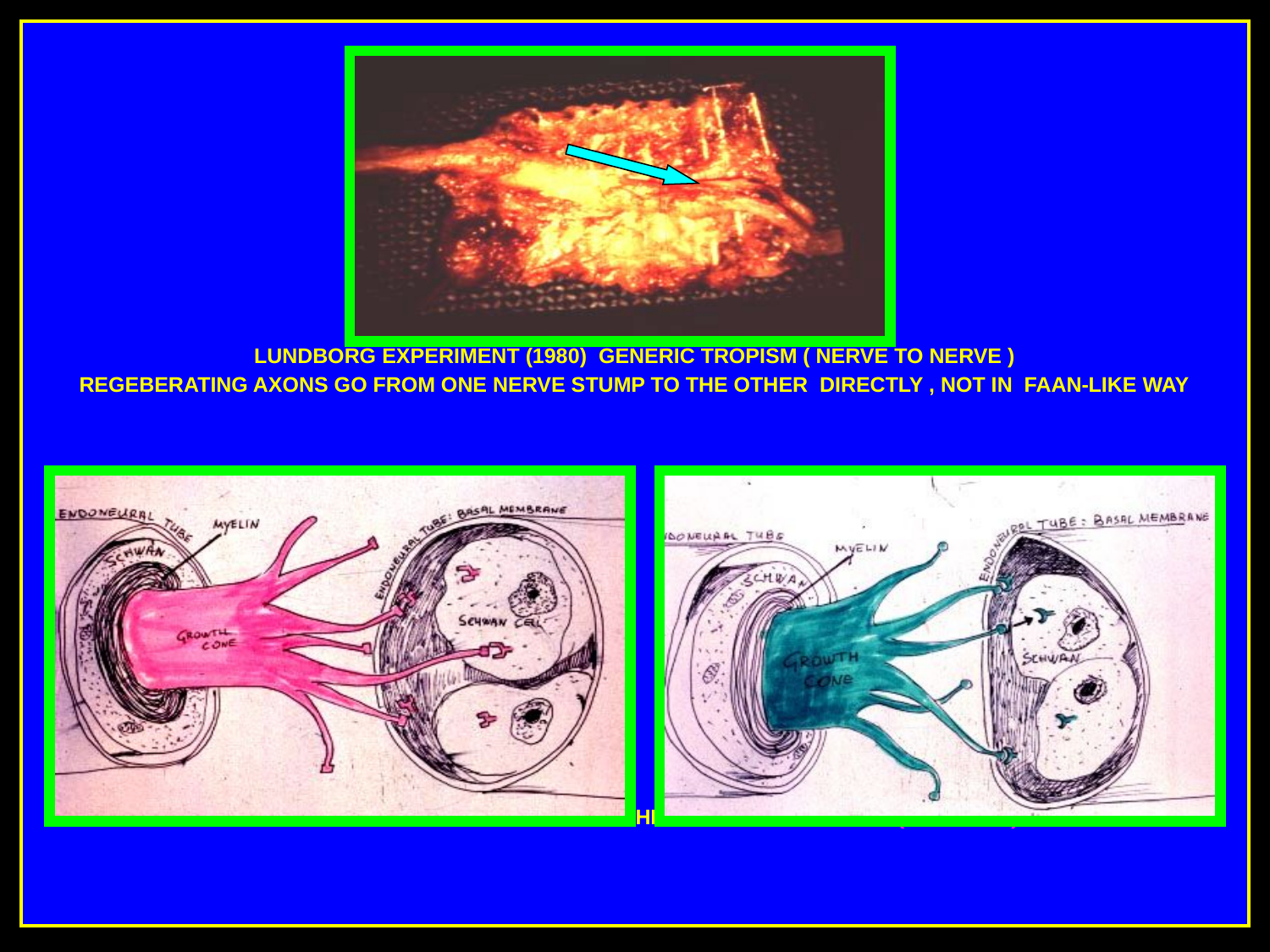

| LUNDBORG EXPERIMENT (1980) GENERIC TROPISM ( NERVE TO NERVE ) REGEBERATING AXONS GO FROM ONE NERVE STUMP TO THE OTHER DIRECTLY , NOT IN FAAN-LIKE WAY SPECIAL MOLECULES EXPLAINING THE SELECTIVE TROPISM (BRUNELLI) |
| --- |

## Slide 14
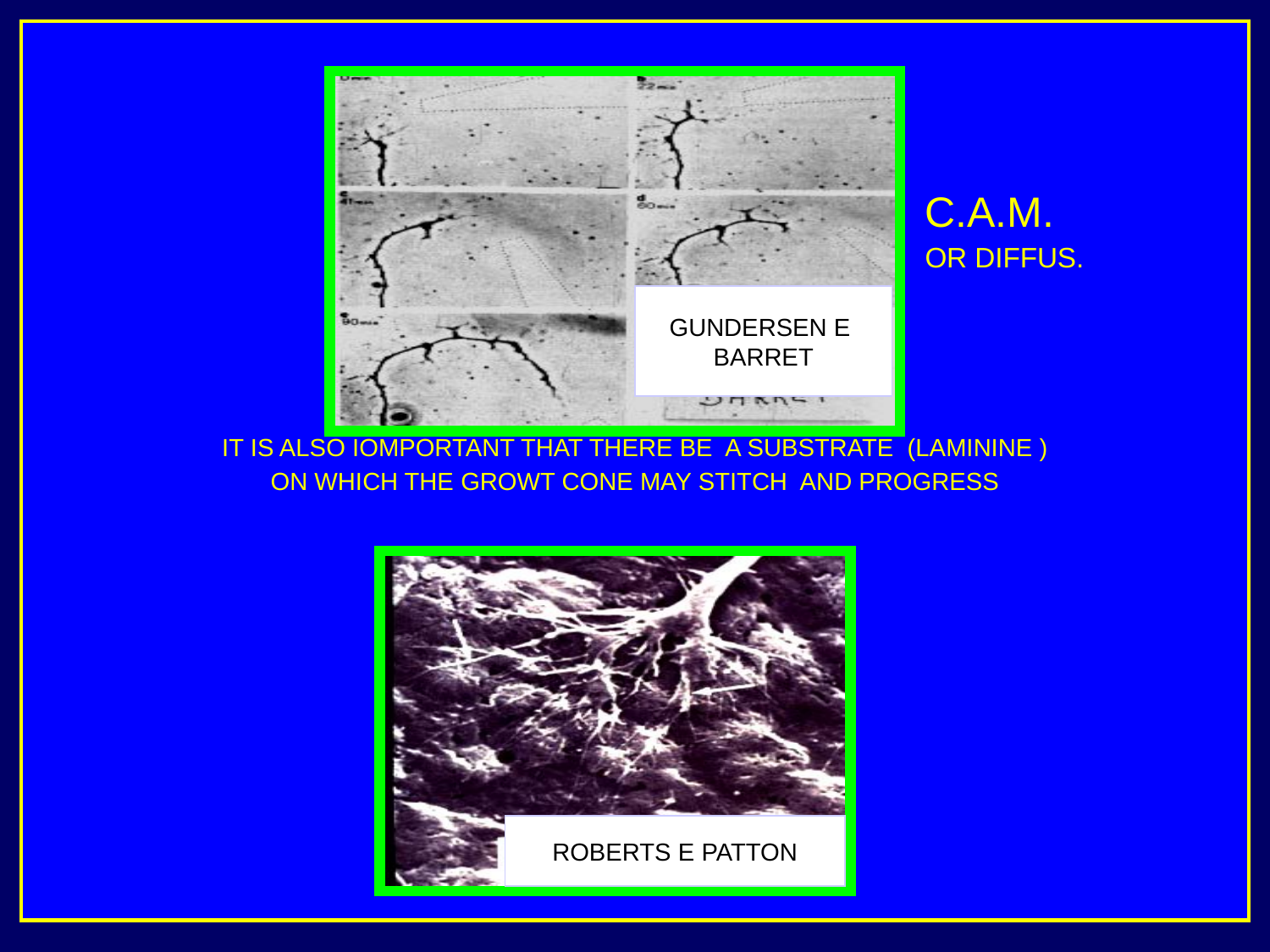

| IT IS ALSO IOMPORTANT THAT THERE BE A SUBSTRATE (LAMININE ) ON WHICH THE GROWT CONE MAY STITCH AND PROGRESS |
| --- |
C.A.M.
OR DIFFUS.
GUNDERSEN E
BARRET
ROBERTS E PATTON

## Slide 15
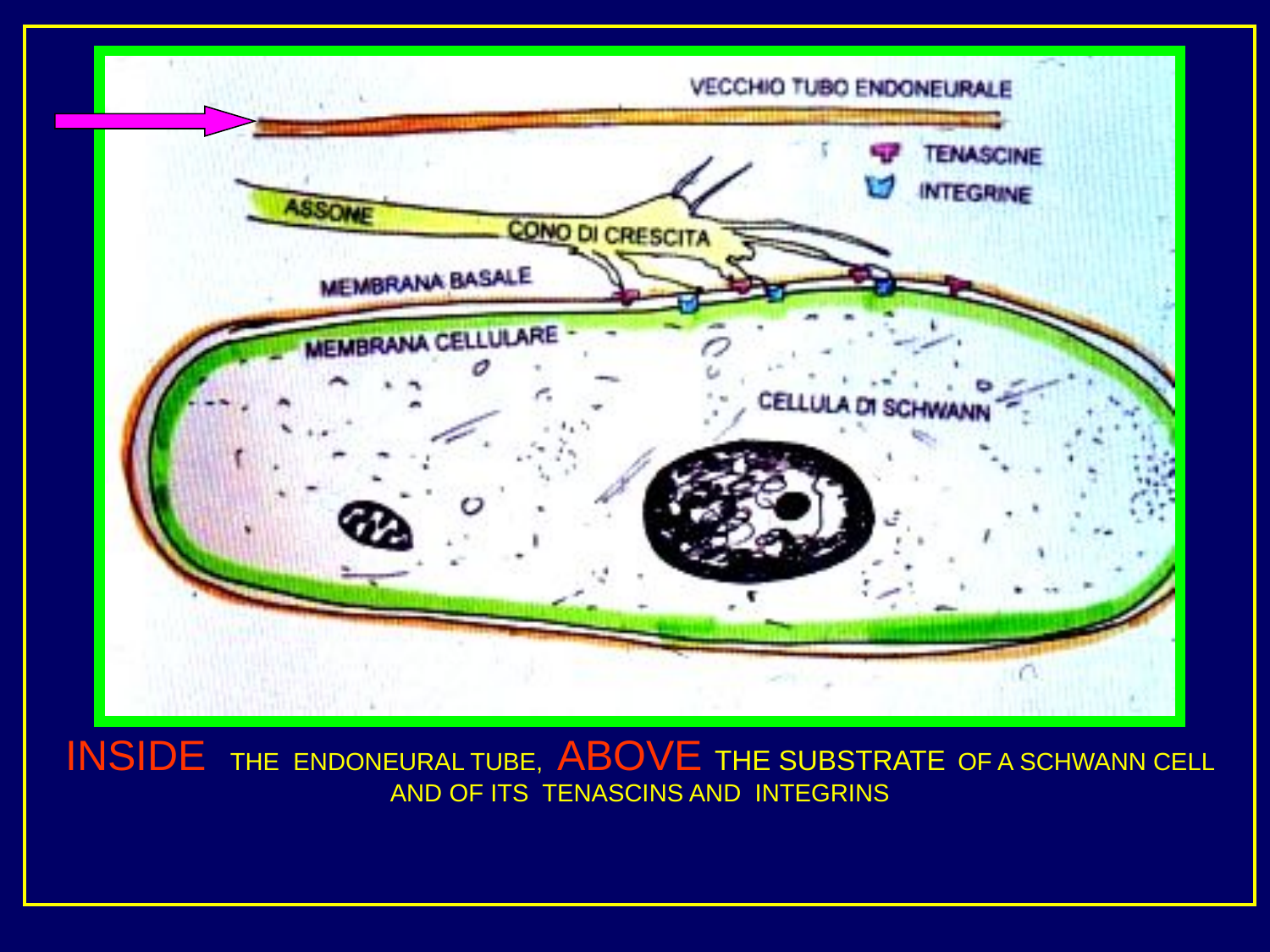

| SCHEME OF THE ADVANCEMENT OF A GROWTH CONE INSIDE THE ENDONEURAL TUBE, ABOVE THE SUBSTRATE OF A SCHWANN CELL AND OF ITS TENASCINS AND INTEGRINS |
| --- |

## Slide 16
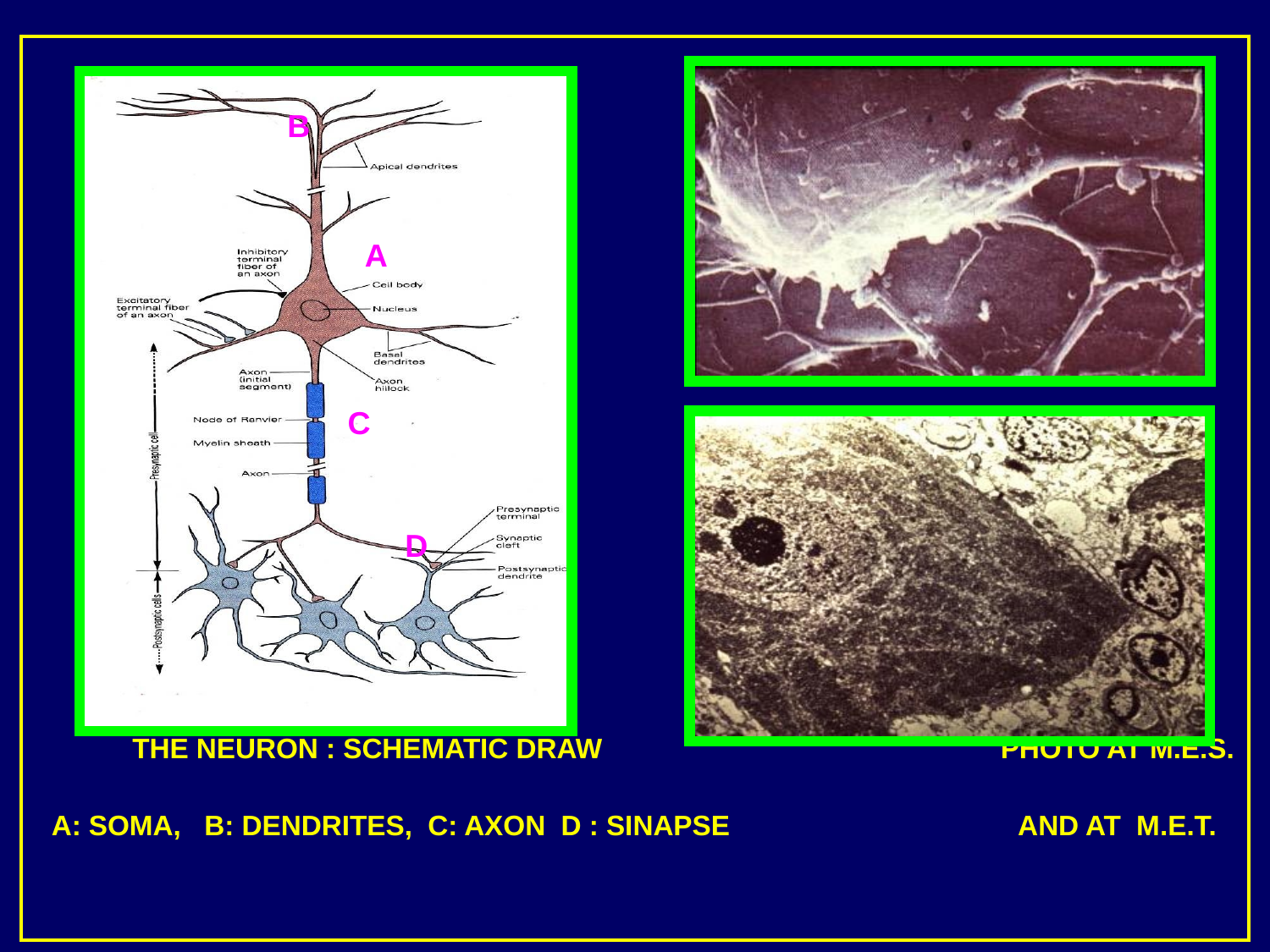

| THE NEURON : SCHEMATIC DRAW PHOTO AT M.E.S. A: SOMA, B: DENDRITES, C: AXON D : SINAPSE AND AT M.E.T. |
| --- |
B
A
C
D

## Slide 17
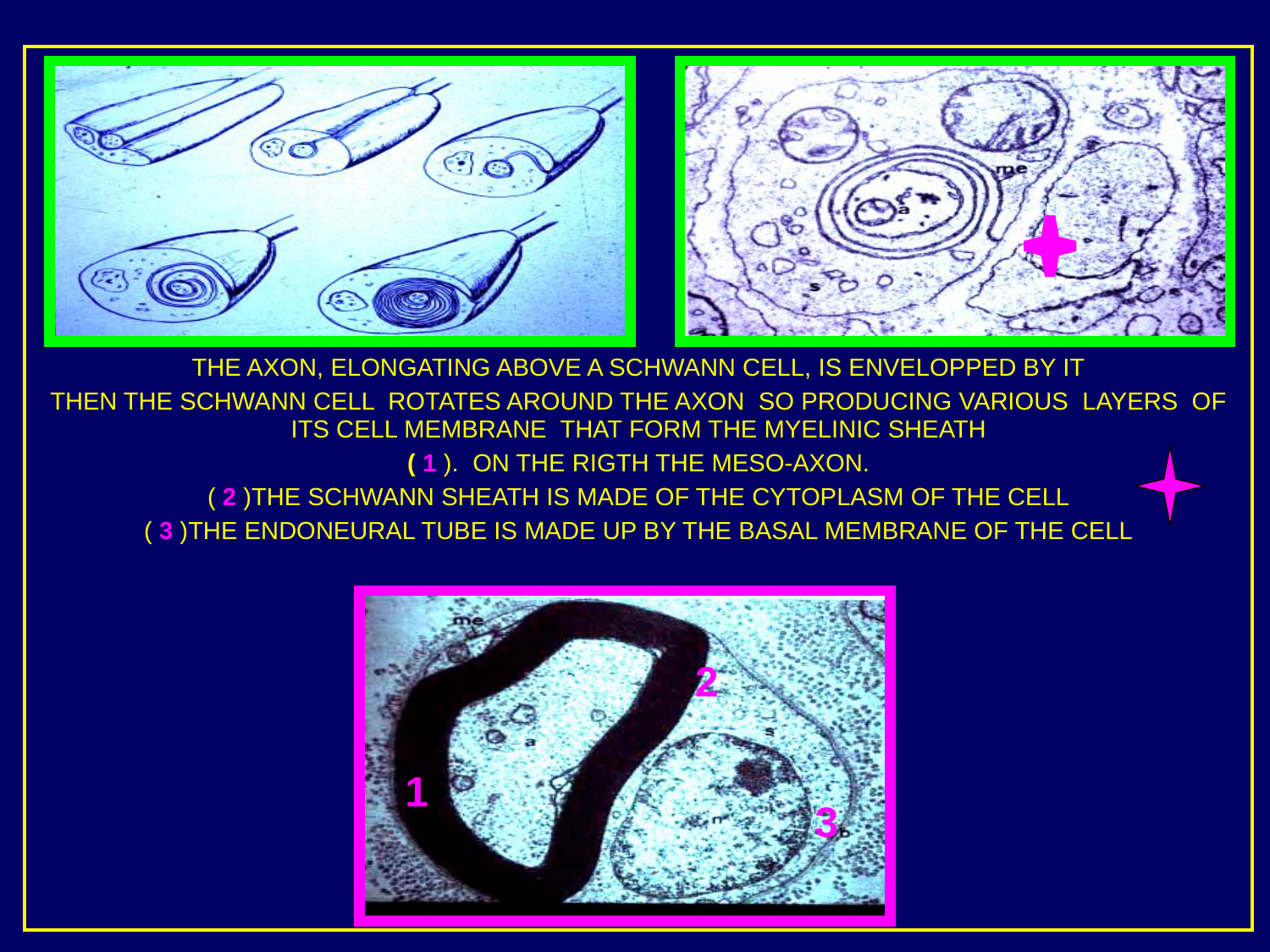

| THE AXON, ELONGATING ABOVE A SCHWANN CELL, IS ENVELOPPED BY IT THEN THE SCHWANN CELL ROTATES AROUND THE AXON SO PRODUCING VARIOUS LAYERS OF ITS CELL MEMBRANE THAT FORM THE MYELINIC SHEATH ( 1 ). ON THE RIGTH THE MESO-AXON. ( 2 )THE SCHWANN SHEATH IS MADE OF THE CYTOPLASM OF THE CELL ( 3 )THE ENDONEURAL TUBE IS MADE UP BY THE BASAL MEMBRANE OF THE CELL |
| --- |
2
1
3

## Slide 18
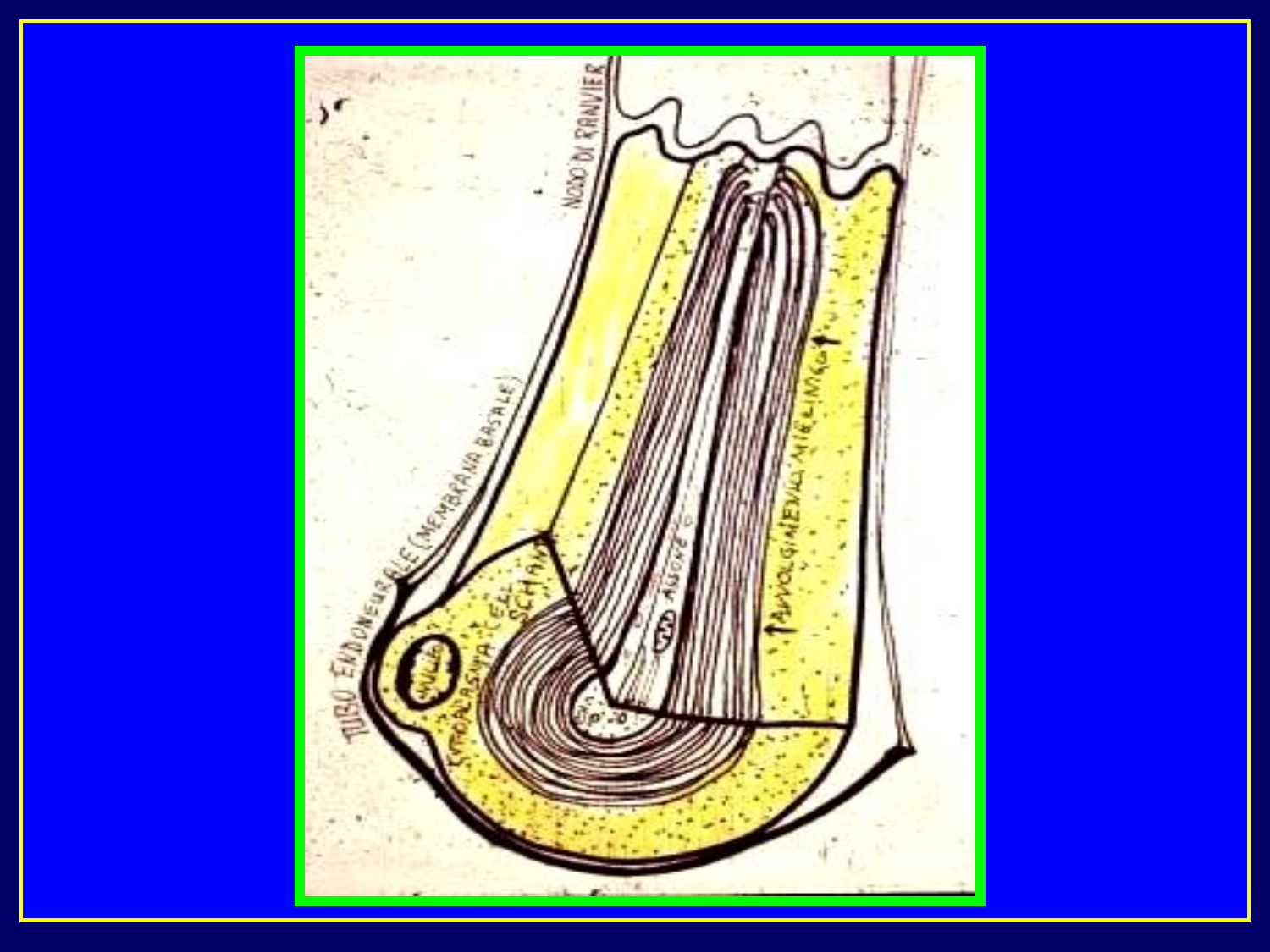

| |
| --- |

## Slide 19
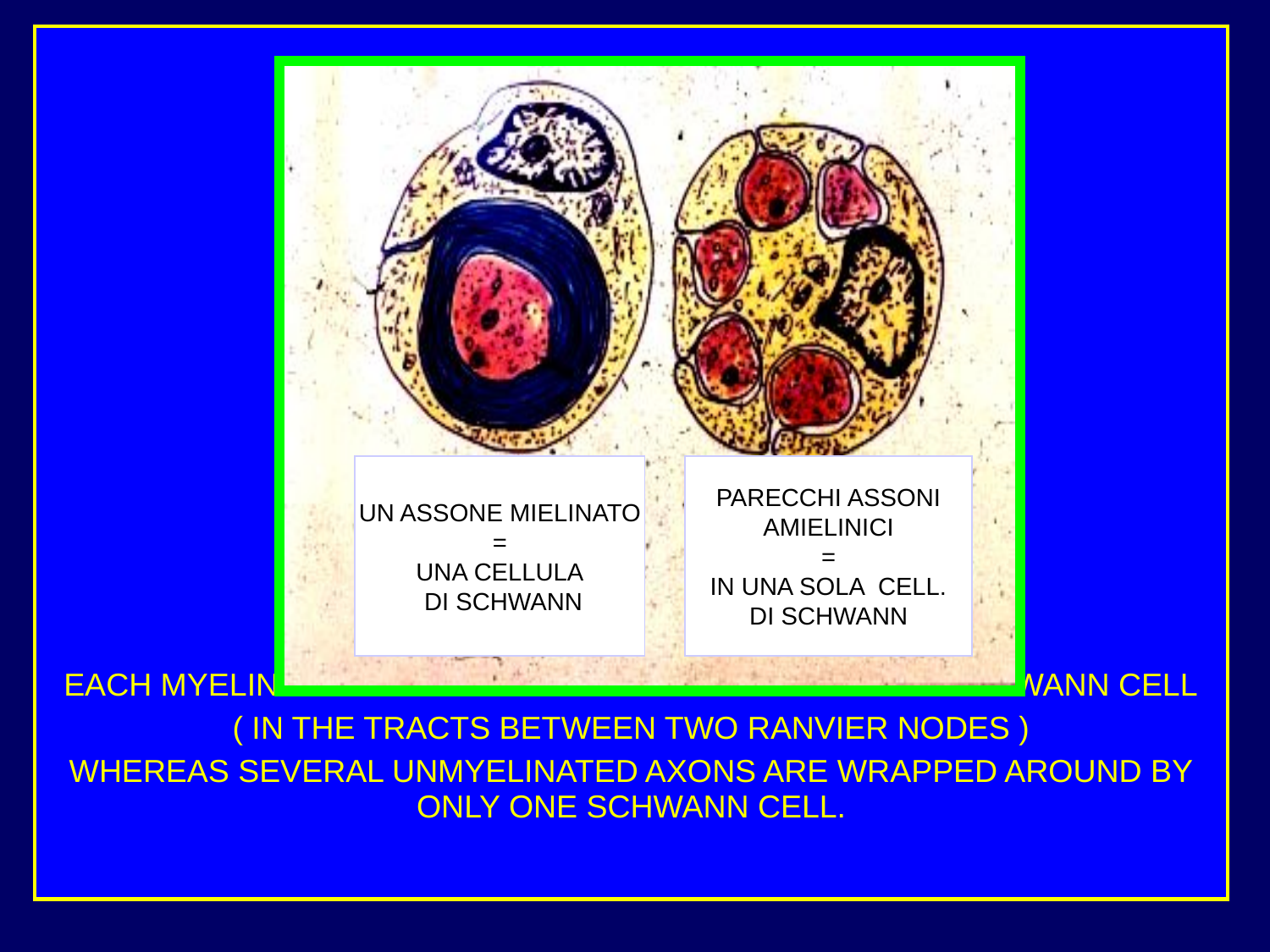

| EACH MYELINATED AXON IS WRAPPED AROUND BY ONE SCHWANN CELL ( IN THE TRACTS BETWEEN TWO RANVIER NODES ) WHEREAS SEVERAL UNMYELINATED AXONS ARE WRAPPED AROUND BY ONLY ONE SCHWANN CELL. |
| --- |
UN ASSONE MIELINATO
=
UNA CELLULA
 DI SCHWANN
PARECCHI ASSONI
AMIELINICI
=
IN UNA SOLA CELL.
DI SCHWANN

## Slide 20
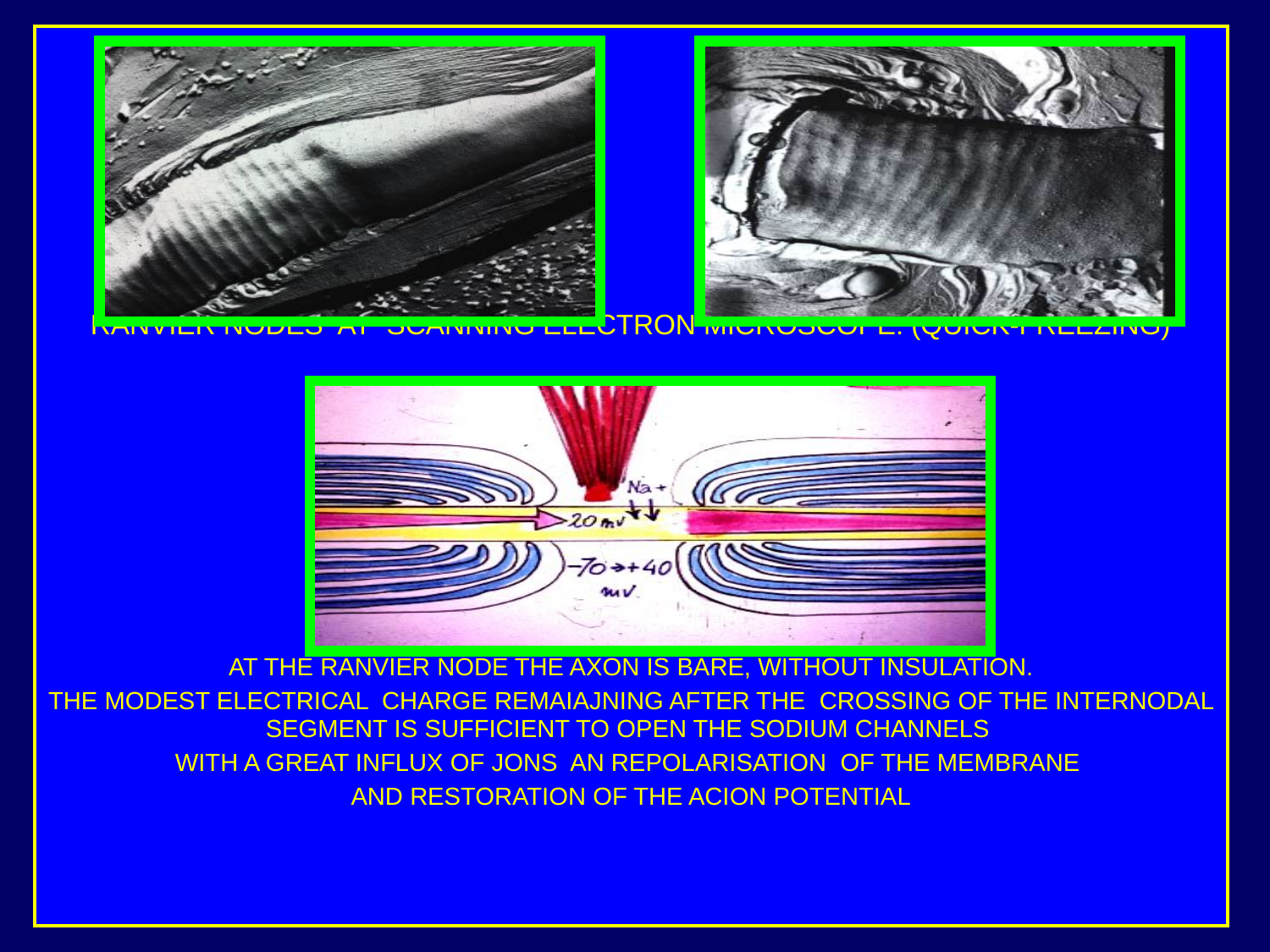

| RANVIER NODES AT SCANNING ELECTRON MICROSCOPE: (QUICK-FREEZING) AT THE RANVIER NODE THE AXON IS BARE, WITHOUT INSULATION. THE MODEST ELECTRICAL CHARGE REMAIAJNING AFTER THE CROSSING OF THE INTERNODAL SEGMENT IS SUFFICIENT TO OPEN THE SODIUM CHANNELS WITH A GREAT INFLUX OF JONS AN REPOLARISATION OF THE MEMBRANE AND RESTORATION OF THE ACION POTENTIAL |
| --- |

## Slide 21
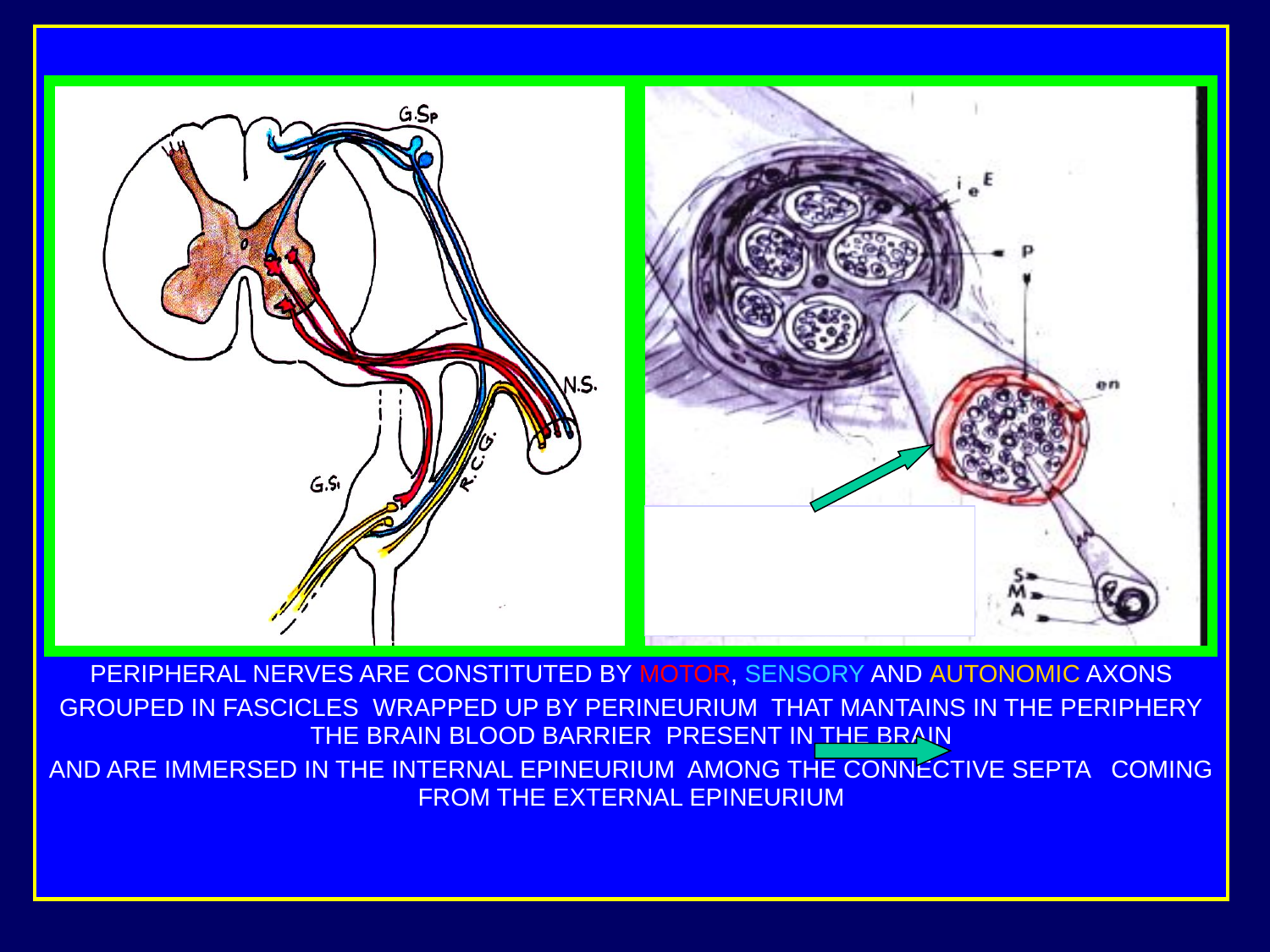

| PERIPHERAL NERVES ARE CONSTITUTED BY MOTOR, SENSORY AND AUTONOMIC AXONS GROUPED IN FASCICLES WRAPPED UP BY PERINEURIUM THAT MANTAINS IN THE PERIPHERY THE BRAIN BLOOD BARRIER PRESENT IN THE BRAIN AND ARE IMMERSED IN THE INTERNAL EPINEURIUM AMONG THE CONNECTIVE SEPTA COMING FROM THE EXTERNAL EPINEURIUM |
| --- |

## Slide 22
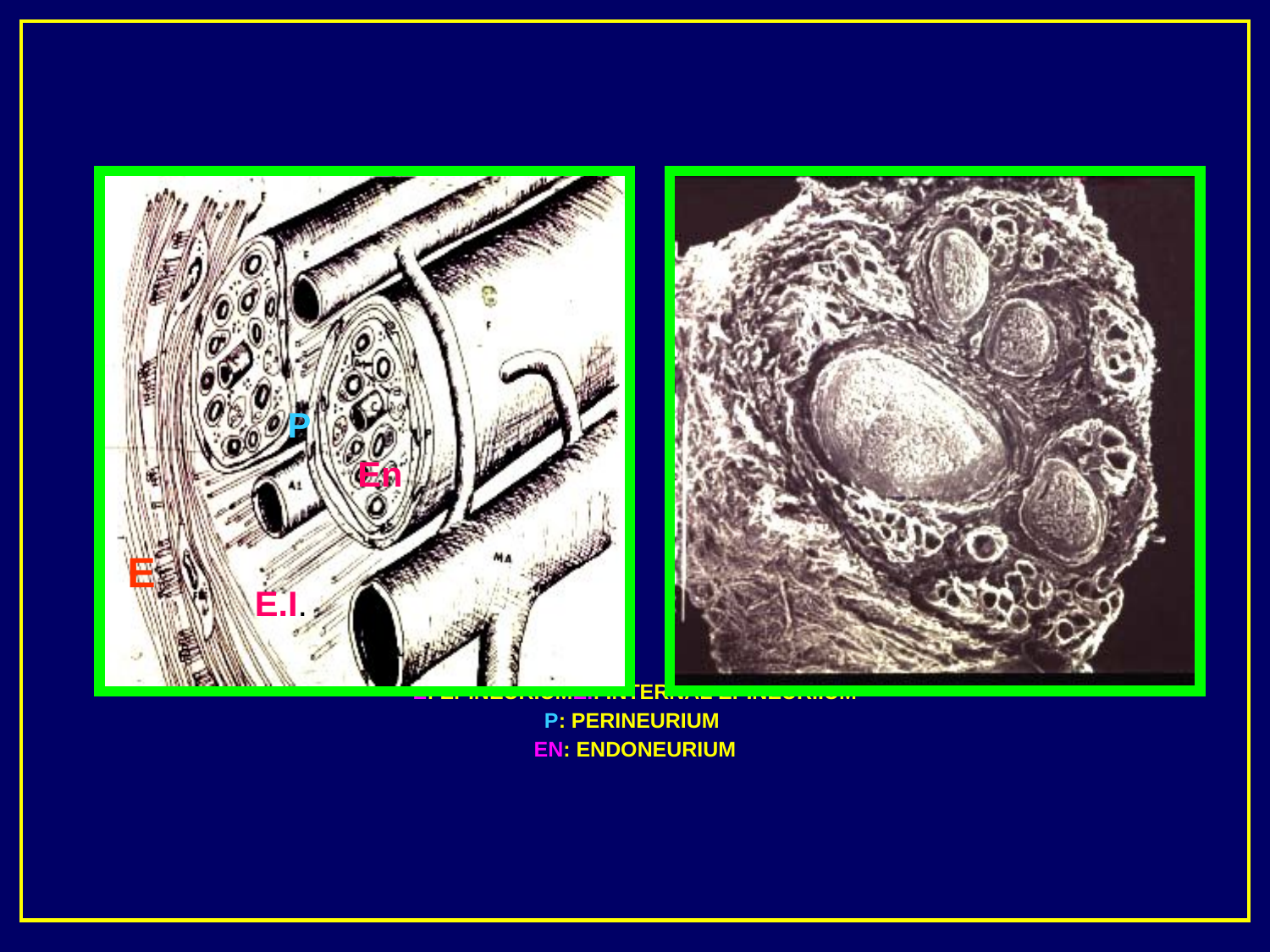

| E: EPINEURIUMEI: INTERNAL EPINEURIIUM P: PERINEURIUM EN: ENDONEURIUM |
| --- |
P
En
E
E.I.

## Slide 23
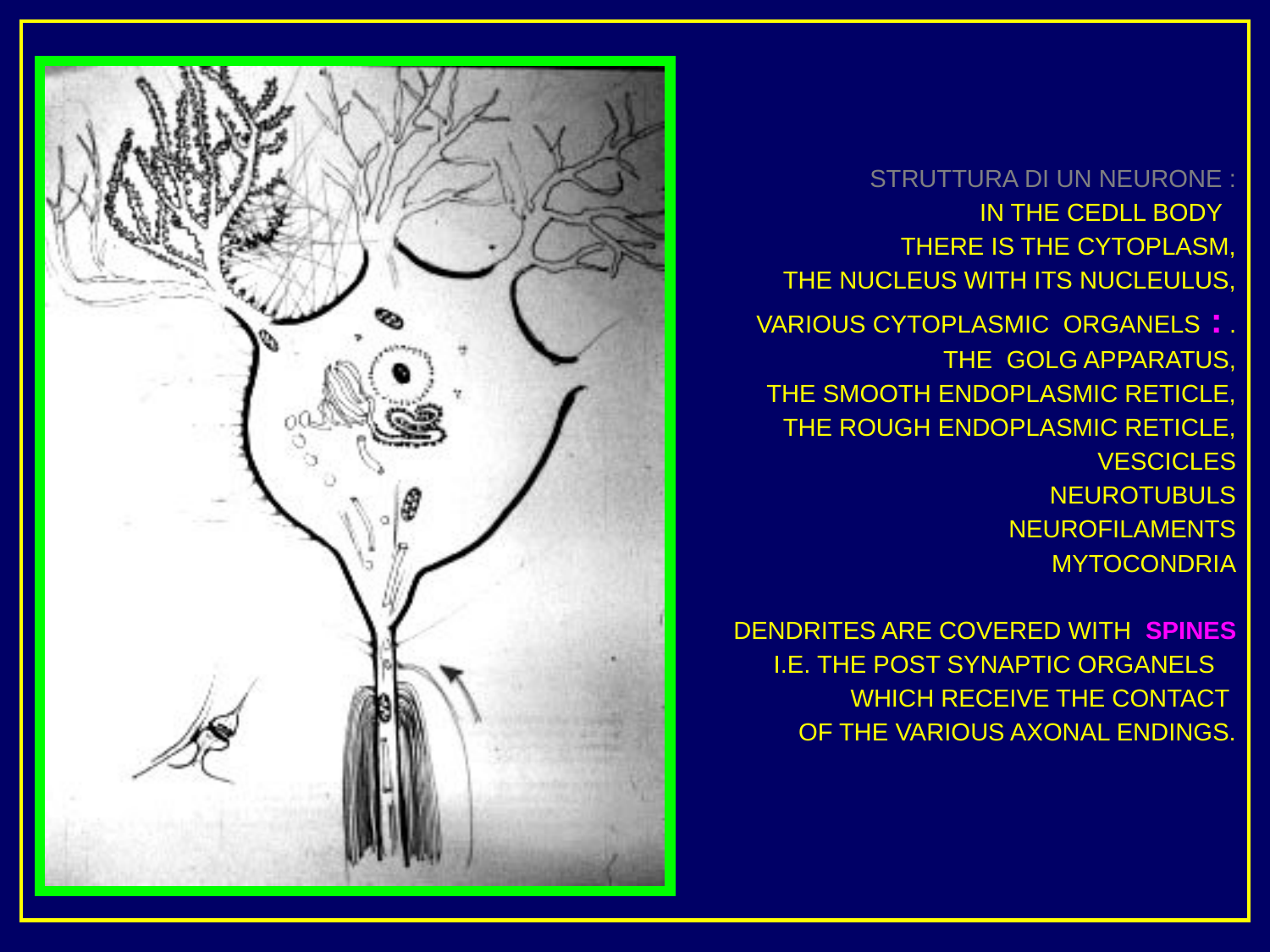

| STRUTTURA DI UN NEURONE : IN THE CEDLL BODY THERE IS THE CYTOPLASM, THE NUCLEUS WITH ITS NUCLEULUS, VARIOUS CYTOPLASMIC ORGANELS : . THE GOLG APPARATUS, THE SMOOTH ENDOPLASMIC RETICLE, THE ROUGH ENDOPLASMIC RETICLE, VESCICLES NEUROTUBULS NEUROFILAMENTS MYTOCONDRIA DENDRITES ARE COVERED WITH SPINES I.E. THE POST SYNAPTIC ORGANELS WHICH RECEIVE THE CONTACT OF THE VARIOUS AXONAL ENDINGS. |
| --- |

## Slide 24
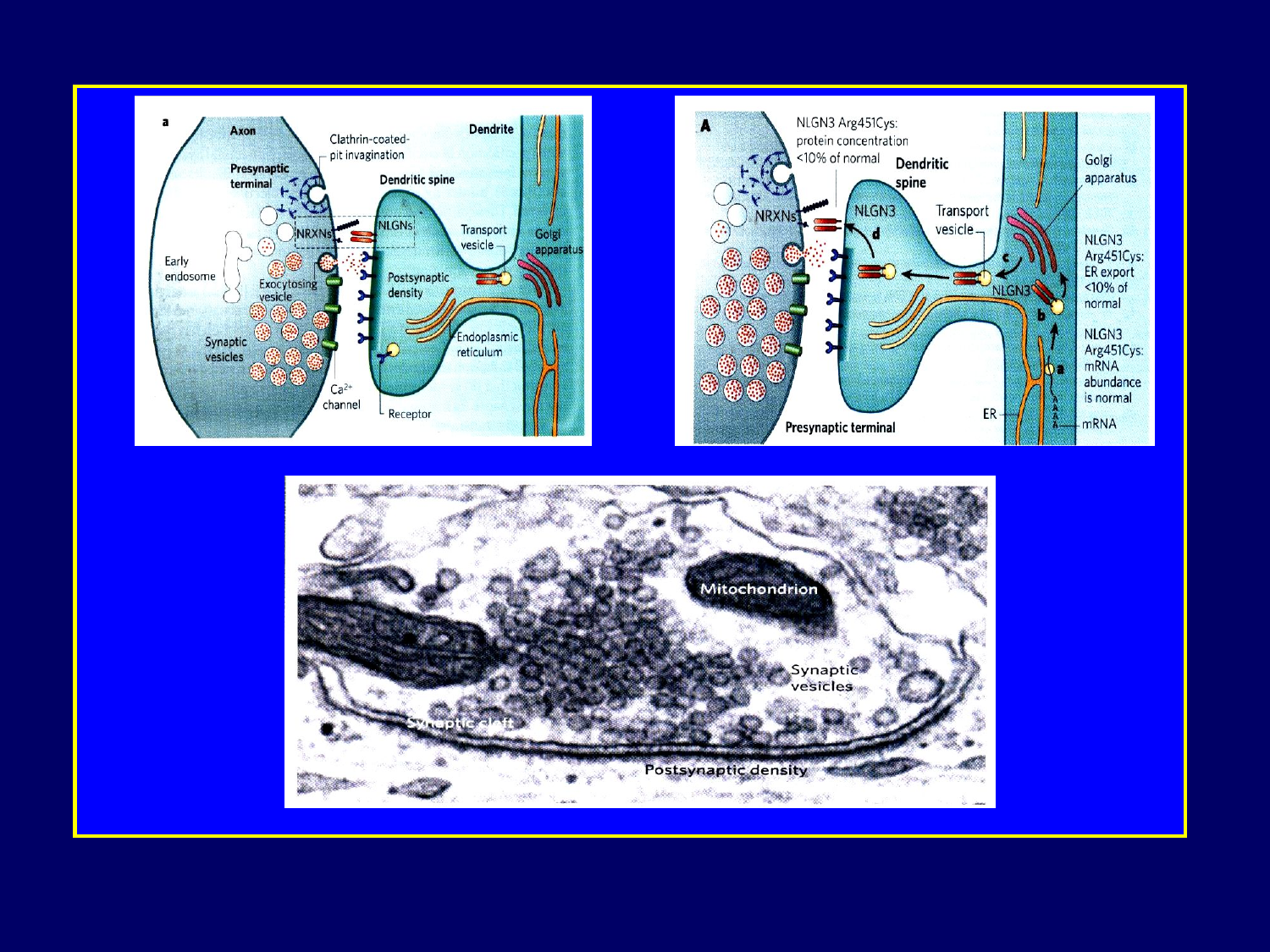

| |
| --- |

## Slide 25
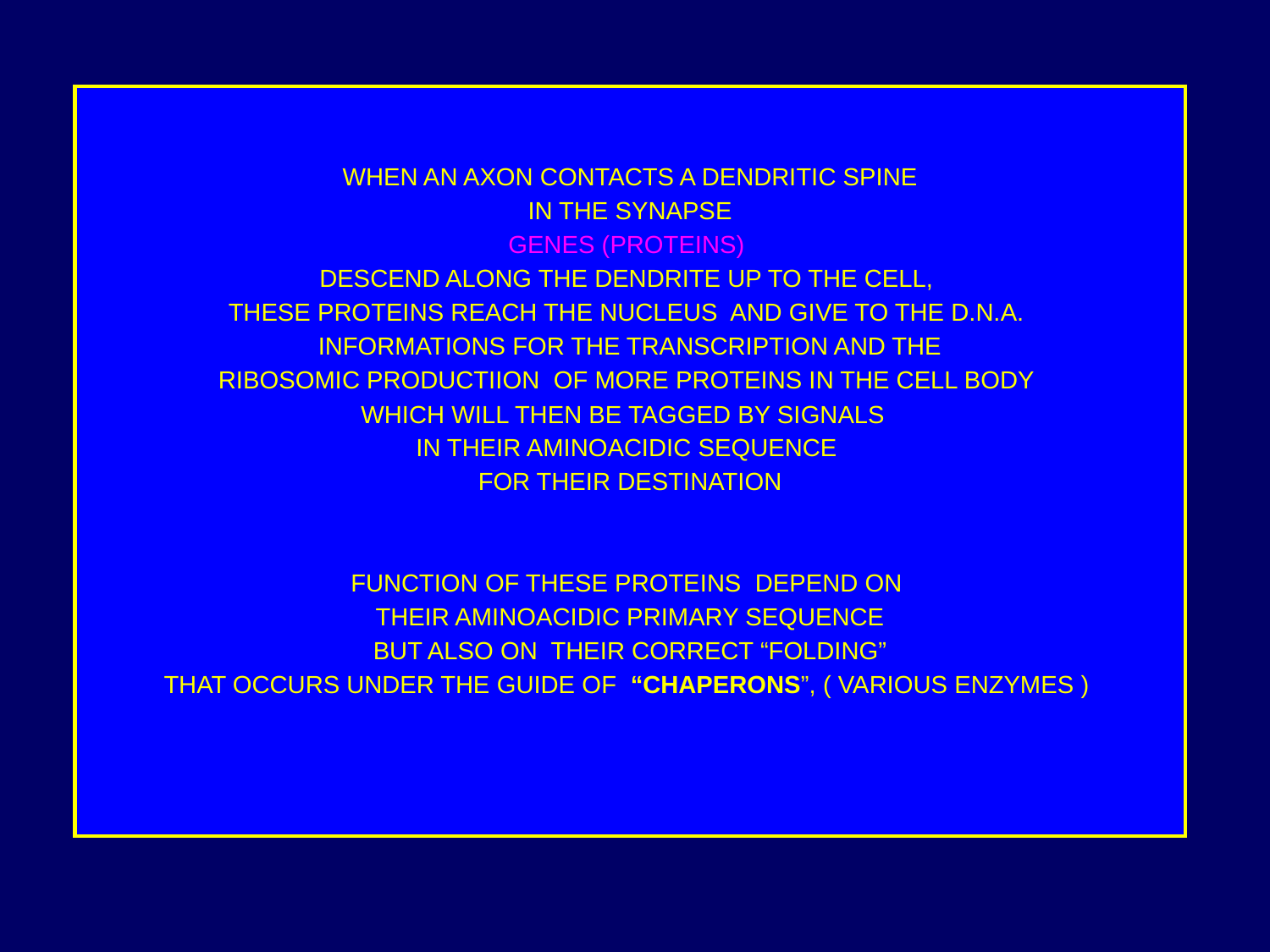

| WHEN AN AXON CONTACTS A DENDRITIC SPINE IN THE SYNAPSE GENES (PROTEINS) DESCEND ALONG THE DENDRITE UP TO THE CELL, THESE PROTEINS REACH THE NUCLEUS AND GIVE TO THE D.N.A. INFORMATIONS FOR THE TRANSCRIPTION AND THE RIBOSOMIC PRODUCTIION OF MORE PROTEINS IN THE CELL BODY WHICH WILL THEN BE TAGGED BY SIGNALS IN THEIR AMINOACIDIC SEQUENCE FOR THEIR DESTINATION FUNCTION OF THESE PROTEINS DEPEND ON THEIR AMINOACIDIC PRIMARY SEQUENCE BUT ALSO ON THEIR CORRECT “FOLDING” THAT OCCURS UNDER THE GUIDE OF “CHAPERONS”, ( VARIOUS ENZYMES ) |
| --- |

## Slide 26
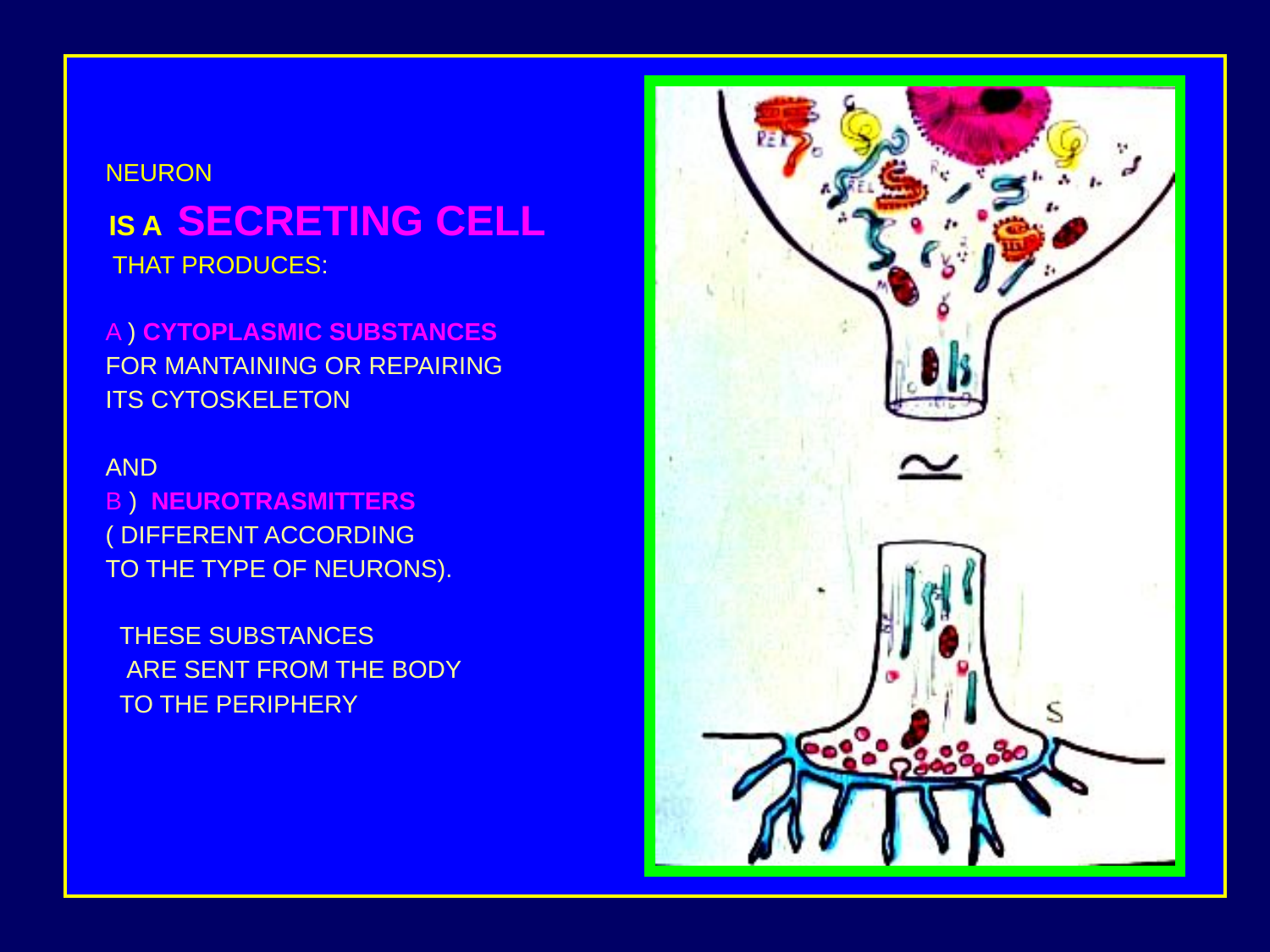

| NEURON IS A SECRETING CELL THAT PRODUCES: A ) CYTOPLASMIC SUBSTANCES FOR MANTAINING OR REPAIRING ITS CYTOSKELETON AND B ) NEUROTRASMITTERS ( DIFFERENT ACCORDING TO THE TYPE OF NEURONS). THESE SUBSTANCES ARE SENT FROM THE BODY TO THE PERIPHERY |
| --- |

## Slide 27
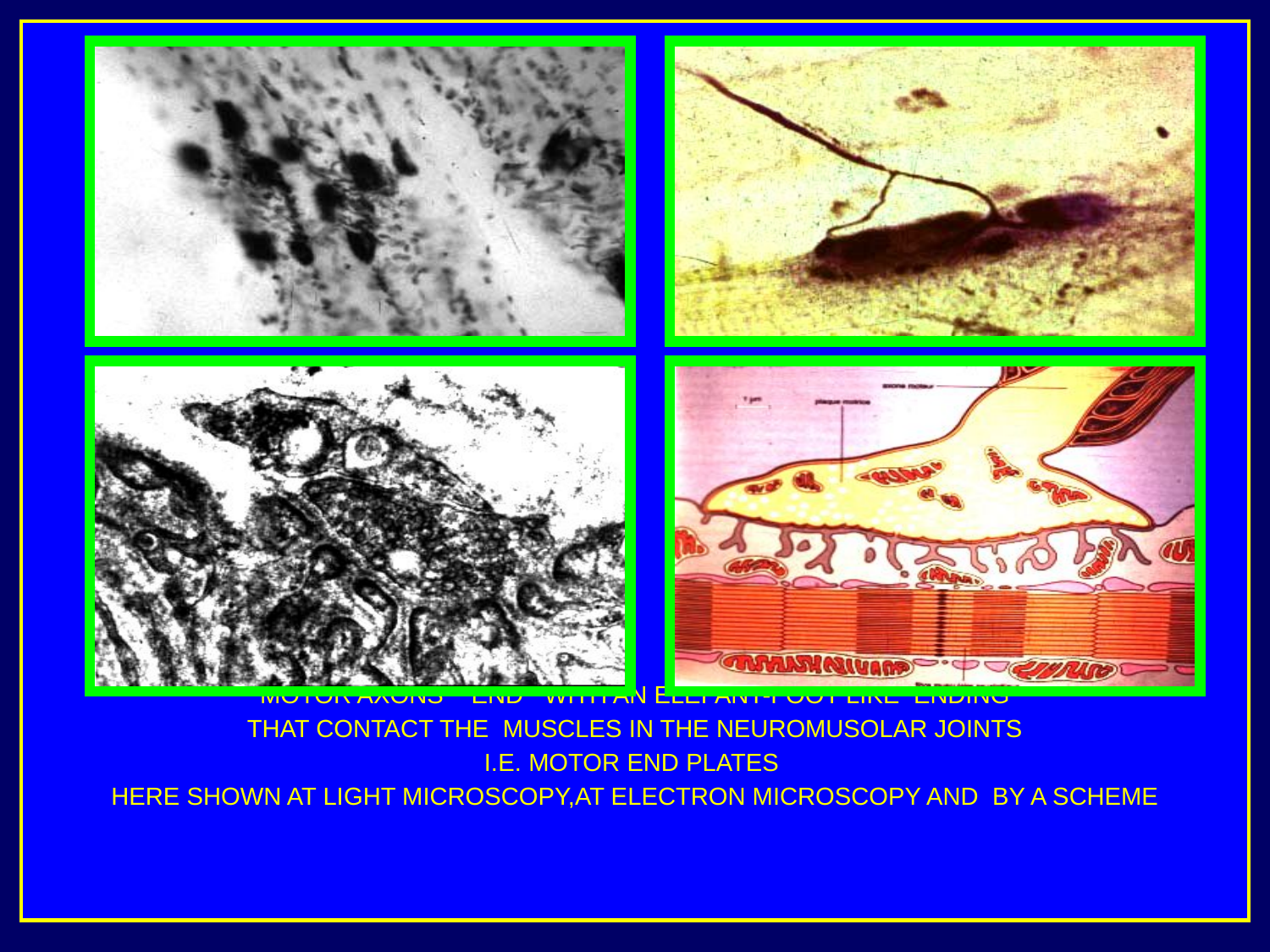

| MOTOR AXONS END WITH AN ELEFANT-FOOT LIKE ENDING THAT CONTACT THE MUSCLES IN THE NEUROMUSOLAR JOINTS I.E. MOTOR END PLATES HERE SHOWN AT LIGHT MICROSCOPY,AT ELECTRON MICROSCOPY AND BY A SCHEME |
| --- |

## Slide 28
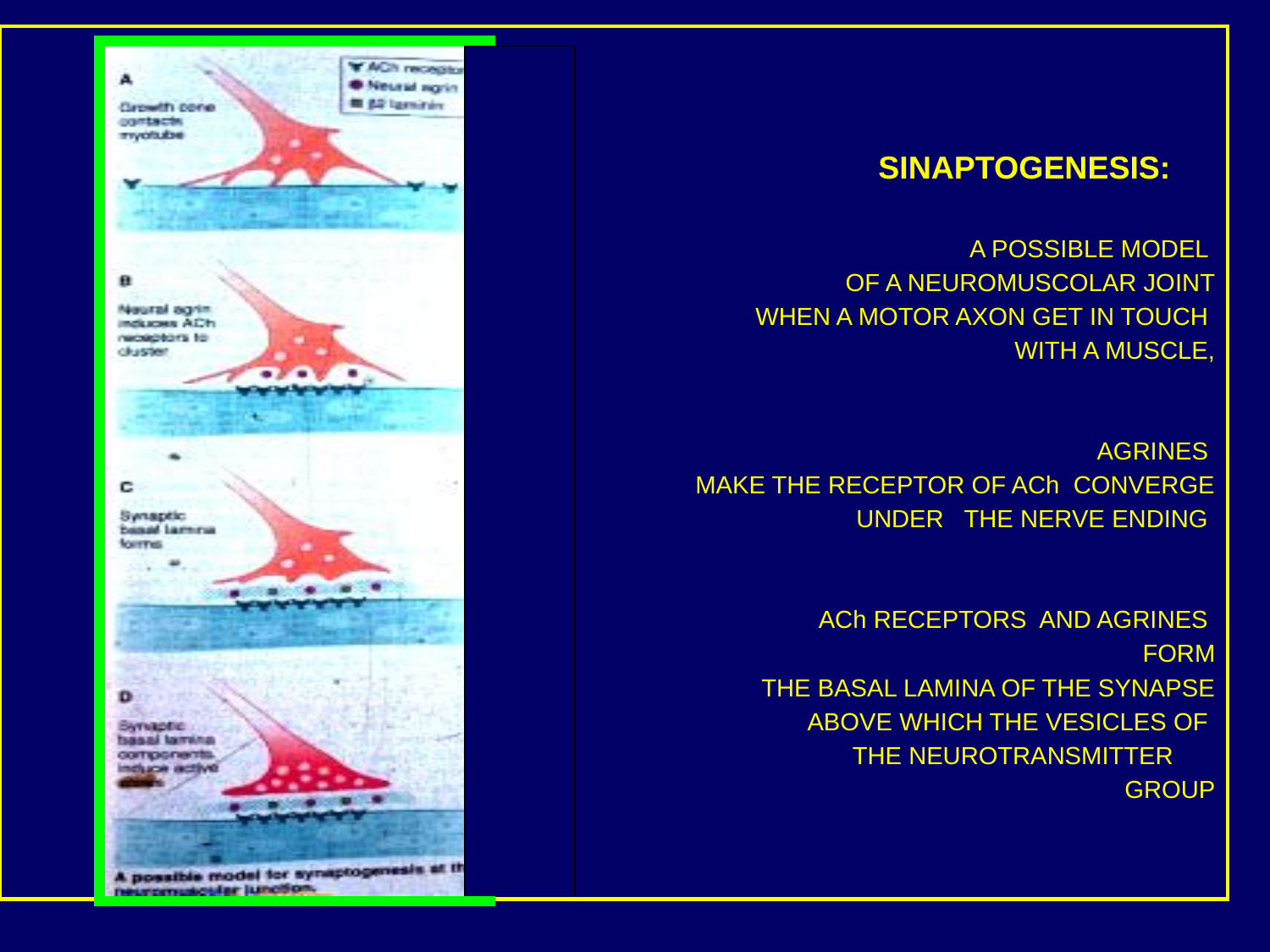

| SINAPTOGENESIS: A POSSIBLE MODEL OF A NEUROMUSCOLAR JOINT WHEN A MOTOR AXON GET IN TOUCH WITH A MUSCLE, AGRINES MAKE THE RECEPTOR OF ACh CONVERGE UNDER THE NERVE ENDING ACh RECEPTORS AND AGRINES FORM THE BASAL LAMINA OF THE SYNAPSE ABOVE WHICH THE VESICLES OF THE NEUROTRANSMITTER GROUP |
| --- |

## Slide 29
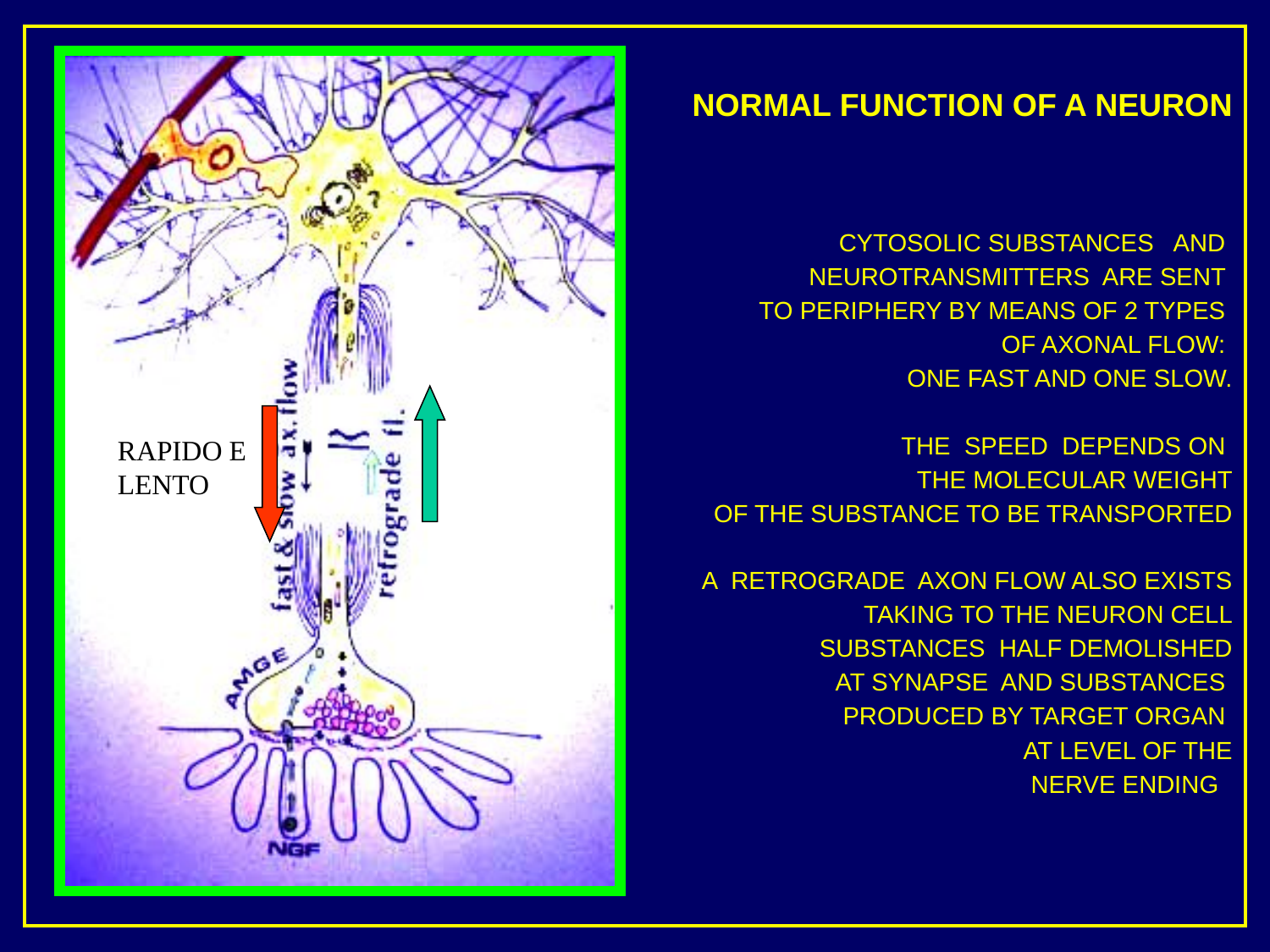

| NORMAL FUNCTION OF A NEURON CYTOSOLIC SUBSTANCES AND NEUROTRANSMITTERS ARE SENT TO PERIPHERY BY MEANS OF 2 TYPES OF AXONAL FLOW: ONE FAST AND ONE SLOW. THE SPEED DEPENDS ON THE MOLECULAR WEIGHT OF THE SUBSTANCE TO BE TRANSPORTED A RETROGRADE AXON FLOW ALSO EXISTS TAKING TO THE NEURON CELL SUBSTANCES HALF DEMOLISHED AT SYNAPSE AND SUBSTANCES PRODUCED BY TARGET ORGAN AT LEVEL OF THE NERVE ENDING |
| --- |
RAPIDO E
LENTO

## Slide 30
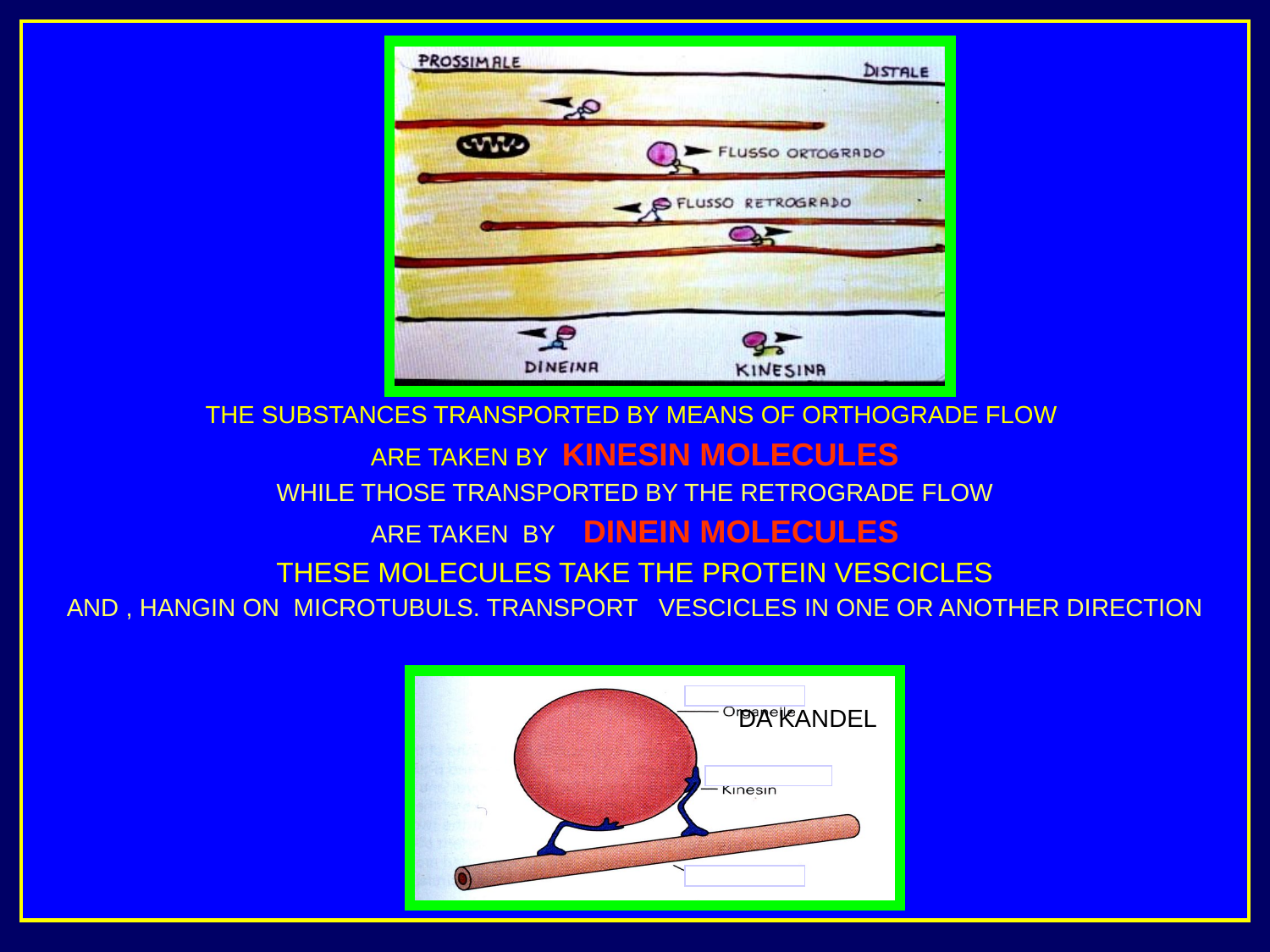

| THE SUBSTANCES TRANSPORTED BY MEANS OF ORTHOGRADE FLOW ARE TAKEN BY KINESIN MOLECULES WHILE THOSE TRANSPORTED BY THE RETROGRADE FLOW ARE TAKEN BY DINEIN MOLECULES THESE MOLECULES TAKE THE PROTEIN VESCICLES AND , HANGIN ON MICROTUBULS. TRANSPORT VESCICLES IN ONE OR ANOTHER DIRECTION |
| --- |
DA KANDEL

## Slide 31
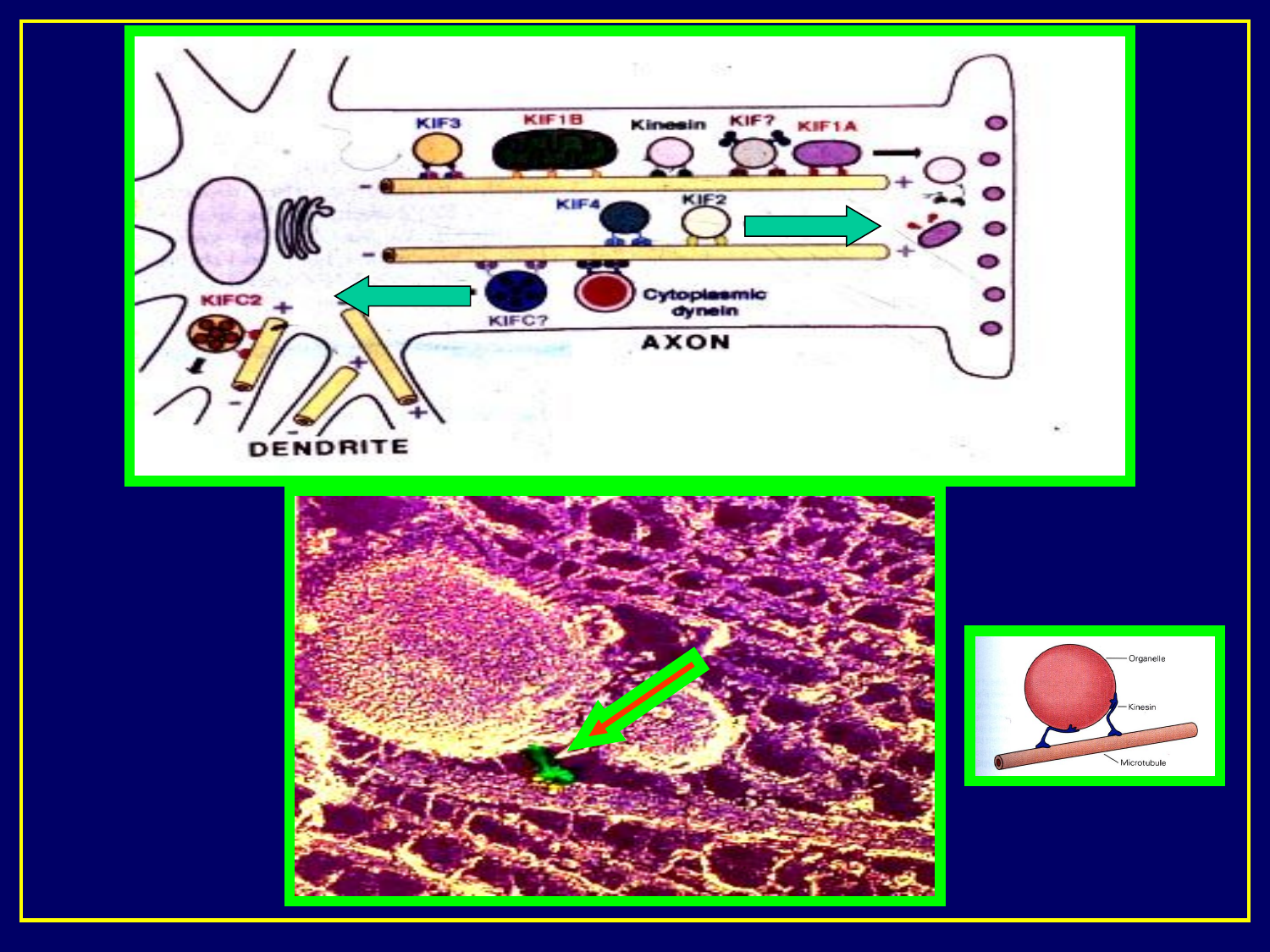

| |
| --- |

## Slide 32
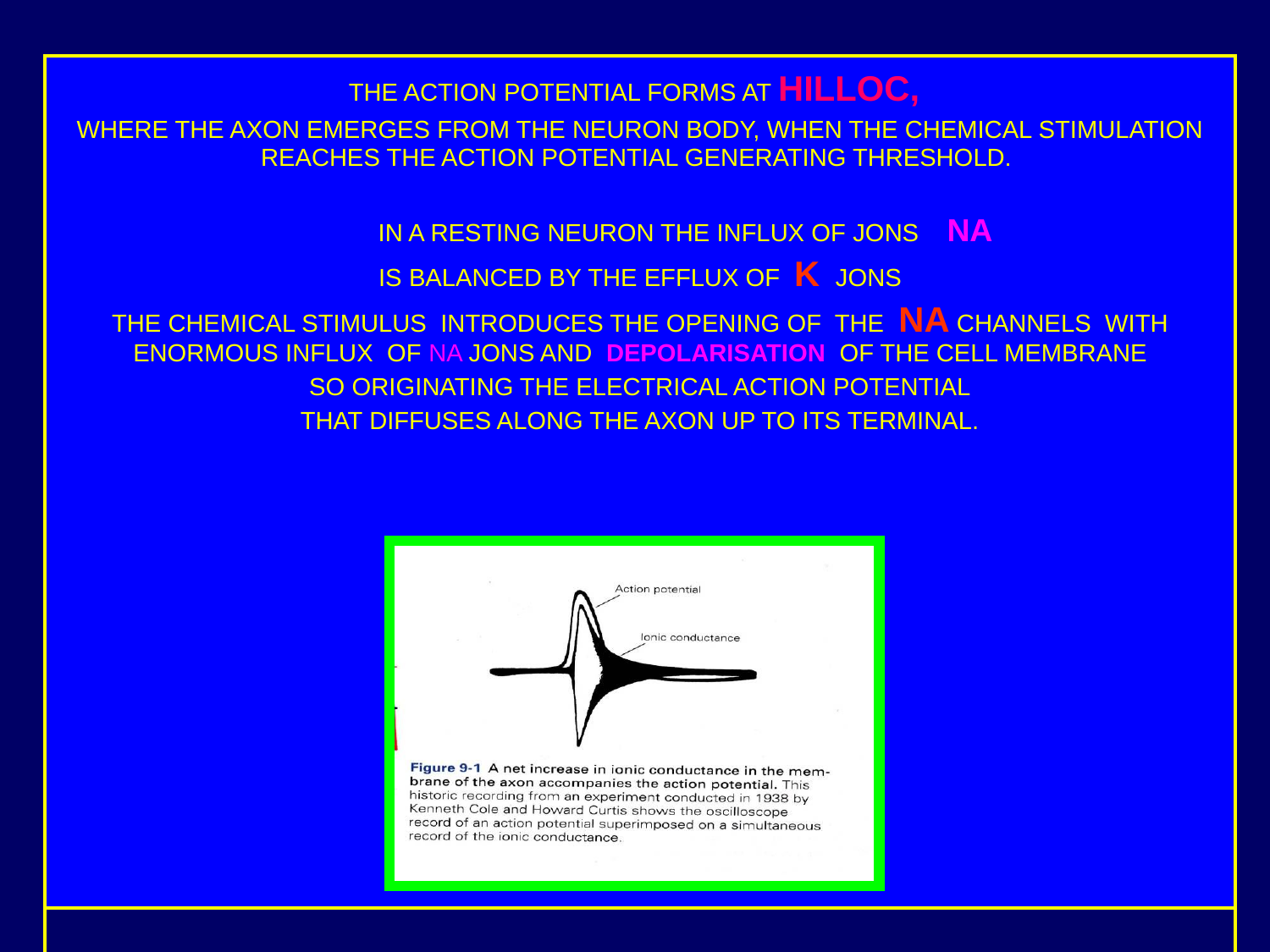

| THE ACTION POTENTIAL FORMS AT HILLOC, WHERE THE AXON EMERGES FROM THE NEURON BODY, WHEN THE CHEMICAL STIMULATION REACHES THE ACTION POTENTIAL GENERATING THRESHOLD. IN A RESTING NEURON THE INFLUX OF JONS NA IS BALANCED BY THE EFFLUX OF K JONS THE CHEMICAL STIMULUS INTRODUCES THE OPENING OF THE NA CHANNELS WITH ENORMOUS INFLUX OF NA JONS AND DEPOLARISATION OF THE CELL MEMBRANE SO ORIGINATING THE ELECTRICAL ACTION POTENTIAL THAT DIFFUSES ALONG THE AXON UP TO ITS TERMINAL. |
| --- |
| |

## Slide 33
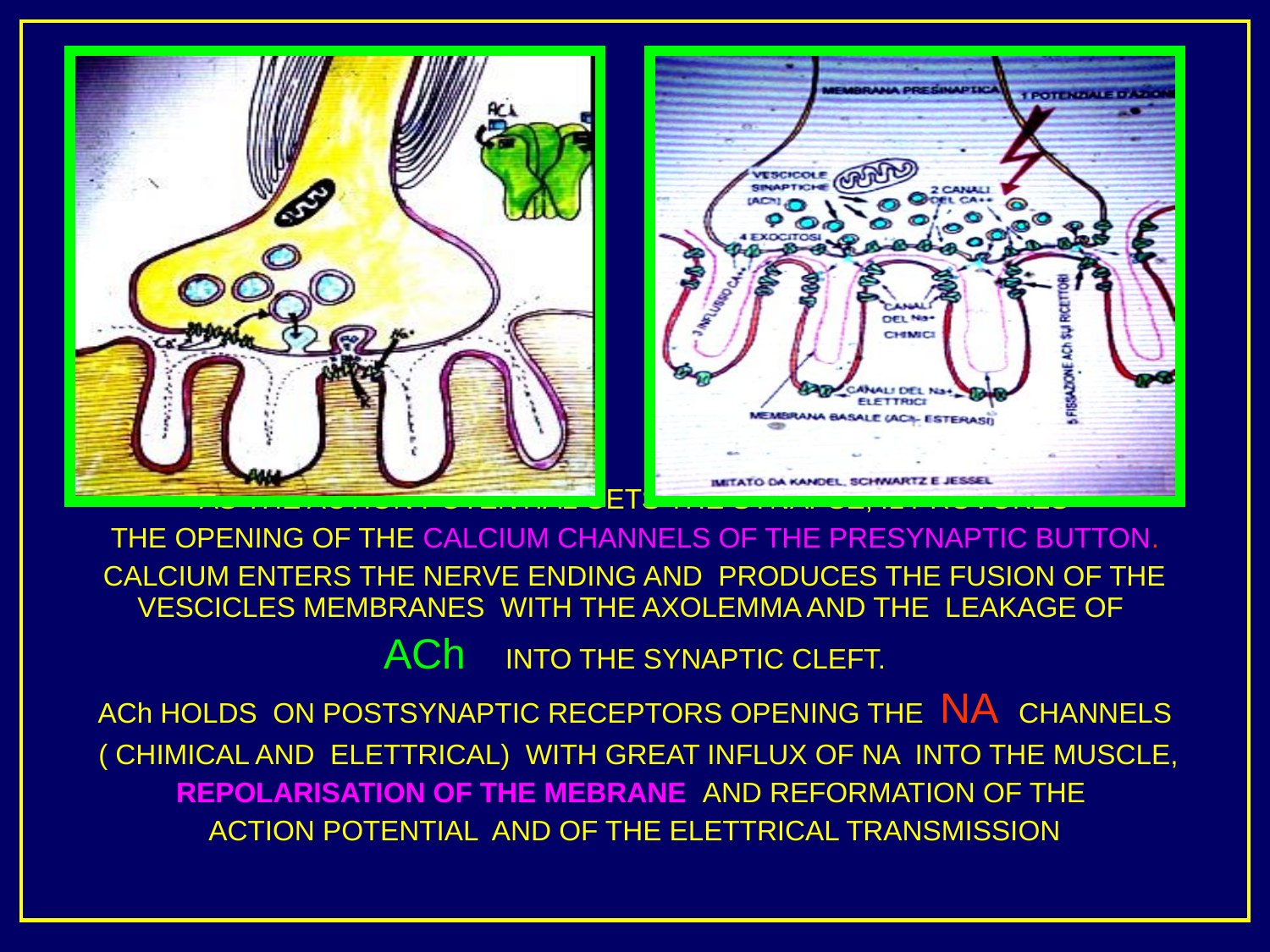

| AS THE ACTION POTENTIAL GETS THE SYNAPSE, IL PROVOKES THE OPENING OF THE CALCIUM CHANNELS OF THE PRESYNAPTIC BUTTON. CALCIUM ENTERS THE NERVE ENDING AND PRODUCES THE FUSION OF THE VESCICLES MEMBRANES WITH THE AXOLEMMA AND THE LEAKAGE OF ACh INTO THE SYNAPTIC CLEFT. ACh HOLDS ON POSTSYNAPTIC RECEPTORS OPENING THE NA CHANNELS ( CHIMICAL AND ELETTRICAL) WITH GREAT INFLUX OF NA INTO THE MUSCLE, REPOLARISATION OF THE MEBRANE AND REFORMATION OF THE ACTION POTENTIAL AND OF THE ELETTRICAL TRANSMISSION |
| --- |

## Slide 34
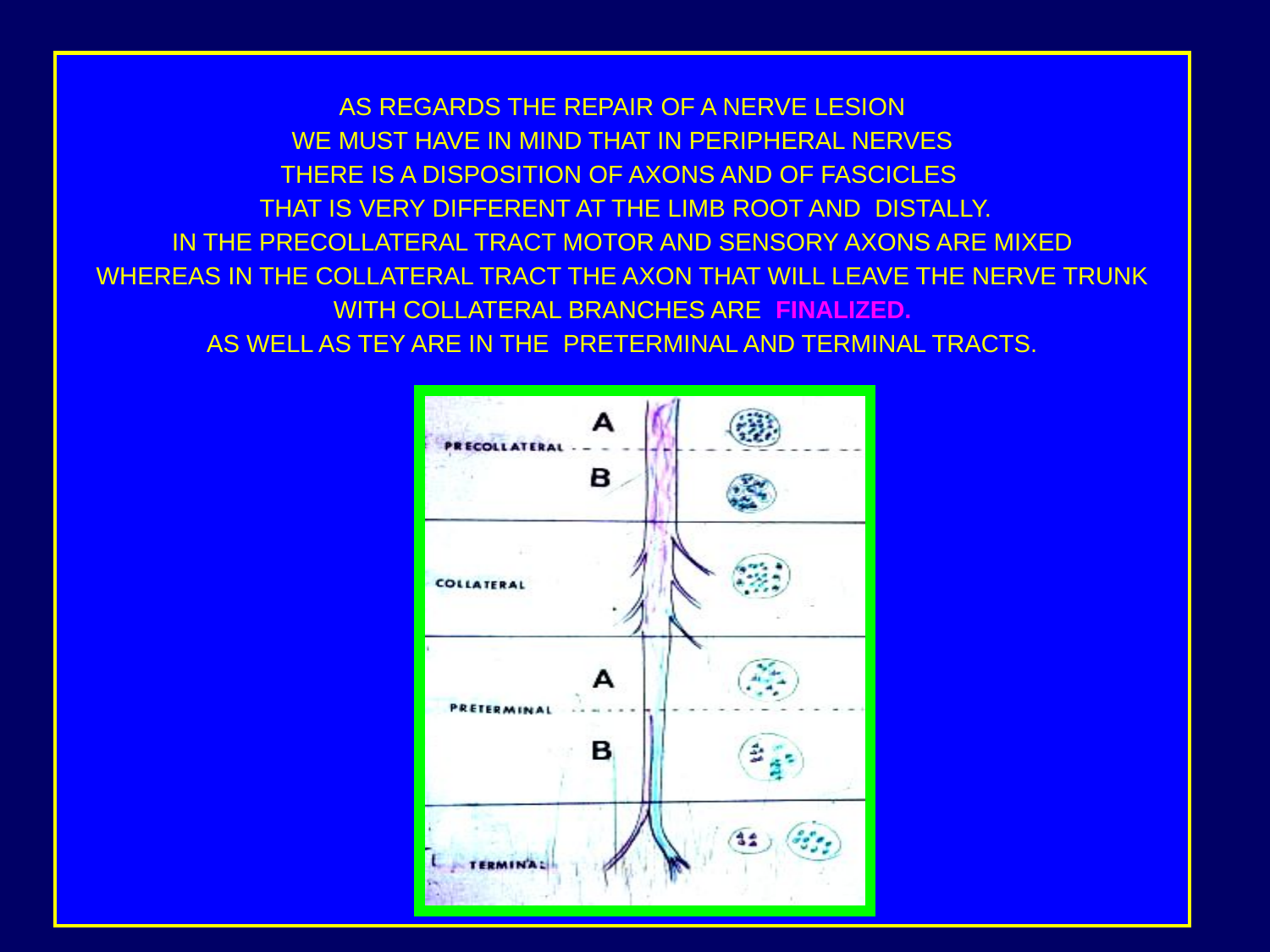

| AS REGARDS THE REPAIR OF A NERVE LESION WE MUST HAVE IN MIND THAT IN PERIPHERAL NERVES THERE IS A DISPOSITION OF AXONS AND OF FASCICLES THAT IS VERY DIFFERENT AT THE LIMB ROOT AND DISTALLY. IN THE PRECOLLATERAL TRACT MOTOR AND SENSORY AXONS ARE MIXED WHEREAS IN THE COLLATERAL TRACT THE AXON THAT WILL LEAVE THE NERVE TRUNK WITH COLLATERAL BRANCHES ARE FINALIZED. AS WELL AS TEY ARE IN THE PRETERMINAL AND TERMINAL TRACTS. |
| --- |

## Slide 35
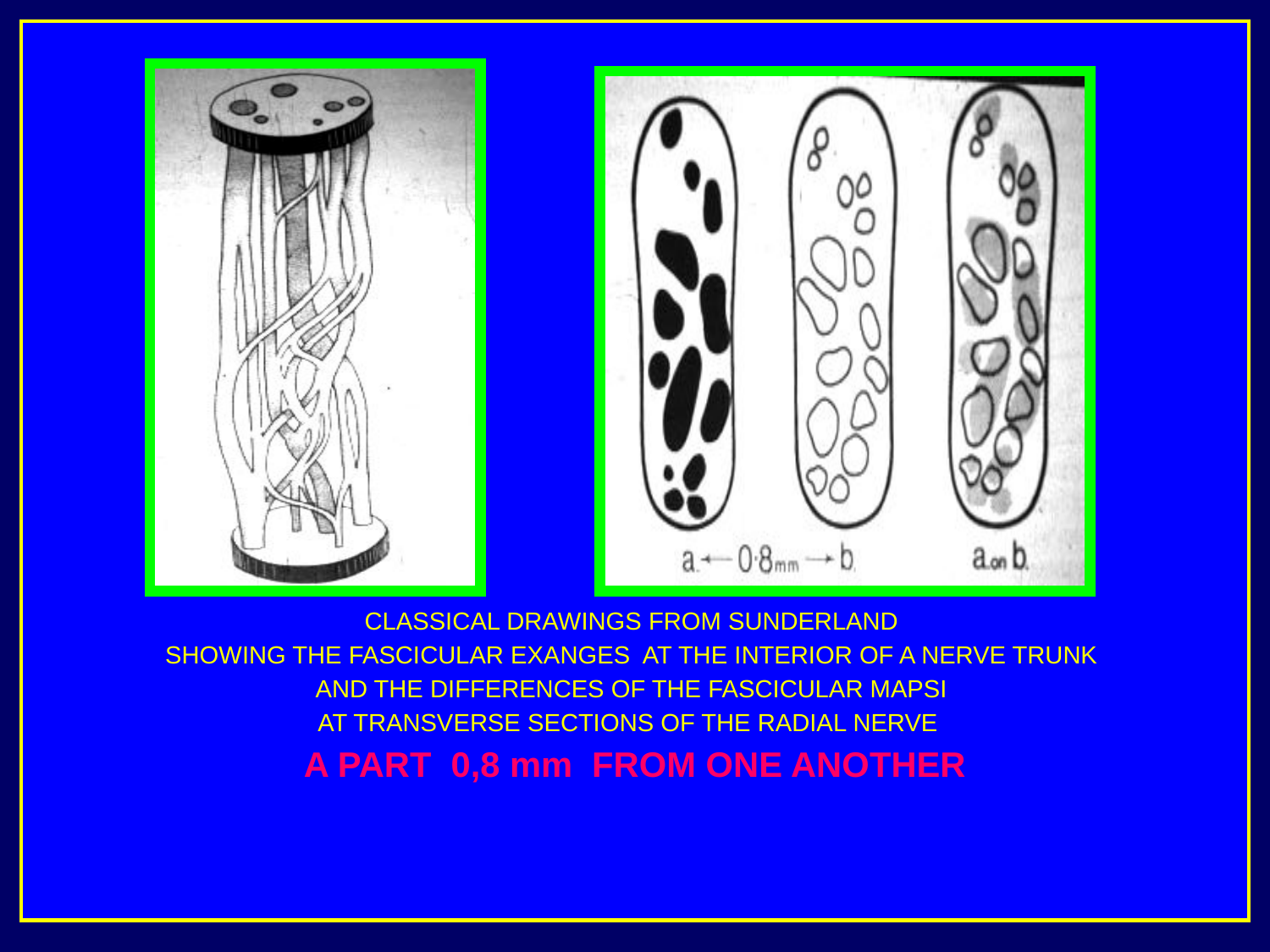

| CLASSICAL DRAWINGS FROM SUNDERLAND SHOWING THE FASCICULAR EXANGES AT THE INTERIOR OF A NERVE TRUNK AND THE DIFFERENCES OF THE FASCICULAR MAPSI AT TRANSVERSE SECTIONS OF THE RADIAL NERVE A PART 0,8 mm FROM ONE ANOTHER |
| --- |

## Slide 36
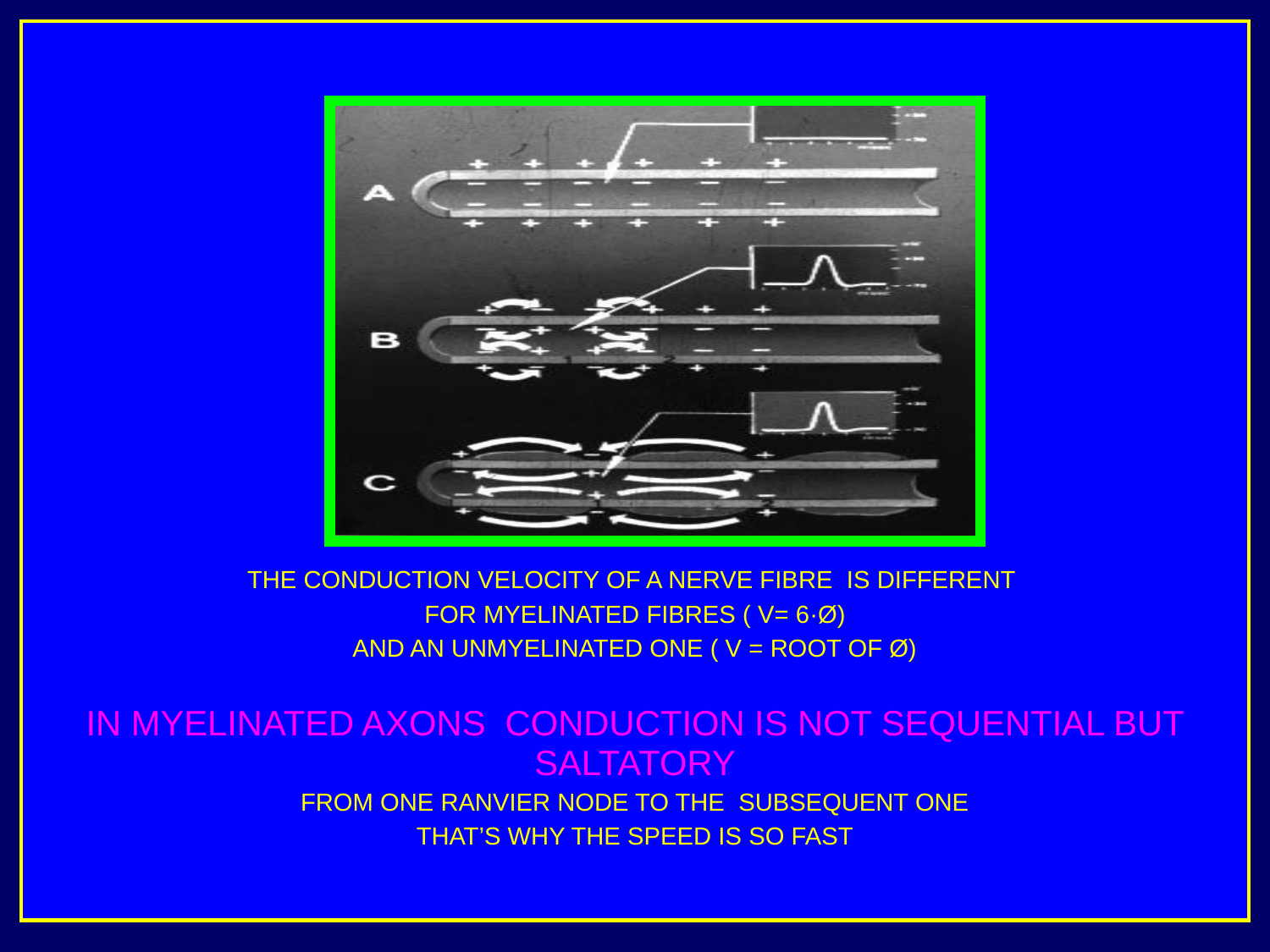

| THE CONDUCTION VELOCITY OF A NERVE FIBRE IS DIFFERENT FOR MYELINATED FIBRES ( V= 6·Ø) AND AN UNMYELINATED ONE ( V = ROOT OF Ø) IN MYELINATED AXONS CONDUCTION IS NOT SEQUENTIAL BUT SALTATORY FROM ONE RANVIER NODE TO THE SUBSEQUENT ONE THAT’S WHY THE SPEED IS SO FAST |
| --- |
# \

## Slide 37
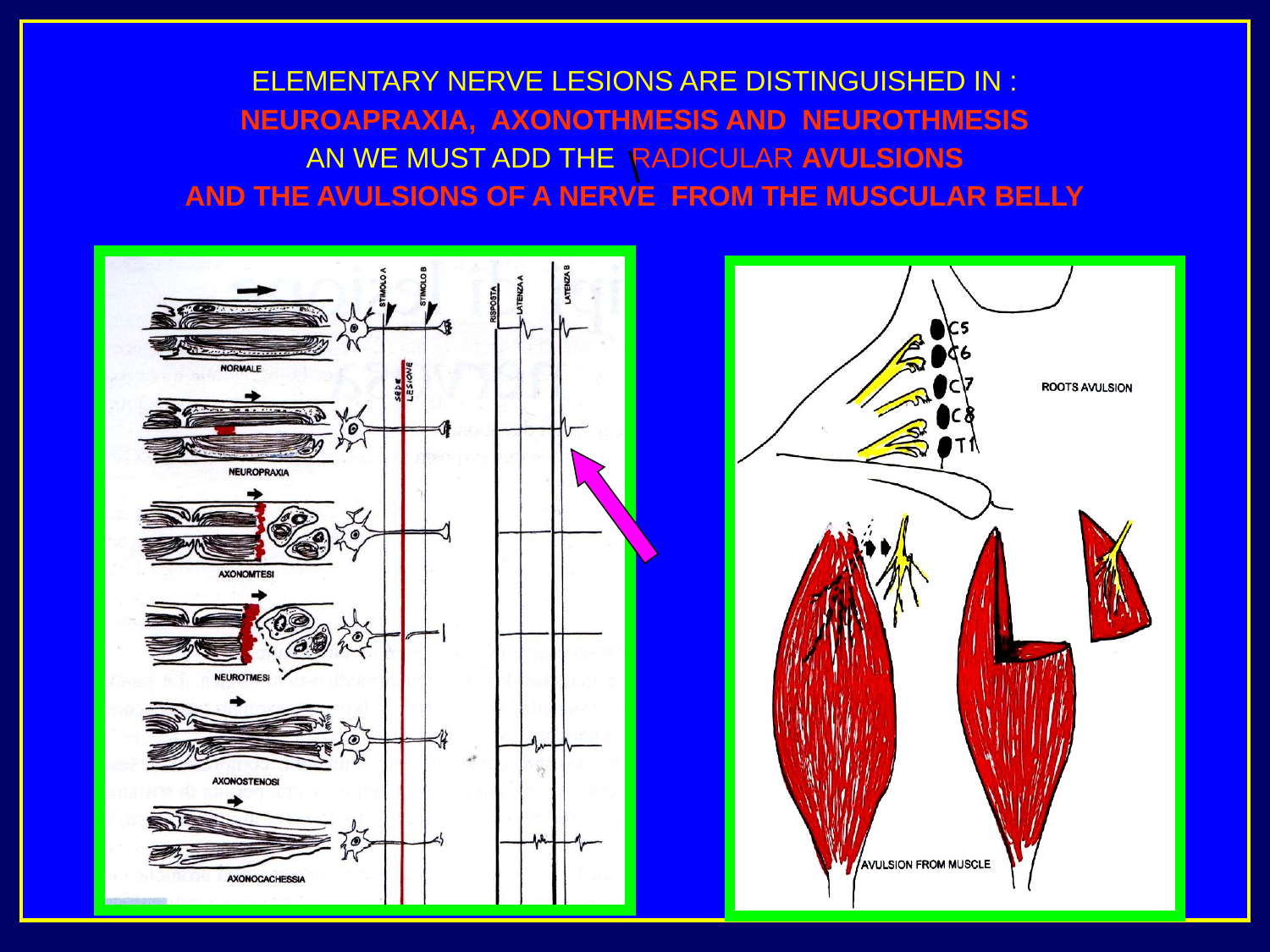

| ELEMENTARY NERVE LESIONS ARE DISTINGUISHED IN : NEUROAPRAXIA, AXONOTHMESIS AND NEUROTHMESIS AN WE MUST ADD THE RADICULAR AVULSIONS AND THE AVULSIONS OF A NERVE FROM THE MUSCULAR BELLY |
| --- |
# \

## Slide 38
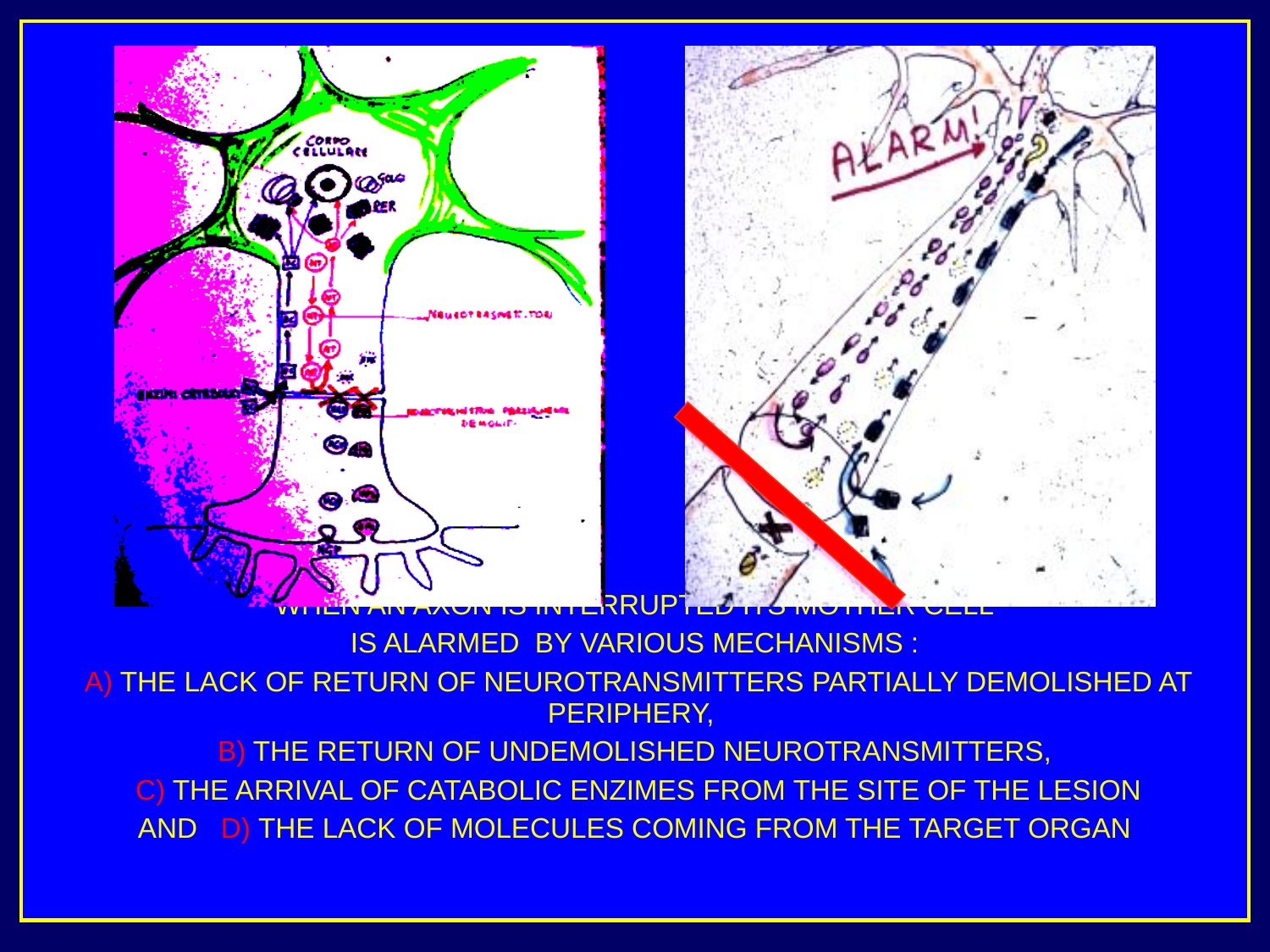

| WHEN AN AXON IS INTERRUPTED ITS MOTHER CELL IS ALARMED BY VARIOUS MECHANISMS : A) THE LACK OF RETURN OF NEUROTRANSMITTERS PARTIALLY DEMOLISHED AT PERIPHERY, B) THE RETURN OF UNDEMOLISHED NEUROTRANSMITTERS, C) THE ARRIVAL OF CATABOLIC ENZIMES FROM THE SITE OF THE LESION AND D) THE LACK OF MOLECULES COMING FROM THE TARGET ORGAN |
| --- |

## Slide 39
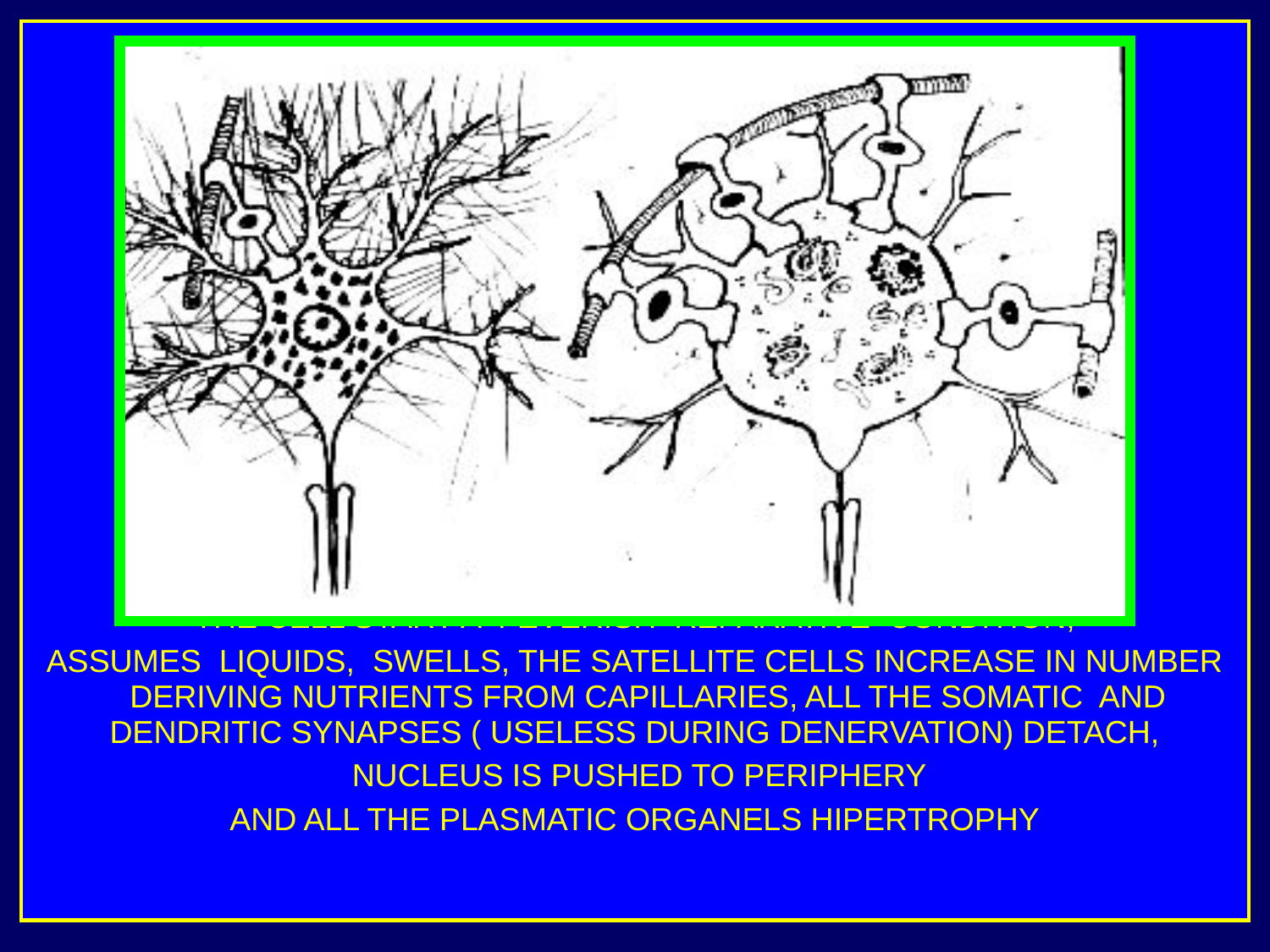

| THE CELL START A FEVERISH REPARATIVE CONDITION, ASSUMES LIQUIDS, SWELLS, THE SATELLITE CELLS INCREASE IN NUMBER DERIVING NUTRIENTS FROM CAPILLARIES, ALL THE SOMATIC AND DENDRITIC SYNAPSES ( USELESS DURING DENERVATION) DETACH, NUCLEUS IS PUSHED TO PERIPHERY AND ALL THE PLASMATIC ORGANELS HIPERTROPHY |
| --- |

## Slide 40
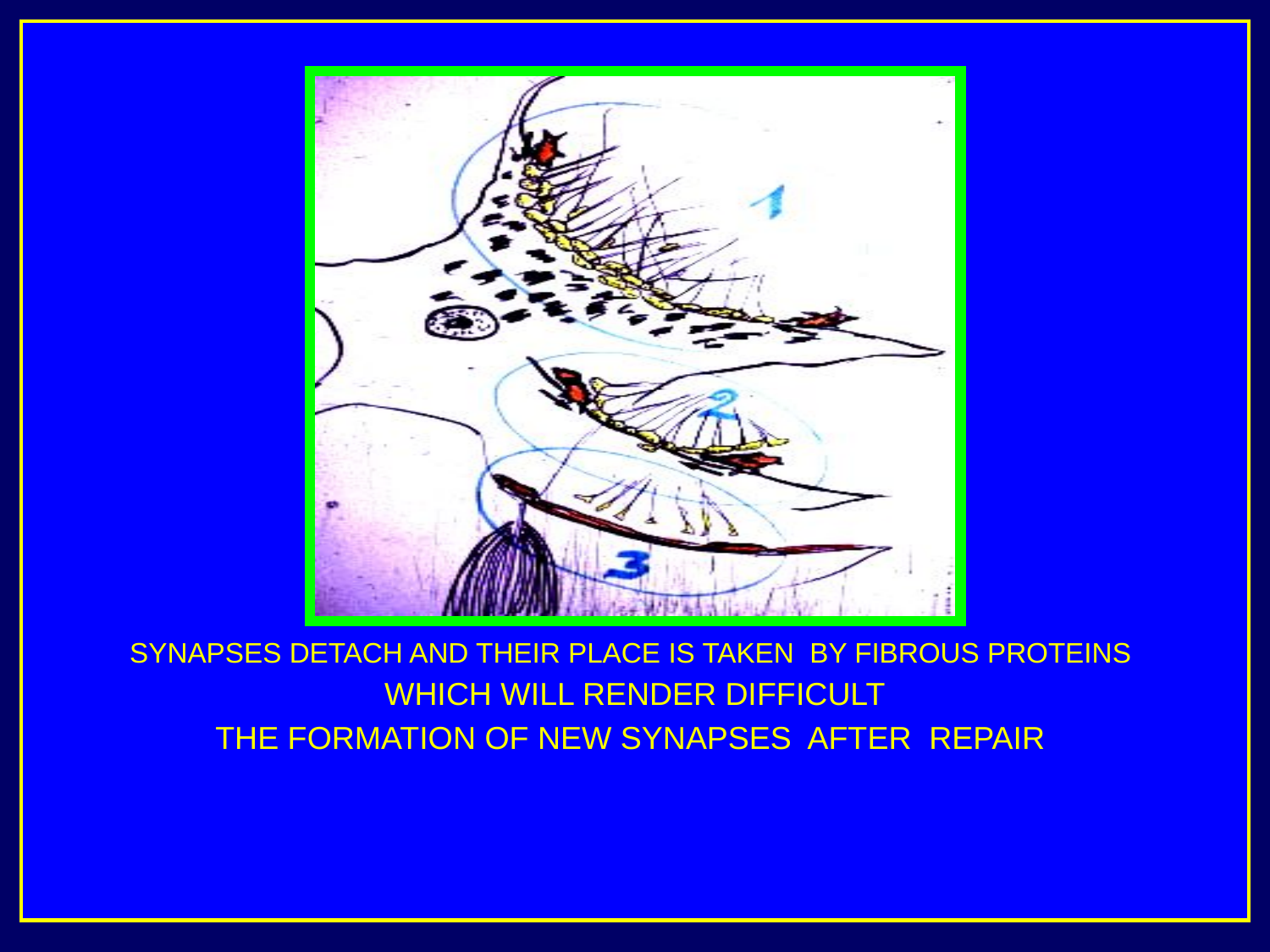

| SYNAPSES DETACH AND THEIR PLACE IS TAKEN BY FIBROUS PROTEINS WHICH WILL RENDER DIFFICULT THE FORMATION OF NEW SYNAPSES AFTER REPAIR |
| --- |

## Slide 41
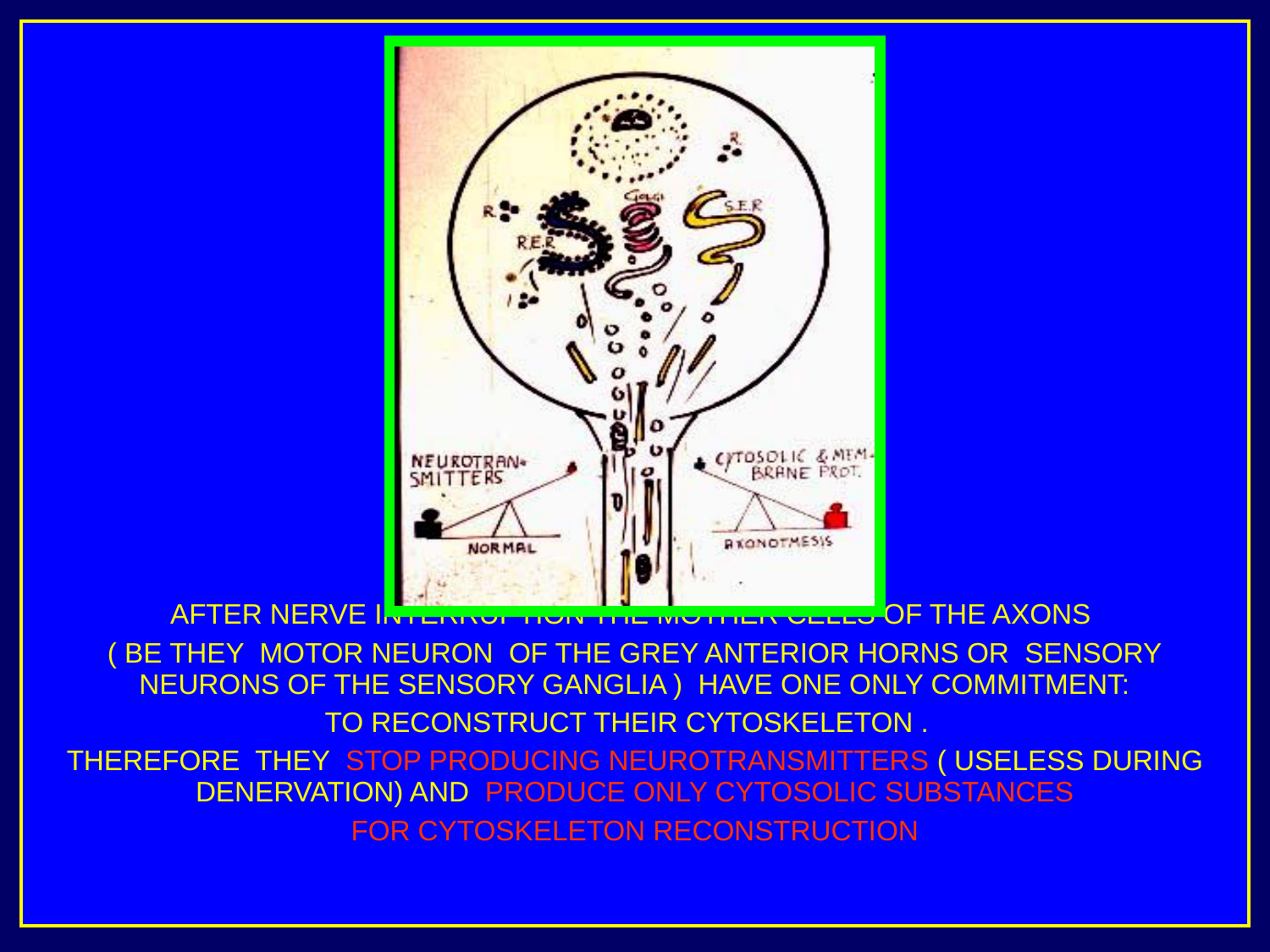

| AFTER NERVE INTERRUPTION THE MOTHER CELLS OF THE AXONS ( BE THEY MOTOR NEURON OF THE GREY ANTERIOR HORNS OR SENSORY NEURONS OF THE SENSORY GANGLIA ) HAVE ONE ONLY COMMITMENT: TO RECONSTRUCT THEIR CYTOSKELETON . THEREFORE THEY STOP PRODUCING NEUROTRANSMITTERS ( USELESS DURING DENERVATION) AND PRODUCE ONLY CYTOSOLIC SUBSTANCES FOR CYTOSKELETON RECONSTRUCTION |
| --- |

## Slide 42
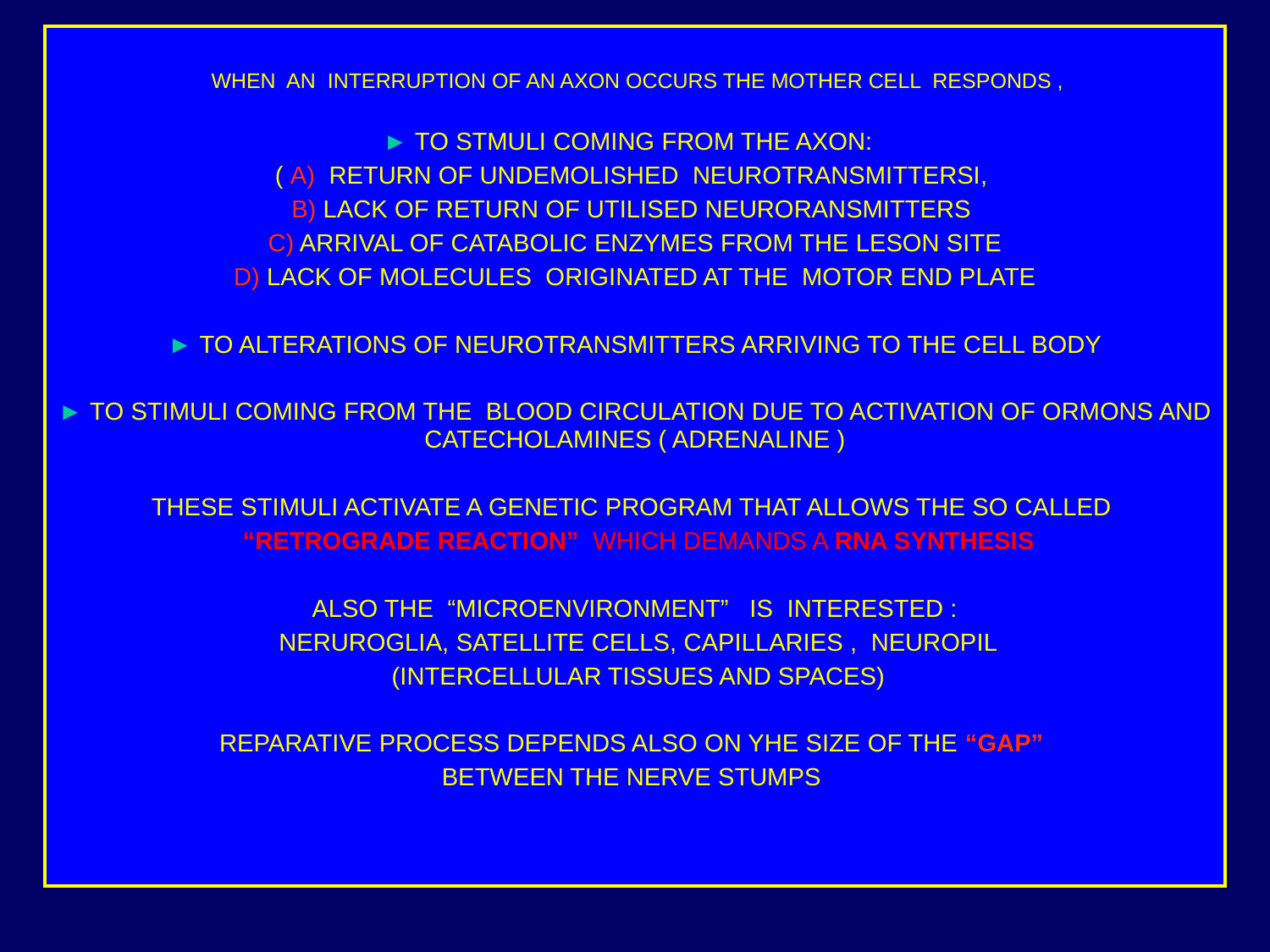

| WHEN AN INTERRUPTION OF AN AXON OCCURS THE MOTHER CELL RESPONDS , ► TO STMULI COMING FROM THE AXON: ( A) RETURN OF UNDEMOLISHED NEUROTRANSMITTERSI, B) LACK OF RETURN OF UTILISED NEURORANSMITTERS C) ARRIVAL OF CATABOLIC ENZYMES FROM THE LESON SITE D) LACK OF MOLECULES ORIGINATED AT THE MOTOR END PLATE ► TO ALTERATIONS OF NEUROTRANSMITTERS ARRIVING TO THE CELL BODY ► TO STIMULI COMING FROM THE BLOOD CIRCULATION DUE TO ACTIVATION OF ORMONS AND CATECHOLAMINES ( ADRENALINE ) THESE STIMULI ACTIVATE A GENETIC PROGRAM THAT ALLOWS THE SO CALLED “RETROGRADE REACTION” WHICH DEMANDS A RNA SYNTHESIS ALSO THE “MICROENVIRONMENT” IS INTERESTED : NERUROGLIA, SATELLITE CELLS, CAPILLARIES , NEUROPIL (INTERCELLULAR TISSUES AND SPACES) REPARATIVE PROCESS DEPENDS ALSO ON YHE SIZE OF THE “GAP” BETWEEN THE NERVE STUMPS |
| --- |

## Slide 43
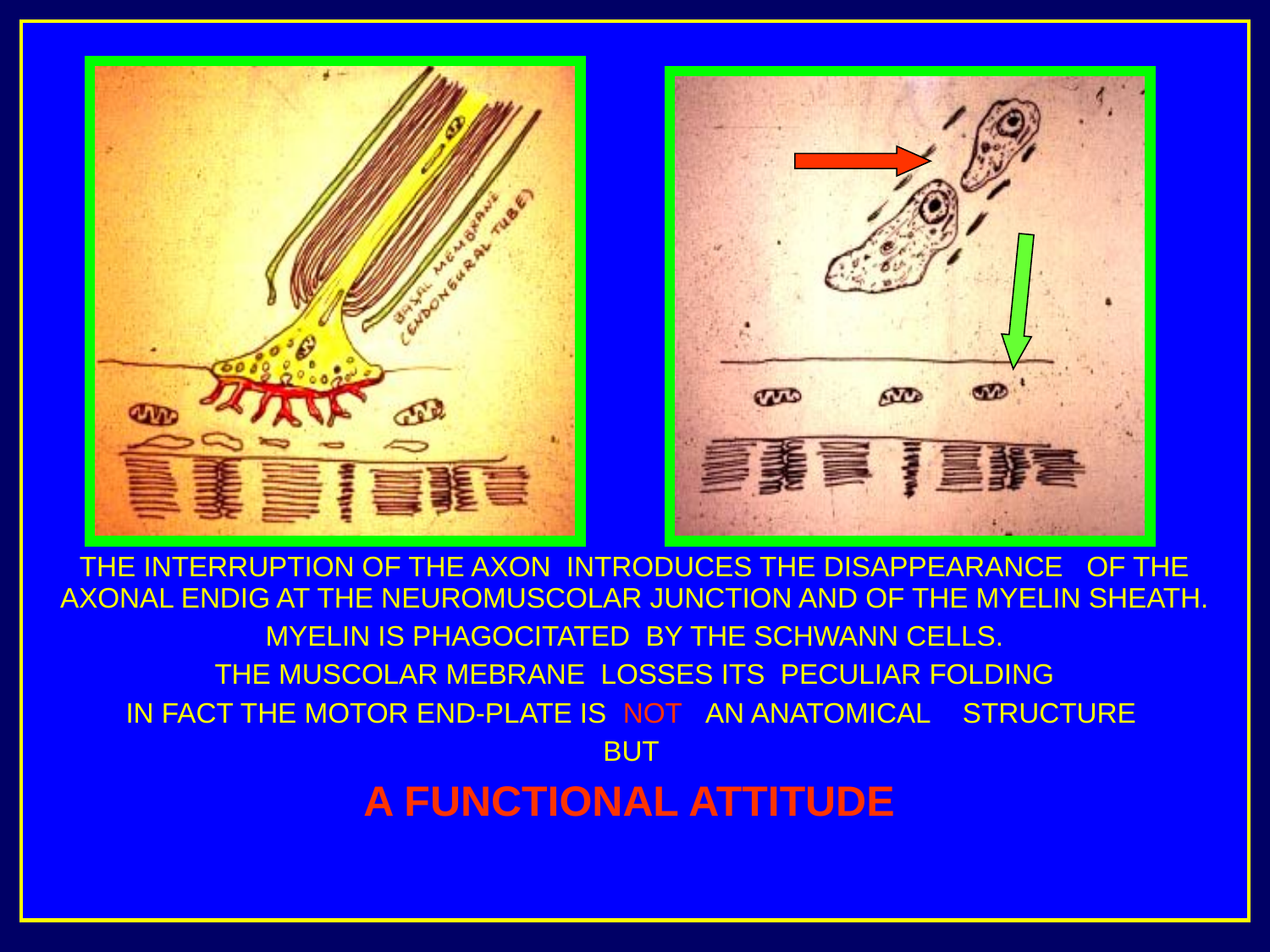

| THE INTERRUPTION OF THE AXON INTRODUCES THE DISAPPEARANCE OF THE AXONAL ENDIG AT THE NEUROMUSCOLAR JUNCTION AND OF THE MYELIN SHEATH. MYELIN IS PHAGOCITATED BY THE SCHWANN CELLS. THE MUSCOLAR MEBRANE LOSSES ITS PECULIAR FOLDING IN FACT THE MOTOR END-PLATE IS NOT AN ANATOMICAL STRUCTURE BUT A FUNCTIONAL ATTITUDE |
| --- |

## Slide 44
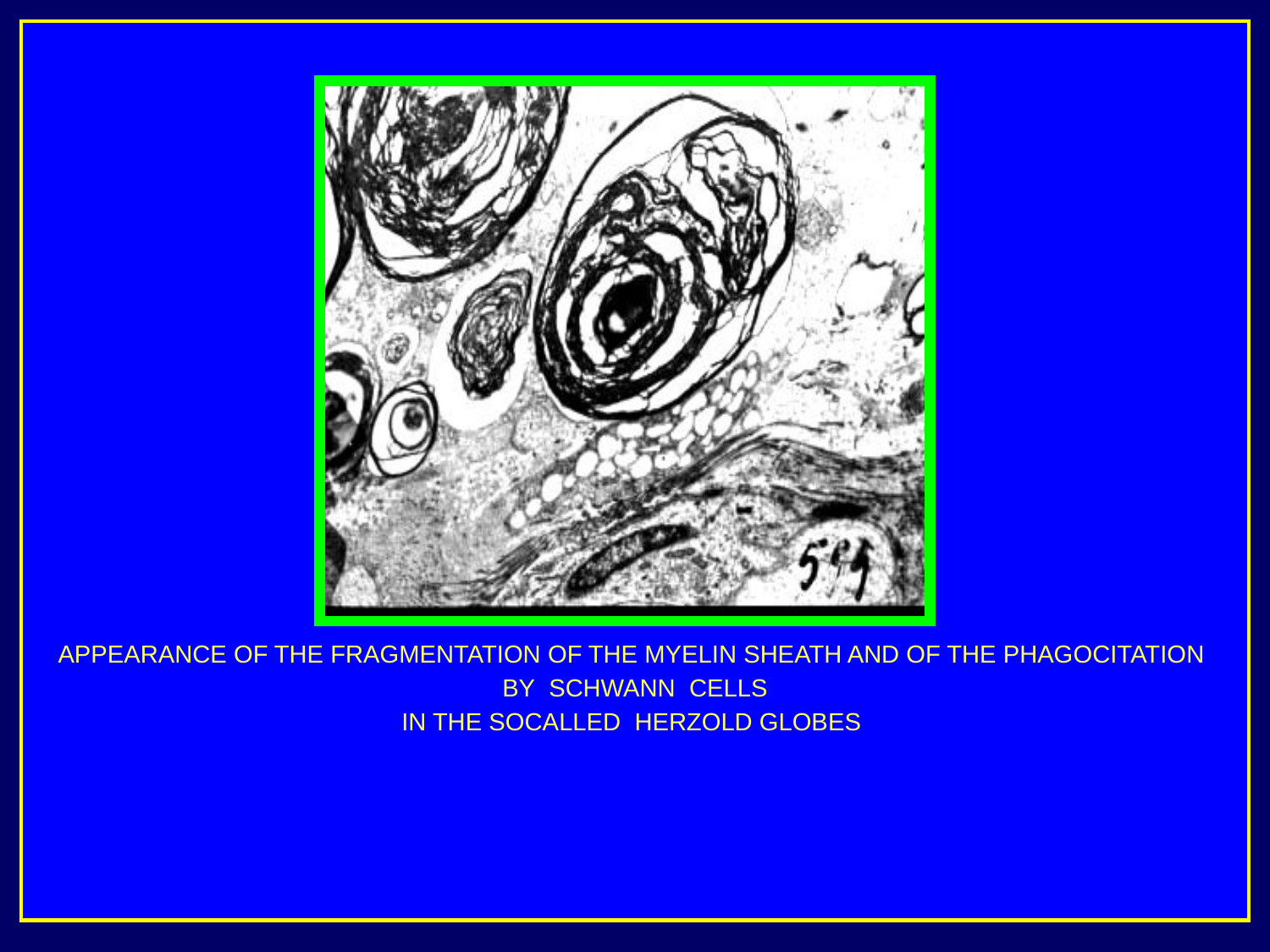

| APPEARANCE OF THE FRAGMENTATION OF THE MYELIN SHEATH AND OF THE PHAGOCITATION BY SCHWANN CELLS IN THE SOCALLED HERZOLD GLOBES |
| --- |
# \

## Slide 45
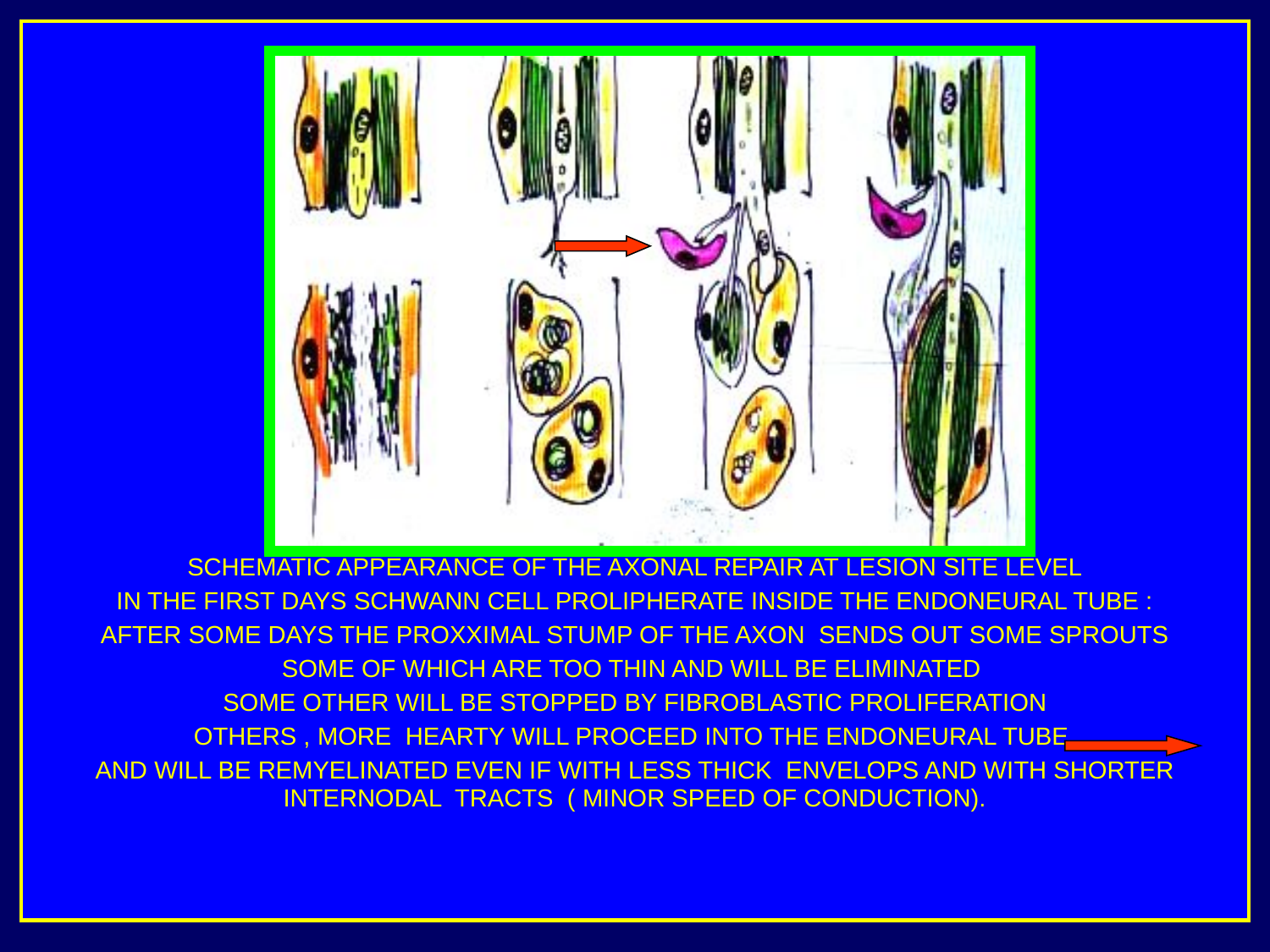

| SCHEMATIC APPEARANCE OF THE AXONAL REPAIR AT LESION SITE LEVEL IN THE FIRST DAYS SCHWANN CELL PROLIPHERATE INSIDE THE ENDONEURAL TUBE : AFTER SOME DAYS THE PROXXIMAL STUMP OF THE AXON SENDS OUT SOME SPROUTS SOME OF WHICH ARE TOO THIN AND WILL BE ELIMINATED SOME OTHER WILL BE STOPPED BY FIBROBLASTIC PROLIFERATION OTHERS , MORE HEARTY WILL PROCEED INTO THE ENDONEURAL TUBE AND WILL BE REMYELINATED EVEN IF WITH LESS THICK ENVELOPS AND WITH SHORTER INTERNODAL TRACTS ( MINOR SPEED OF CONDUCTION). |
| --- |
# \

## Slide 46
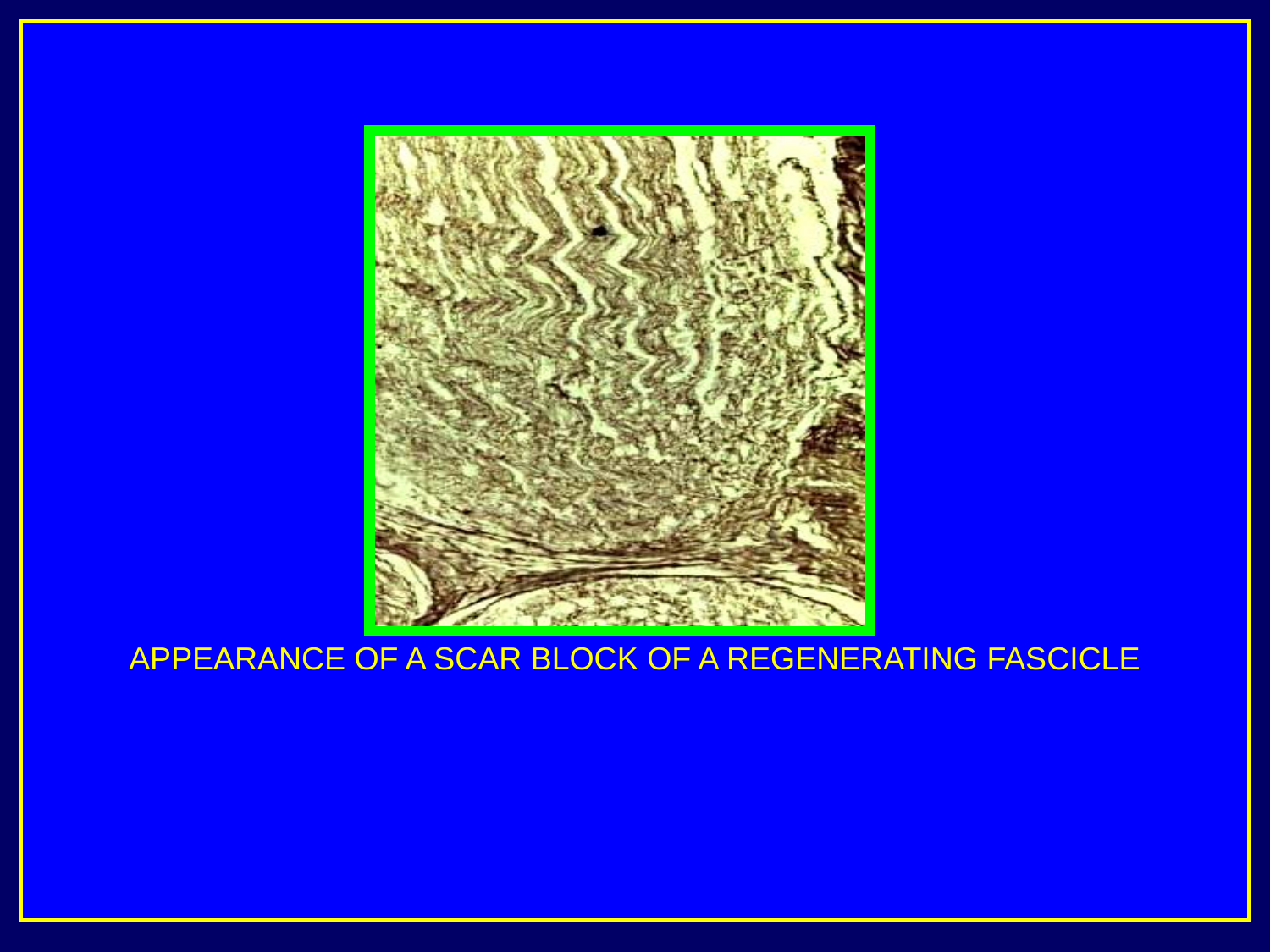

| APPEARANCE OF A SCAR BLOCK OF A REGENERATING FASCICLE |
| --- |
# \

## Slide 47
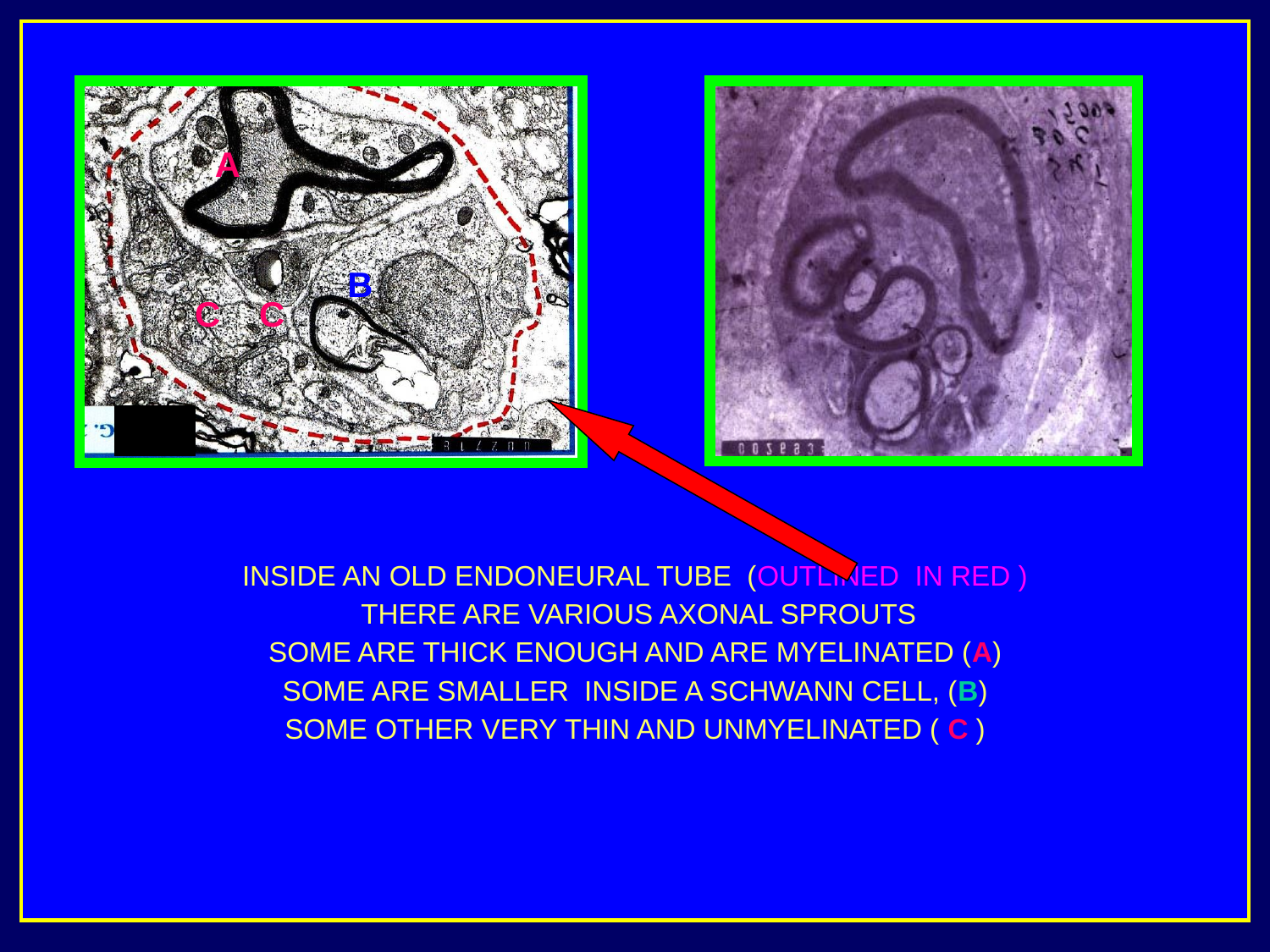

| INSIDE AN OLD ENDONEURAL TUBE (OUTLINED IN RED ) THERE ARE VARIOUS AXONAL SPROUTS SOME ARE THICK ENOUGH AND ARE MYELINATED (A) SOME ARE SMALLER INSIDE A SCHWANN CELL, (B) SOME OTHER VERY THIN AND UNMYELINATED ( C ) |
| --- |
A
B
C C

## Slide 48
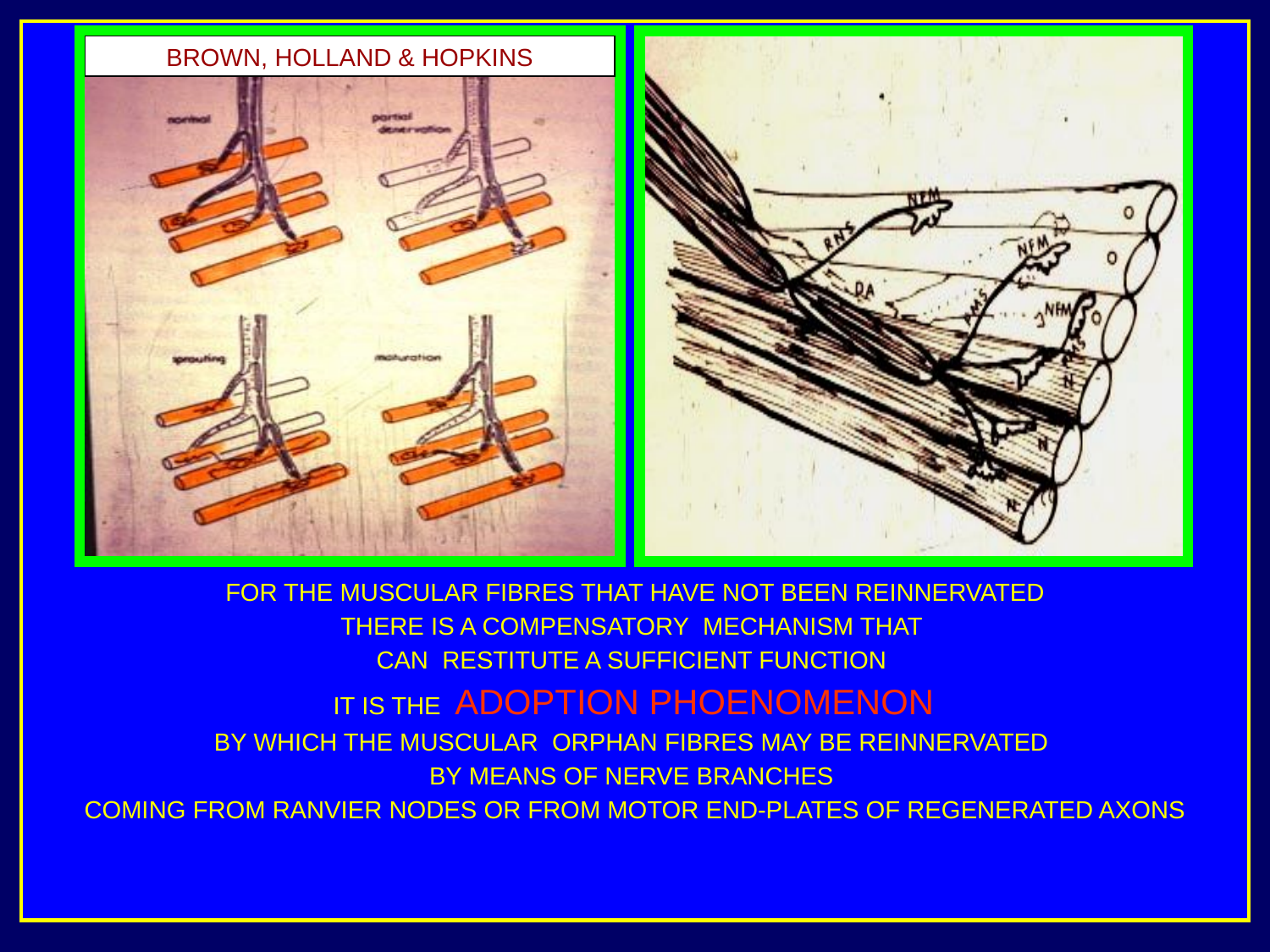

| FOR THE MUSCULAR FIBRES THAT HAVE NOT BEEN REINNERVATED THERE IS A COMPENSATORY MECHANISM THAT CAN RESTITUTE A SUFFICIENT FUNCTION IT IS THE ADOPTION PHOENOMENON BY WHICH THE MUSCULAR ORPHAN FIBRES MAY BE REINNERVATED BY MEANS OF NERVE BRANCHES COMING FROM RANVIER NODES OR FROM MOTOR END-PLATES OF REGENERATED AXONS |
| --- |
BROWN, HOLLAND & HOPKINS

## Slide 49
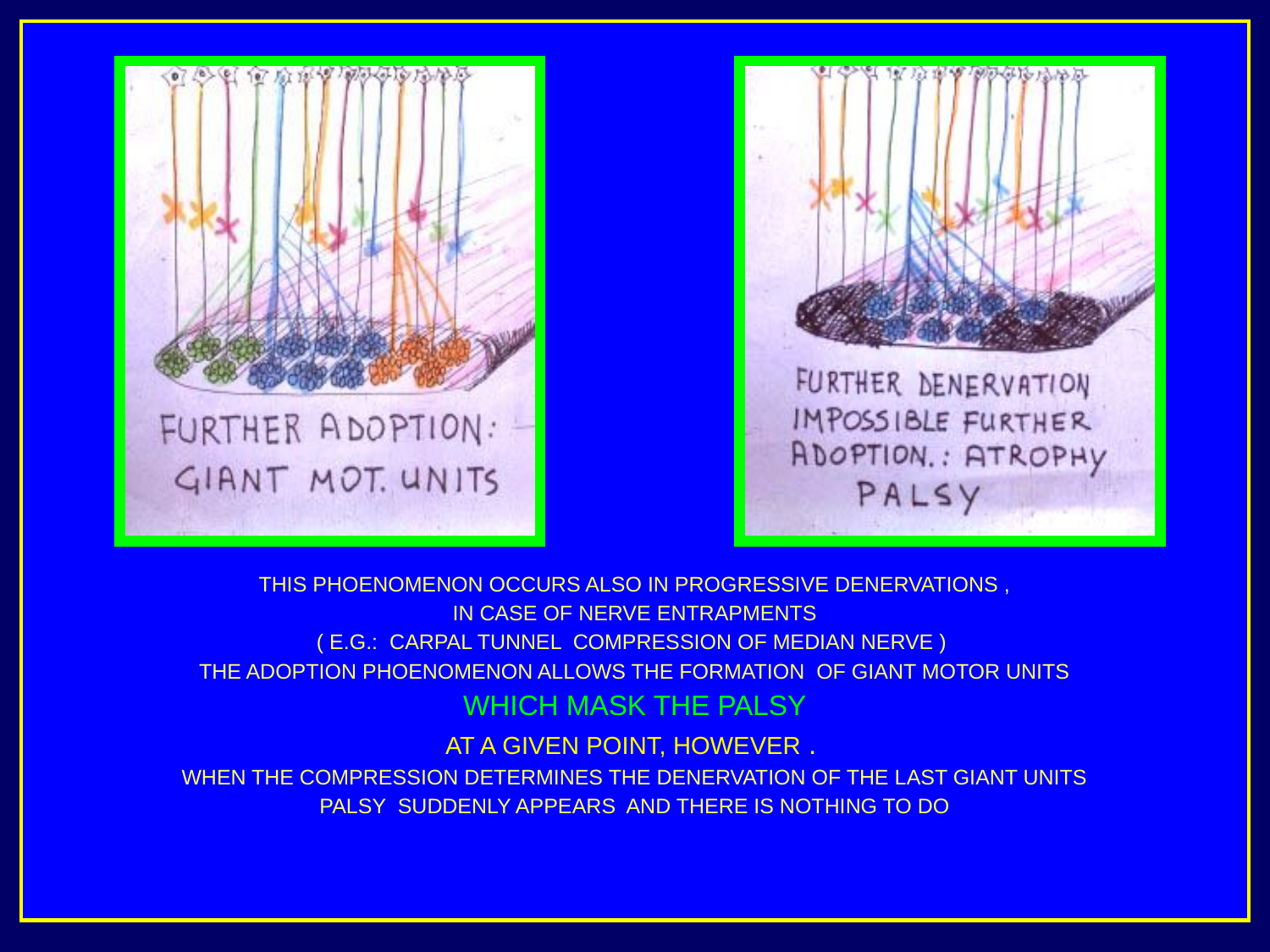

| THIS PHOENOMENON OCCURS ALSO IN PROGRESSIVE DENERVATIONS , IN CASE OF NERVE ENTRAPMENTS ( E.G.: CARPAL TUNNEL COMPRESSION OF MEDIAN NERVE ) THE ADOPTION PHOENOMENON ALLOWS THE FORMATION OF GIANT MOTOR UNITS WHICH MASK THE PALSY AT A GIVEN POINT, HOWEVER . WHEN THE COMPRESSION DETERMINES THE DENERVATION OF THE LAST GIANT UNITS PALSY SUDDENLY APPEARS AND THERE IS NOTHING TO DO |
| --- |

## Slide 50
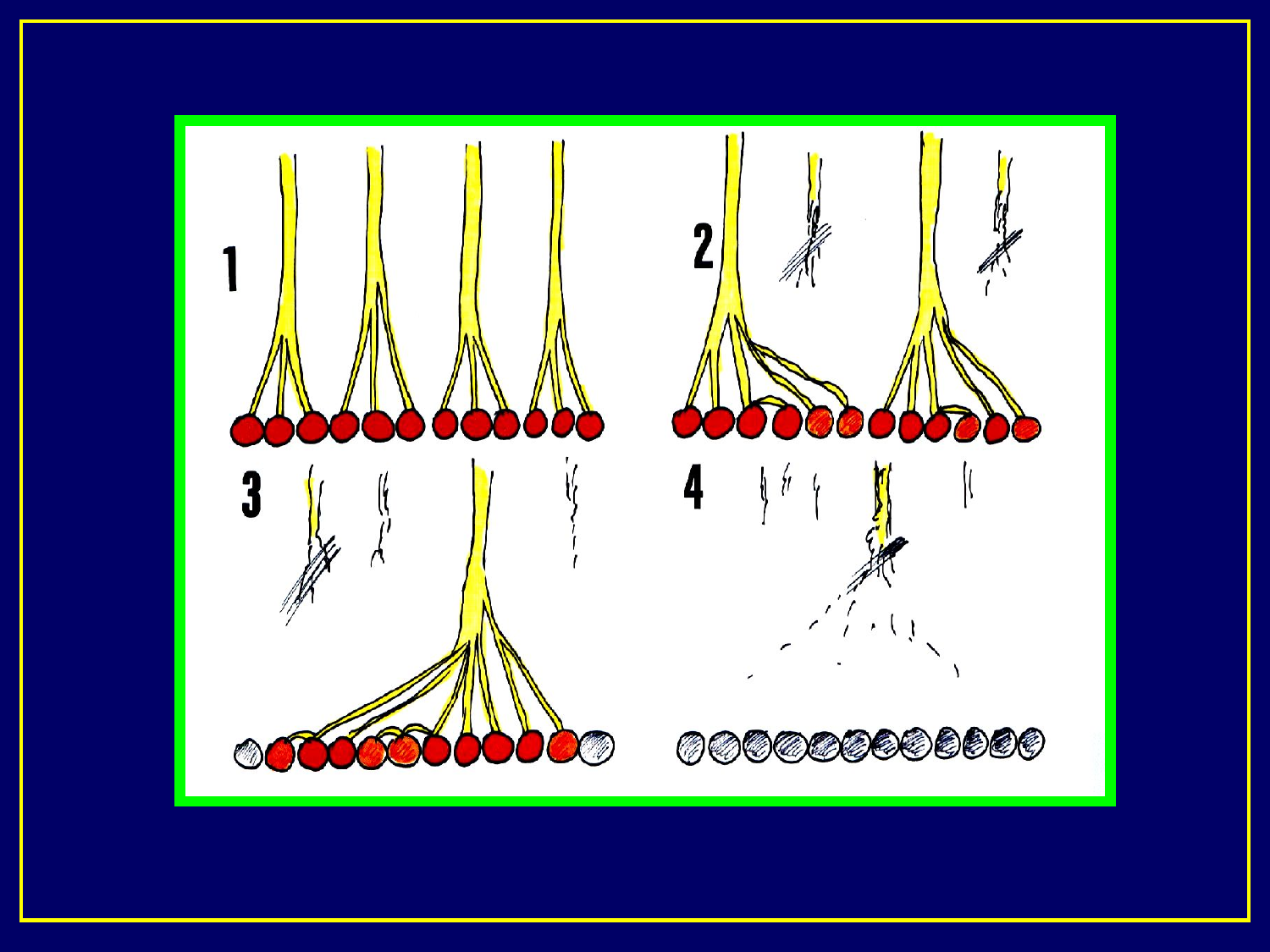

| |
| --- |

## Slide 51
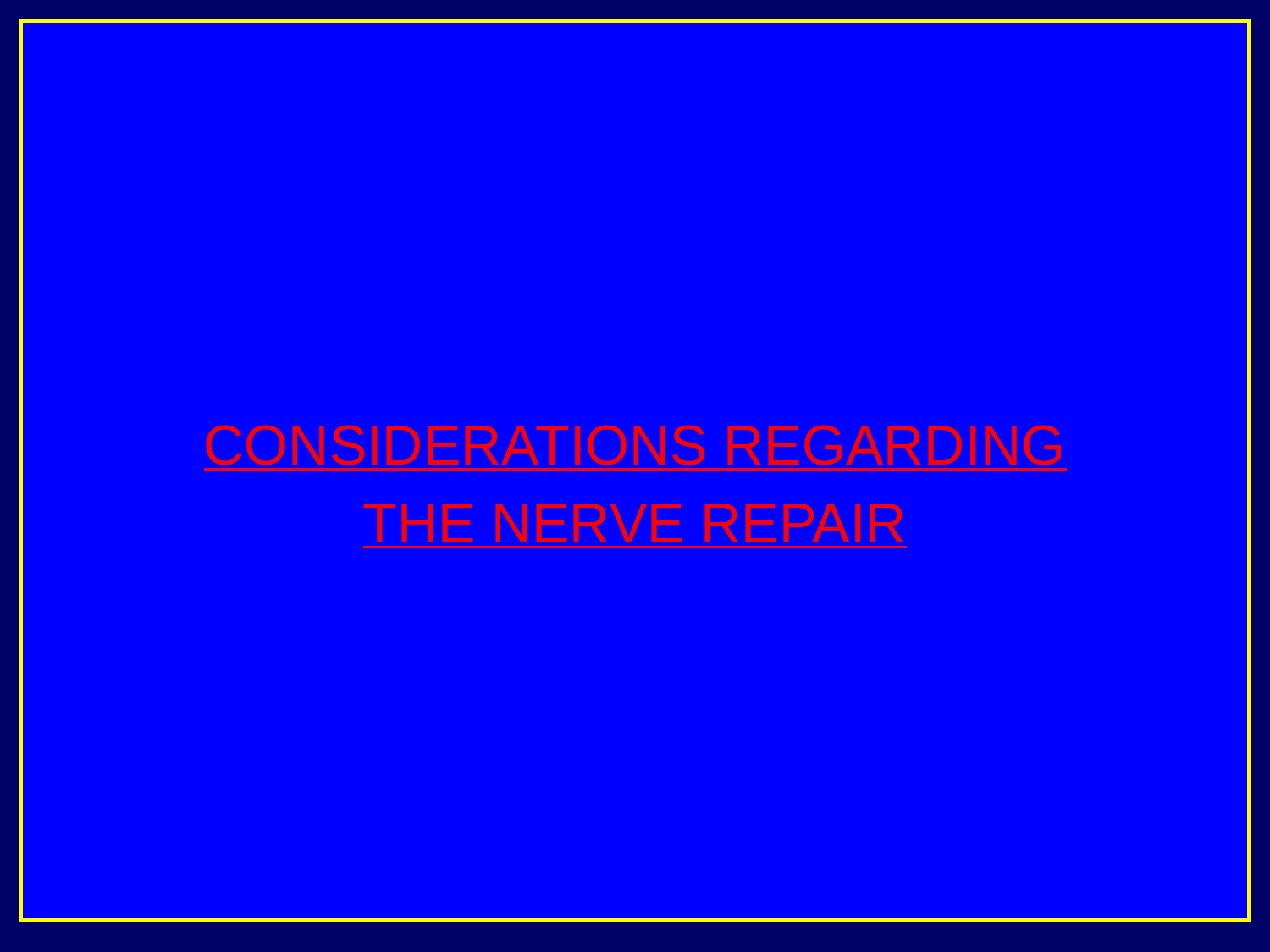

| CONSIDERATIONS REGARDING THE NERVE REPAIR |
| --- |

## Slide 52
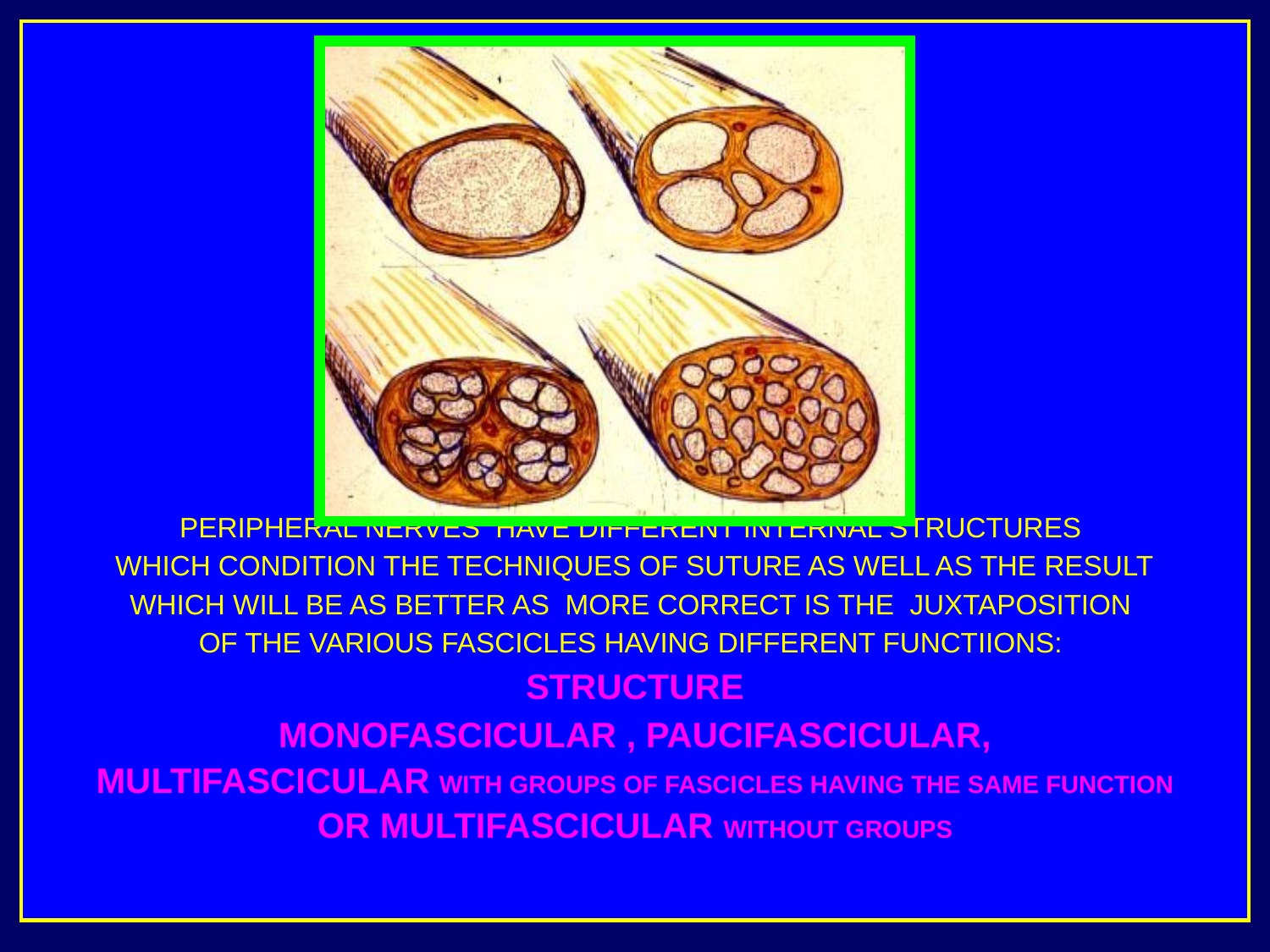

| PERIPHERAL NERVES HAVE DIFFERENT INTERNAL STRUCTURES WHICH CONDITION THE TECHNIQUES OF SUTURE AS WELL AS THE RESULT WHICH WILL BE AS BETTER AS MORE CORRECT IS THE JUXTAPOSITION OF THE VARIOUS FASCICLES HAVING DIFFERENT FUNCTIIONS: STRUCTURE MONOFASCICULAR , PAUCIFASCICULAR, MULTIFASCICULAR WITH GROUPS OF FASCICLES HAVING THE SAME FUNCTION OR MULTIFASCICULAR WITHOUT GROUPS |
| --- |

## Slide 53
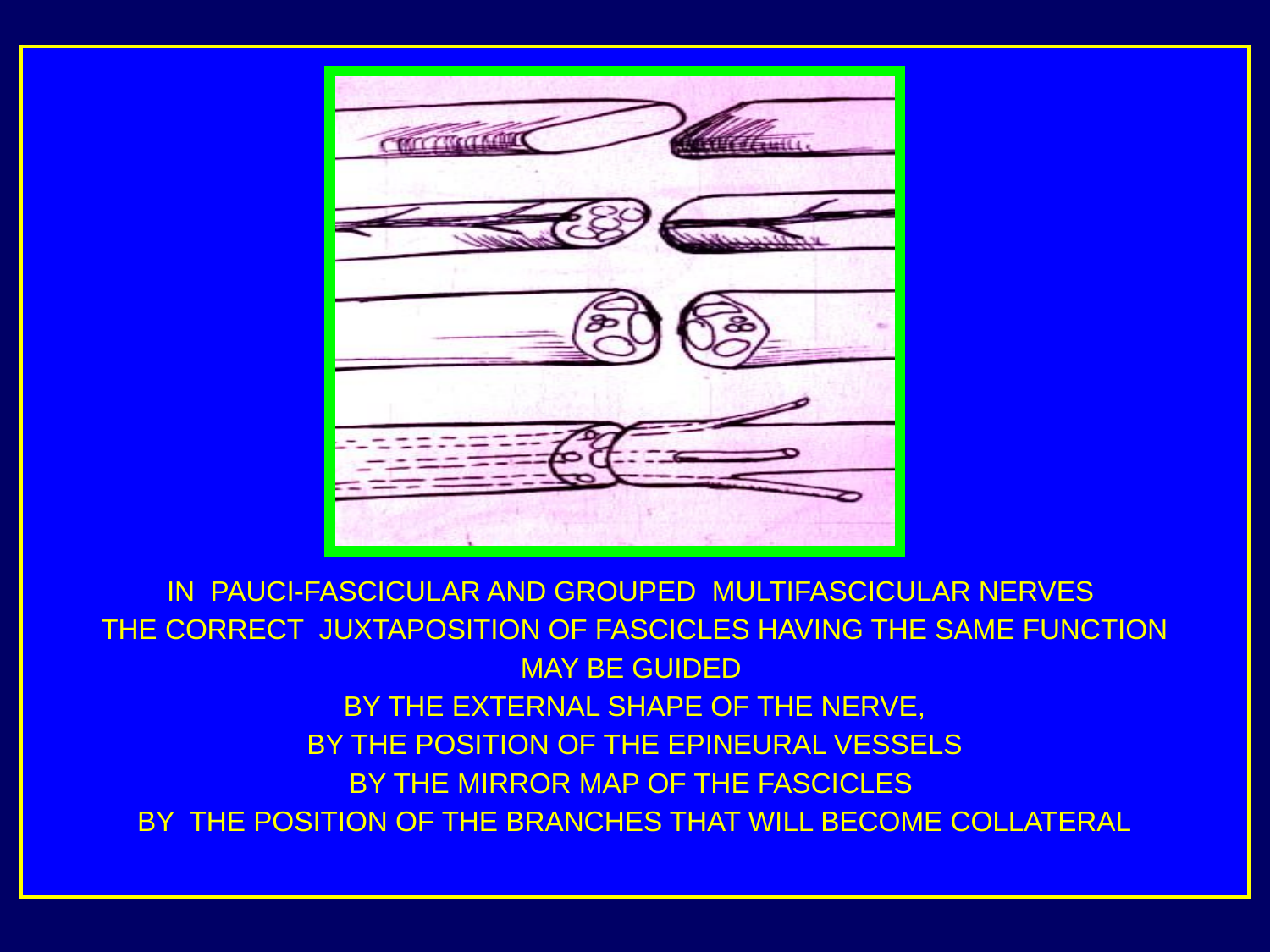

| IN PAUCI-FASCICULAR AND GROUPED MULTIFASCICULAR NERVES THE CORRECT JUXTAPOSITION OF FASCICLES HAVING THE SAME FUNCTION MAY BE GUIDED BY THE EXTERNAL SHAPE OF THE NERVE, BY THE POSITION OF THE EPINEURAL VESSELS BY THE MIRROR MAP OF THE FASCICLES BY THE POSITION OF THE BRANCHES THAT WILL BECOME COLLATERAL |
| --- |

## Slide 54
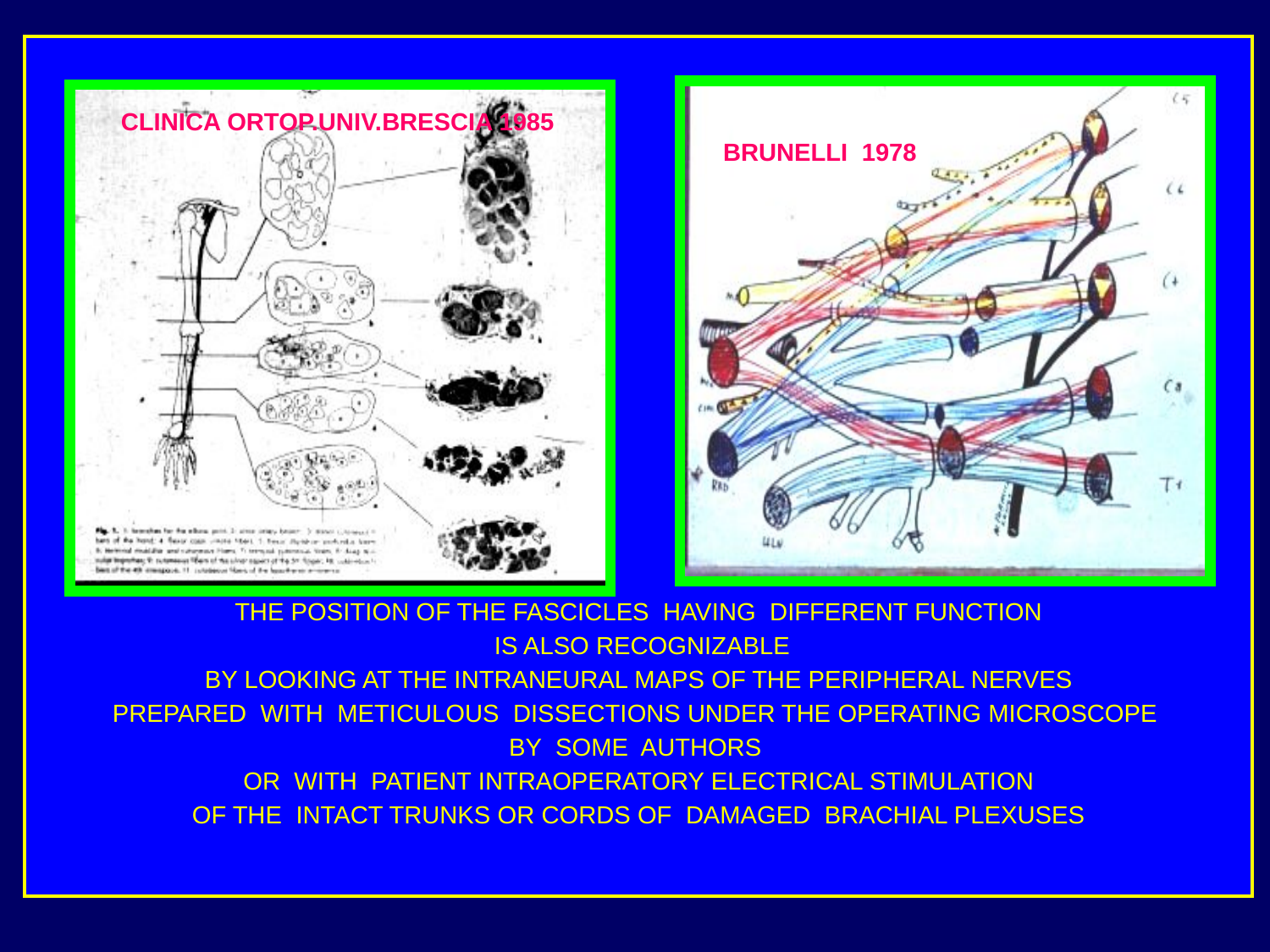

| THE POSITION OF THE FASCICLES HAVING DIFFERENT FUNCTION IS ALSO RECOGNIZABLE BY LOOKING AT THE INTRANEURAL MAPS OF THE PERIPHERAL NERVES PREPARED WITH METICULOUS DISSECTIONS UNDER THE OPERATING MICROSCOPE BY SOME AUTHORS OR WITH PATIENT INTRAOPERATORY ELECTRICAL STIMULATION OF THE INTACT TRUNKS OR CORDS OF DAMAGED BRACHIAL PLEXUSES |
| --- |
CLINICA ORTOP.UNIV.BRESCIA 1985
BRUNELLI 1978

## Slide 55
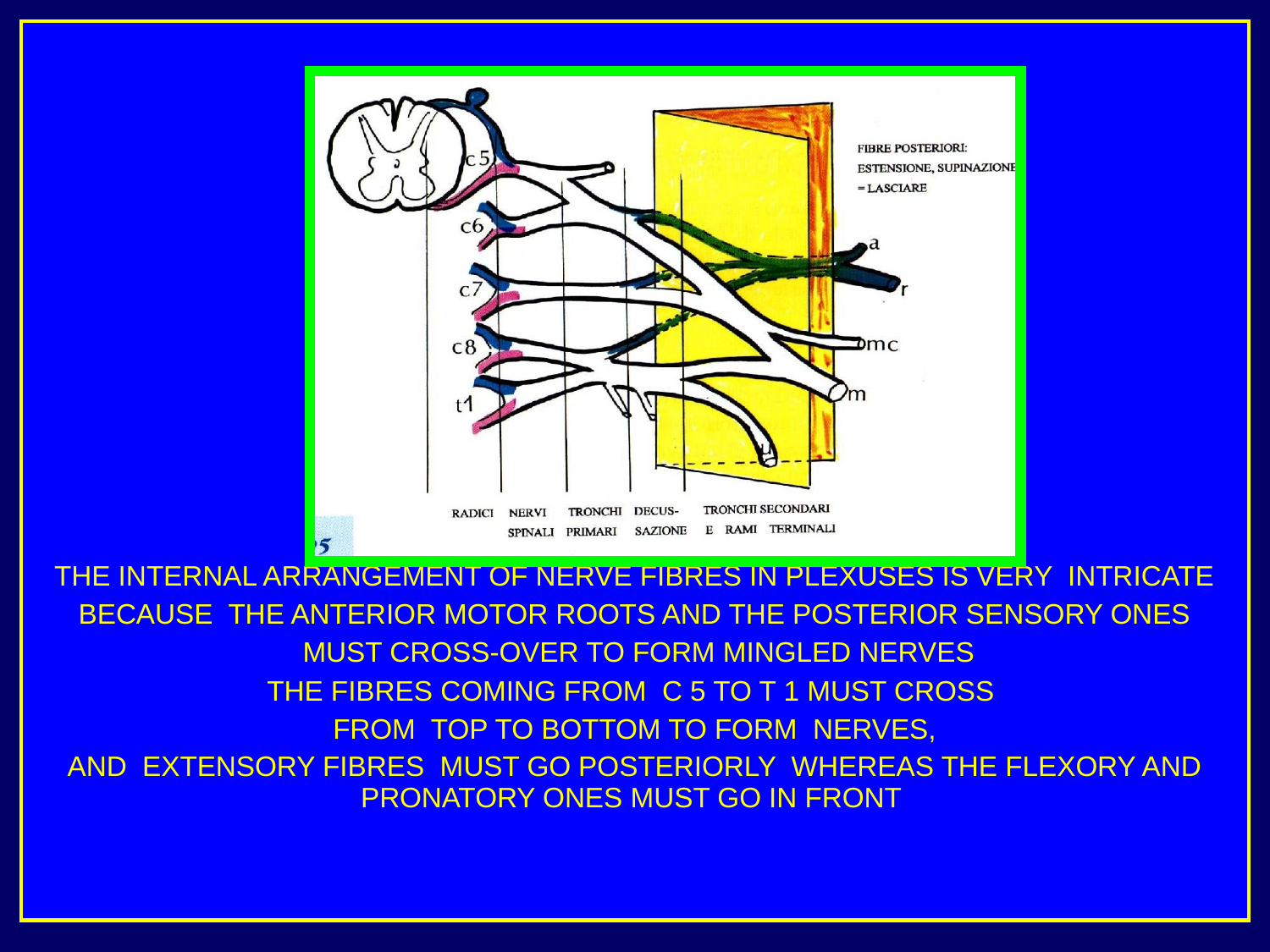

| THE INTERNAL ARRANGEMENT OF NERVE FIBRES IN PLEXUSES IS VERY INTRICATE BECAUSE THE ANTERIOR MOTOR ROOTS AND THE POSTERIOR SENSORY ONES MUST CROSS-OVER TO FORM MINGLED NERVES THE FIBRES COMING FROM C 5 TO T 1 MUST CROSS FROM TOP TO BOTTOM TO FORM NERVES, AND EXTENSORY FIBRES MUST GO POSTERIORLY WHEREAS THE FLEXORY AND PRONATORY ONES MUST GO IN FRONT |
| --- |

## Slide 56
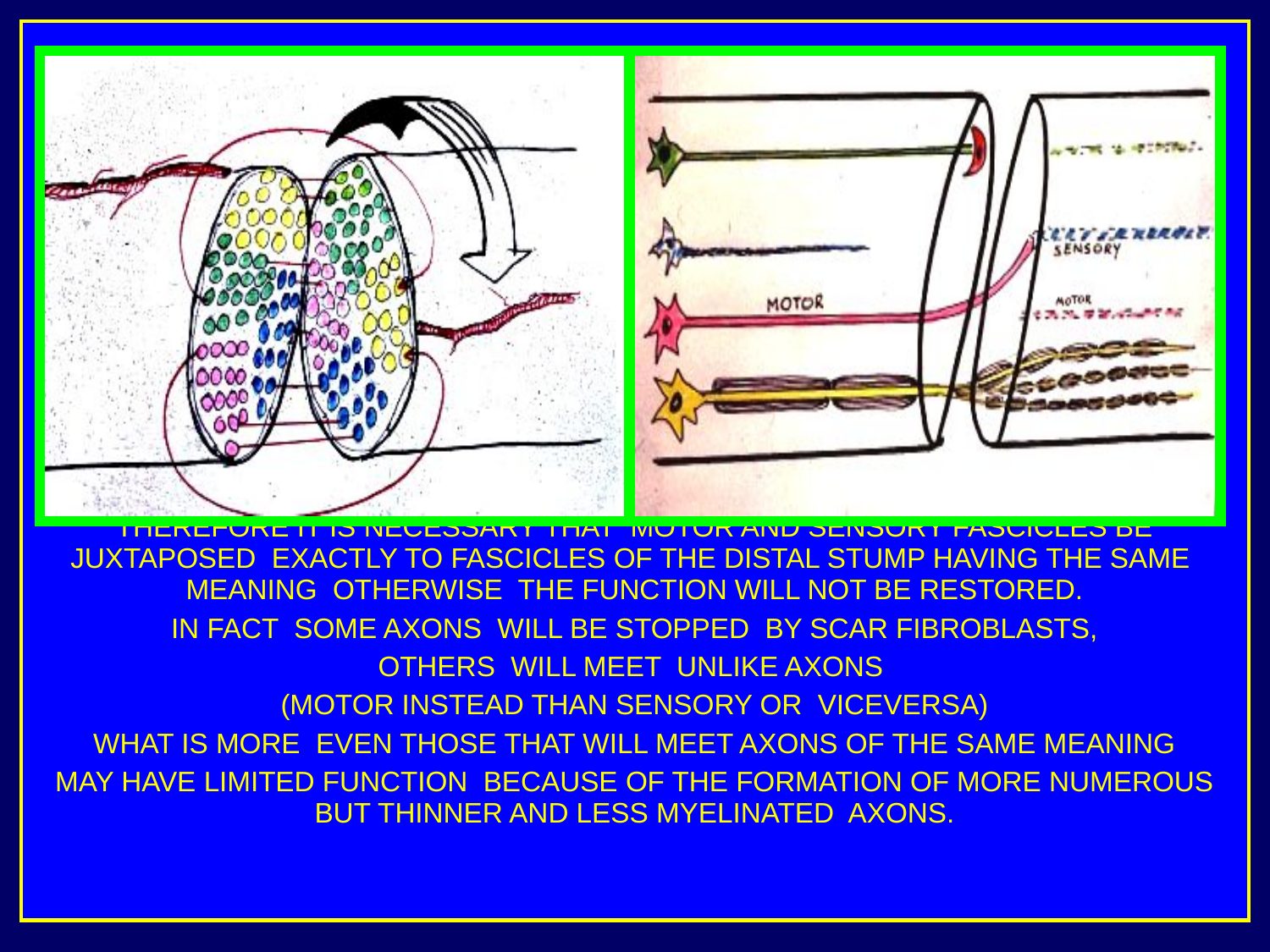

| THEREFORE IT IS NECESSARY THAT MOTOR AND SENSORY FASCICLES BE JUXTAPOSED EXACTLY TO FASCICLES OF THE DISTAL STUMP HAVING THE SAME MEANING OTHERWISE THE FUNCTION WILL NOT BE RESTORED. IN FACT SOME AXONS WILL BE STOPPED BY SCAR FIBROBLASTS, OTHERS WILL MEET UNLIKE AXONS (MOTOR INSTEAD THAN SENSORY OR VICEVERSA) WHAT IS MORE EVEN THOSE THAT WILL MEET AXONS OF THE SAME MEANING MAY HAVE LIMITED FUNCTION BECAUSE OF THE FORMATION OF MORE NUMEROUS BUT THINNER AND LESS MYELINATED AXONS. |
| --- |

## Slide 57
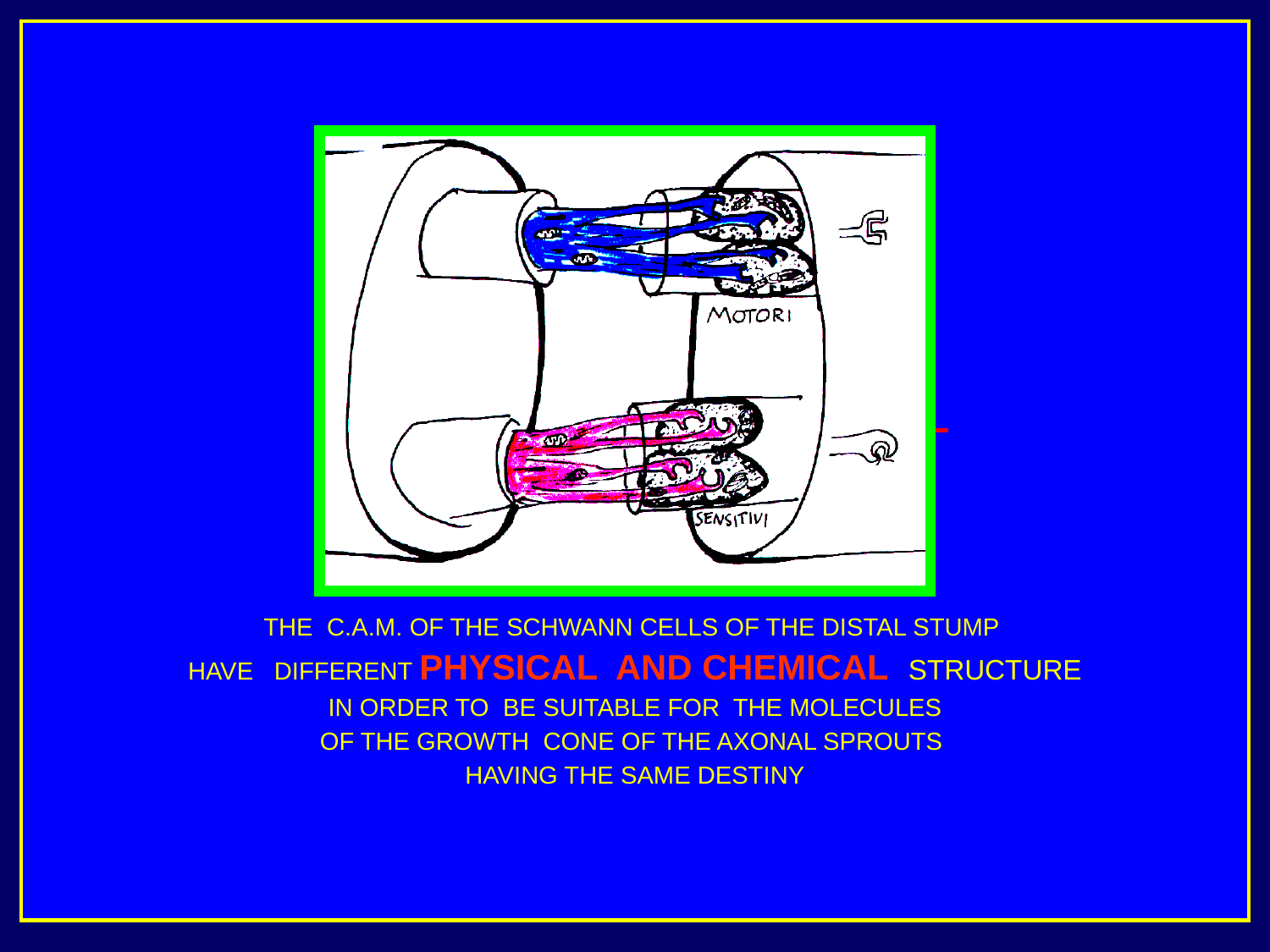

| THE C.A.M. OF THE SCHWANN CELLS OF THE DISTAL STUMP HAVE DIFFERENT PHYSICAL AND CHEMICAL STRUCTURE IN ORDER TO BE SUITABLE FOR THE MOLECULES OF THE GROWTH CONE OF THE AXONAL SPROUTS HAVING THE SAME DESTINY |
| --- |
#

## Slide 58
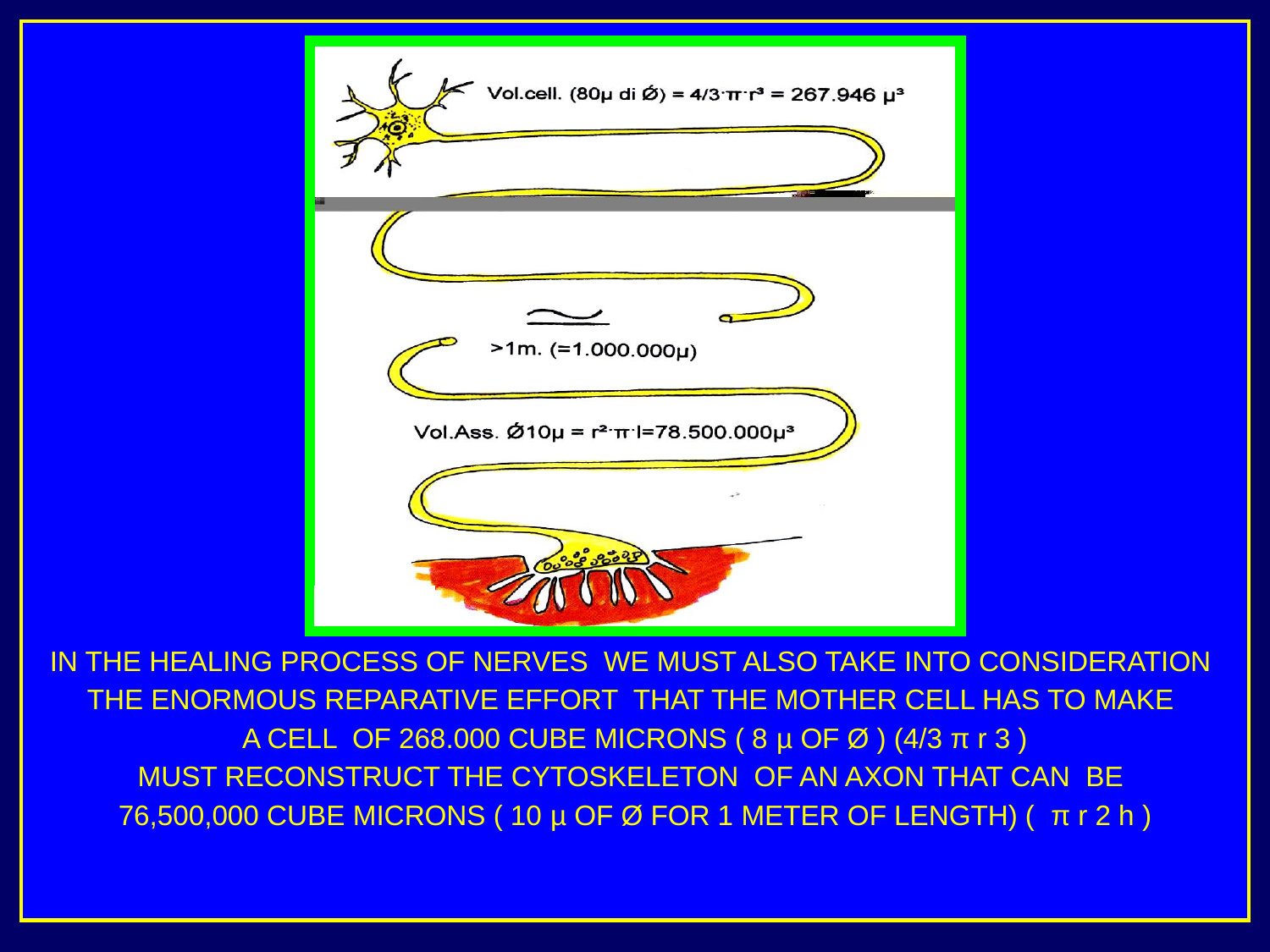

| IN THE HEALING PROCESS OF NERVES WE MUST ALSO TAKE INTO CONSIDERATION THE ENORMOUS REPARATIVE EFFORT THAT THE MOTHER CELL HAS TO MAKE A CELL OF 268.000 CUBE MICRONS ( 8 µ OF Ø ) (4/3 π r 3 ) MUST RECONSTRUCT THE CYTOSKELETON OF AN AXON THAT CAN BE 76,500,000 CUBE MICRONS ( 10 µ OF Ø FOR 1 METER OF LENGTH) ( π r 2 h ) |
| --- |

## Slide 59
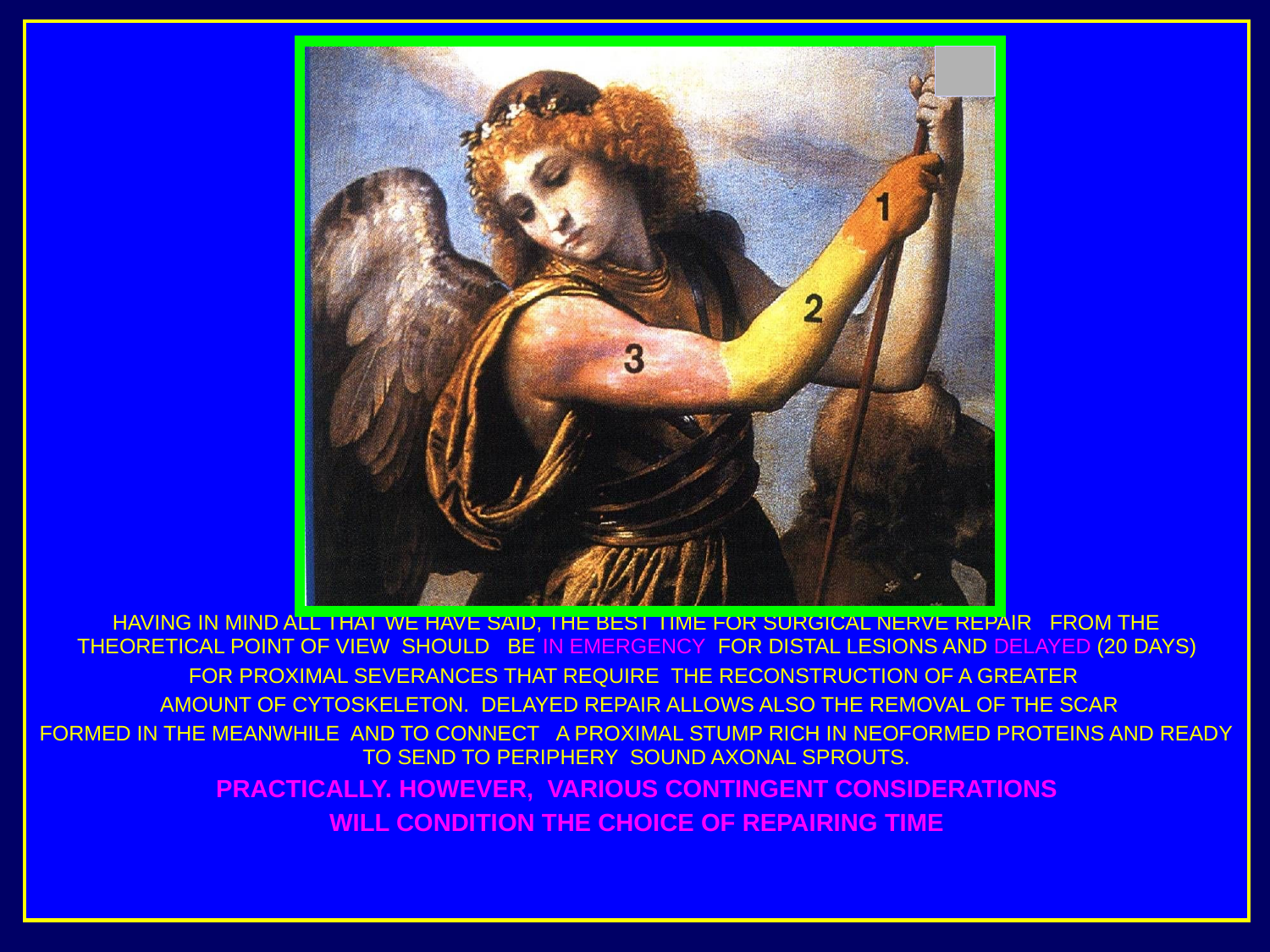

| HAVING IN MIND ALL THAT WE HAVE SAID, THE BEST TIME FOR SURGICAL NERVE REPAIR FROM THE THEORETICAL POINT OF VIEW SHOULD BE IN EMERGENCY FOR DISTAL LESIONS AND DELAYED (20 DAYS) FOR PROXIMAL SEVERANCES THAT REQUIRE THE RECONSTRUCTION OF A GREATER AMOUNT OF CYTOSKELETON. DELAYED REPAIR ALLOWS ALSO THE REMOVAL OF THE SCAR FORMED IN THE MEANWHILE AND TO CONNECT A PROXIMAL STUMP RICH IN NEOFORMED PROTEINS AND READY TO SEND TO PERIPHERY SOUND AXONAL SPROUTS. PRACTICALLY. HOWEVER, VARIOUS CONTINGENT CONSIDERATIONS WILL CONDITION THE CHOICE OF REPAIRING TIME |
| --- |

## Slide 60
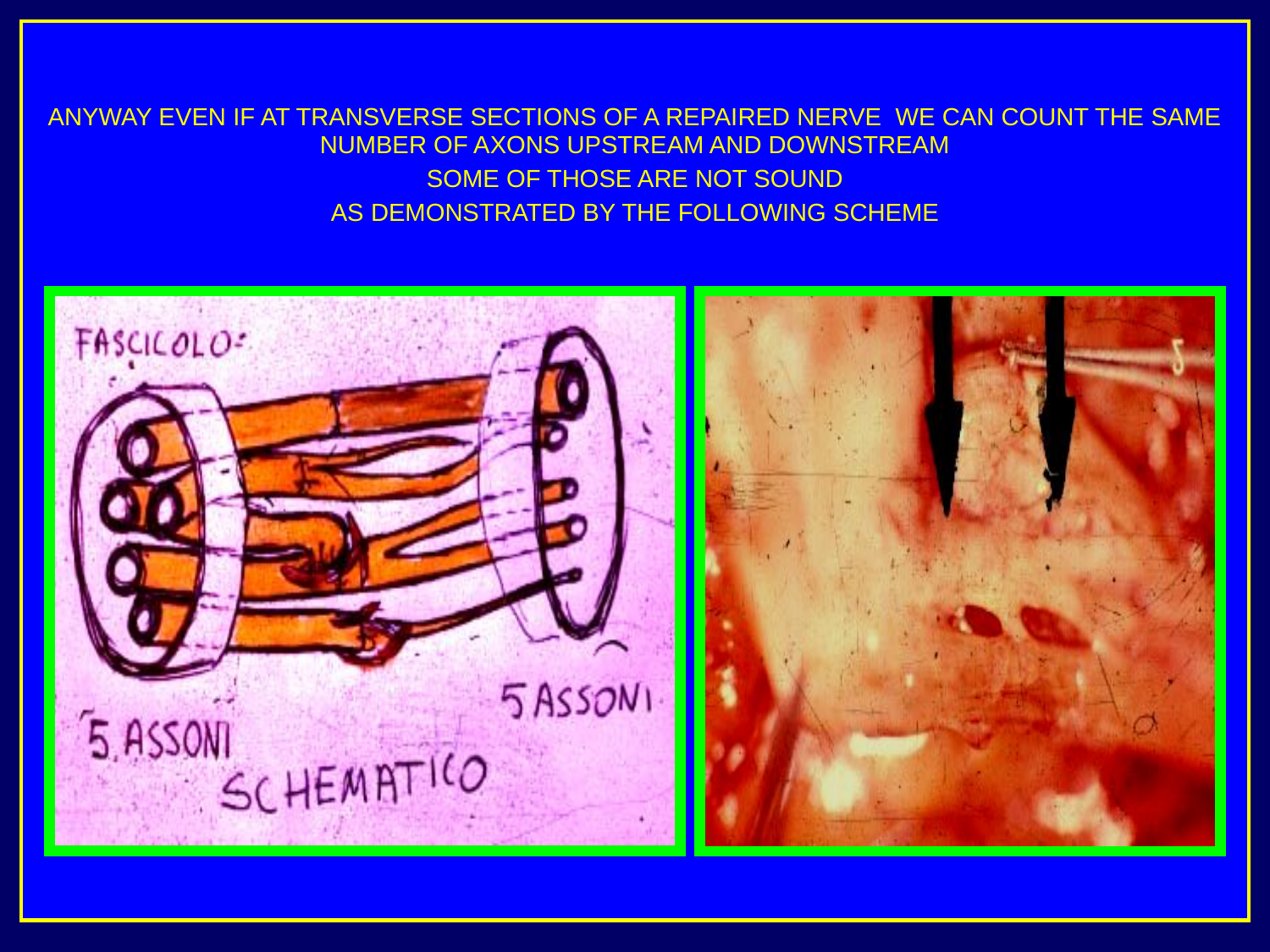

| ANYWAY EVEN IF AT TRANSVERSE SECTIONS OF A REPAIRED NERVE WE CAN COUNT THE SAME NUMBER OF AXONS UPSTREAM AND DOWNSTREAM SOME OF THOSE ARE NOT SOUND AS DEMONSTRATED BY THE FOLLOWING SCHEME |
| --- |

## Slide 61
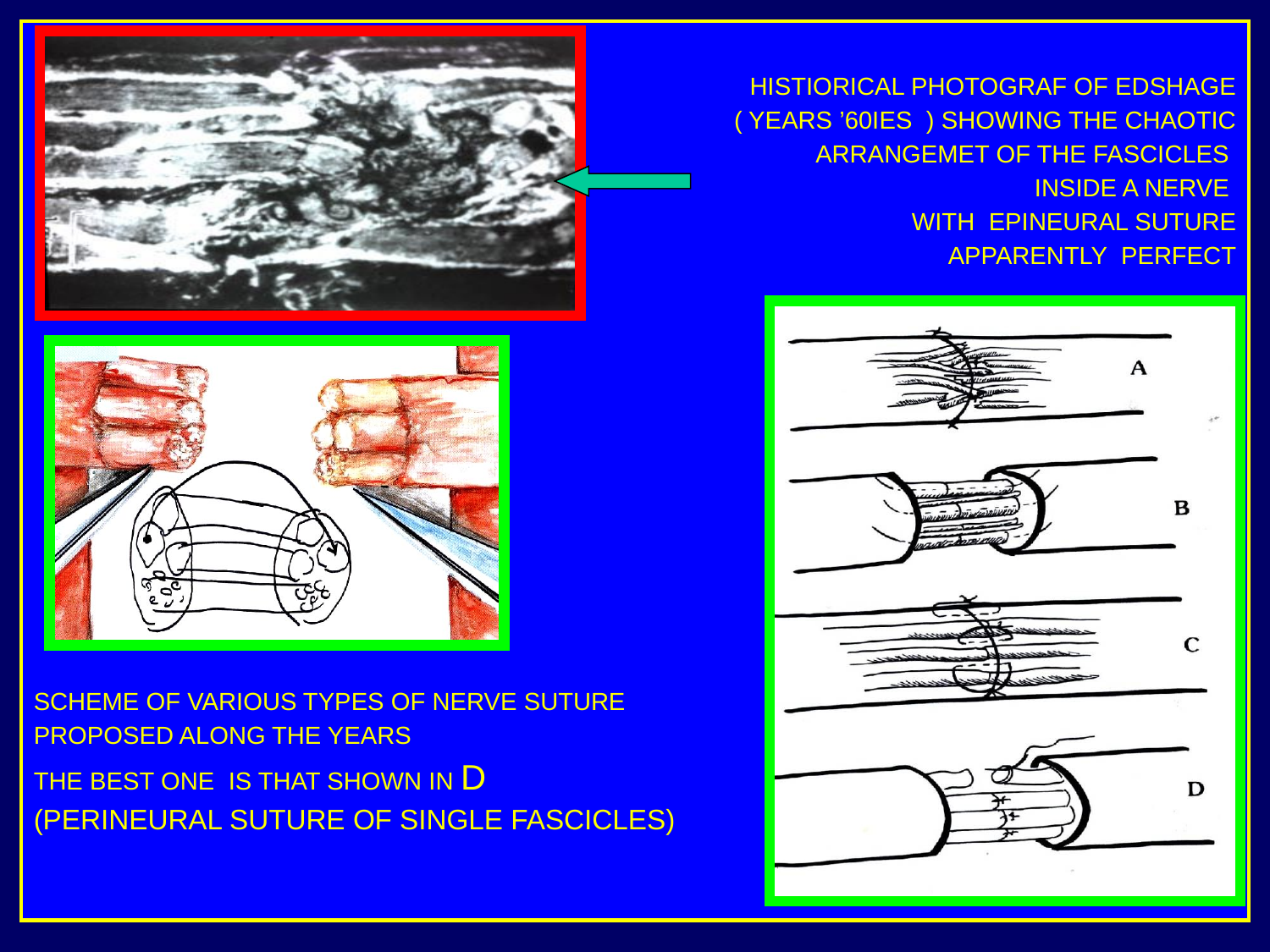

| HISTIORICAL PHOTOGRAF OF EDSHAGE ( YEARS ’60IES ) SHOWING THE CHAOTIC ARRANGEMET OF THE FASCICLES INSIDE A NERVE WITH EPINEURAL SUTURE APPARENTLY PERFECT SCHEME OF VARIOUS TYPES OF NERVE SUTURE PROPOSED ALONG THE YEARS THE BEST ONE IS THAT SHOWN IN D (PERINEURAL SUTURE OF SINGLE FASCICLES) |
| --- |

## Slide 62
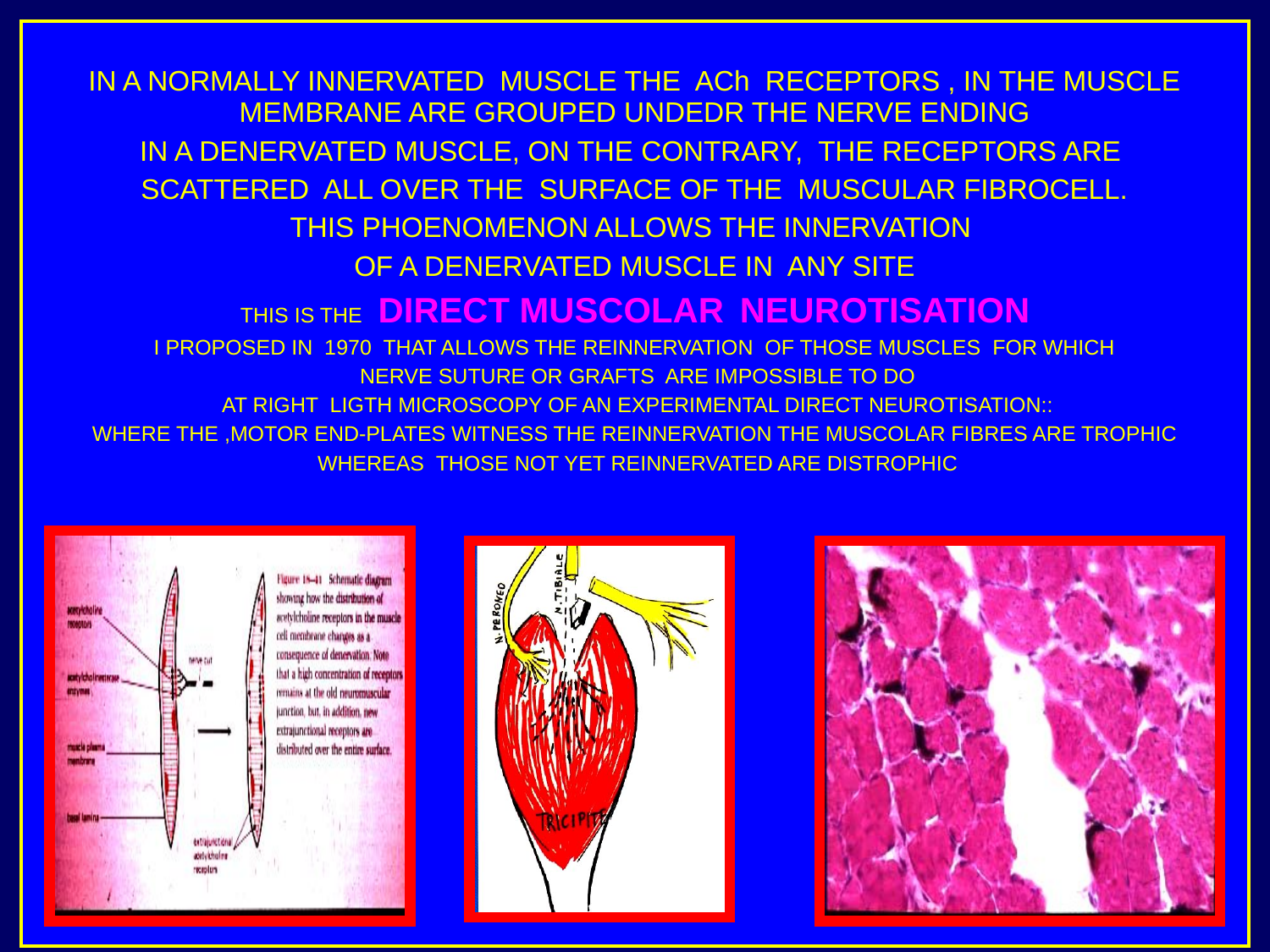

| IN A NORMALLY INNERVATED MUSCLE THE ACh RECEPTORS , IN THE MUSCLE MEMBRANE ARE GROUPED UNDEDR THE NERVE ENDING IN A DENERVATED MUSCLE, ON THE CONTRARY, THE RECEPTORS ARE SCATTERED ALL OVER THE SURFACE OF THE MUSCULAR FIBROCELL. THIS PHOENOMENON ALLOWS THE INNERVATION OF A DENERVATED MUSCLE IN ANY SITE THIS IS THE DIRECT MUSCOLAR NEUROTISATION I PROPOSED IN 1970 THAT ALLOWS THE REINNERVATION OF THOSE MUSCLES FOR WHICH NERVE SUTURE OR GRAFTS ARE IMPOSSIBLE TO DO AT RIGHT LIGTH MICROSCOPY OF AN EXPERIMENTAL DIRECT NEUROTISATION:: WHERE THE ,MOTOR END-PLATES WITNESS THE REINNERVATION THE MUSCOLAR FIBRES ARE TROPHIC WHEREAS THOSE NOT YET REINNERVATED ARE DISTROPHIC |
| --- |

## Slide 63
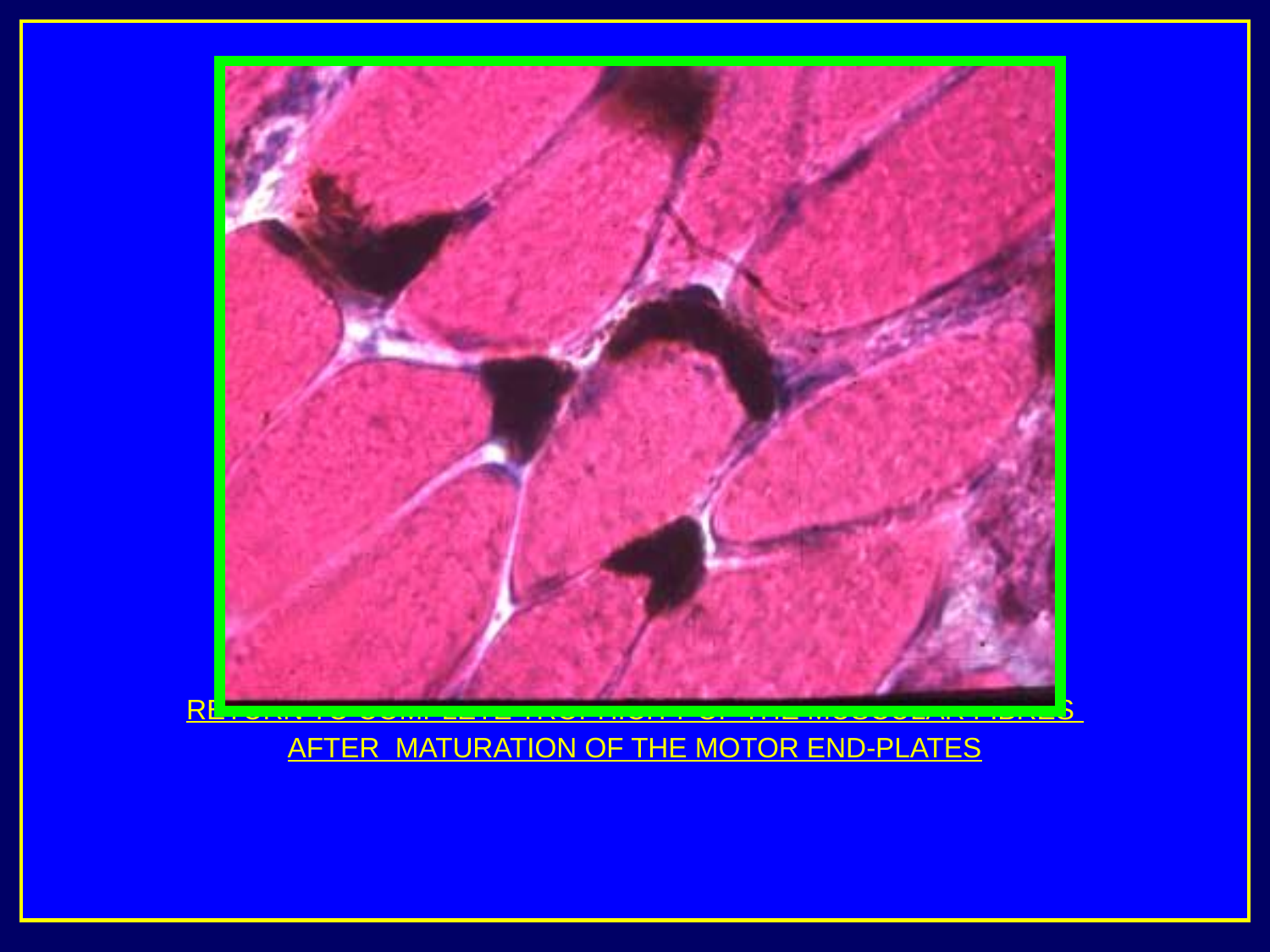

| RETURN TO COMPLETE TROPHICITY OF THE MUSCULAR FIBRES AFTER MATURATION OF THE MOTOR END-PLATES |
| --- |

## Slide 64
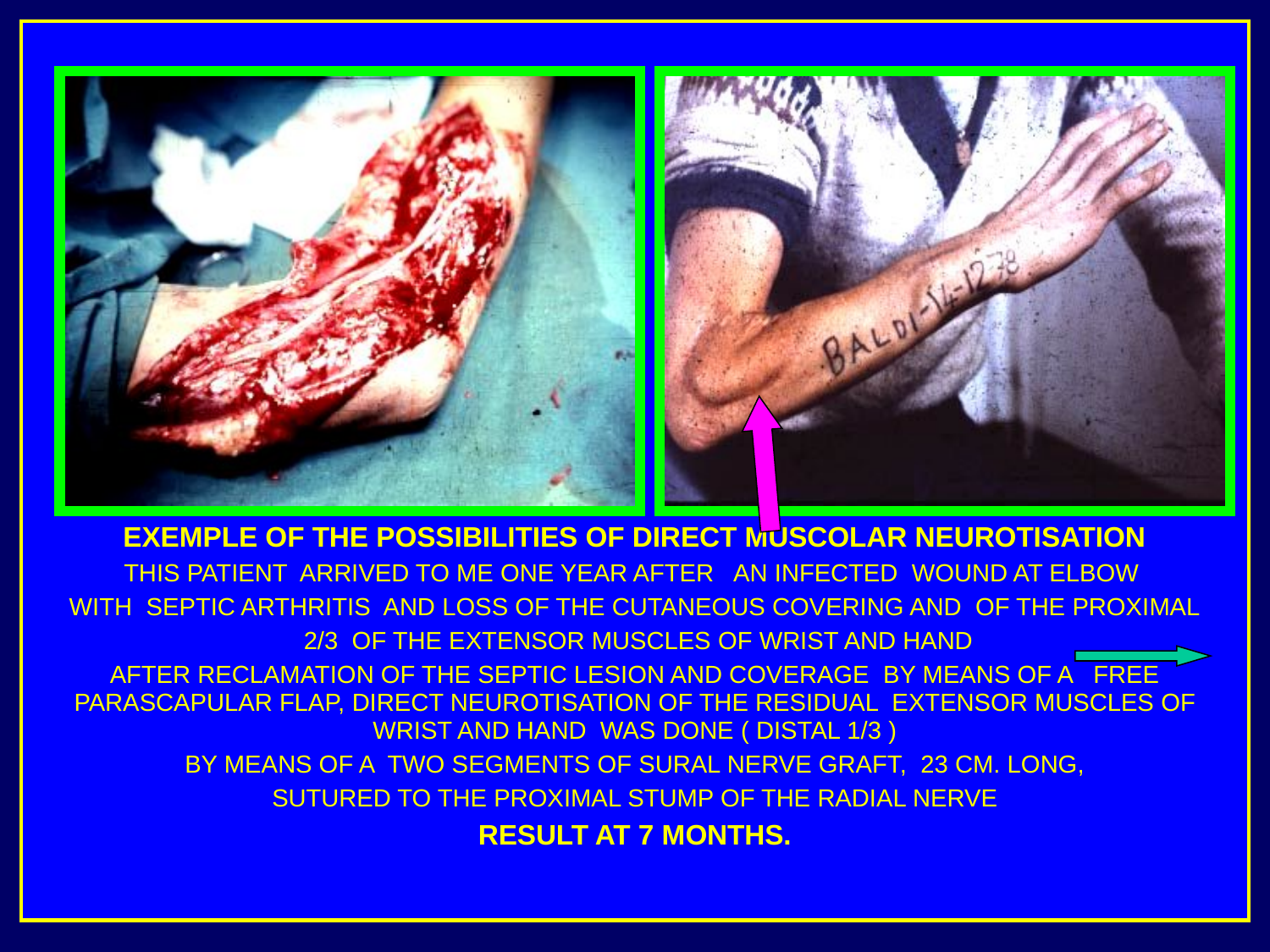

| EXEMPLE OF THE POSSIBILITIES OF DIRECT MUSCOLAR NEUROTISATION THIS PATIENT ARRIVED TO ME ONE YEAR AFTER AN INFECTED WOUND AT ELBOW WITH SEPTIC ARTHRITIS AND LOSS OF THE CUTANEOUS COVERING AND OF THE PROXIMAL 2/3 OF THE EXTENSOR MUSCLES OF WRIST AND HAND AFTER RECLAMATION OF THE SEPTIC LESION AND COVERAGE BY MEANS OF A FREE PARASCAPULAR FLAP, DIRECT NEUROTISATION OF THE RESIDUAL EXTENSOR MUSCLES OF WRIST AND HAND WAS DONE ( DISTAL 1/3 ) BY MEANS OF A TWO SEGMENTS OF SURAL NERVE GRAFT, 23 CM. LONG, SUTURED TO THE PROXIMAL STUMP OF THE RADIAL NERVE RESULT AT 7 MONTHS. |
| --- |

## Slide 65
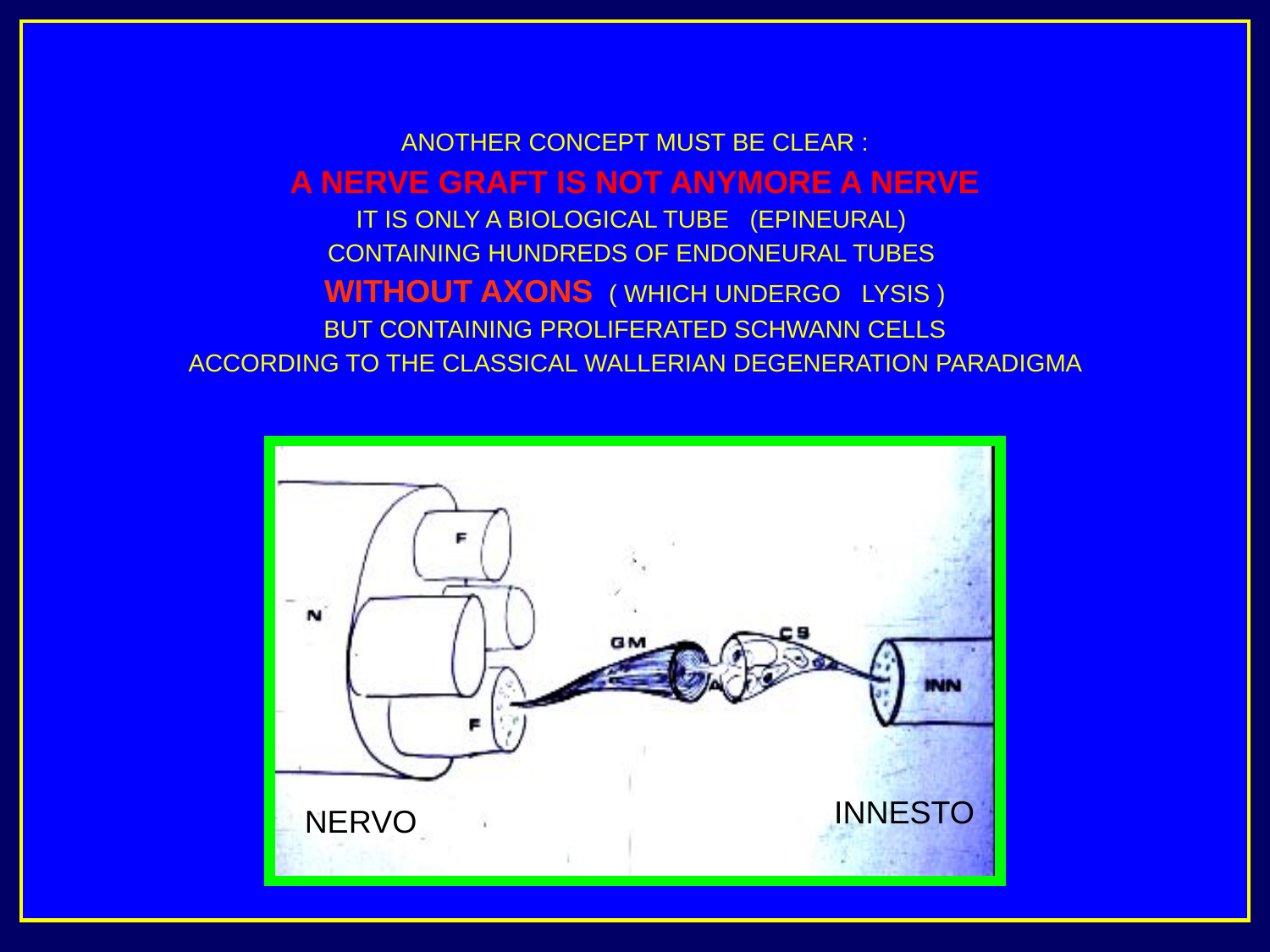

| ANOTHER CONCEPT MUST BE CLEAR : A NERVE GRAFT IS NOT ANYMORE A NERVE IT IS ONLY A BIOLOGICAL TUBE (EPINEURAL) CONTAINING HUNDREDS OF ENDONEURAL TUBES WITHOUT AXONS ( WHICH UNDERGO LYSIS ) BUT CONTAINING PROLIFERATED SCHWANN CELLS ACCORDING TO THE CLASSICAL WALLERIAN DEGENERATION PARADIGMA |
| --- |
 INNESTO
NERVO

## Slide 66
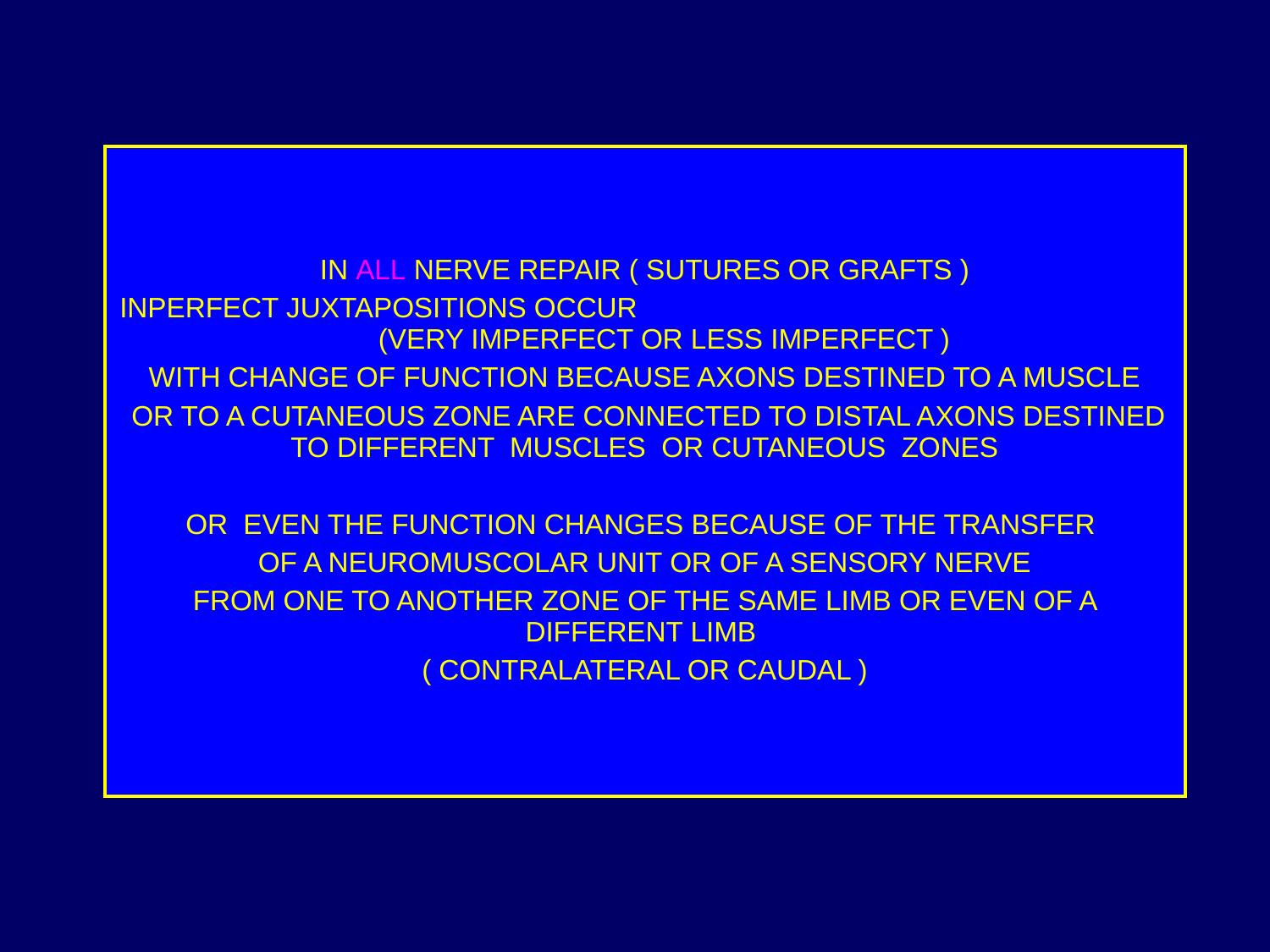

| IN ALL NERVE REPAIR ( SUTURES OR GRAFTS ) INPERFECT JUXTAPOSITIONS OCCUR (VERY IMPERFECT OR LESS IMPERFECT ) WITH CHANGE OF FUNCTION BECAUSE AXONS DESTINED TO A MUSCLE OR TO A CUTANEOUS ZONE ARE CONNECTED TO DISTAL AXONS DESTINED TO DIFFERENT MUSCLES OR CUTANEOUS ZONES OR EVEN THE FUNCTION CHANGES BECAUSE OF THE TRANSFER OF A NEUROMUSCOLAR UNIT OR OF A SENSORY NERVE FROM ONE TO ANOTHER ZONE OF THE SAME LIMB OR EVEN OF A DIFFERENT LIMB ( CONTRALATERAL OR CAUDAL ) |
| --- |

## Slide 67
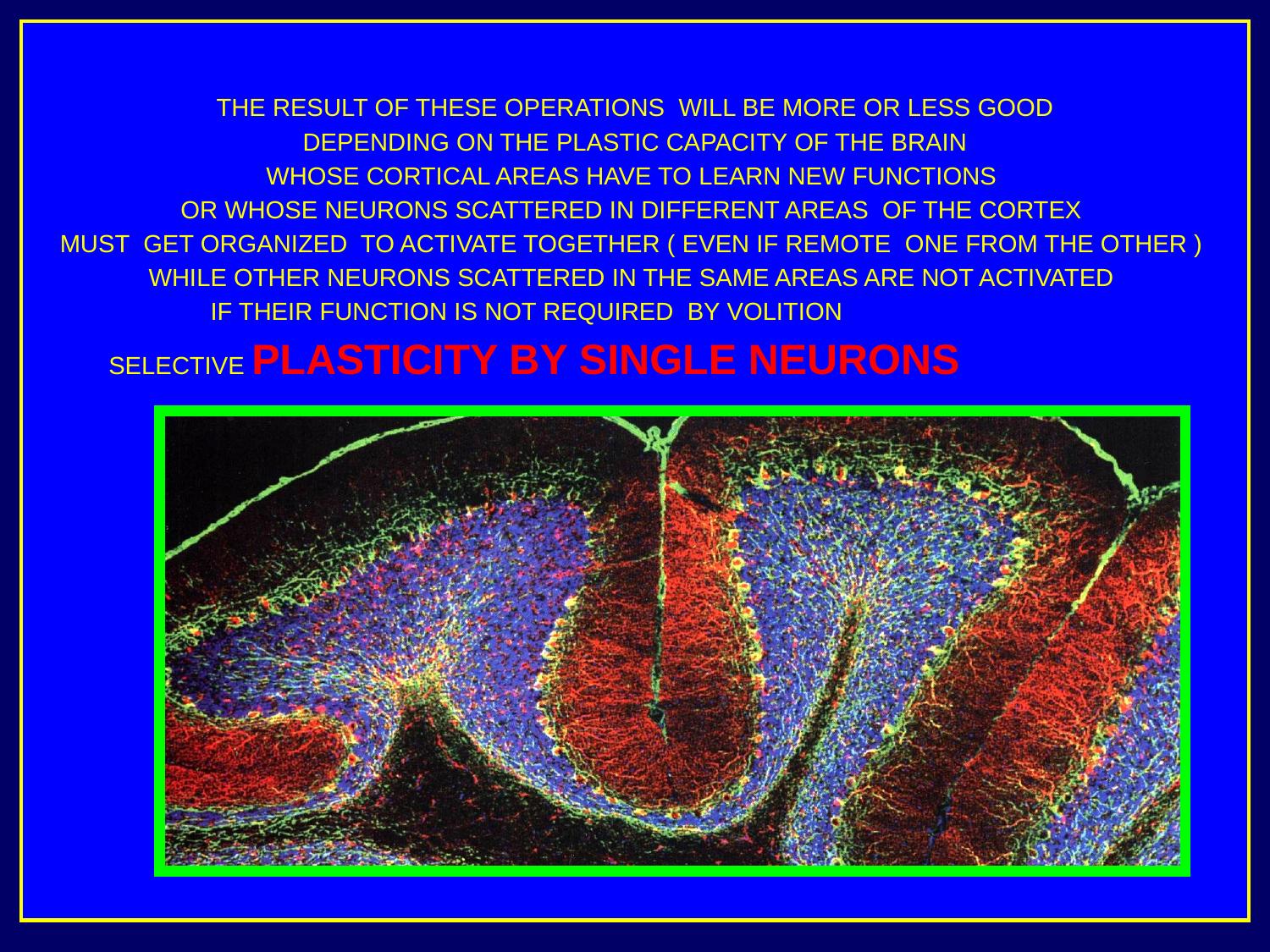

| THE RESULT OF THESE OPERATIONS WILL BE MORE OR LESS GOOD DEPENDING ON THE PLASTIC CAPACITY OF THE BRAIN WHOSE CORTICAL AREAS HAVE TO LEARN NEW FUNCTIONS OR WHOSE NEURONS SCATTERED IN DIFFERENT AREAS OF THE CORTEX MUST GET ORGANIZED TO ACTIVATE TOGETHER ( EVEN IF REMOTE ONE FROM THE OTHER ) WHILE OTHER NEURONS SCATTERED IN THE SAME AREAS ARE NOT ACTIVATED IF THEIR FUNCTION IS NOT REQUIRED BY VOLITION SELECTIVE PLASTICITY BY SINGLE NEURONS |
| --- |

## Slide 68
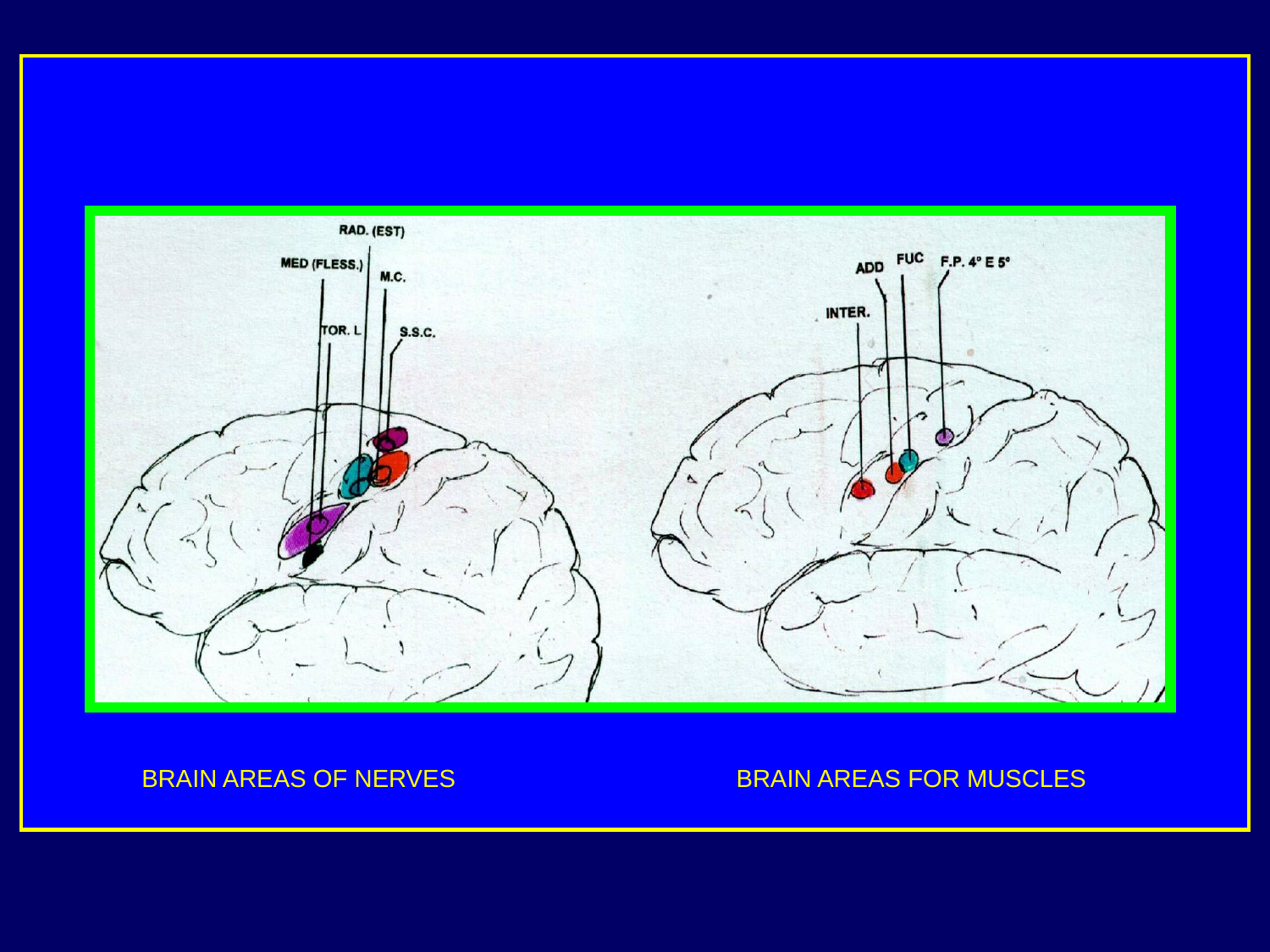

| |
| --- |
 BRAIN AREAS OF NERVES BRAIN AREAS FOR MUSCLES

## Slide 69
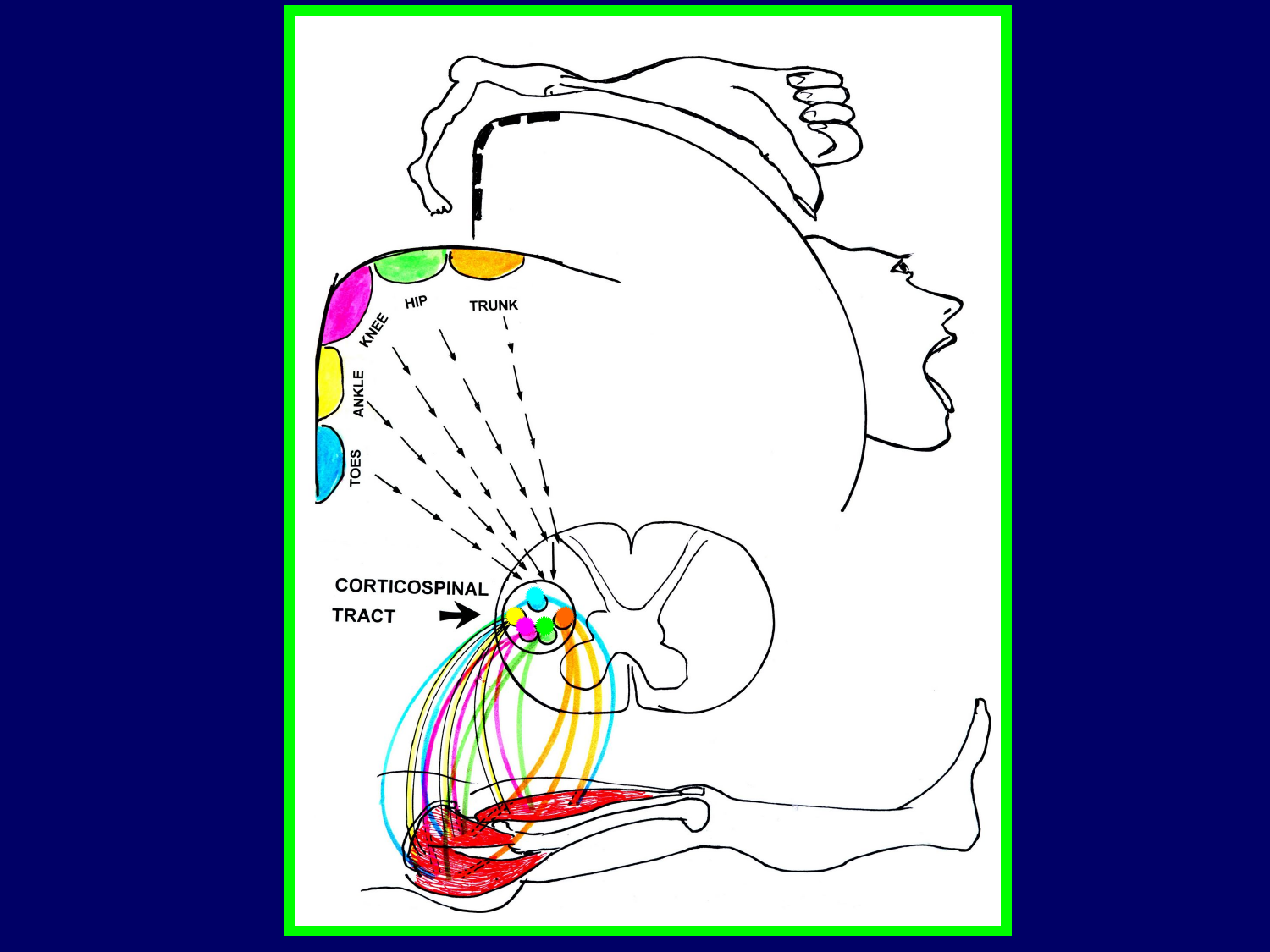

## Slide 70
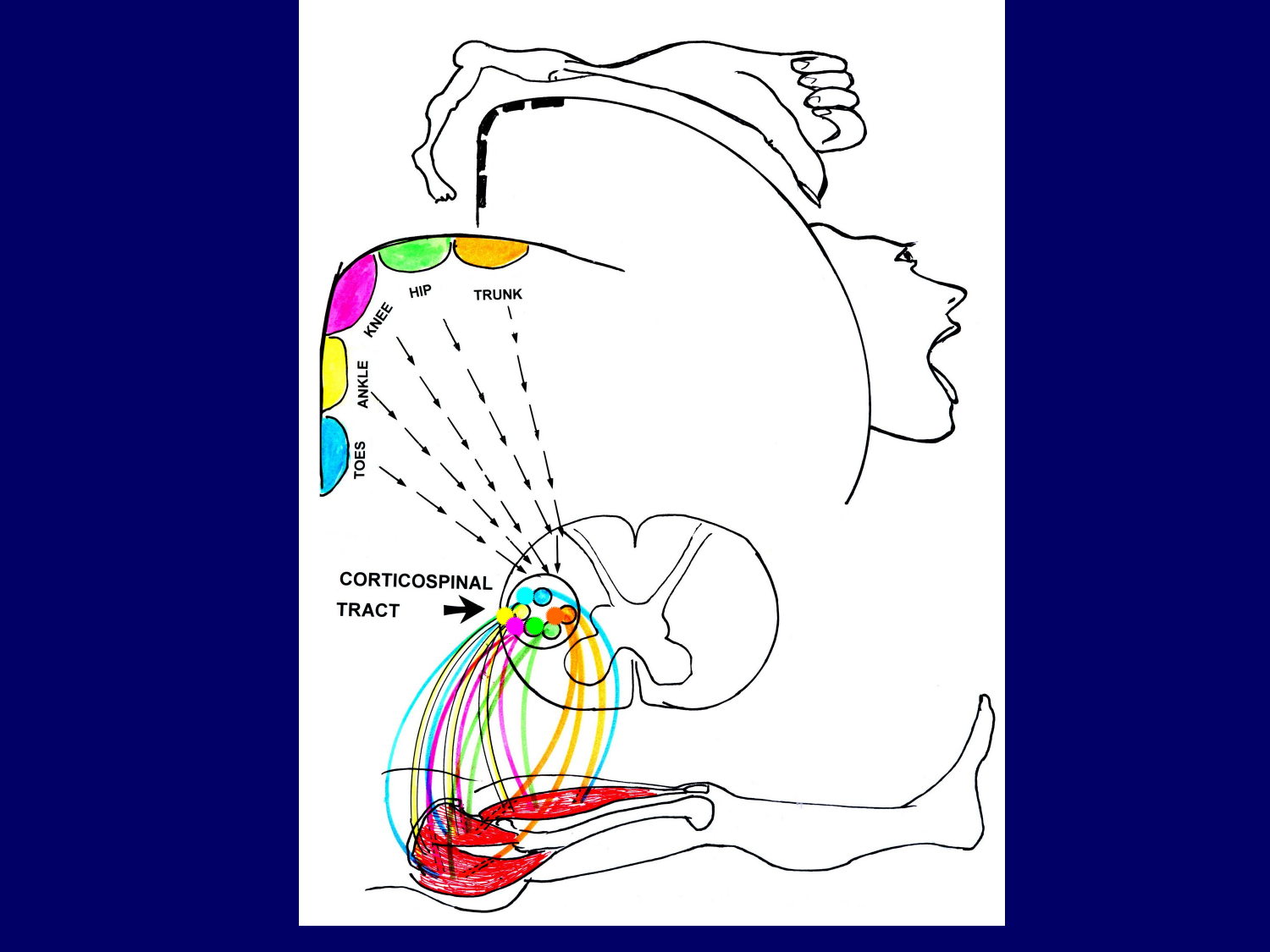

## Slide 71
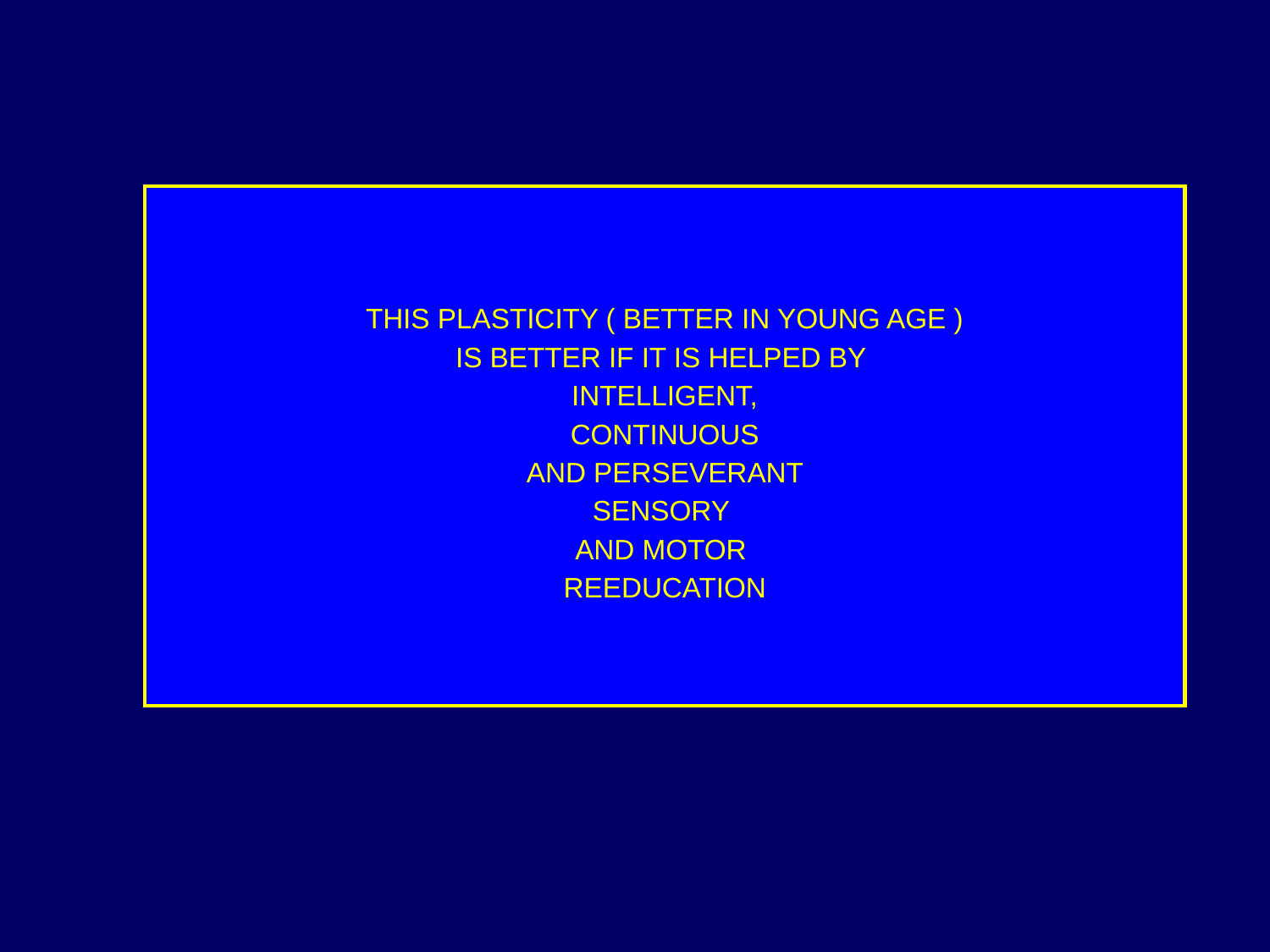

| THIS PLASTICITY ( BETTER IN YOUNG AGE ) IS BETTER IF IT IS HELPED BY INTELLIGENT, CONTINUOUS AND PERSEVERANT SENSORY AND MOTOR REEDUCATION |
| --- |

## Slide 72
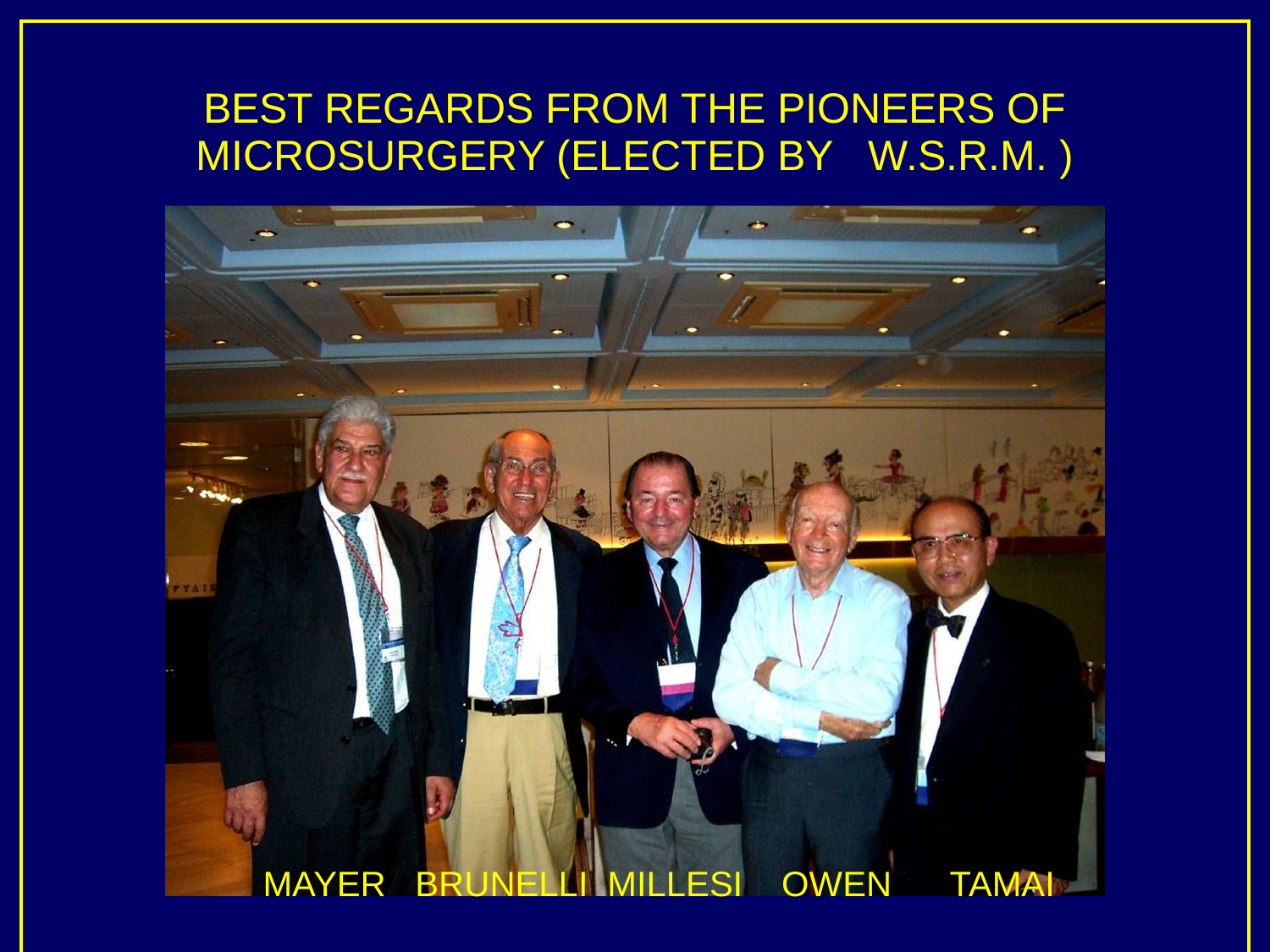

| BEST REGARDS FROM THE PIONEERS OF MICROSURGERY (ELECTED BY W.S.R.M. ) |
| --- |
 MAYER BRUNELLI MILLESI OWEN TAMAI

## Slide 73
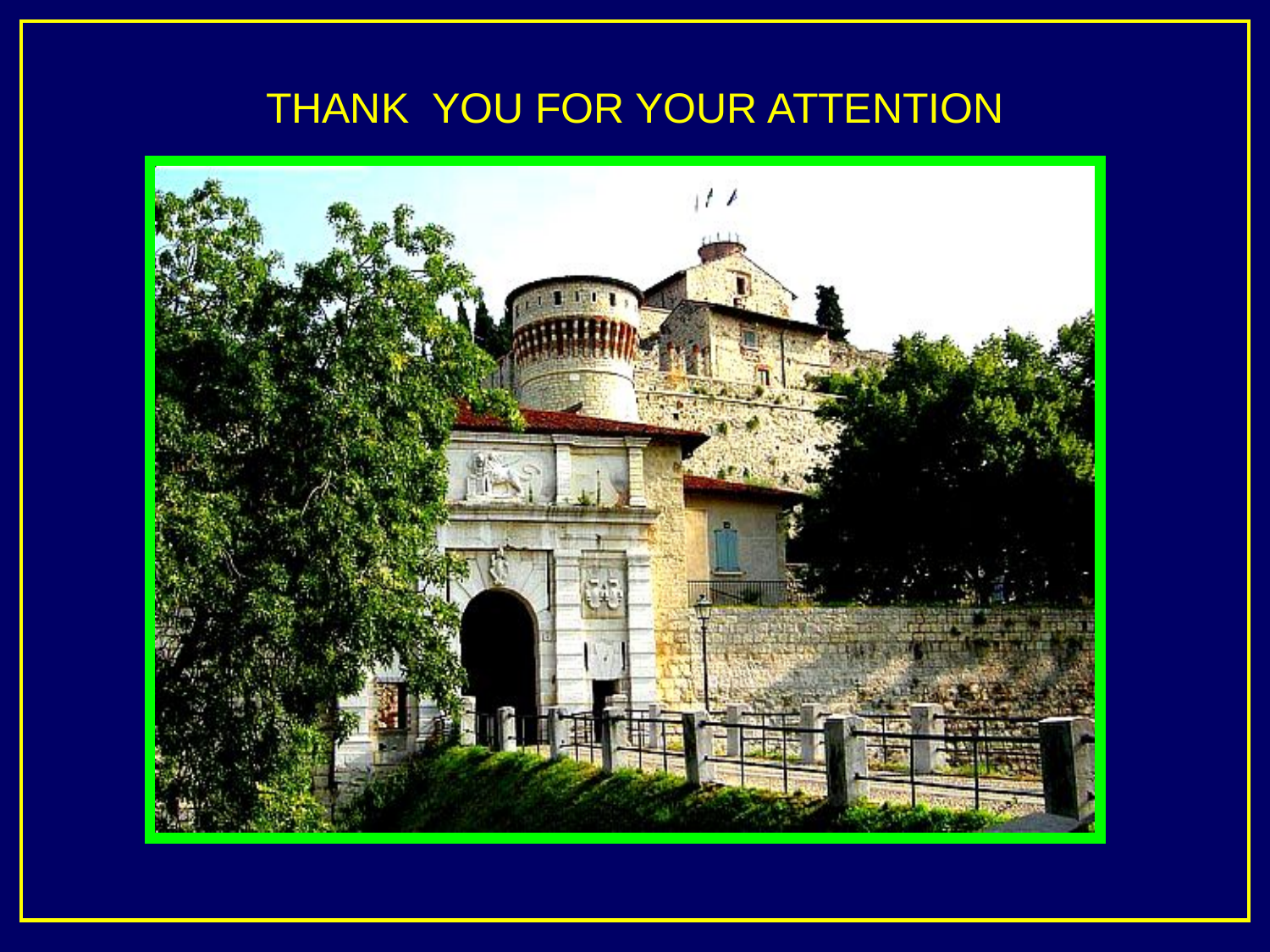

| THANK YOU FOR YOUR ATTENTION THE VENETIAN CASTLE OF BRESCIA |
| --- |
